# PD-1 is conserved from sharks to humans: new insights into PD-1, PD-L1, PD-L2, and SHP-2 evolution

**DOI:** 10.3389/fimmu.2025.1573492

**Published:** 2025-05-28

**Authors:** Ryohei Kondo, Kohei Kondo, Kei Nabeshima, Akihiko Nishikimi, Yasumasa Ishida, Toshiaki Shigeoka, Johannes M. Dijkstra

**Affiliations:** ^1^ Biosafety Division, Research Institute, National Center for Geriatrics and Gerontology, Obu, Aichi, Japan; ^2^ Antimicrobial Resistance Research Center, National Institute of Infectious Diseases, Higashimurayama, Tokyo, Japan; ^3^ Biodiveristy Division, National Institute for Environmental Studies, Tsukuba, Japan; ^4^ Division of Biological Science, Nara Institute of Science and Technology, Nara, Japan; ^5^ Center for Medical Science, Fujita Health University, Toyoake, Aichi, Japan

**Keywords:** PD-1, PD-L1, PD-L2, SHP-1, SHP-2, SHP-2L, evolution, fish

## Abstract

Programmed cell death protein 1 (PD-1) is an immune checkpoint molecule until recently believed to exist only in tetrapod species. However, together with a very recent study dedicated to the CD28/CTLA4 molecule family, this study—using database information—identifies the *PD-1* gene in both bony and cartilaginous fish, while being the first to present a detailed molecular analysis of the evolution of PD-1 and its ligands. Conserved sequence motifs imply an ancient origin of PD-1’s binding modes to its extracellular ligand PD-L1 and its intracellular ligand Src homology region 2 domain-containing phosphatase-2 (SHP-2), and also of its N116 glycosylation motif—a less well known PD-1 feature—important for binding galectins. The PD-1 cytoplasmic tail binds SHP-2 by two motifs, defined as an immunoreceptor tyrosine-based inhibitory motif (ITIM) and immunoreceptor tyrosine-based switch motif (ITSM), but sequence conservation patterns show that these definitions warrant a discussion. As in mammals, *PD-1* transcripts in fish could be found co-expressed with markers of regulatory and exhausted T cells, suggesting a similar immune checkpoint function. Agreeing with previous reports, the *PD-L1*/*PD-L2* gene duplication was only found in tetrapod species, while we newly discovered that features that consistently distinguish the two molecules are PD-L2 IgC domain motifs. Among PD-L1 (the name given to the single PD-L ancestral molecule) of many ray-finned fish, conservation of a very long cytoplasmic tail motif supports previous claims that PD-L1 cytoplasmic tails may have a function. Surprisingly, we found a gene similar to *SHP-2*—that we named *SHP-2-like* (*SHP-2L*)—to be conserved from sharks to mammals, although lost or inactivated in higher primates and rodents. SHP-2L is expected to bind PD-1 similar to SHP-2. This comparative analysis of PD-1 and its interacting molecules across jawed vertebrates highlights conserved immune checkpoint features while revealing new insights and lineage-specific adaptations.

## Introduction

*Programmed cell death 1* (*PD-1*) was identified in 1992 as a gene upregulated during apoptosis (1). Later studies, however, revealed PD-1 (CD279) as an “immune checkpoint” molecule involved in various regulatory processes, rather than being directly involved in apoptosis (2–4). The antibody targeting of PD-1 or its ligand PD-L1 have become well-established forms of cancer immunotherapy (5).

PD-1 is expressed by activated T cells, but also found in other immune cells like B cells and myeloid cells (2, 6, 7). The PD-1 immune regulatory function involves reductions in proliferation and activation of the PD-1 expressing cells, as shown for various T cells upon engagement of their PD-1 molecules by the PD-1 ligands 1 or 2 (PD-L1 or PD-L2) (3, 8, 9). *PD-1* knockout mice revealed the importance of PD1 in preventing autoimmune diseases (10, 11), and PD-1 is said to “put a brake” on immune activations.

PD-1 is a transmembrane signal receptor molecule that—together with CD28, cytotoxic T-lymphocyte associated protein 4 (CTLA-4), and inducible T cell co-stimulator (ICOS)—belongs to the CD28 family of immunoglobulin superfamily (IgSF) molecules (12). Generally, the ectodomains of these molecules interact with ligands of the B7 family, and their cytoplasmic tails participate in signaling through phosphorylation. In the case of PD-1, the B7 family ligands expressed on interacting cells are PD-L1 and PD-L2. The PD-1 cytoplasmic tail carries an immunoreceptor tyrosine-based inhibitory motif (ITIM) and an Immunoreceptor tyrosine-based switch motif (ITSM), which, once phosphorylated by a kinase such as LCK (13), together predominantly bind and activate Src homology region 2 domain-containing phosphatase-2 (SHP-2) but can also interact with SHP-1 (14, 15). For convenience, in this study we use similar names for the genes and the proteins, but alternative gene names are: *PD-1*, *PDCD1*; *PD-L1*, *PDCD1LG2*/*CD274*; *PD-L2*, *PDCD1LG2*; *SHP-1*, *Protein Tyrosine Phosphatase Non-receptor type 6* (*PTPN6*); *SHP-2*, *PTPN11*.

PD-L1 and PD-L2 are similar molecules (∼38% amino acid [aa] identity) with similar effects on PD-1-expressing cells, but PD-L1 is more widely expressed than PD-L2 that is predominantly found on myeloid cells (3, 16). Compared to the inhibitory CTLA-4/(CD80/CD86) system, which especially provides an immune checkpoint during immune response initiation in lymphoid tissues, the PD-1/PD-L1 system is considered more important in the periphery where PD-1 can engage with PD-L1 on non-immune cells such as epithelial cells, endothelial cells, or tumor cells (16, 17).

The immune systems among all jawed vertebrates, already from the level of Chondrichthyes (cartilaginous fish including sharks), are quite similar in regard to major cell types and molecules (18–21). Therefore, it has been puzzling that earlier studies, despite active searching, did not find fish *PD-1* (12, 22, 23). However, this mostly arose from attempts to identify *PD-1* in teleost (modern bony) fish through a direct comparison with mammals, which was hampered by the teleost PD-1 sequence being highly diverged and rearrangements of the genomic region. A very recent study by Quiniou et al. (24)—published during the preparation of our manuscript—circumvented this problem in a similar way as we did, namely by first identifying *PD-1* in more basal fish groups like sharks and then find teleost *PD-1* from there. However, as their study was addressed to the broader CD28/CTLA4 family, their analysis of PD-1 molecular evolution was relatively superficial.

As a note, for language simplicity and as done by most researchers, in this article we use words like “primitive/basal” or “modern/higher” to describe species clades with sets of characteristic features that are more or less ancient, respectively. However, we are aware that all extant species share an equally long evolution (25). For the readers’ convenience, **Supplementary File 1A** provides a phylogenetic time tree depicting evolutionary relationships of the species clades discussed here.

The present study describes that *PD-1*, similar to *PD-L1* (*PD-L* would be a better name for this single ancestral gene, but the name was already given), is well-conserved throughout different clades of fishes, and compares the molecular motifs in detail. We also provide new insights into PD-L1 versus PD-L2 evolution. Furthermore, surprisingly, a second ancient *SHP-2* gene was found, which is well conserved throughout most jawed vertebrates but lost in higher primates and rodents.

## Methods

### Identification of genes and analysis of nucleotide and amino acid sequences

For identifying *PD-1* and other genes in various species, we used a combination of blast similarity searches against NCBI databases (26); available at https://www.ncbi.nlm.nih.gov), gene predictions by “FGenesh” software (27); available at http://www.softberry.com), and sequence alignments and comparisons (28). Deduced PD-1, PD-L1, PD-L2, SHP-1, SHP-2, and SHP-2L sequences with their sources are listed in **Supplementary File 1B**. Leader peptides were predicted using “SignalP 5.0” software (29); available at http://www.cbs.dtu.dk/services/SignalP/). For comparing genomic organization in different species (gene synteny), the following NCBI datasets were utilized: *Homo sapiens* (GRCh38.p14), *Bos taurus* (ARS-UCD2.0), *Ornithorhynchus anatinus* (mOrnAna1.pri.v4), *Xenopus tropicalis* (UCB_Xtro_10.0), *Protopterus annectens* (PAN1.0), *Scyliorhinus canicula* (ScyCan1.1), *Polypterus senegalus* (ASM1683550v1), *Danio rerio* (GRCz11).

### Analysis and predictions of protein structure

For structural predictions of the structures of, and interactions between, the tarpon PD-1 IgSF domain and PD-L1 membrane-distal IgSF domain (for regions see **Supplementary File 1B**), the MultiFOLD server was used (30); available https://www.reading.ac.uk/bioinf/MultiFOLD/). For making structural superimpositions and display of structures, we used “The PyMOL Molecular Graphics System, Version 2.0” (Schrödinger, LLC; available at https://pymol.org/2/).

### Expression analysis using single-cell RNA sequencing datasets

For the alignment of fish datasets, FASTQ files were processed using the Cell Ranger pipeline (version 7.2.0, 10x Genomics) (31). Reference genome FASTA files (Atlantic salmon: GCF_905237065.1_Ssal_v3.1; Zebrafish: danRer11; Nurse shark: GCF_021869965.1, derived from the Rhincodon typus genome) and gene annotation GTF files (Atlantic salmon: GCF_905237065.1_Ssal_v3.1; Zebrafish: danRer11.ncbiRefSeq; Nurse shark: GCF_021869965.1, derived from the Rhincodon typus genome) were used to generate custom reference packages for each species using the cellranger mkref command. The genome FASTA and gene annotation files were manually modified to include custom genes (32) where necessary. For the analysis of mammalian datasets, count matrix files were downloaded from the GEO database. Clustering of scRNA-seq data was conducted using the Seurat R package (version 5.0.2) (33) in R version 4.3.2. T cell clusters were identified based on the expression of T cell markers (*CD3*, *CD4*, and *CD8*). For correlation analysis, spleen cells with more than 2,000 unique molecular identifiers (UMIs) were included. Pearson’s correlation scores between the expression levels of *PD-1* and those of all other genes were calculated using the scaled count matrix generated by Seurat.

The raw FASTQ files for the analyzed fish datasets were obtained from the GEO database (https://www.ncbi.nlm.nih.gov/geo/) under the following accession numbers: Atlantic salmon spleen, GSE252828 (34); zebrafish spleen, GSE186158 (35); and nurse shark spleen, GSE232302 (36). The count matrix files for the mammalian datasets were also retrieved from the GEO database under the following accession numbers: mouse spleen, GSE132901 (37); and cattle PBMC, GSE166245 (38).

## Results

### 1 Identification of fish PD-1 gene in conserved genomic locations

Identifying *PD-1* in Chondrichthyes (cartilaginous fish like sharks and rays) was simply done by finding their deduced PD-1 sequences as top hits for known PD-1 sequences by BLASTP homology searches at NCBI. In some cases automatic programs had already named these molecules PD-1 (e.g., see GenBank accession XP_032887962 for the thorny skate *Amblyraja radiata*). Finding *PD-1* in ray-finned fish was more difficult because of the degeneration of ectodomain motifs, but homology searches did pick up the highly conserved motifs in the cytoplasmic tails of—what proved to be—proper *PD-1* genes.

**Figure 1:**
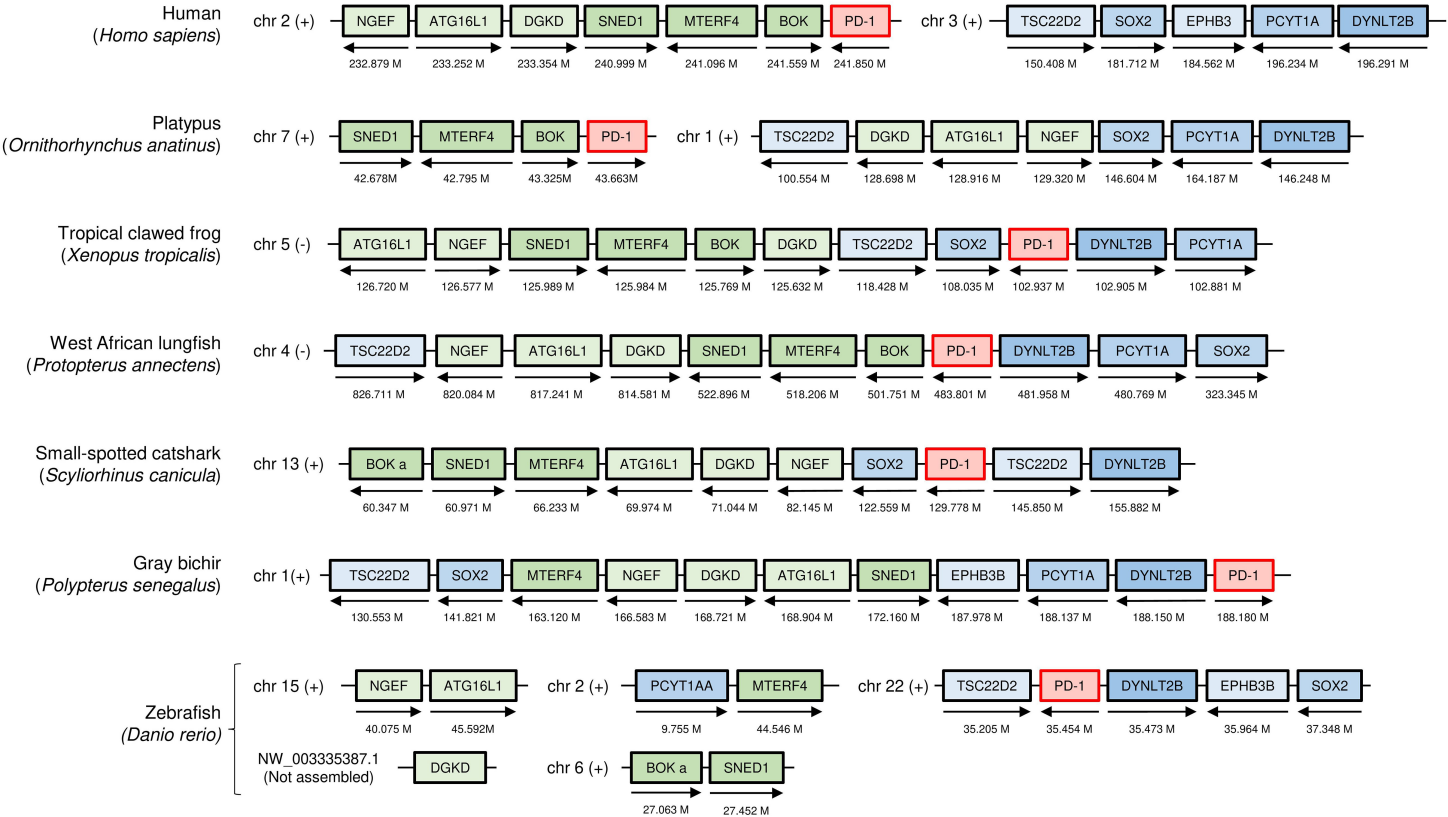
The genomic locations of *PD-1* genes from shark to human, compared in seven representative species. Selected genes and their orthologues in other species that in human are linked to *PD-1* on chromosome 2 are indicated as green boxes: *neuronal guanine nucleotide exchange factor* (*NGEF*); *autophagy related 16 like 1* (*ATG16L1*); *diacylglycerol kinase delta* (*DGKD*); *sushi, nidogen and EGF like domains 1* (*SNED1*); *mitochondrial transcription termination factor 4* (*MTERF4*); and *BCL2 family apoptosis regulator* (*BOK*). Relevant genes located on human chromosome 3 are indicated as blue boxes: *TSC22 domain family member 2* (*TSC22D2*); *dynein light chain Tctex-type 2B* (*DYNLT2B*); *eph receptor B3b* (*EPHB3B*); *SRY-box transcription factor 2* (*SOX2*); *and phosphate cytidylyltransferase 1A* (*PCYT1A*). The arrows indicate the gene direction. Genes are not necessarily neighbors, and the locations on the chromosome are indicated in Mb. chr, chromosome; M, Mb; (+), forward relationship (–); reverse relationship.

When directly comparing *PD-1* genomic regions between mammals and teleost fish (modern bony fish, e.g., zebrafish), past chromosomal translocation events obscure recognition of regional similarity (**Figure 1**). However, when primitive species of the ray-finned fish lineage and the tetrapod lineage, here exemplified by gray bichir (*Polypterus senegalus*) and tropical clawed frog (*Xenopus tropicalis*), are compared, their similarity in *PD-1* genomic regions (gene synteny) becomes immediately apparent (**Figure 1**). Also in cartilaginous fish, though at different distances because of interchromosomal genomic rearrangements, a similar set of genes is linked to *PD-1*, as exemplified in **Figure 1** by small-spotted catshark.

### Conservation of PD-1 sequence motifs for binding of PD-L1 and SHP-2

**Figure 2:**
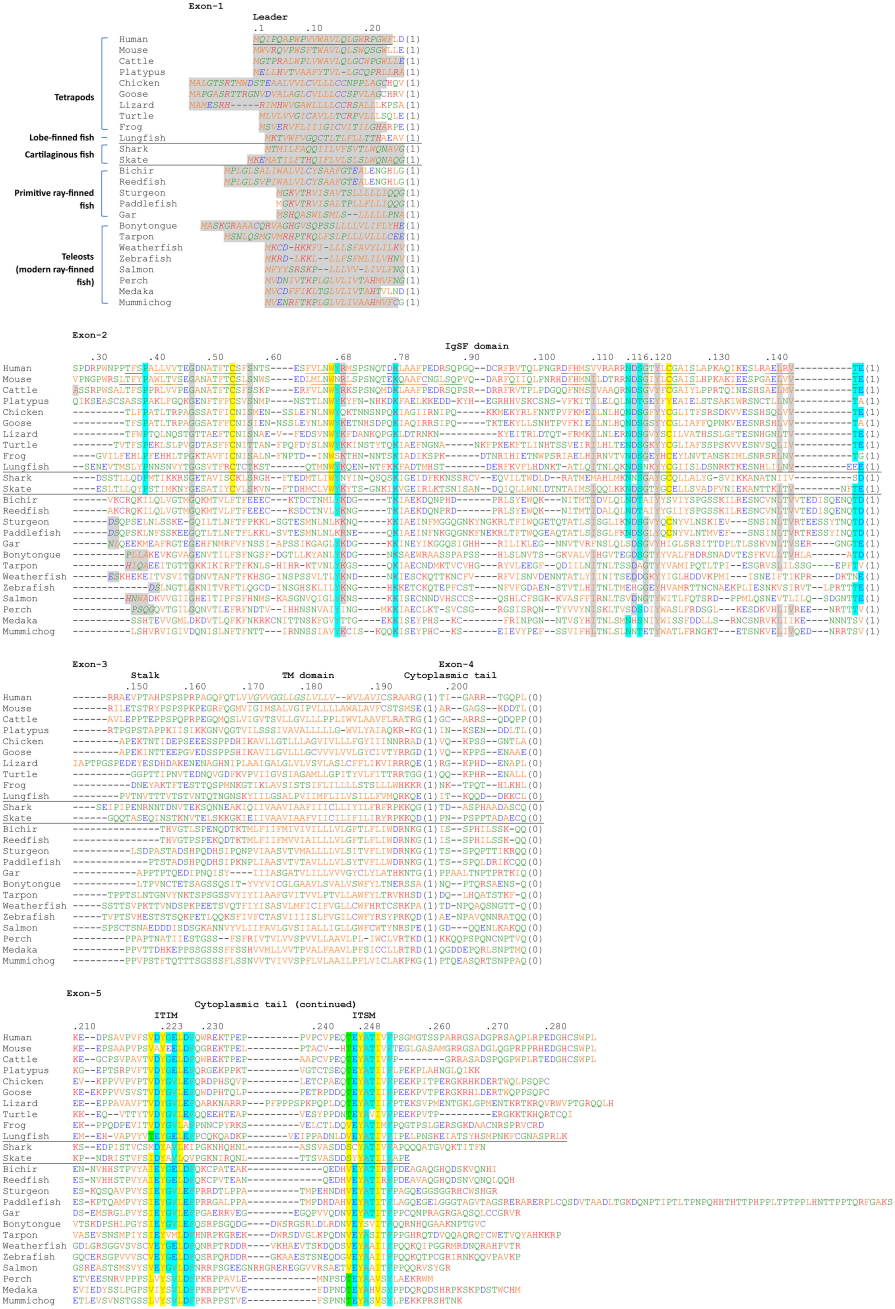
Alignment of deduced PD-1 amino acid sequences in representative species of Sarcopterygii (tetrapods and lobe-finned fishes), Condrichthyes (cartilaginous fish), and Actinopterygii (ray-finned fish). The (predicted) leader sequences are shown in Italic font and gray shading. In the IgSF domain, colored shading indicates: yellow, the most characteristic residues of IgSF domains; gray, other characteristic IgSF V category residues; cyan, residues more typical of PD-1 from shark to human. In the cytoplasmic tail, colored shading indicates: yellow, residues matching ITIM and ITSM definitions (S/I/V/L)xYxx(I/V/L) and TxYxx(V/I), respectively, except for the threonine at position -2 in the ITSM motif which is colored green; cyan, other residues of the (I/V)(D/E)Y(A/G)(E/V)L(D/E)F and (T/V)EYATIx(F/Y) motifs. The sequences and their sources are shown in **Supplementary File 1B**. Residue numbering above the alignment follows the human PD-1 protein. The numbers between brackets refer to introns and to their phases at the indicated position (0) or in the preceding codon (1,2) in the corresponding genomic sequences. Cysteines are in purple and, based on Hopp and Woods, 1981 (82): red font is used for basic residues, blue for acidic residues, and of the other residues (green and orange) the more hydrophilic ones are in green. Underlining of human and mouse PD-1 strecthes in the ectodomain indicates β-strands (following PDB accessions 3RRQ and 1NPU), and the indication of the human transmembrane region follows UniProt accession Q15116. The species are: human (*Homo sapiens*), mouse (*Mus musculus*), cattle (*Bos taurus*), platypus (*Ornithorhynchus anatinus*), chicken (*Gallus gallus*), goose (swan goose; *Anser cygnoides domesticus*), lizard (green anole lizard; *Anolis carolinensis*), turtle (green sea turtle; *Chelonia mydas*), frog (tropical clawed frog; *Xenopus tropicalis*), lungfish (West African Lungfish (*Protopterus annectens*), shark (small-spotted catshark; *Scyliorhinus canicula*), skate (thorny skate; *Amblyraja radiata*), bichir (gray bichir (*Polypterus senegalus*), reedfish (reedfish; *Erpetoichthys calabaricus*), sturgeon (sterlet sturgeon; *Acipenser ruthenus*), paddlefish (Mississippi paddlefish; *Polyodon* sp*athula*), gar (spotted gar; *Lepisosteus oculatus*), bonytongue (Asian bonytongue; *Scleropages formosus*), tarpon (*Megalops atlanticus*), weatherfish (oriental weatherfish; *Misgurnus anguillicaudatus*), zebrafish (*Danio rerio*), salmon (Atlantic salmon; *Salmo salar*), perch (Barramundi perch; *Lates calcarifer*), medaka (*Oryzias latipes*), mummichog (*Fundulus heteroclitus*).

**Figure 3:**
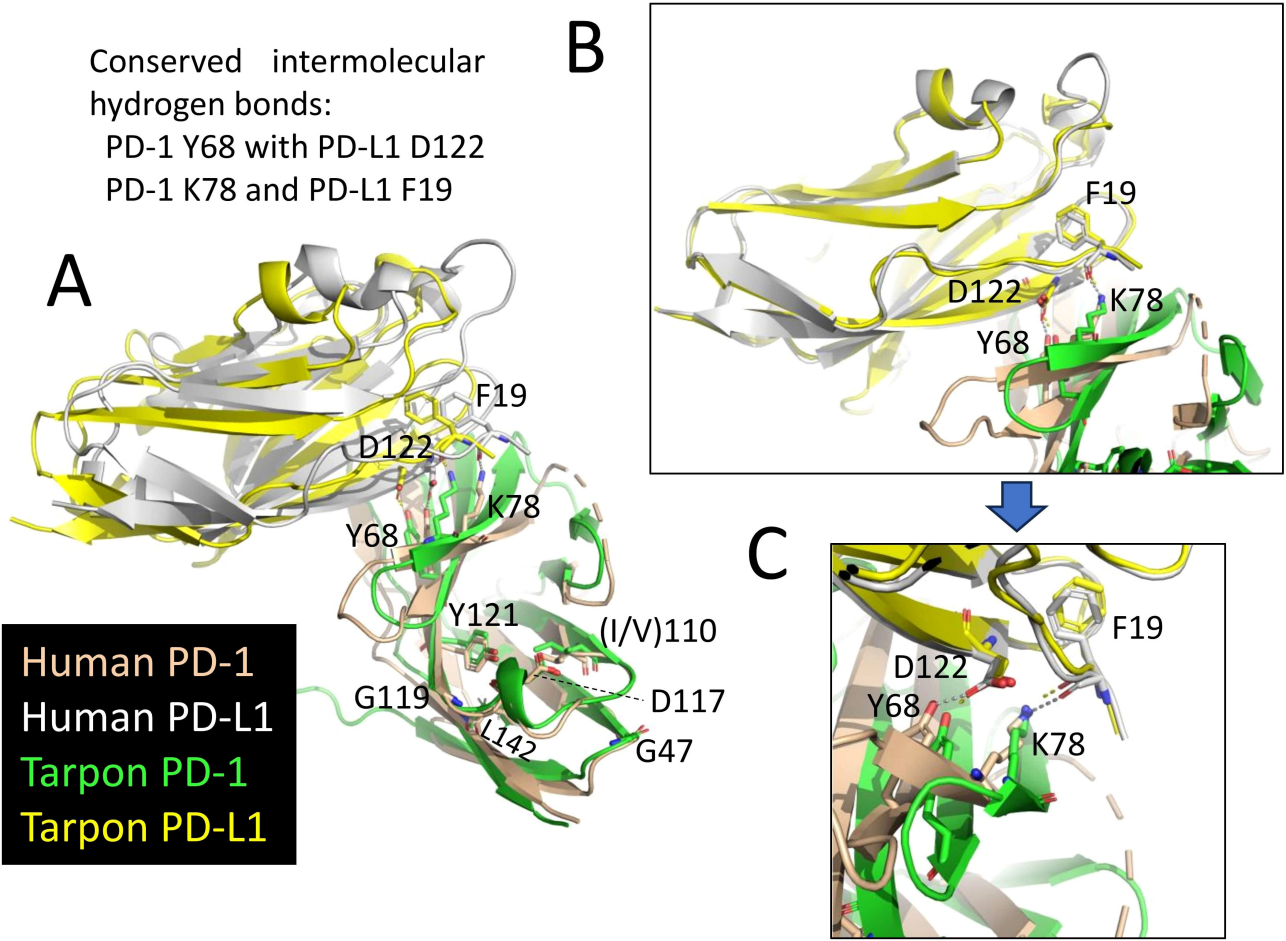
Conservation of PD-1 and PDL-1 interaction between fish and mammals. The figures show superimpositions of the (membrane-distant) IgSF domains of human PD-1 bound to human PD-L1 (PDB accession 4ZQK) and a MultiFOLD predicted interaction structure of the corresponding domains in tarpon PD-1 and PD-L1. Depictions are in cartoon format, except for the highlighted residues that are also in sticks format. The conserved intermolecular hydrogen bonds of PD-1 Y68 with PD-L1 D122 and of PD-1 K78 and PD-L1 F19 are indicated by dashed lines. **(A)** Superimposition guided by the compared complex structures. The figure shows that also in tarpon the PD-1 domain is predicted to have a typical IgSF structure with conserved positions of the IgSF-typical residues G47, (I/V)110, D117, G119, Y121, and L142. **(B)** A superimposition guided by the membrane-distant PD-L1 IgSF domain shows how well conserved this domain is. **(C)** Similar as B, but highlighting the conserved intermolecular hydrogen bonds.

**Figure 2** shows an alignment of deduced PD-1 aa sequences in representative species. The sequences are aligned per coding exon, residue colors reflect their chemical properties, and residue shading highlights interesting conservation patterns. Even though the overall sequence similarities among PD-1 in divergent jawed vertebrates are low (in many cases <20% aa identity), the structural organization has been maintained: a leader sequence, an IgSF domain, a Ser/Thr-rich stalk (“connecting peptide”) region—which in human PD-1 is O-glycosylated (39), a hydrophobic transmembrane domain, and a cytoplasmic tail with ITIM (including human PD-1 Y223; residue numbering as in Ishida et al., 1992 (1)) and ITSM (including human PD-1 Y248) motifs. In Chondrichthyes (represented here by small-spotted catshark and thorny skate) and Sarcoptherygii (e.g., lungfish and human), the ectodomain includes typical IgSF V-category domain residues such as the most characteristic C54, W67, and C123 (yellow shading in **Figure 2**) and somewhat less characteristic G47, S/T57, R/K69, I/L110, D117, G119, Y121, L142, and V144 (non-italic font and gray shading in **Figure 2**) (21, 40–42), and also the more PD-1-specific P39, Y68, K78, the N-glycosylation motif at N116-(S/T)118, T145, and D/E146 (cyan shading in **Figure 2**). For unknown reasons, these sequence motifs have partly deteriorated in the PD-1 IgSF domain in ray-finned fish, including the primitive bichir, reedfish, sturgeon, and gar, and also the “modern ray-finned fish” (teleosts), which are here represented by bonytongue, tarpon, weatherfish, zebrafish, salmon, perch, medaka, and mummichog (**Figure 2**). That these ray-finned fish PD-1 ectodomains nevertheless represent IgSF domains is indicated by the conservation of some of the IgSF-characteristic residues and by structural predictions using computer software. **Figure 3A** shows how for PD-1 of the teleost fish tarpon (*Megalops atlanticus*) computer software predicts an IgSF-typical globular structure with two β-sheets and the conservation of the IgSF-typical residues G47, (I/V)110, D117, G119, Y121, and L142 at similar locations as in human PD-1. This structural prediction of tarpon PD-1 and tarpon PD-L1 membrane-distal IgSF domain also shows that the conserved PD-1 residues Y68 and K78, which in mammals are important for binding the D122 sidechain and F19 main chain of PD-L1 through hydrogen bonds (43, 44), respectively, probably fulfill a similar role in teleosts (**Figure 3**).

### Closer inspection of the ITIM and ITSM motifs

The two conserved tyrosine motifs in the cytoplasmic tail with Y223 and Y248 have been classified as an ITIM and an ITSM, respectively (14). ITIM and ITSM motifs are also found in other molecules and their consensus motifs have been defined as (S/I/V/L)xYxx(I/V/L) (45) and TxYxx(V/I) (46) (x denotes any amino acid), respectively. However, the consensus sequences of both PD-1 sites across jawed vertebrates, are, with exceptions and with some residues not established yet in cartilaginous fish, (I/V)(D/E)YG(E/V)L(D/E)F and (T/V)EYATIx(F/Y), respectively (**Figure 2**). The evolutionary pattern suggests that some other residues in the region that are not part of the ITIM and ITSM definitions are also quite important, and that the threonine defining the ITSM is not beneficial in PD-1 of primitive vertebrates.

### Alignment of PD-L1 and PD-L2 sequences

**Figure 4:**
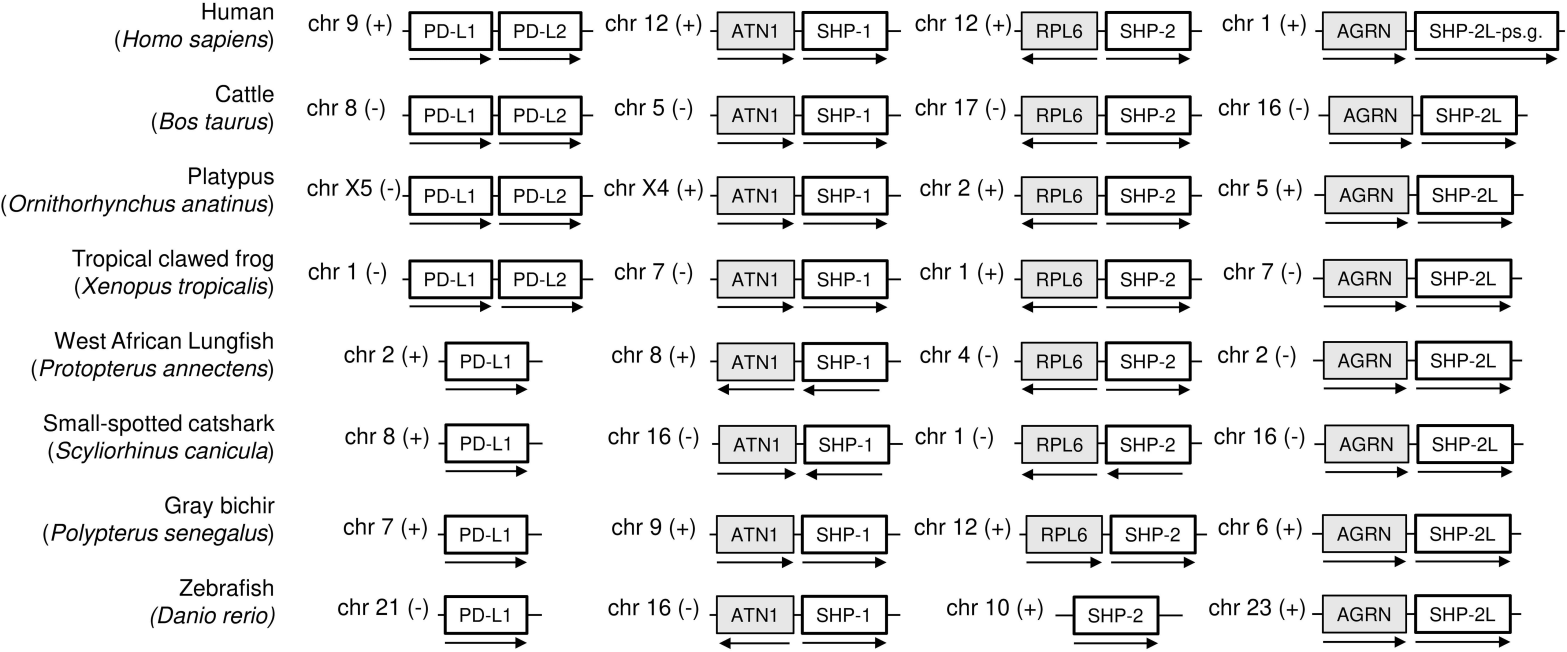
Schematic depiction of the genomic locations of *PD-L1*, *SHP-1*, *SHP-2*, and *SHP-2L* genes. For the same model species as shown in **Figure 1**, this figure shows that (i) a second *PD-L* gene was only found in tetrapods, (ii) *SHP-1*, *SHP-2*, and *SHP-2L* are located on different chromosomes, showing regional conservation between species, and (iii) human *SHP-2L* is only represented by pseudogene fragments. Genes depicted on the same chromosome are not necessarily neighboring genes. *ATN1*, *atrophin 1*; *RPL6*, *ribosomal protein L6*; *AGRN*, *agrin*.

As concluded by Philips et al. (47) and Hu and et al. (23), the *PD-L1/2* gene duplication appears to only have occurred in an ancestor of tetrapod species, and the gene present in fish is called *PD-L1* (**Figure 4**; **Supplementary File 2**). **Supplementary File 2A** shows an alignment of deduced PD-L1 and PD-L2 amino acid sequences in representative jawed vertebrates, including—what to the best of our knowledge has not been reported in article form previously—PD-L1 in primitive ray-finned fish and cartilaginous fish. The alignment figure shows that the above-mentioned PD-L1 residues F19 and D122, which can interact with PD-1, are well conserved. Overall, PD-L1 is better conserved than PD-1 (compare **Figure 2** with **Supplementary File 2A**), which is also reflected in a better match between the (predicted) structures (**Figure 3**).

**Figure 5:**
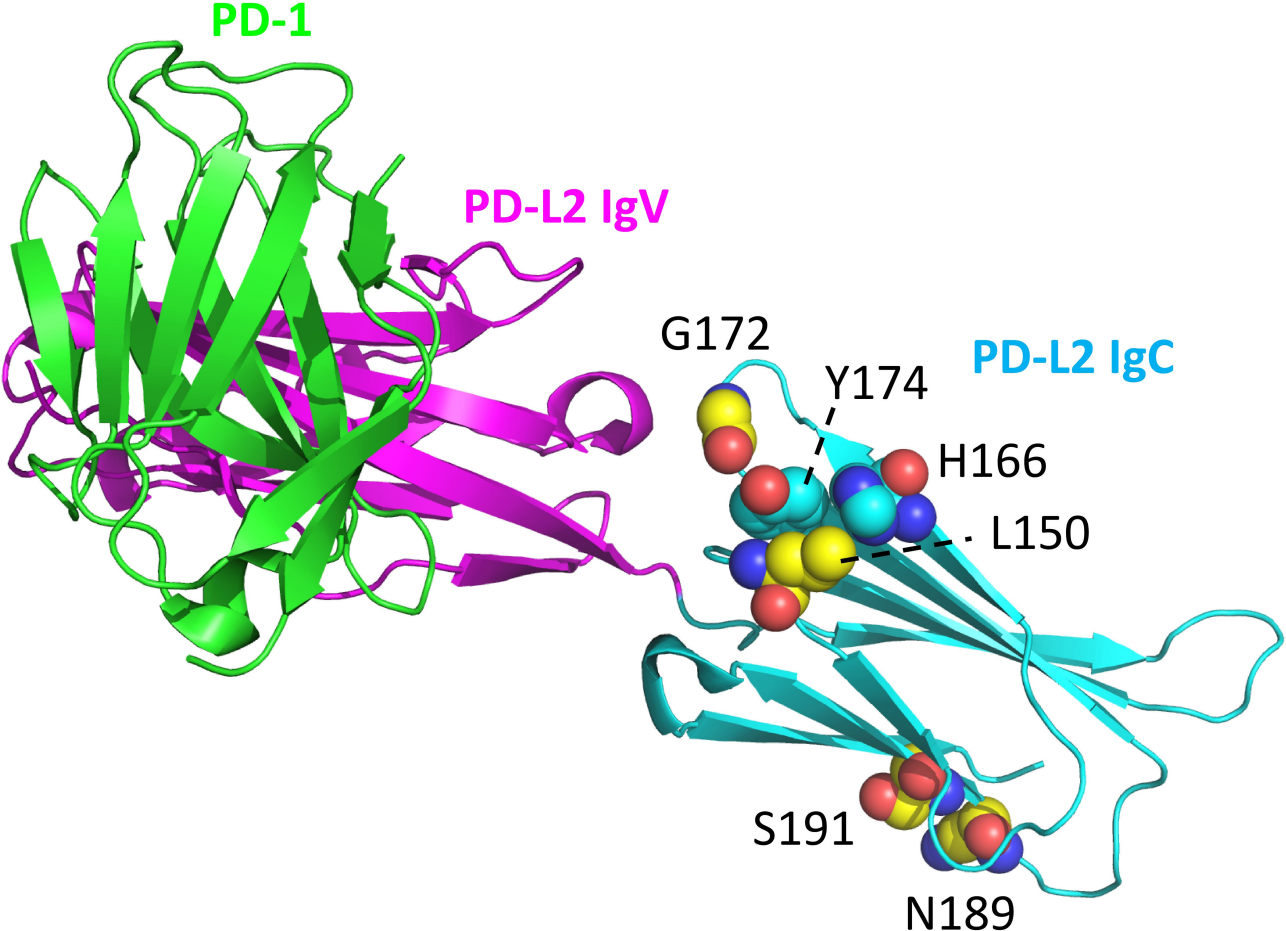
Structural position of PD-L2 residues that distinguish PD-L2 from PD-L1 throughout tetrapods. PD-L2 residues L150, G172, N189, and S191 are highly conserved among, and in tetrapods specific for, PD-L2 (C atoms in yellow). The L150 sidechain forms a complex with aromatic groups of residues at positions 166 and 174 (C atoms in cyan). Highlighted resides are shown in spheres format and element coloring (red for O, blue for N). The depicted structure shows mouse PD-1 and PD-L2 ectodomains (PDB accession 3BP5).

The ectodomains of PD-L1 and PD-L2 consist of two IgSF domains, with the membrane-distal one of the variable (IgV) category and the membrane-proximal one of the constant (IgC) category. Comparing representative PD-L1 and PD-L2 sequences from amphibians to mammals (**Supplementary File 2A**) reveals that the only consistently differing residues between the two are PD-L2 IgC domain motifs: residues L150 and G172 located at a domain shoulder surface close to the IgC-IgV hinge region and an NxS glycosylation motif at position 189 that is expected to be close to the cell membrane (**Figure 5**). The PD-L2 L150 residue forms a complex with aromatic groups that are better conserved in PD-L2 than in PD-L1, involving tyrosine at position 174 and any aromatic residue at position 166 (**Figure 5**).

**Figure 6:**
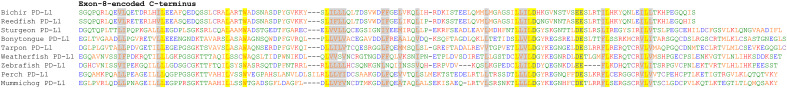
Impressive conservation of long PD-L1 cytoplasmic tail extensions in many ray-finned fish. This figure shows an alignment of the exon-8-encoded C-termini of the nine, out of 13, representative ray-finned fish PD-L1 sequences (see **Supplementary file 2A**) in which such extension could be found. Highly conserved residues are highlighted by yellow (when identical or highly similar) or gray (when similar) shading. Font coloring of residues is as in **Figure 2**.

A peculiar observation—which in principle but without detailed analysis was already reported for teleost fish by Hu et al., 2023 (23)—is that in some ray-finned fish but not in others the cytoplasmic tail has a large extension (**Figure 6**; **Supplementary File 2A**). This variation between ray-finned fish species cannot be explained by splicing variation, as, for example, in salmonid fishes for which abundant genomic sequence and transcript information is available in databases, we could not find the unique extension. The long extension concerns a highly conserved C-terminal sequence encoded by a single exon and includes multiple conserved negatively charged residues and hydrophobic patches, and can already be found in the most primitive ray-finned fish like bichir (**Figure 6**; **Supplementary File 2A**).

### SHP-1 and SHP-2 have well-conserved binding sites for the ITIM and ITSM motifs of PD-1

**Supplementary File 3A** shows a sequence alignment of SHP-1 and SHP-2 sequences in representative species to analyze if distinguishing features in their tandemly arranged Src-homology-2 (SH2) domains (N-SH2 and C-SH2) for binding PD-1 have been well conserved in evolution. This appears to be the case indeed, as indicated by color highlighting in this alignment figure. This suggests that the preference of PD-1 for SHP-2 over SHP-1, and the preferences of the PD-1 ITIM and ITSM motifs to bind the N-SH2 and C-SH2 domains of SHP-2, respectively (15), are also conserved throughout jawed vertebrate species.

### Identification of a second SHP-2 gene, designated SHP-2-like (SHP-2L), conserved from sharks to mammals but lost in primates and rodents

While making the SHP-1 and SHP-2 alignment figure in **Supplementary File 3A**, we realized that there was a second *SHP-2* gene in a conserved gene environment in almost all jawed vertebrates that we investigated, although lost or inactivated in glires (including rabbits and rodents) and higher primates (examples in **Figure 4** and **Supplementary Files 1B**, **3A**). In humans, an apparent pseudogene fragment of this gene is present on Chr. 1 (**Figure 4**; **Supplementary File 1B**). We here designate the molecule as SHP-2-like (SHP-2L). The *SHP-2L* gene had been identified before in zebrafish, but then, probably because of the >60% amino acid identity between SHP-2 and SHP-2L sequences, was mistakenly discussed in the context of teleost fish-specific gene duplications (48). In the expressed sequence tag (EST) database of GenBank, also reports of human transcripts including parts of the *SHP-2L-pseudogene* can be found (e.g., accessions BG945396 and BI601978), seemingly forming long non-coding RNAs (lncRNAs) of >200 nucleotides. Intact *SHP-2L* open reading frames appear to be common among most mammals, including, for example, cattle and platypus (**Figure 4**; **Supplementary File 3**). Intact *SHP-2L* open reading frames were also found in the tree shrew tupaia (*Tupaia chinensis*), which is phylogenetically closer to primates than to glires (49), and in Philippine flying lemur (*Cynocephalus volans*), a primitive primate (**Supplementary File 3**). This suggests that inactivations/losses of *SHP-2L* in the evolution towards humans and mice were acquired independently. In mouse, we have not been able to find remnants of *SHP-2L*.

### Gene expression analysis using single-cell transcriptome datasets

**Figure 7:**
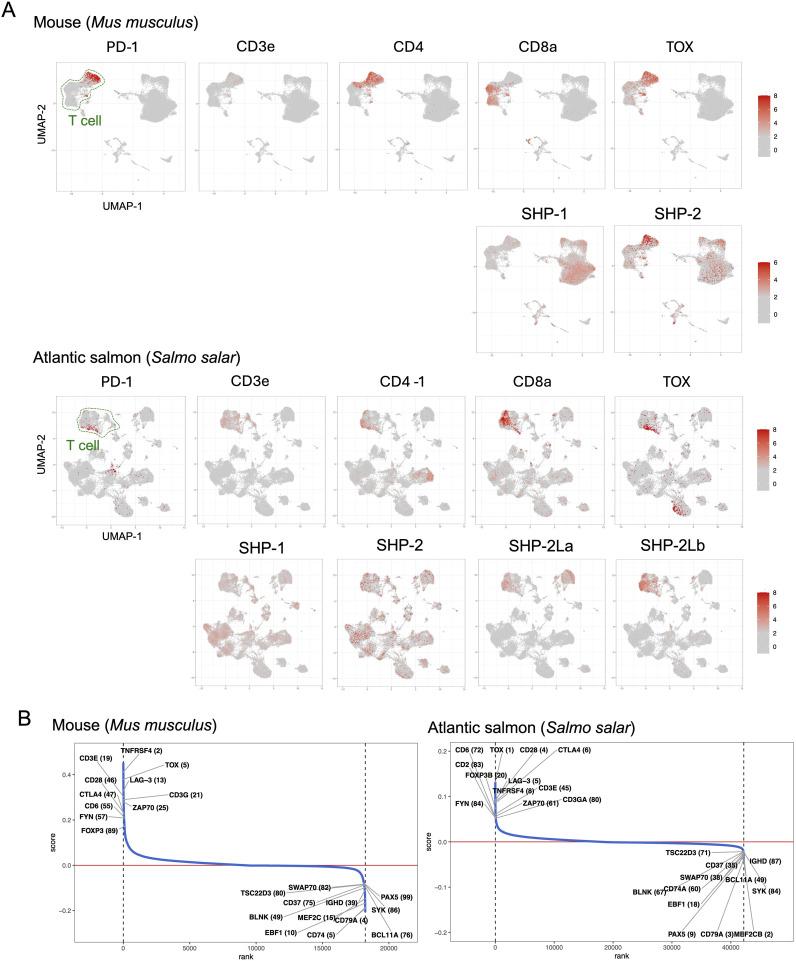
Expression patterns of immunosuppressive genes in salmon and mouse spleens. **(A)** UMAP visualization of gene expression patterns for T cell markers and immunosuppressive genes across spleen cells. The color gradient indicates the normalized expression level. **(B)** Gene ranking based on co-expression correlation with *PD-1*. Genes that show the highest correlation with *PD-1* (top 100) in both mouse and salmon are labeled with their ranking in parentheses. Gene identities are explained in **Supplementary File 5**.

We analyzed *PD-1*, *SHP-2*, and *SHP-2L* expression patterns by using published spleen single-cell transcriptome datasets for the teleost fishes Atlantic salmon (*Salmo salar*) (34) and zebrafish (*Danio rerio*) (35), for nurse shark (*Ginglymostoma cirratum*) (36), and the mammals mouse (37) and cattle (38). This analysis shows that in fish, like in mammals, *PD-1* expression is highest in T cells (**Figure 7A**; **Supplementary File 4**). However, expression patterns of *SHP-2* and *SHP-2L* each show more variation in cellular distribution between samples/species (**Figure 7A**; **Supplementary File 4**), and for now the only solid conclusion is that these two related genes are not consistently co-expressed. Notably, in the sample of Atlantic salmon, a species in which the *SHP-2L* genes are duplicated (50), transcripts of *SHP-2La* and even more so of *SHP-2Lb*, show a much higher specificity for T cells than found for *SHP-1* or *SHP-2* (**Figure 7A**). T-cell specificity of *SHP-2L* can also be clearly seen in the nurse shark sample, but not in the zebrafish sample although even in this sample the association with T cells is stronger for *SHP-2L* than for *SHP-1* or *SHP-2* (**Supplementary File 4**).

In the investigated samples of both mouse and Atlantic salmon, *PD-1* expression showed a strong correlation with multiple genes implicated in T cell immunosuppression (**Figure 7B**). Especially in the Atlantic salmon sample the correlation was strong: the best correlation was found with *TOX* (*thymocyte selection-associated high mobility group box*), known in mammals as a master transcription factor of exhausted T cells (51); the fifth strongest correlation was with *LAG-3* (*lymphocyte activation gene 3*), in mammals associated with T cell inactivation/exhaustion and Tregs (21, 51); the above-discussed *CTLA-4* ranked sixth; and *FOXP3B*, representing a master transcription factor of Tregs (52, 53), ranked 20^th^.

## Discussion

### Identification of PD-1 in fish and conservation of important binding motifs

The present study identified *PD-1* gene in both bony and cartilaginous fish, although previous studies reported its absence (12, 22, 23). While we were preparing our manuscript, another study identified fish *PD-1* as well (24); however, that study was broadly dedicated to the CD28/CTL4 family and did not discuss PD-1 evolution in similar detail as the present study (e.g., for teleost fish PD-1 they only showed one sequence). The failure to find fish *PD-1* in the earlier studies was mostly related to the respective researchers directly comparing mammalian and teleost genetics, which in the case of *PD-1* is complicated because of genomic region rearrangements and partial deterioration of the IgSF domain consensus sequence in the ray-finned fish PD-1 ectodomain. If, however, many species are compared, the conserved gene synteny of *PD-1* throughout jawed vertebrates becomes apparent (**Figure 1**), and even in teleost fish the overall PD-1 molecular structure has been conserved (**Figures 2**, **3**).

In PD-1 throughout jawed vertebrates, the cytoplasmic tail ITIM and ITSM motif regions are impressively conserved, and also the ectodomain residues Y68 and K78 that can bind PD-L1 through hydrogen bonds are well conserved. Furthermore, from sharks to humans, and also in the most primitive ray-finned fish (Cladistia including bichir and reedfish and Acipenseriformes including sturgeon and paddlefish), an N-glycosylation motif (NxS/T) is well conserved at position 116. N-glycosylation at this position was reported to be the primary mediator of binding between PD-1 and galectin-9 (Gal-9), and necessary for the function of PD-1 to prevent Gal-9/TIM-3-induced apoptosis (54). Also another galectin, Gal-7, was found to be a functional PD-1 ligand predominantly through binding N116-bound glycans, thereby inducing PD-1 recruitment of SHP-2 and inhibiting T cell immunity (55). The evolutionary conservation of the N116 glycosylation motif supports the importance of the interactions of PD-1 with galectins, and in the future this should be studied more intensively.

The recent study by Quiniou et al. (24), like we do here, also concluded deterioration of the PD-I IgSF domain consensus sequence in bony fish and reported absence of the threonine in the “ITSM” motif in PD-1 of primitive species. However, those authors did not provide detailed discussions on these topics, and also did not perform *PD-1* transcriptional analysis.

We found *PD-1* transcripts to be most abundant in T cells across diverse clades of jawed vertebrates, including sharks, teleost fish, and mammals (**Figure 7A**; **Supplementary File 4**). In an Atlantic salmon spleen sample, *PD-1* expression showed the strongest correlation with *TOX* expression and was also highly correlated with several other genes involved in T cell immunosuppression (**Figure 7B**). This suggests that—in evolution—the association of PD-1 with immunosuppression was already established in fish, although functional experiments are needed to confirm this. As *TOX* is a master transcription factor of exhausted T cells, this finding also suggests the presence of such cells in fish, which, to our knowledge, has not yet been reported. As in mammals, not all investigated fish samples showed this pronounced association between *PD-1* and *TOX* expression (not shown), likely due to variations in the immune status of the respective samples.

### Detection of a second ancient SHP-2 gene

The present study did not only look at PD-1 itself, but also at the evolution of its interaction partners PD-L1/PD-L2, SHP-1, and SHP-2. Surprisingly, we found an extra copy of an *SHP-2* gene, which we named *SHP-2L*, throughout most jawed vertebrates. Some previous studies had noted this gene but were unaware of its ancientness. For example, Bonetti et al., 2014 (48), seemed to think that the two molecules derived from a teleost fish-specific gene duplication. That research group also studied the functions of zebrafish SHP-2 (their “SHP-2a”) and SHP-2L (their “SHP-2b”) by making knockout and rescue mutants. They concluded that the molecules have overlapping activities that can replace each other, but that in the early development of zebrafish embryos only SHP-2 is critical because it is expressed at higher levels (48). The present study indicates that in teleost fish, like Atlantic salmon and zebrafish, as well as in nurse shark, *SHP-2L* transcripts show a stronger association with T cells than found for *SHP-1* or *SHP-2* transcripts—however, also *SHP-2L* transcripts are expressed in various cell types in these species (**Figure 7A**; **Supplementary File 4**). In cattle, on the other hand, we did not observe that *SHP-2L* shows a stronger association with T cells than found for *SHP-1* or *SHP-2* (**Supplementary File 4**).

The greatest degree of sequence divergence between SHP-2 and SHP-2L is located in their C-termini, in the sequences encoded by exons 13 and 14 and including the residues within or directly surrounding the two YxN phosphorylation motifs (**Supplementary File 3A**). This is also the molecular region of largest difference between SHP-1 and SHP-2 and believed to have a function in regulating activities (56). Future research will have to clarify why most investigated jawed vertebrate species appear to possess both *SHP-2* and *SHP-2L*, whereas *SHP-2L* was independently inactivated or lost in ancestors of glires (e.g., rabbit and mouse) and higher primates. It also remains clarification whether transcripts of the human *SHP-2L-pseudogene* region may have a function at the RNA level. The independent inactivation in ancestors of glires and most primates of an ancient gene was also reported for *interleukin 15-like* (*IL-15L*), which at least in teleost fish participates in type 2 immunity (57, 58). SHP-2 is important for numerous processes and not only for immunity (59), but we speculate that one of the functions of having both SHP-2 and SHP-2L might be to help generate different immune polarizations by initiating different intracellular activation cascades.

### How may the SHP molecules interact with PD-1?

**Figure 8:**
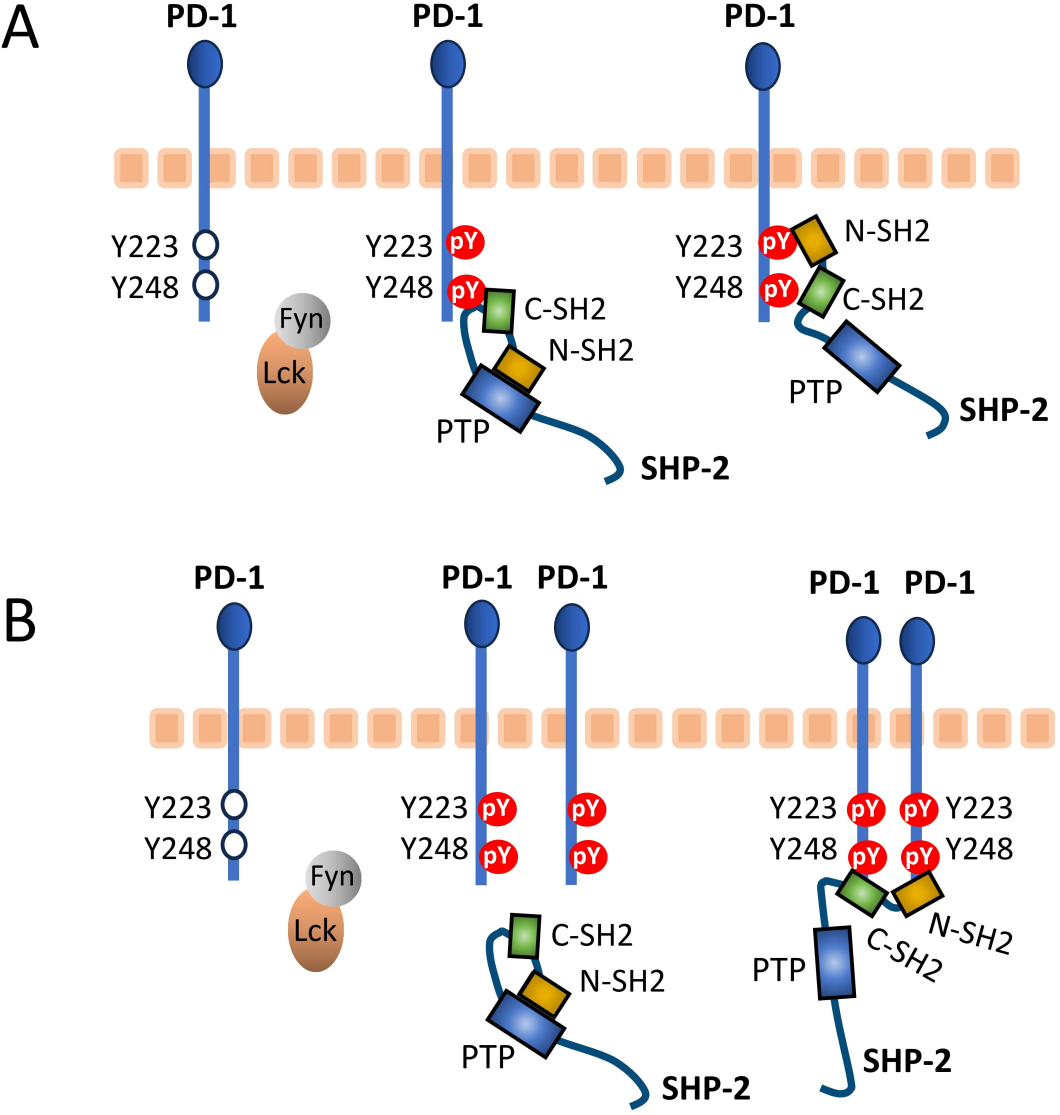
Proposed PD-1/SHP-2 interaction modes. This figure is reused from Patsoukis et al., Interaction of SHP-2 SH2 domains with PD-1 ITSM induces PD-1 dimerization and SHP-2 activation, *Communications Biology*, 2020a (60), under a CC BY 4.0 license. This figure was also used in Patsoukis et al., 2020b (61), together with the following explanation: **(A)** Two-step binding model, according to which SHP-2 C-SH2 binds to PD-1 pY-ITSM with strong affinity, recruiting PD-1 to SHP-2, while PD-1 pY-ITIM binds to N-SH2, displacing it from the PTP site [the Protein Tyrosine Phosphatase domain] to activate the phosphatase. **(B)** Dimerization model, according to which SHP-2 bridges two pY-ITSM residues on two PD-1 molecules via its N-SH2 and C-SH2 domains forming a PD-1:PD-1 dimer and inducing SHP-2 activation.

To discuss this question effectively, **Figure 8** shows the two proposed models for human PD-1/SHP-2 interaction as depicted by Patsoukis et al. (60, 61). Both models recognize that in the resting state the phosphatase domain of SHP-2, which represents its core function, is autoinhibited by binding to its N-SH2 domain, only to be set free after a change in SHP-2 configuration when its two SH2 domains bind phosphorylated tyrosine (pY) sites in the PD-1 ITSM (and ITIM) motifs. For both the “two-step binding model” (**Figure 8A**) (15) and the “dimerization model” (**Figure 8B**) (60) there is experimental support, and they may depend on the cellular conditions (like PD-1 density), and/or the two-step binding mode may precede the dimer binding mode. The below discussion will mostly be based on the two-step model, because several studies show that the PD-1 ITIM motif can contribute to SHP-2 binding (15, 62–64) and we believe that the ITIM region conservation pattern (**Figure 2**) underlines its importance.

**Figure 9:**
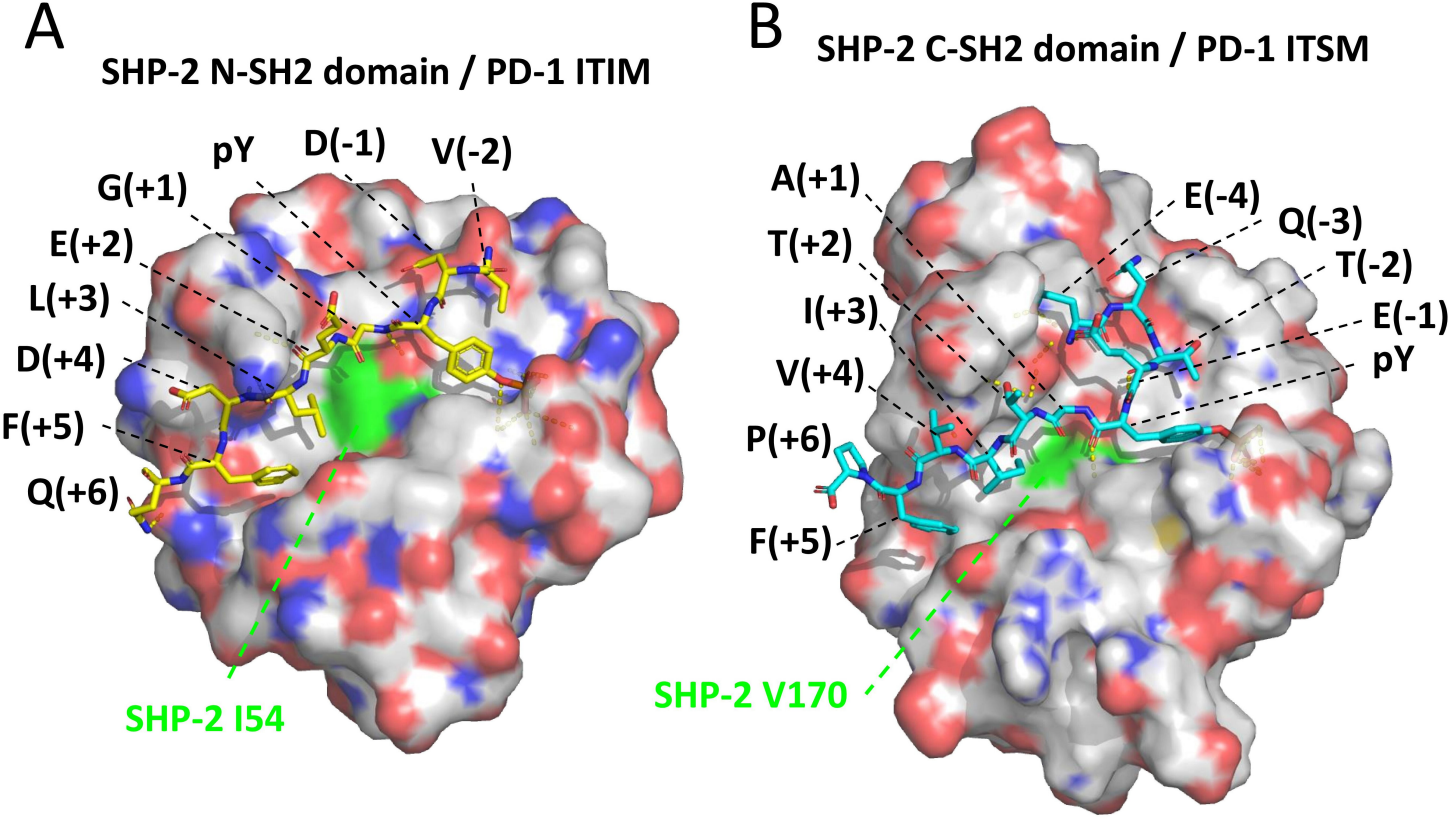
Structures of SHP-2 domains bound to pY peptides derived from the PD-1 cytoplasmic tail motifs. The structures are based on human molecules and were determined by Marasco et al., 2020 (15). **(A)** SHP-2 N-SH2 domain bound to a pY peptide derived from the PD-1 ITIM region; the structure was determined using X-ray crystallography (PDB accession 6ROY). **(B)** SHP-2 C-SH2 domain bound to a pY peptide derived from the PD-1 ITSM region; the structure was determined using NMR spectroscopy (PDB accession 6R5G). The depicted SHP-2 domains are from the WFH residues until the PLNC or PLNT residues (see **Supplementary File 3A**), and are presented in semi-transparent surface style and element coloring (white for C, red for O, blue for N)—except for SHP-2 residues I54 and V170, which are fully in green and contribute to the specificity pockets. Black font descriptions indicate the PD-1 residues and their positions relative to the pY residues. Dashed yellow lines indicate intermolecular polar contacts, and these include interactions between the phosphate groups of the respective pY residues with R32 in N-SH2 or R138 in C-SH2, and one between ITSM-T(pY+2) and C-SH2-E204 (respective SHP-2 residues are not highlighted).

SH2 domains can be found in a wide variety of proteins—over 100 in human alone—and commonly include a conserved arginine (R32 and R138 in domains N-SH2 and C-SH2 of SHP-2; **Supplementary File 3A**) that can pair with the phosphate group of a phosphotyrosine of a target peptide stretch (65, 66). The phosphorylation of the tyrosine determines the (level of) binding and can be used as a switch to initiate signaling cascades by activating the SH2 domain-containing protein. The selectivity of the binding is determined by the SH2 domain’s two pockets, the “pY pocket” and the “specificity pocket.” Those pockets together bind the target peptide, which is in an extended conformation, from around residues -3 to +6 relative to pY (**Figure 9**). It is rather common for SH2 domains to prefer a hydrophobic residue at the +3 position in the phosphotyrosine peptide for inserting into the specificity pocket, a selectivity importantly contributed by having a hydrophobic residue at β-strand D position 4 (65, 66), which in our investigated SHP-1, SHP-2, and SHP-2L sequences across jawed vertebrate species are isoleucine or valine indeed (SHP-2 positions 54 and 170; **Figure 9** and **Supplementary File 3A**). **Supplementary File 3A** shows, by color highlighting, that almost all human SHP-2 residues believed to be important for binding the PD-1 ITIM and ITSM motifs (15, 67) are near-perfectly conserved in both SHP-2 and SHP-2L, while exhibiting several pronounced differences from SHP-1. Between SHP-2 and SHP-2L, the only replacement among the most important ITIM and ITSM binding residues is the conservative exchange of the specificity pocket L210 residue in the C-SH2 domain of SHP-2 for a valine in SHP-2L, suggesting that they may have slightly different preferences for the hydrophobic residue at the ITSM pY+3 position. Overall, however, the conservation pattern suggests that SHP-2 and SHP-2L bind to similar regulatory motifs, including also the PD-1 ITIM and ITSM motifs, and that their mode of binding is very well conserved from before the duplication in evolution of the *SHP-2*/*SHP-2L* ancestral gene.

### Conservation of the PD-1 cytoplasmic tail ITIM and ITSM region sequences

As explained above, binding of SHP-2 to the PD-1 cytoplasmic tail involves two SH2 domains, each having a different function. The SHP-2 C-SH2 domain provides binding energy and specificity as it has a very high affinity for the ITSM motif of PD-1, being >10 times higher than the other possible interactions between the individual PD-1 ITIM or ITSM regions and SHP-2 N-SH2 or C-SH2 domains (15). The ITIM motif of PD-1 has a higher affinity for the N-SH2 domain of SHP-2 than for its C-SH2 domain, but the N-SH2 domain itself has a higher affinity for the PD-1 ITSM motif—although for this binding it will be outcompeted by the C-SH2 domain (15). The fine-tuning of these affinities and interaction modes can be expected to be quite selective for the sequences involved, and reflected in evolutionary conservation. Indeed, for the ITIM and ITSM motifs of PD-1 across bony vertebrates, we found the consensus sequences (I/V)(D/E)YG(E/V)L(D/E)F and (T/V)EYATIx(F/Y), respectively. It is complicated to understand the implications of the differences in consensus sequence directly from the resolved binding structures for the individual PD-1 ITIM and ITSM regions and SHP-2 N-SH2 and C-SH2 domains (15) (**Figure 9**). For example, Marasco et al., 2020 (15), concluded that hydrogen bonds between ITSM-T(pY+2) and C-SH2–E204 (**Figure 9**) help explain why the C-SH2 domain has a higher affinity for the ITSM than for the ITIM motif, but it should be realized that also the N-SH2 domain has a glutamic acid at a matching position (N-SH2-E90; **Supplementary File 3A**).

Some of the conserved residues can at least partially be explained by pY-peptide consensus sequence preferences determined for the two human SHP-2 SH2 domains by *in vitro* binding experiments. For the SHP-2 N-SH2 domain, separate studies using randomized mixes of degenerate peptides found these to be (I/L/V/m)(m/n/r)pY(T/V/A)(e/q/t/v)(I/V/L/f) for positions -2 to +3 (lower case letters represent less frequently selected residues) (Sweeney et al., 2005 (68)), and hydrophobic aromatic residues at the pY +4 and +5 positions (Imhof et al., 2006 (69)). Meanwhile, for the SHP-2 C-SH2 domain, the consensus of preferred pY-peptide sequences was (T/V/I/y)XpY(A/s/t/v)(i/v/t/q)X(I/v/l) for positions -2 to +3 (68) and hydrophobic aromatic residues at the pY +4 and +5 positions (69). This largely agreed with studies by De Souza et al., 2002 (70), on preferences for residues on pY positions from +1 to +5 by the two SH2 domains, although those authors found pronounced opposite preferences for L(pY+3) versus I(pY+3) by N-SH2 and C-SH2, respectively, agreeing with the respective conservations of those residues in the ITIM and ITSM motifs among PD-1 sequences (**Figure 2**). Those authors also found a strong preference for A(pY+1) by C-SH2 but not by N-SH2 (70), suggesting that this residue in the ITSM motif (**Figure 2**) enhances C-SH2 binding. On the other hand, the G(pY+1) residue conserved in PD-1 ITIM of bony vertebrates was not found to contribute to binding either of the two SH2 domains. Sweeney et al., 2005 (68), reported that only the C-SH2 domain and not the N-SH2 domain of PD-1 includes threonine among the residues preferred at the pY -2 position, which can help explain why in higher vertebrates C-SH2 outcompetes N-SH2 domain for binding the ITSM motif. Imhof et al., 2006 (69), showed that phenylalanine at pY+5 contributed to binding of both the N-SH2 and C-SH2 domains of SHP-2. Residue F(pY+5) was also found in a pY-peptide motif of the CagA protein of a virulent strain of infectious bacterium *Helicobacter pylori*, and shown to enhance CagA binding to SHP-2 in injected host cells (71).

Interestingly, a recent study by Masubuchi et al., 2025 (64), showed that, compared to the mammalian consensus situation as found in humans, PD-1 in rodents is attenuated by having a reduced affinity for SHP-2 through deviations of the ITIM region sequences and of the region between the ITIM and ITSM motifs. Our analysis shows that the typical mammalian PD-1 ITIM region residues not found in mouse, being (D/E)(pY-1) and G(pY+1), are also common in non-mammalian species (**Figure 2**). Their combined exchange in human PD-1 for the mouse ITIM sequence reduced the affinity of human PD-1 for SHP-2 (64).

In short, for the PD-1 ITIM and ITSM evolutionary consensus sequences in bony vertebrates, which we determined as (I/V)(D/E)YG(E/V)L(D/E)F and (T/V)EYATIx(F/Y), the following can probably be concluded (1): The (I/V)(D/E)YG(E/V)LxF and (T/V)xYATIx(F/Y) motifs contribute to binding the N-SH2 and C-SH2 domain of SHP-2, respectively (2); T(pY-2) found in PD-1 ITSM of higher vertebrates and some fish increases selectivity for SHP-2 C-SH2 (3); Some evolutionary consensus residues within the PD-1 ITIM and ITSM motifs may not be selected to enhance binding to SHP-2 SH2 domains, including ITIM residue (D/E)(pY+4) and ITSM residue E(pY-1).

Replacement of PD-1 ITIM G(pY+1) for an alanine promoted SHP-1 recruitment (63), emphasizing how these motifs are fine-tuned for positive as well as negative selectivity towards different SH2 domains. Notably, cartilaginous fish PD-1 molecules possess an alanine at the ITIM pY+1 position, and also lack some of the other residues that are common in the ITIM or ITSM regions of PD-1 in Osteichthyes (bony animals) (**Figure 2**). This suggests that in Chondrichthyes compared to Osteichthyes, the binding of PD-1 to SHP-2, and/or SHP-1 and SHP-2L, may be somewhat different.

Some of the conserved residues in the PD-1 ITIM and ITSM motifs may also be involved in selective interactions with kinases, about which in the case of PD-1 much less is known (13, 72).

### Are ITIM and ITSM good descriptions for the PD-1 cytoplasmic tail motif?

Evolutionary, the system of SH2 domains recognizing phosphotyrosine peptides is developed for specificity (65). SHP-1 and SHP-2 have been described as the only mammalian cytosolic tyrosine phosphatases with tandemly arranged SH2 domains (SHP-2L would be a third), and this tandem arrangement further increases selectivity (65, 73). It can be argued that the PD-1 ITIM and ITSM motifs should be considered as a combined motif, because both contribute to efficient interaction with SHP-2 (15, 62–64, 74), and it is debatable whether naming the PD-1 motifs an ITIM and ITSM is very helpful for understanding their function. Furthermore, in PD-1 of primitive jawed vertebrates, the T(pY-2) residue that is part of the ITSM sequence definition is not common and even in tetrapods may have only been established in an ancestor of Amniotes (reptiles, birds, and mammals). The ITSM pY-2 positions do appear important though, as we only found conservative replacements of threonine for a serine or valine (which has a similar size), in either case creating a motif agreeing with the ITIM consensus definition.

The name immunoreceptor tyrosine-based inhibitory motif (ITIM) was coined for SH2 domain binding motifs of the (S/I/V/L)xYxx(I/V/L) type in the cytoplasmic tails of receptors that, generally, counteract immune activating receptors, and, in many cases, ITIMs function as an individual motif (45, 75). In the case of PD-1 function, however, several experiments fail to find a role for its ITIM motif (14, 60), and it may better be understood as a motif that complements the ITSM motif, the latter having a more prominent role in PD-1 function (14, 60, 62, 74).

The name immunoreceptor tyrosine-based switch motif (ITSM) was originally based on a CD150 motif TIYxx(V/I) that preferentially binds SHP-2 when phosphorylated but, if the tyrosine is not phosphorylated, can still efficiently bind the adaptor protein SH2 domain protein 1A (SH2D1A), creating a “switch” in molecular interactions (46). However, this high affinity of SH2D1A for the CD150 motif regardless of phosphorylation depends on having a hydrophobic residue at the motif pY-1 position (76), whereas at that position the PD-1 “ITSM” sequences consistently have a glutamic acid (**Figure 2**). Therefore, PD-1 ITSM having a similar “switch” function is unlikely, and we are not aware of any evidence in that direction.

Also in the cytoplasmic tail of the inhibitory receptor BTLA, an ITIM and an ITSM motif are found in tandem (77), and at least under some conditions they work synergistically to bind SHP-2 (78, 79).

For convenience, and lack of better options, it probably is best to currently keep the ITIM and ITSM designations for the respective PD-1 motifs. However, readers should be aware that these designations refer to functions that these PD-1 motifs probably do not have, and that it may be better to understand them as a combined motif (as in the model in **Figure 8A**).

### Motifs consistently distinguishing between tetrapod PD-L1 and PD-L2 reside in the PD-L2 IgC domain

Philips et al. (47) and Hu et al. (23) already reported that the *PD-L1/2* gene duplication occurred in a direct ancestor of tetrapod species. This was confirmed in the present study by gene synteny analysis (**Figure 4**), phylogenetic tree analysis—although with low bootstrap values—(**Supplementary File 2B**), and conservation of unique motifs in the PD-L2 IgC domain that are absent in tetrapod PD-L1 (**Figure 5**; **Supplementary File 2A**). We refer to Philips et al. (47) for characteristic residues that differ between PD-L1 and PD-L2 IgV domains in placental mammals, and agree with those authors that a similar divide is not found between these IgV domains in ectotherm tetrapods. However, to the best of our knowledge, the present article is the first to observe PD-L2 IgC motifs that are conserved throughout PD-L2 evolution and not found in tetrapod PD-L1. These include a motif with residues L150 and G172 located at a domain shoulder close to the IgC-IgV hinge region (**Figure 5**), which is probably inherited from the shared PD-L1/2 ancestor because “PD-L1” (the name given to the common ancestor) in sharks and rays also shares such motif (**Supplementary File 2A**). Additionally, and this seems to have been newly acquired in PD-L evolution, the PD-L2 sequences have an NxS glycosylation motif at position 189 (**Figure 5**). We can only speculate about the function of these conserved motifs, but point out that the N189 site was confirmed as an actual glycosylation site and was found—although in combination with other PD-L2 N-glycosylation sites, therefore prohibiting conclusions on the individual motif contribution—to promote PD-L2 function and stability (80).

### PD-L1 cytoplasmic tails may have a function

In the cytoplasmic tails of PD-L1 of many ray-finned fish, we found a long and highly conserved stretch (**Figure 6**; **Supplementary File 2A**), which had been identified already in several teleost fish (23). Some studies have implicated functionality for the human PD-L1 cytoplasmic tail. For example, Ghosh et al., 2021, found that phospholipase C-γ1 (PLC-γ1) binds to the N-terminal part of the human PD-L1 cytoplasmic tail, thereby enhancing PLC-γ1 activation by epidermal growth factor receptor (EGFR). However, this region of the PD-L1 cytoplasmic tail is not well-conserved outside mammals. Furthermore, Wen et al., 2021 (81), reported that a regulated association of the human PD-L1 cytoplasmic tail with the cell membrane regulates PD-L1 degradation. Given the several hydrophobic patches in these ray-finned fish-specific PD-L1 tail segments (**Figure 6**), they may also play a role in interactions with the cell membrane. Although the function of these long ray-finned fish-specific PD-L1 tail segments remains speculative, they do support the general notion that PD-L1 function can be regulated in part through its cytoplasmic tail.

## Conclusion

By identifying *PD-1* genes in cartilaginous as well as bony jawed fish, the present study confirms that throughout jawed vertebrates the immune systems are quite similar (18–21). Comparison of PD-1 in different species shows that especially the “ITIM” and “ITSM” motif regions are well conserved, which suggests conservation of selectivity for binding particular SH2 domains such as those of SHP-2. The names “ITIM” and “ITSM” have their origin in different molecular contexts, and in the case of PD-1 can probably be considered misleading as they fail to recognize the collaboration between the motifs and the high level of evolutionary conservation of several other residues in these stretches that are not part of the ITIM and ITSM consensus definitions. After future studies will have clarified the functions of the various residues, maybe a better name for these PD-1 motifs can be considered. The high level of evolutionary conservation of the N-glycosylation motif at PD-1 residue 116 supports the recently described functional importance of PD-1 interacting with galectins. The PD-L2 IgC domain has motifs that distinguish it from PD-L1 throughout tetrapod species, and it would be interesting to investigate their function. The long cytoplasmic tail of PD-L1 in many ray-finned fish supports the model that PD-L1 is not only an inert surface marker but that its cytoplasmic tail may be used to modify its function. Unexpectedly, we found that an *SHP-2L* gene has been conserved from the level of sharks, and that its absence as a functional gene in mouse and human is due to independent inactivation events.

We hope that this evolutionary analysis will enhance the understanding of PD-1 function and ultimately contribute to further advancements in immune checkpoint therapy.

## Data Availability

The original contributions presented in the study are included in the article/**Supplementary Material**. Further inquiries can be directed to the corresponding author/s.

## References

[1] Ishida Y Agata Y Shibahara K Honjo T. Induced expression of PD-1, a novel member of the immunoglobulin gene superfamily, upon programmed cell death. EMBO J. (1992) 11:3887–95. doi: 10.1002/j.1460-2075 PMC556898 1396582

[2] Agata Y Kawasaki A Nishimura H Ishida Y Tsubata T Yagita H. Expression of the PD-1 antigen on the surface of stimulated mouse T and B lymphocytes. Int Immunol. (1996) 8:765–72. doi: 10.1093/intimm/8.5.765 8671665

[3] Latchman Y Wood CR Chernova T Chaudhary D Borde M Chernova I. PD-L2 is a second ligand for PD-1 and inhibits T cell activation. Nat Immunol. (2001) 2:261–8. doi: 10.1038/85330 11224527

[4] Cai L Li Y Tan J Xu L Li Y. Targeting LAG-3, TIM-3, and TIGIT for cancer immunotherapy. J Hematol Oncol. (2023) 16:101. doi: 10.1186/s13045-023-01499-1 37670328 PMC10478462

[5] Twomey JD Zhang B. Cancer immunotherapy update: FDA-approved checkpoint inhibitors and companion diagnostics. AAPS J. (2021) 23:39. doi: 10.1208/s12248-021-00574-0 33677681 PMC7937597

[6] Thibult ML Mamessier E Gertner-Dardenne J Pastor S Just-Landi S Xerri L. PD-1 is a novel regulator of human B-cell activation. Int Immunol. (2013) 25:129–37. doi: 10.1093/intimm/dxs098 23087177

[7] Strauss L Mahmoud MAA Weaver JD Tijaro-Ovalle NM Christofides A Wang Q. Targeted deletion of PD-1 in myeloid cells induces antitumor immunity. Sci Immunol. (2020) 5:eaay1863. doi: 10.1126/sciimmunol.aay1863 31901074 PMC7183328

[8] Dong H Zhu G Tamada K Chen L. B7-H1, a third member of the B7 family, co-stimulates T-cell proliferation and interleukin-10 secretion. Nat Med. (1999) 5:1365–9. doi: 10.1038/70932 10581077

[9] Freeman GJ Long AJ Iwai Y Bourque K Chernova T Nishimura H. Engagement of the PD-1 immunoinhibitory receptor by a novel B7 family member leads to negative regulation of lymphocyte activation. J Exp Med. (2000) 192:1027–34. doi: 10.1084/jem.192.7.1027 PMC2193311 11015443

[10] Nishimura H Nose M Hiai H Minato N Honjo T. Development of lupus-like autoimmune diseases by disruption of the PD-1 gene encoding an ITIM motif-carrying immunoreceptor. Immunity. (1999) 11:141–51. doi: 10.1016/s1074-7613(00)80089-8 10485649

[11] Nishimura H Okazaki T Tanaka Y Nakatani K Hara M Matsumori A. Autoimmune dilated cardiomyopathy in PD-1 receptor-deficient mice. Science. (2001) 291:319–22. doi: 10.1126/science.291.5502.319 11209085

[12] Bernard D Hansen JD Du Pasquier L Lefranc MP Benmansour A Boudinot P. Costimulatory receptors in jawed vertebrates: conserved CD28, odd CTLA4 and multiple BTLAs. Dev Comp Immunol. (2007) 31:255–71. doi: 10.1016/j.dci.2006.06.003 16928399

[13] Hui E Cheung J Zhu J Su X Taylor MJ Wallweber HA. T cell costimulatory receptor CD28 is a primary target for PD-1-mediated inhibition. Science. (2017) 355:1428–33. doi: 10.1126/science.aaf1292 PMC6286077 28280247

[14] Chemnitz JM Parry RV Nichols KE June CH Riley JL. SHP-1 and SHP-2 associate with immunoreceptor tyrosine-based switch motif of programmed death 1 upon primary human T cell stimulation, but only receptor ligation prevents T cell activation. J Immunol. (2004) 173:945–54. doi: 10.4049/jimmunol.173.2.945 15240681

[15] Marasco M Berteotti A Weyershaeuser J Thorausch N Sikorska J Krausze J. Molecular mechanism of SHP2 activation by PD-1 stimulation. Sci Adv. (2020) 6:eaay4458. doi: 10.1126/sciadv.aay4458 32064351 PMC6994217

[16] Buchbinder EI Desai A. CTLA-4 and PD-1 pathways: similarities, differences, and implications of their inhibition. Am J Clin Oncol. (2016) 39:98–106. doi: 10.1097/COC.0000000000000239 26558876 PMC4892769

[17] Chamoto K Hatae R Honjo T. Current issues and perspectives in PD-1 blockade cancer immunotherapy. Int J Clin Oncol. (2020) 25:790–800. doi: 10.1007/s10147-019-01588-7 31900651 PMC7192862

[18] Venkatesh B Lee AP Ravi V Maurya AK Lian MM Swann JB. Elephant shark genome provides unique insights into gnathostome evolution. Nature. (2014) 505:174–9. doi: 10.1038/nature12826 PMC3964593 24402279

[19] Dijkstra JM. TH2 and Treg candidate genes in elephant shark. Nature. (2014) 511:E7–9. doi: 10.1038/nature13446 25008534

[20] Flajnik MF. Re-evaluation of the immunological big bang. Curr Biol. (2014) 24:R1060–5. doi: 10.1016/j.cub.2014.09.070 PMC4354883 25517375

[21] Takizawa F Hashimoto K Miyazawa R Ohta Y Veríssimo A Flajnik MF. CD4 and LAG-3 from sharks to humans: related molecules with motifs for opposing functions. Front Immunol. (2023) 14:1267743. doi: 10.3389/fimmu.2023.1267743 38187381 PMC10768021

[22] Hansen JD Du Pasquier L Lefranc MP Lopez V Benmansour A Boudinot P. Origin and evolution of the adaptive immune system: genetic events and selective pressures. The B7 family of immunoregulatory receptors: a comparative and evolutionary perspective. Mol Immunol. (2009) 46:457–72. doi: 10.1016/j.molimm.2008.10.007 19081138

[23] Hu CB Huang C Wang J Hong Y Fan DD Chen Y. PD-L1/BTLA checkpoint axis exploited for bacterial immune escape by restraining CD8+ T cell-initiated adaptive immunity in zebrafish. J Immunol. (2023) 211:816–35. doi: 10.4049/jimmunol.2300217 37486225

[24] Quiniou SMA Clark T Bengtén E Rast JP Ohta Y Flajnik M. Extraordinary diversity of the CD28/CTLA4 family across jawed vertebrates. Front Immunol. (2024) 15:1501934. doi: 10.3389/fimmu.2024.1501934 39606244 PMC11599192

[25] Rigato E Minelli A. The great chain of being is still here. Evol Educ Outreach. (2013) 6:18. doi: 10.1186/1936-6434-6-18

[26] Sayers EW Bolton EE Brister JR Canese K Chan J Comeau DC. Database resources of the national center for biotechnology information. Nucleic Acids Res. (2022) 50:D20–6. doi: 10.1093/nar/gkab1112 PMC8728269 34850941

[27] Solovyev V Kosarev P Seledsov I Vorobyev D. Automatic annotation of eukaryotic genes, pseudogenes and promoters. Genome Biol. (2006) 7:S10. doi: 10.1186/gb-2006-7-s1-s10 PMC1810547 16925832

[28] Dijkstra JM. A method for making alignments of related protein sequences that share very little similarity; shark interleukin 2 as an example. Immunogenetics. (2021) 73:35–51. doi: 10.1007/s00251-020-01191-5 33512550

[29] Almagro Armenteros JJ Tsirigos KD Sønderby CK Petersen TN Winther O Brunak S. SignalP 5.0 improves signal peptide predictions using deep neural networks. Nat Biotechnol. (2019) 37:420–3. doi: 10.1038/s41587-019-0036-z 30778233

[30] Adiyaman R Edmunds NS Genc AG Alharbi SMA McGuffin LJ. Improvement of protein tertiary and quaternary structure predictions using the ReFOLD refinement method and the AlphaFold2 recycling process. Bioinform Adv. (2023) 3:vbad078. doi: 10.1093/bioadv/vbad078 37359722 PMC10290552

[31] Zheng GX Terry JM Belgrader P Ryvkin P Bent ZW Wilson R. Massively parallel digital transcriptional profiling of single cells. Nat Commun. (2017) 8:14049. doi: 10.1038/ncomms14049 28091601 PMC5241818

[32] Pettinello R Redmond AK Secombes CJ Macqueen DJ Dooley H. Evolutionary history of the T cell receptor complex as revealed by small-spotted catshark (Scyliorhinus canicula). Dev Comp Immunol. (2017) 74:125–35. doi: 10.1016/j.dci.2017.04.015 28433528

[33] Stuart T Butler A Hoffman P Hafemeister C Papalexi E Mauck WM 3rd. Comprehensive integration of single-cell data. Cell. (2019) 177:1888–902.e21. doi: 10.1016/j.cell.2019.05.031 31178118 PMC6687398

[34] Sun J Ruiz Daniels R Balic A Andresen AMS Bjørgen H Dobie R. Cell atlas of the Atlantic salmon spleen reveals immune cell heterogeneity and cell-specific responses to bacterial infection. Fish Shellfish Immunol. (2024) 145:109358. doi: 10.1016/j.fsi.2024.109358 38176627

[35] Jiao A Zhang C Wang X Sun L Liu H Su Y. Single-cell sequencing reveals the evolution of immune molecules across multiple vertebrate species. J Adv Res. (2024) 55:73–87. doi: 10.1016/j.jare.2023.02.017 36871615 PMC10770119

[36] Matz H Taylor RS Redmond AK Hill TM Ruiz Daniels R Beltran M. Organized B cell sites in cartilaginous fishes reveal the evolutionary foundation of germinal centers. Cell Rep. (2023) 42:112664. doi: 10.1016/j.celrep.2023.112664 37342909 PMC10529500

[37] Kimmel JC Penland L Rubinstein ND Hendrickson DG Kelley DR Rosenthal AZ. Murine single-cell RNA-seq reveals cell-identity- and tissue-specific trajectories of aging. Genome Res. (2019) 29:2088–103. doi: 10.1101/gr.253880.119 PMC6886498 31754020

[38] Gao Y Li J Cai G Wang Y Yang W Li Y. Single-cell transcriptomic and chromatin accessibility analyses of dairy cattle peripheral blood mononuclear cells and their responses to lipopolysaccharide. BMC Genomics. (2022) 23:338. doi: 10.1186/s12864-022-08562-0 35501711 PMC9063233

[39] Tit-Oon P Wonglangka A Boonkanta K Ruchirawat M Fuangthong M Sasisekharan R. Intact mass analysis reveals the novel O-linked glycosylation on the stalk region of PD-1 protein. Sci Rep. (2023) 13:9631. doi: 10.1038/s41598-023-36203-3 37316505 PMC10267102

[40] Halaby DM Poupon A Mornon J. The immunoglobulin fold family: sequence analysis and 3D structure comparisons. Protein Eng. (1999) 12:563–71. doi: 10.1093/protein/12.7.563 10436082

[41] Cannon JP Haire RN Litman GW. Identification of diversified genes that contain immunoglobulin-like variable regions in a protochordate. Nat Immunol. (2002) 3:1200–7. doi: 10.1038/ni849 12415263

[42] SMART SM00406. Immunoglobulin V-Type sequence characteristics description by the SMART (Simple Modular Architecture Research Tool) web resource of the European Molecular Biology Laboratory . Available online at: http://smart.embl.de/smart/do_annotation.pl?DOMAIN=SM00406 (Accessed 1 March, 2024).

[43] Zak KM Kitel R Przetocka S Golik P Guzik K Musielak B. Structure of the complex of human programmed death 1, PD-1, and its ligand PD-L1. Structure. (2015) 23:2341–8. doi: 10.1016/j.str.2015.09.010 PMC4752817 26602187

[44] Du J Qin Y Wu Y Zhao W Zhai W Qi Y. The design of high affinity human PD-1 mutants by using molecular dynamics simulations (MD). Cell Commun Signal. (2018) 16:25. doi: 10.1186/s12964-018-0239-9 29879980 PMC5992718

[45] Ravetch JV Lanier LL. Immune inhibitory receptors. Science. (2000) 290:84–9. doi: 10.1126/science.290.5489.84 11021804

[46] Shlapatska LM Mikhalap SV Berdova AG Zelensky OM Yun TJ Nichols KE. CD150 association with either the SH2-containing inositol phosphatase or the SH2-containing protein tyrosine phosphatase is regulated by the adaptor protein SH2D1A. J Immunol. (2001) 166:5480–7. doi: 10.4049/jimmunol.166.9.5480 11313386

[47] Philips EA Garcia-España A Tocheva AS Ahearn IM Adam KR Pan R. The structural features that distinguish PD-L2 from PD-L1 emerged in placental mammals. J Biol Chem. (2020) 295:4372–80. doi: 10.1074/jbc.AC119.011747 PMC7135984 31882544

[48] Bonetti M Rodriguez-Martinez V Paardekooper Overman J Overvoorde J van Eekelen M Jopling C. Distinct and overlapping functions of ptpn11 genes in Zebrafish development. PloS One. (2014) 9:e94884. doi: 10.1371/journal.pone.0094884 24736444 PMC3988099

[49] Kundu S Pakrashi A Kamalakannan M Singha D Tyagi K Banerjee D. Complete mitogenome of the endangered and endemic Nicobar treeshrew (Tupaia nicobarica) and comparison with other Scandentians. Sci Rep. (2022) 12:877. doi: 10.1038/s41598-022-04907-7 35042947 PMC8766473

[50] Lien S Koop BF Sandve SR Miller JR Kent MP Nome T. The Atlantic salmon genome provides insights into rediploidization. Nature. (2016) 533:200–5. doi: 10.1038/nature17164 PMC8127823 27088604

[51] Alfei F Kanev K Hofmann M Wu M Ghoneim HE Roelli P. TOX reinforces the phenotype and longevity of exhausted T cells in chronic viral infection. Nature. (2019) 571:265–9. doi: 10.1038/s41586-019-1326-9 31207605

[52] Sakaguchi S Yamaguchi T Nomura T Ono M. Regulatory T cells and immune tolerance. Cell. (2008) 133:775–87. doi: 10.1016/j.cell.2008.05.009 18510923

[53] Yamaguchi T Takizawa F Fischer U Dijkstra JM. Along the axis between type 1 and type 2 immunity; principles conserved in evolution from fish to mammals. Biol (Basel). (2015) 4:814–59. doi: 10.3390/biology4040814 PMC4690019 26593954

[54] Yang R Sun L Li CF Wang YH Yao J Li H. Galectin-9 interacts with PD-1 and TIM-3 to regulate T cell death and is a target for cancer immunotherapy. Nat Commun. (2021) 12:832. doi: 10.1038/s41467-021-21099-2 33547304 PMC7864927

[55] Wu G Deng W Chen HY Cho HJ Kim J. Galectin 7 leads to a relative reduction in CD4+ T cells, mediated by PD-1. Sci Rep. (2024) 14:6625. doi: 10.1038/s41598-024-57162-3 38503797 PMC10951237

[56] Poole AW. Jones ML. A SHPing tale: Perspect Regul SHP-1 SHP-2 tyrosine phosphatases by C-terminal tail. Cell Signal. (2005) 17:1323–32. doi: 10.1016/j.cellsig.2005.05.016 16084691

[57] Dijkstra JM Takizawa F Fischer U Friedrich M Soto-Lampe V Lefèvre C. Identification of a gene for an ancient cytokine, interleukin 15-like, in mammals; interleukins 2 and 15 co-evolved with this third family member, all sharing binding motifs for IL-15Rα. Immunogenetics. (2014) 66:93–103. doi: 10.1007/s00251-013-0747-0 24276591 PMC3894449

[58] Yamaguchi T Chang CJ Karger A Keller M Pfaff F Wangkahart E. Ancient cytokine interleukin 15-like (IL-15L) induces a type 2 immune response. Front Immunol. (2020) 11:549319. doi: 10.3389/fimmu.2020.549319 33193315 PMC7658486

[59] Niogret C Birchmeier W Guarda G. SHP-2 in lymphocytes’ Cytokine and inhibitory receptor signaling. Front Immunol. (2019) 10:2468. doi: 10.3389/fimmu.2019.02468 31708921 PMC6823243

[60] Patsoukis N Duke-Cohan JS Chaudhri A Aksoylar HI Wang Q Council A. Interaction of SHP-2 SH2 domains with PD-1 ITSM induces PD-1 dimerization and SHP-2 activation. Commun Biol. (2020) 3:128. doi: 10.1038/s42003-020-0845-0 32184441 PMC7078208

[61] Patsoukis N Wang Q Strauss L Boussiotis VA. Revisiting the PD-1 pathway. Sci Adv. (2020) 6:eabd2712. doi: 10.1126/sciadv.abd2712 32948597 PMC7500922

[62] Yokosuka T Takamatsu M Kobayashi-Imanishi W Hashimoto-Tane A Azuma M Saito T. Programmed cell death 1 forms negative costimulatory microclusters that directly inhibit T cell receptor signaling by recruiting phosphatase SHP2. J Exp Med. (2012) 209:1201–17. doi: 10.1084/jem.2011274 PMC3371732 22641383

[63] Xu X Masubuchi T Cai Q Zhao Y Hui E. Molecular features underlying differential SHP1/SHP2 binding of immune checkpoint receptors. Elife. (2021) 10:e74276. doi: 10.7554/eLife.74276 34734802 PMC8631942

[64] Masubuchi T Chen L Marcel N Wen GA Caron C Zhang J. Functional differences between rodent and human PD-1 linked to evolutionary divergence. Sci Immunol. (2025) 10:eads6295. doi: 10.1126/sciimmunol.ads6295 39752535 PMC11774210

[65] Marasco M Carlomagno T. Specificity and regulation of phosphotyrosine signaling through SH2 domains. J Struct Biol X. (2020) 4:100026. doi: 10.1016/j.yjsbx.2020.100026 32647828 PMC7337045

[66] Diop A Santorelli D Malagrinò F Nardella C Pennacchietti V Pagano L. SH2 domains: folding, binding and therapeutical approaches. Int J Mol Sci. (2022) 23:15944. doi: 10.3390/ijms232415944 36555586 PMC9783222

[67] Wang Q Zhao WC Fu XQ Zheng QC. Exploring the allosteric mechanism of src homology-2 domain-containing protein tyrosine phosphatase 2 (SHP2) by molecular dynamics simulations. Front Chem. (2020) 8:597495. doi: 10.3389/fchem.2020.597495 33330386 PMC7719740

[68] Sweeney MC Wavreille AS Park J Butchar JP Tridandapani S Pei D. Decoding protein-protein interactions through combinatorial chemistry: sequence specificity of SHP-1, SHP-2, and SHIP SH2 domains. Biochemistry. (2005) 44:14932–47. doi: 10.1021/bi051408h 16274240

[69] Imhof D Wavreille AS May A Zacharias M Tridandapani S Pei D. Sequence specificity of SHP-1 and SHP-2 Src homology 2 domains. Critical roles of residues beyond the pY+3 position. J Biol Chem. (2006) 281:20271–82. doi: 10.1074/jbc.M601047200 16702225

[70] De Souza D Fabri LJ Nash A Hilton DJ Nicola NA Baca M. SH2 domains from suppressor of cytokine signaling-3 and protein tyrosine phosphatase SHP-2 have similar binding specificities. Biochemistry. (2002) 41:9229–36. doi: 10.1021/bi0259507 12119038

[71] Higashi H Tsutsumi R Fujita A Yamazaki S Asaka M Azuma T. Biological activity of the Helicobacter pylori virulence factor CagA is determined by variation in the tyrosine phosphorylation sites. Proc Natl Acad Sci U S A. (2002) 99:14428–33. doi: 10.1073/pnas.222375399 PMC137900 12391297

[72] Bardhan K Aksoylar HI Le Bourgeois T Strauss L Weaver JD Delcuze B. Phosphorylation of PD-1-Y248 is a marker of PD-1-mediated inhibitory function in human T cells. Sci Rep. (2019) 9:17252. doi: 10.1038/s41598-019-53463-0 31754127 PMC6872651

[73] Lorenz U. SHP-1 and SHP-2 in T cells: two phosphatases functioning at many levels. Immunol Rev. (2009) 228:342–59. doi: 10.1111/j.1600-065X.2008.00760.x PMC2669678 19290938

[74] Okazaki T Maeda A Nishimura H Kurosaki T Honjo T. PD-1 immunoreceptor inhibits B cell receptor-mediated signaling by recruiting src homology 2-domain-containing tyrosine phosphatase 2 to phosphotyrosine. Proc Natl Acad Sci U S A. (2001) 98:13866–71. doi: 10.1073/pnas.231486598 PMC61133 11698646

[75] Daëron M Latour S Malbec O Espinosa E Pina P Pasmans S. regulates negatively BCR-, TCR-, and FcR-dependent cell activation. Immunity. (1995) 3:635–46. doi: 10.1016/1074-7613(95)90134-5 7584153

[76] Poy F Yaffe MB Sayos J Saxena K Morra M Sumegi J. Crystal structures of the XLP protein SAP reveal a class of SH2 domains with extended, phosphotyrosine-independent sequence recognition. Mol Cell. (1999) 4:555–61. doi: 10.1016/s1097-2765(00)80206-3 10549287

[77] Watanabe N Gavrieli M Sedy JR Yang J Fallarino F Loftin SK. BTLA is a lymphocyte inhibitory receptor with similarities to CTLA-4 and PD-1. Nat Immunol. (2003) 4:670–9. doi: 10.1038/ni944 12796776

[78] Gavrieli M Watanabe N Loftin SK Murphy TL Murphy KM. Characterization of phosphotyrosine binding motifs in the cytoplasmic domain of B and T lymphocyte attenuator required for association with protein tyrosine phosphatases SHP-1 and SHP-2. Biochem Biophys Res Commun. (2003) 312:1236–43. doi: 10.1016/j.bbrc.2003.11.070 14652006

[79] Chemnitz JM Lanfranco AR Braunstein I Riley JL. B and T lymphocyte attenuator-mediated signal transduction provides a potent inhibitory signal to primary human CD4 T cells that can be initiated by multiple phosphotyrosine motifs. J Immunol. (2006) 176:6603–14. doi: 10.4049/jimmunol.176.11.6603 16709818

[80] Xu Y Gao Z Hu R Wang Y Wang Y Su Z. PD-L2 glycosylation promotes immune evasion and predicts anti-EGFR efficacy. J Immunother Cancer. (2021) 9:e002699. doi: 10.1136/jitc-2021-002699 34697216 PMC8547513

[81] Wen M Cao Y Wu B Xiao T Cao R Wang Q. PD-L1 degradation is regulated by electrostatic membrane association of its cytoplasmic domain. Nat Commun. (2021) 12:5106. doi: 10.1038/s41467-021-25416-7 34429434 PMC8384847

[82] Hopp TP Woods KR. Prediction of protein antigenic determinants from amino acid sequences. Proc Natl Acad Sci U S A. (1981) 78:3824–8. doi: 10.1073/pnas.78.6.3824 PMC319665 6167991

