# Supplementary material for: PD-1 is conserved from sharks to humans: new insights into PD-1, PD-L1, PD-L2, and SHP-2 evolution
Source: Front Immunol. 2025 May 28;16:1573492. doi: 10.3389/fimmu.2025.1573492 (PMC12151841; doi:10.3389/fimmu.2025.1573492)
Supplement: Supplementary file 1 [file DataSheet1.pdf]

Supplementary file 1

Deduced PD-1, PD-L1, SHP-1, and SHP-2 amino acid sequences in representative species

(A) Phylogenetic tree showing the time in millions of years ago (MYA) when the major species clades discussed in the present study separated. Information is based on Benton and Donoghue, 2007, doi: 10.1093/molbev/msl150, and Broughton et al., 2013, doi: 10.1371/currents.tol.2ca8041495ffafd0c92756e75247483e. This figure is only intended as a helpful reminder.

(B) Representative sequences of programmed cell death protein 1 (PD-1), PD Ligand 1 (PD-L1), Src homology region 2 domain-containing phosphatase-1 (SHP-1), -2 (SHP-2), and -2-like (SHP-2L) are shown together with their GenBank accession number. For some sequences extra information is provided.

The sequences are shown on the following pages:

PD-1 sequences

Page 3 Tetrapods

Page 5 Lungfish

Page 5 Sharks and Rays

Page 6 Bichirs and Reedfish

Page 7 Sturgeons and Paddlefishes

Page 7 Gars

Page 7 Teleost fishes

PD-L1 and PD-L2 sequences

Page 9 Tetrapods

Page 15 Lungfish

Page 15	Sharks and Rays
Page 16	Bichirs and Reedfish
Page 17	Sturgeons and Paddlefishes
Page 17	Gars
Page 18	Teleost fishes

SHP-1 sequences

Page 21	Tetrapods
Page 24	Lungfish
Page 24	Sharks and Rays
Page 26	Bichirs and Reedfish
Page 27	Sturgeons and Paddlefishes
Page 27	Gars
Page 28	Teleost fishes

SHP-2 and SHP-2L sequences

Page 31	Tetrapods
Page 38	Lungfish
Page 38	Sharks and Rays
Page 40	Bichirs and Reedfish
Page 41	Sturgeons and Paddlefishes
Page 43	Gars
Page 43	Teleost fishes

(A) Phylogenetic time tree of species clades

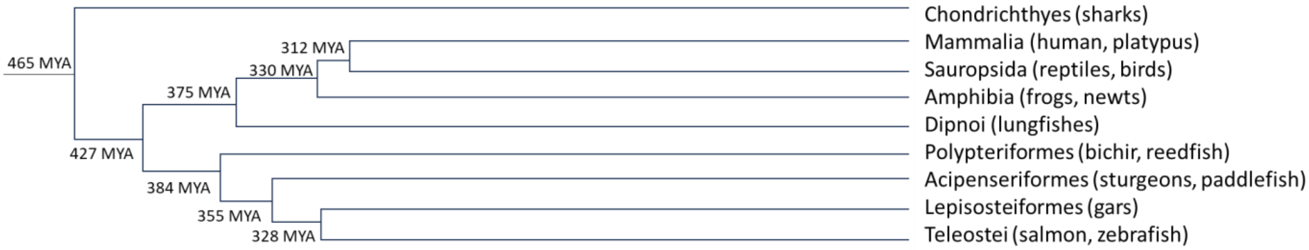

(B) Sequences

<u>Programmed cell death protein 1 (PD-1)</u>

Tetrapods (Class: Sarcopterygii [lobe-finned fishes plus tetrapods]; Infraclass: Tetrapoda)

>Human_(*Homo_sapiens*)_PD-1

Source: GenBank NP_005009

MQIPQAPWPVVWAVLQLGWRPGWFLDSPDRPWNPPTFSPALLVVTEGDNATFTCSFSNTSESFVLNWYRMS
PSNQTDKLAAFPEDRSQPGQDCRFRVTQLPNGRDFHMSVVRARRNDSGTYLCGAISLAPKAQIKESLRAELRVT
ERRAEVPTAHPSPSPRPAGQFQTLVVGVVGGLLGSLVLLVWVLAVICSRAARGTIGARRTGQPLKEDPSAVPVFSV
DYGELDFQWREKTPEPPVPCVPEQTEYATIVFPSGMGTSSPARRGSADGPRSAQPLRPEDGHCSWPL

>Mouse_(*Mus_musculus*)_PD-1

Source: GenBank NP_032824

MWVRQVPWSFTWAVLQLSWQSGWLLEVPNGPWRSLTFYPAWLTVSEGANATFTCSLSNWSEDLMLNWNRL
SPSNQTEKQAAFCNGLSQPVQDARFQIIQLPNRHDFHMNILDTRRNDSGIYLCGAISLHPKAKIEESPGAELVVTE
RILETSTRYPSPSPKPEGRFQGMVIGIMSALVGIPVLLLLAWALAVFCSTSMSEARGAGSKDDTLKEEPSAAPVPSV
AYEELDFQGREKTPELPTACVHTEYATIVFTEGLGASAMGRRGSADGLQGPRPPRHEDGHCSWPL

>Cattle_(*Bos_taurus*)_PD-1

Source: GenBank NP_001076975

MGTPRALWPLVWAVLQLGCWPGWLLEASSRPWSALTFSPPRLVVPEGANATFTCSFSSKPERFVLNWYRKSPS
NQMDKLAAFPEDRSQPSRDRRFRVTPLPDGQQFNMSIVAAQRNDSGVYFCGAIYLPPRTQINESHSAELMVTE
AVLEPPTEPPSPQPRPEGQMQSLVIGVTSVLLGVLLLPPLIWVLAAVFLRATRGGCARRSQDQPPKEGCPSVPAVT
VDYGELDFQWREKTPEPAAPCVPEQTEYATIVFPGRRASADSPQGPWPLRTEDGHCSWPL

>Platypus_(*Ornithorhynchus_anatinus*)_PD-1

Source: GenBank XP_007667778

MELLHVTVAAFYTVLLGCQPRLLRAQIKSEASCSASSPAKLFGQKGENFTFFCSVSNMPNSTTLNWYKYFNNHKD
KFAELKKEDDKYHEGRHHVSKCSNSVFKITILELQLNDTGEYFYEAIELSTSAKIWRSNCTLLNVTARTPGPSTAPPKI
ISIKKGNVQGTVILSSSIVAVALLLLLGWVLYAIAQKRKGINKSENDDLTLKGEPTSRPVFTVDYGELEFQRGEKPPKT
VGTCTSEQTEYATIIFLPEKPLAHNGLQIKK

>Chicken_(*Gallus_gallus*)_PD-1

Source: GenBank XP_040534565

MALGTSRTMWDSTEAALVVLCVLLLCCNPPLAGCHQVTLFPATLTRPAGSSATFICNISMENSSLEFNLNWYQKT
NNSNPQKIAGIIRNIPQKKMEKYRLFNNTPVFKMEILNLHQNDSGFYYCGLITFSRSDKVVESSHSQLVVTEAPEKT
NTIDEPSEEESSPPDHIKAVLLGTLLLAGVIVLLLFGYIIINNRRADVQKPSSGNTLAEVKPPVVPVPTVDYGVLEFQR
DPHSQVPLETCPAEQTEYATIVFPEEKPITPERGKRHKDERTWQLPSQPC

>Swan_goose_(*Anser_cygnoides_domesticus*)_PD-1

Source: GenBank XP_013050415

MAPGASRTTRGNVDVALAGLCVLLLCCSPVLAGCHRVTFSPATLTRPAGGSATFFCNISIENNSSLEYNLNWYKET
NHSDPQKIAQIRRSIPQTKTEKYLLSNHTPVFKIEILNLHQNDSGSYYCGLIAFFQPNKVEESNRSHLVVTAAPEKIN
TTEEPGVEDSSPPSHIKAVILGVLLLGCVVVLVVLGYCIVTYRRGDVQKPPSENAAEKEEKPPVVSVSTVDYGVLEF
QWDPHTQLPPETRPDDQTEYATIIFPEEKPVTPERGKRHLDERTWQPPSQPC

>Green_anole_lizard_(*Anolis_carolinensis*)_PD-1

Source: GenBank XP_008120853

MAMESRHRIMHWVGAWLLLLCCRSALLLKPSATFWPTQLNQSTGTTAEFTCNISNAEVFEDSVNWYKFDANKQ
PGKLDTRNKNKYEITRLDTQTFRMKILNLERNDSGVYSCILVATHSSLGFTESNHGNLTVTEIAPTPGSPEDEYESDH

DAKENENAGHNIPLAAIGALGLVLVSVLASLCFFLIKVIRRRQERGKPHDENAPLEEEPPAVAVFTVDYGVLEFQAR
KNARRPPFPPPSPKPQPLDQTEYATIIFPTEKSVPMENTGKLGPMENTKRTKRQVRWVPTGRQQLH

>Green_sea_turtle_(Chelonia_mydas)_PD-1

Source: GenBank XP_007057904 (modified compared to this GenBank prediction)

MLVLVVGICAVLLTCRPVLLLSQLETVTFSPEKLSLPVGDTASFFCNITTANFPQFDYSLNWYKKINSTYTQKIAEFNG
NANKFPKEKFTLINHTSSVEIRILHLTENDSGKYYCGLIAFSSPSKVVESNVSQLIVTEGGPTTIPNVTEDNQVGDFK
VPVIIGVSIAGAMLLGPITYVLFITTRRTGGQQKPHRENALLKKEQVTTYTVDYGVLEFQQEEHTEAPVESYPPDNT
EYAVIVFPEEKPVTPERGKKTKHQRTCQI

>Tropical_clawed_frog_(*Xenopus_tropicalis*)_PD-1

Source: GenBank XP_031758711 (modified compared to this GenBank prediction in order to match with amphibian TSA transcript information)

MTHSPILCASLMSVERVFLIIIGICVITILGHARPEGVILFEHLPFEHHLTPGKTAVFICNISALNFNPTDINWSKTHNN
NTSKIADIKSPKDTNRIHIETNWPSRIAELHIRNVTVNDSGEYHCEYLNVTANSKIMLSNRSRLNVTGDNEYAKTFT
ESTTQSPMNKGTIKLAVSISTSIFLILLLLSTSLLLWHKKRNKTPQTHLKHLEKPPQDPEVITVDYGVLAFPNNCPYRK
SVELCTLDQVEYATIMFPQGTPSLGERSGKDAACNRSPRVCRD

Lungfishes (Class: Sarcopterygii [lobe-finned fishes plus tetrapods]; Subclass: Dipnoi)

>West_African_Lungfish_(*Protopterus_annectens*)_PD-1

Source: GenBank XP_043926841

MKTVWFVGQCTLTLFLLTTHAEAVSENEVTMSLYPNNSNVYTGGSVTFRCTCTKSTQTMNWYKQENNTFKKFA
DTMHSTDERFKVFLHDNKTATLQITNLQKNDSNKYYCGIISLDSNRKTKESNHLILNVEEEPVTNVTTTVTSTVNTQ
TNGNSKYIILGSALPVIIMFLILVSILLFVMQRKQEITKQQDDKKCLEMEHVAPVYVTEYGELEFPCQKQADKPVEIP
PADNLDVEYATIVFIPELPNSKEIATSYHSMPNKFCGNASPRLK

Sharks and Rays (Class: Chondrichthyes [cartilaginous fish]; Subclass: Elasmobranchii)

>Small-spotted_catshark_(*Scyliorhinus_canicula*)_PD-1

Source: GenBank XP_038672536

MTMILFAQQIILVFSVTLWQNAVGDSSTLLQDPMTIKKRSGETAVISCKLSRGHFTEDMTLIWYNYINQS
QSKIGEIDFKKNSSRCVEQVILTWDLDRATMEMAHLMKNNSGAYGCQLLALYGSVIKKANATNIIVSDS
EIPIPENRNNTDNVTEKSQNNEAKIQIIVAAVIAAFIIICLLIYILFRFRPKKQGTDASPHAADASCQKSEDPI
STVCSMDYAVLKIPGKNHQHNLASSVASDDSCYATIVFAPQQQATGVQKTITFN

>Thorny_skate_(*Amblyraja_radiata*)_PD-1

Source: GenBank XP_032887962

MKEMATILFTHQIFLVLSLSLWQNAQGESLTLLQYPSTIMKNYGESATIYCVLSKVNFTDHMCLVWYKYTSGNKIK
VGEIRLKTSNISANDQIQLLWNLDTNTAIMSIQQLKKNDSGTYGCELLSVADFVNIEKANTTKITVNFTEGQQTASE
QINSTKNVTELSKKGKIEIIVAAVIAAFVIICILIFILIRYRPKKQDPNPSPPTADAECQKPNDRISTVFSIDYAVLQVPGK
NIRQNLTTSVASDDSYYATIIFAPE

Bichirs and Reedfish (Class: Actinopterygii [ray-finned fish]; Order: Polypteriformes)

>Gray_bichir_(*Polypterus_senegalus*)_PD-1

Source: GenBank JAAWVN010020317 (deduced from genomic DNA)

MPLGLSALIWALVLCYSAAFGTEALENGHLGVKCRQKILQLVGTMGQKMTVLFTFEEECKTDCTNMLYKDGTKIA
EKDQNPHDPRLSYEWQKNITMTIDVLQLNDTAIYYSVIYSPGSSKILRESNCVNLTVVTEDISQENQTDTHVGTLSP
ENQDTKTMLFIIFMIVIVILLLVLGFTLFLIWDRNKGISSPHILSSKQQENNVHHSTPVYAIEYGELDFQKCPATEAKQ
EDHVEYATIRFPDEAGAQGHQDSKVQNHI

>Reedfish_(*Erpetoichthys_calabaricus*)_PD-1

Source: GenBank NC_041395 (deduced from genomic_DNA)

MPLGLSVPIWALVLCYSAAFGTEALENGHLGAKCRQKILQLVGTMGQKMTVLFTFEEECKSDCTNVLYKNGTKIA
EKDQNPRDPRLSYEWQKNITMTIDALQLNDTAIYYGIIYSPGSSKILRESNCVNLTVVTEDISQENQTDTHVGTPSP
EKQDTKTMLFIIFMVVIAILLLVLGFTLFLIWDRNKGISSPHILSSKQQESNVHHSTPVYAIEYGELDFQKCPVTEANQ
EDHVEYATIRFPDEAVAQGHQDSNVQNQLQQH

Sturgeons and Paddlefishes (Class: Actinopterygii [ray-finned fishes]; Order: Acipenseriformes)

>Sterlet_sturgeon_(*Acipenser_ruthenus*)_PD-1

Source: GenBank XP_033883562

MGKVTRVISAVTSLLLLLIQQGDSQPSELNLSSKEGQILTLNFTFPKKLSGTESMNLNLKKNQKKIAEINFMGGQG
NKYNGKRLTFIWQGETQTATLSISGLIKNDSGVYQCNYVLNSKIEVSNSINLTVRTEESSYTNQTDLSDPASTADSHP
QDHSIPQNPVIAASVTVMALLLLVLSITVFLIWDRNKGTSSPQPTTIKRQQESKQSQAVPVYSIEYGVLEFPRRDTL
PPATMPEHNDHVEYATITFPAGQEGGSGGRHCWSHGR

> Paddlefish_(*Polyodon_spathula*)_PD-1

Source: GenBank XP_041120684

MGKVTRVISALTPLLFLLIQQGDSQPSKLNFSSKEEGQTLTLNFTFLKKLSVTESMNLNLKRNEQKIAEINFNKGQG
NKFNEKRLTFTWQGEAQTATLSISGLFKNDSGLYQCNYVLNTMVEFSNSINLTVRTRESSYINQTDPTSTADSHPQ
DHSIPKNPLIAASVTVTAVLLLVLSYTVFLLWDRNKGTSSPQLDRIKCQQESKPTQAMPVYSIEYGVLEFPRRGALPP
ATMPDHDAHVEYATITFLAGQEGELGGGTAGVTAGSRERARERPLCQSDVTAADLTGKDQNPTIPTLTPNPQHH
THTTPHPPLTPTPPLHNTTPPTQRFGAKS

Gars (Class: Actinopterygii [ray-finned fishes]; Order: Lepisosteiformes)

>Spotted_gar_(*Lepisosteus_oculatus*)_PD-1

Source: GenBank XP_015216858 (but N-terminus modified based on GenBank GFIM01007684)

MSHQASWLSMLSLLLLLPNANLQEEKMEAFRGTEGEHFNMRFVFNSSIAPSSIKGAGLYKDGKKINEYIKGGQSI
GHHRILIKLEGNNVTVRFNSLQLSDSGVYHIGLSRSITTDPLTLSSKVNLTVSERGNGTEAPPTPTQEDIPNQISYIIIA
SGATVLILLVVVGYCLYLATHKNTGPPAALTNPTPRTKIQDSEMSRGLPVYSIEYGVLEFPGAERRVEGEGQPVVQD
NVEYATITFPPCQNPRAGRGAQSQLCCGRVR

Teleost fish (Class: Actinopterygii [ray-finned fishes]; Infraclass: Teleostei)

>Asian_bonytongue_ (*Scleropages_formosus*)_PD-1

Source: GenBank XP_018586611
MASKGRAAACQRVAGHGVSQPSSLLLLVLIFLYHEPLLAKEVKGVAGENVTILFSFNGSFDGTLLKYANLYKDGNKS
AEWRAASSPAPSSHLSLNVTSGKVALVIHGVTEGDSGTYYVALFHDRSNADVTESFKVLLTVHLAATDLTPVNCTE
TSAGSSQSITYVYVICGLGAAVLSVALVSWFYLTNERSSANQPTQRSAENSQVTSKDPSHLPGYSIEYGVLEFQSRP
SGQDGDWSRGSRLDLRDNVEYSVITFQQRNHQGAAKNPTGVC

>Tarpon_(*Megalops_atlanticus*)_PD-1

Source: GenBank KAG7488134

The underlined sequence was used for structural modeling.

MSNLQSMGVMRHPTKQLFSLPLLLVLLLCEEHIQAEEITGTTGKKIKIRFTFKNLSHIHRKTVNLYKGSQKIAECND
MKTVCVHGRYVLEEGFQDIILNITNLTSSDAGTYYVAMIPQTLTPIESGRVSLILSSGEPFTVTPPTSLNTGNVYNKT
SPSGSSVYIYIIAAFGVITVVLPTVLLAWFYLTRVKHSDDQLHQATSTKFQVASEVSNSMPIYSIEYVMLDFHNRPK
GREKDWRSDVGLKPQDNVEYASITFPPGHRQTDVQQAQRQFCWETVQYAHKKRP

>Oriental_weatherfish_(*Misgurnus_anguillicaudatus*)_PD-1

Source: GenBank XP_055074768

MKCDHKKFILLLSFAVYLILKVESKHEKEITVSVITGDNVTANFTFKHSGINSPSSVLTLYKNDNKIESCKQTTKNCFVR
FVISNVDENNTATLYIINITSEDDGKYYIGLHDDVKPMIISNEIFTIKPRDKTNESSTTSVPKTTVNDSPKPEETSVQTF
IYISASVLMFICIFVGLLCWFHRTCSRKPATDNPQAQSNGTTQGDLGRSGGVSVSCVEYGELDFQNRPTRDDRVK
HAEVTSKDQDSVEYAAIIFPQQKQIPGGRMRDNQRAHPVTR

>Zebrafish_(*Danio_rerio*)_PD-1

Source: GenBank GDQH01026610

MKRDLKKLLLFSFMLILVHNVDSLNGTLGKNITVRFTLQGCDINSGHSKLILYKNGHKKETCKQTEPFCSTNFVFGD
AENSTVTLHITNLTMEHGGEYHVAMRTTNCNAEKPLIESNKVSIRVTLPRTTTETVPTSVHESTSTSQKPETLQQK
SFIVFCTASVIIIISLFVGILCWFYRSYPRKQDAENPAVQNNRATQQGQCERSGPVVVSCVEYGELDFQSRPQRDDR
GKAAESTSNEQDGVEYAAIIFPQQKQTPCGRIRNKQQVPAVKP

>Atlantic_salmon_(*Salmo_salar*)_PD-1

Source: GenBank XP_014005316

MFYYSRSKPLLLVVLIVLFNGHNWADKVVGIIGHEITIPFSFHNMSKASGNVQIGLYKNSKKISECNNDVHSCCSQS
HLCFSGKKVASFFIRNLTSVDNGTYWITLFQTSRDPPMLQSNEVTLILQSDGNTTESPSCTSNAEDDDISDSGKAN
NVYVLIIFAVLGVSIIALLIGLLGWFCWTYNRSPEGDQQENLKAKQQGSREASTSMSVYSVEYGVLDFNRRPSGEE
GNRHGREREGGVVRSAETVEYAAITFPPQQRVSYGR

>Barramundi_perch_(*Lates_calcarifer*)_PD-1

Source: GenBank XP_018528498

MVDNIVTKPLGLVLIVTAHMVFNGPSQGQVTGILGQNVTLEFRFNDTVIHHNSNVAIYINGMKKIAECLKTSVCS
GRGSISRQNTYVVYNISKLTVSDSDIYWASLFRDSGLKESDKVHLIVREENRTTTVPPAPTNATIIESTGSSFSFRIVT
VLVVSPVVLLAAVLPLIWCLVRTKDKKQQPSPQNCNPTVQETVEESNRVPPPSLVYSVLDFPKRPPAVLEMNPSDT
EYAAVSYLAEKRWM

>Medaka_(*Oryzias_latipes*)_PD-1

Source: GenBank XP_011472395

MVCDFFIKLTGLVLIVTAHTVLNDSSHTEVVGMLDKDVTLQFKFNKRKCNITTNSKFGVYTTGEKKISEYPHSKCFRI
NPGNNTVSYHITNLSMNHSNIYWISSFDDLLSRNCSNRVKLIIKENNNTSVPPVTTDHKEPPSSGSSSFFSSHVVM
LLVVTPVALFAAVLPFSICCLLRTRDQGDDEPQRLSNPTMQEVIEDYSSLPGPSVIYSVLDFPKRPPTVVEFDPNDT
EYAHVSYPPDQRQDSHRPKSKPDSTWCHM

>Mummichog_(*Fundulus_heteroclitus*)_PD-1

Source: GenBank XP_012737867

MVENRFTKPLGLVLIVAAHMVFCGLSHVRVIGIVDQNISLNFTFNTTIRNNSSIAVYKCISKQQKISEYPHCKSEIEVY
PEFSSVIFHLTNLSLNNTETYWATLFRNGKTETSNKVELIVQEDNRRTSVPPVPSTFTQTTTSGSSSFLSSNVVTVIV
VSPFLVLAAILPFLVICLAKPKGPTQEASQRTSNPPAQETLEVSVNSTGSSLVYSVLDFPKRPPSTVEFSPNNTEYASV
SYLPEKKPRSHTNK

<u>PD Ligand 1 (PD-L1) and 2 (PD-L2)</u>

Tetrapods (Class: Sarcopterygii [lobe-finned fishes plus tetrapods]; Infraclass: Tetrapoda)

>Human_(*Homo_sapiens*)_PD-L1

Source: GenBank Q9NZQ7

MRIFAVFIFMTYWHLLNAFTVTVPKDLYVVEYGSNMTIECKFPVEKQLDLAALIVYWEMEDKNIIQFVHGEEDLK
VQHSSYRQRARLLKDQLSLGNAALQITDVKLQDAGVYRCMISYGGADYKRITVKVNAPYNKINQRILVVDPVTSE
HELTCQAEGYPKAEVIWTSSDHQVLSGKTTTTNSKREEKLFNVTSTLRINTTTNEIFYCTFRRLDPEENHTAELVIPE
LPLAHPPNERTHLVILGAILLCLGVALTFIFRLRKGRMMDVKKCGIQDTNSKKQSDTHLEET

>Human_(*Homo_sapiens*)_PD-L2

Source: GenBank NP_079515

MIFLLLMLSLELQLHQIAALFTVTVPKELYIIEHGSNVTLECNFDTGSHVNLGAITASLQKVENDTSPHRERATLLEE
QLPLGKASFHIPQVQVRDEGQYQCIIIYGVAWDYKYLTLKVKASYRKINTHILKVPETDEVELTCQATGYPLAEVSW
PNVSVPANTSHSRTPEGLYQVTSVLRLKPPPGRNFSCVFWNTHVRELTLASIDLQSQMEPRTHPTWLLHIFIPFCII
AFIFIATVIALRKQLCQKLYSSKDTTKRPVTTTKREVNSAI

>Mouse_(*Mus_musculus*)_PD-L1

Source: GenBank NP_068693

MRIFAGIIFTACCHLLRAFTITAPKDLYVVEYGSNVTMECRFPVERELDLLALVVYWEKEDEQVIQFVAGEEDLKPQ
HSNFRGRASLPKDQLLKGNAALQITDVKLQDAGVYCCIISYGGADYKRITLKVNAPYRKINQRISVDPATSEHELICQ
AEGYPEAEVIWTNSDHQPVSGKRSVTTSRTEGMLLNVTSSLRVNATANDVFYCTFWRSQPGQNHTAELIIPELPA
THPPQNRTHWVLLGSILLFLIVVSTVLLFLRKQVRMLDVEKCGVEDTSSKNRNDTQFEET

>Mouse_(*Mus_musculus*)_PD-L2

Source: GenBank NP_067371

MLLLLPILNLSLQLHPVAALFTVTAPKEVYTVDVGSSVSLECDFDRRECTELEGIRASLQKVENDTSLQSERATLLEE
QLPLGKALFHIPSVQVRDSGQYRCLVICGAAWDYKYLTVKVKASYMRIDTRILEVPGTGEVQLTCQARGYPLAEVS
WQNVSVPANTSHIRTPEGLYQVTSVLRLKPQPSRNFSCMFWNAHMKELTSAIIDPLSRMEPKVPRTWPLHVFIP
ACTIALIFLAIVIIQRKRI

>Cattle_(*Bos_taurus*)_PD-L1

Source: GenBank NP_001156884

MRIYSVLTFMAYCCLLKAFTITVSKDLYVVEYGSNVTLECRFPVDKQLNLLVLVVYWEMEDKKIIQFVNGKEDPNV
QHSSYHGRAQLLKDQLFLGKAALQITDVKLQDAGVYCCLISYGGADYKRITLKVNAPYRKIYHTISVDPVTSEHELT

CQAEGYPEADVIWTSSDHQVLSGKTSITSSKREEKLFNVTSTLRINTTADKIFYCTFRRLGHEENNTAELVIPEPYLDP
AKKRNHLVTLGALFLCLSVTLAVIFCLKRDVRMMDVEKCDTRDMNSKQQNATQFEET

>Cattle_(*Bos_taurus*)_PD-L2

Source: GenBank NP_001278965

This sequence suggests an extra N-terminal exon and an extra C-terminal exon compared to consensus. TSA transcript database sequences reveal that the indicated C-terminus is common (e.g. GenBank GGVC01060549), but there is also a shorter version (e.g. GenBank GGVC01060571) in which the sequence ends with CQKLYSGEGK.*

MMRLPQSDPVQSMFLLLILSLGLQLQQTVALFTVTIPKEMYMVDYGSNVTLECDFDTGGPVELGILKASLQKVEN
DTVLLSERATLLEEQLPLGKALFLIPRIQLKDAGQYRCLIIYGIAWDYKYLTLKVKASYKKINTRHLKVPGTDEVELTCQ
AEGYPLAEVSWPNISIPTNTSHTKTSEGLYQVTSVLRLKPHPGRNFSCVFWNANVKELTSATIVQGPLEDPKIPSSS
LLHVFILSLIVAPIFIATVVALRKRLCQKLYSGEDSTKRSATMVRREVDRAI

>Platypus_(*Ornithorhynchus_anatinus*)_PD-L1

Source: GenBank XP_028911635

MKILPVFTFMLDWQLLNALFEVEVLKESFTVVYGSNVTMECSFPFKDRLDLEALSVYWDTEDDKHIVKFVKGKVD
LKIQHHSYRGRATLLKDKLLLGKAMLQITNVQLTDAGVYRCLIGYEGADYKWITLTVQAPYSKINQRISRNPTTLEY
EMTCQSEGYPEASVIWKNNYHEDLSDKAITNSSRGPDMLFNVTSTLGINATVNDTFYCFFWNKKAEENTTAVLII
PEQTKIPLMNNRNHLATLTAAILLGSLLLHLFCSKICVRVADVEKWDCQSVTCEGQRYPSTGKT

>Platypus_(*Ornithorhynchus_anatinus*)_PD-L2

Source: GenBank XP_028912158

MCLFLLVLILEMQLHLMPALFTVQIPKDFYTVDYGSNVTMECNFNVENQMDFNILLVFWDKDEKNIVKFAKGQE
DLKTQDEHYRGRATLLREELSSGKALLRISDVKITDAGQYRCLISYGGADYKYITLKVKASYKPVNTQILAAPGGDEE
DLICQAKGFPLAEVSWQNVSAPANTSYTRTPDGLYHITSVLRLPANSNKNVSCIFWNKDVNERTTANLDTPAPTE
VPLAEKTSLFYLFIPTCIIAFIFISALIFLRRPLCRELSNRKENRTRGNGLKLQQEKVRHKEDRGKNFFECLNL

>Chicken_(*Gallus_gallus*)_PD-L1

Source: GenBank XP_040512325

MMEKLLLLHIFLFCWRSLNALFTVEAPKSLYTAELGSNVTMECVFPVNGKLKFRDLSVIWEKKDEVRKDVYILLKG
KEDSGSQHSDFQGRIKLLKENLDFGQSLLQISNVKLRDAGLYHCLIEYGGADYKTINLKVQAPYRTITQEVVSTGDK

EWKLTCQSEGYPKAEVMWQNGECQDLTDKANTSYETGSDQLYRVTSTLTVKNRTCENFRCIFWNKEIQENTSAN
LYILDSADDVLWTESRRFVWPVLIVSALVGSVPITVCIRKARASKDCRTRMAKSSIHITKLSKDKGAHDCRGPSFED
AELKCKHK

>Chicken_(*Gallus_gallus*)_PD-L2

Source: GenBank XP_004949125

MLLRGAEELFTVEVPQQLYVVEYGSNVTMECRFPVNGSLNLGLLSVVWEQKRQGQLESREVYTLRNGKALTSSQ
HHDYMGRAALLRNELKLGRAILHITSVKITDAGSYLCLIDYQGADYKYITLEVKASYKIINTQKTREWNENKFALICQ
SEGFPLAEVFWQNEKNFSLSESANTTYALTADGLYNVTSILIINQNMNENYSCIFWNKELNENTSADIYSLALMST
QYGGQKSLILFIAATCVIVLVLLSVLTIFQKRKSFKNFRAKKDRKGKLSPTVTDENRHSSNSQTETSYLSTVAYSGESR
NVSL

>Swan_goose_(*Anser_cygnoides_domesticus*)_PD-L1

Source: GenBank XP_047906701

MEKPLLLCMFSFYWHFLNALFTVEAPQSLYTVELGNNVTMECTFPVNGKLKFGDLSVSWEKKEELGKDVYVLLK
GEEDFKSQHSDFRGRIKLLKENLKLGQSLLQIMDVKLRDAGFYRCLIDYGGADYKTINLKVQAPYRNITQGVVSTG
DKEWKLTCQSEGYPKAEVIWQNGEYEDLTDKADTSYETGSDQLYRVTSTLTIKNRTHENFRCIFWNKELQKNTSAI
LYIADSADDVLRTESRRFVGAILVVTALVGSVLFILCIRKASTSKDKRTCMANSSVNTANIQIEKT

>Swan_goose_(*Anser_cygnoides_domesticus*)_PD-L2

Source: GenBank XP_013052856

MFQILTMLLLEMQLWVVSGLFTVEVPQQLYVAAYGSNVTMECRFPVNGSLNLGLLSVVWEQKRQGQPESRDVY
TLHKGQALPPSQHHDYMGRAALLHDELKSGRAILQITRVKVTDAGSYLCLIDYQGADYKYITLEVTASYRIINTQV
MRKGNEEKFVFICQSEGFPLAEVFWQNEKNISLTGSANTTYMLTADGLYNVTSILMFKPNTSENYSCIFWNKELN
EETSAHFSTLALLSTLYSGQKSLILFIIATCVIVTVLLSALIIFQKRKSFKKCHAKKKTGKLNPSLTDENRDDYSPQTEAAY
FSTATFSRDSGNVSL

>Green_anole_lizard_(*Anolis_carolinensis*)_PD-L1

We did not find a sequence for green anole PD-L1, which does not have to mean it is absent.

>Green_anole_lizard_(*Anolis_carolinensis*)_PD-L2

Source: GenBank XP_016846530. This reference calls the sequence PD-L1, as does Hu et al. 2023 (doi: 10.4049/jimmunol.2300217), but our phylogenetic tree analysis clearly indicates PD-L2 identity (Supplementary file 2B).

MFGPLLVVLVQIQLHLVSALFTVEVLQPRYFAEHGGTVTMGCRFPVHDPFNLTNLSVLWQRKPSQGNEVKEVYK
LSKGQEDLRQQHADYQDRARVAPSELKIGLSMLCLNNVKIADSGIYVCLVHYEGSDLKYIYLDVKAPYKRIHFQKR
KEKTELNLTCQSQGYPLAEVSWQNEKSTNLSKFAFTTHQMTEDGLFNITSTLRVKPRNHGNYSCVFWNRELNEN
TSAHGAVSSLDFANKSNTEKKSLTVMVPICLLLLLVLIIFAFMKLRRKQFTNYYPQKEEDLDLQTKNLNAATIPD

>Green_sea_turtle_(*Chelonia_mydas*)_PD-L1

Source: GenBank GGMX01076332 (a TSA)

MENALLLCIFVSHWHFLNALFTVEVPQPQYIVEYGSNVTMECRFRVNGQLKLQDLSVIWEKKEEHTKEVYKLHK
GKENFNNQHSSYSGRVQLLKDKLQFGRSMLQVTSVKFTDAGTYLCLIGYEGADYKMIALQVKAPYRNITKRTVIV
QRTAGQNEWELTCQSKGYPKAEVIWHNGEHQDFSDKANTSYERGTDQLYSVTSTLKTNTSINETFHCIFWNKEF
KENTSAILIIPDDTVDSQRINSRHYFATAGAALVLFGSLLLFMLWLEKIRTGRCTNNAAVKVIKLSTNEEEGDCREISP
ENEELENVRIEVT

>Green_sea_turtle_(*Chelonia_mydas*)_PD-L2

Source: GenBank XP_007065662

MFRILPILILEVQLYLIRAFFTVEVPQLQYIAEYGSNVTMECRFPVDGQLNLKDLSVSWEQKGLKEQKPKEVYTLQK
GEEDLKSQHRDYRGRATLLRDKLNLGYSVLQITSVKLMDAGSYLCLIDYRGADYKYITLEVKASYTRINVQIMSEPA
EEELVLTCQSEGFPLAEVFWQNEKNLKVNVSVNTTYTLTTDGLYNVTSMLTFKPNASGNYSCTFWSKELNEETSYI
FTLARQTSVVGKKSLNLFIIPTCVIAVVLISALIIFLKRKSLTKLHAKKDKKRKCLQLQKEDKRHLNHEIQDLIMTNVTD
SRNCTDVCP

>Tropical_clawed_frog_(*Xenopus_tropicalis*)_PD-L1

Source: GenBank XP_017946448

MAMHRRLIIAVLFLCHCGVLSALFTVKAGKSHYTAEYGGKVNMECHFQVGKGTKADDVEVYWEYIAAEGGRKE
VIKLIRGTENLSAQHEDYRGRVRILKEELHKGHAVLQISNVELTDSGRYICIISAQGSDYKSMGLTVQAHYKEINIRVT
DIMASSGDIVKQIECQSIGYPEAEVTWVHGEKNLSSLVNTSYTVTAAKLFNVTSVVRVSSISNNTFTCMFWNEAL
QEATVLTFTIPVSGKSQSTNKRSFQLSLTAIAVAAALCIFVCVYIKKHDLENT

>Tropical_clawed_frog_(*Xenopus_tropicalis*)_PD-L2

Source: GenBank XP_031746478

MLVMKEEKMERKIFCLLCILLMILDGHSVLALFVVTAPRSSYTAQYGDTVQLICSFPPEENVYISKKLKVSWEHIDSF
QGKSQDVLMLTDGKLVLEKQSDTFRGRTTLLMEELNNGRAVLEITNVKLTDSGKYRCVLQLDGSDYKTISLKVKAS
YKIIDIYRSENEDLLTCQSLGFPIAEVSWQNNGDNVSLPSNFSQFLRPDGVYNITSTIRISRDVTQNYTCVFWNKEL
NEKTQASFHLEPYQGLTKDQFPMNTGKILTIISVILAVMVTMMVIYIKRKHCFRCFRKKGKRNYSTGSVCETMCPR
RNAKHEQDSLPDNPRK

>Tropical_clawed_frog_(*Xenopus_tropicalis*)_PD-L2

MLVMKEEKMERKIFCLLCILLMILDGHSVLALFVVTAPRSSYTAQYGDTVQLICSFPPEENVYISKKLKVSWEHIDSF
QGKSQDVLMLTDGKLVLEKQSDTFRGRTTLLMEELNNGRAVLEITNVKLTDSGKYRCVLQLDGSDYKTISLKVKAS
YKIIDIYRSENEDLLTCQSLGFPIAEVSWQNNGDNVSLPSNFSQFLRPDGVYNITSTIRISRDVTQNYTCVFWNKEL
NEKTQASFHLEPYQGLTKDQFPMNTGKILTIISVILAVMVTMMVIYIKRKHCFRCFRKKGKRNYSTGSVCETMCPR
RNAKHEQDSLPDNPRK

>Pleurodeles_waltl_(Iberian_ribbed_newt)_ XP_069096376_PD-L1

MGKMEKTFLVFTCIFKWNYINALFSVKVPESSYTVQIGTTVDLECNFPVIDTLRIKDLTITWLWRKSPETDQQEVYV
FHNGKEDISKQDSRYKGRATLVKDTLFTGRVVLQISNIRLSDRGTYQCLIGYGGADYRHIDLLVEAPYSTINTKLSTFL
TDSGMQERQMECQSQGYPKAEVIWLNEWKDISEKAITSYTVSTDLLHNVTSVLQIKGSENNTYHCIFWNKELQQ
NTTATFILSDEIDHTLWPNKRGYYGVILAGSLIVTIMLITVGLSQIYGSRSTS

>Pleurodeles_waltl_(Iberian_ribbed_newt)_ XP_069061916_PD-L2

Modified (shortened N-terminus)

MSPLIFMIVMVNLLHPQTALFSVEVPQSSFIVRVGSTVALECHFPLSGTLRVEELIVIWLHSAPSQPDQRDVIAFER
GKIDLSVQDASYKGRAILLNEALYKGQAVLQITNVKLSDSGIYSCIIGYGGADYKHIQLTVGASYERIHTQTEVVTGV
KELILTCGAKGFPLPDVFWQCNKVNISLPSNTSHTVTEDGLYNITSTIRSDLRLGKDCSCLFWNKDLQETTSAKLFTE
GTILFGKSLQLNLRFMFNLVNVGVVAWTAKVMDGLF

> **Microcaecilia__unicolor_PD_L1_XP_030048232**

MEKTVLILTFLSYWCSINALFTVEIARSVYVTQYGSTVNMTCTFPVAGGLRMKDLKVYWHQISSSQMVEKEIYAV
DSGKENLTLQDVSYRGRATLLKDELYKGQAVLEISNVKLTDAGTYRCLIIYGGADYKQVTLQVQVPFRKINISISGQE
KRELICQSEGYPKPEVMWQHEGQDLSDKANTTYRINPEQLYEVTSVLRVNTTVNATYRCIFWNKELQENSSESFS
FSDKDNLKSPGISEQKAYYGTMLAGCVLAVFSLALLIWMQRNGHCRKPTTLYKDRKKTTGCFVCERNPSNHIWK
VIRNFCLHLTHSIEKENTTEDHNIKIPIERELKMPLSMDITDASERLYIPSEENRAYPPRAPVNKVEVDVHHGQEMD
VKEM

> **Microcaecilia_ unicolor_PD_L2_XP_030049656**

MFTFQVLLLLESIVHLVIALFTIQVPRSVYVVQYDSTVNMTCTFPVAGGLRMKDLKVYWHQISSSQMVEKEIYAVD
SGKENLTLQDVSYRGRATLLKDELYKGQAVLEISNVKLTDAGTYRCLIIYGGADYKQVSLQVQASFTKITTWTERTPF
SATDVSLSCQSEGFPLPEVFWLSNGLNSNLSPSTSHTMTADGLYNVTSTLQIKPSSSQNYSCVFWNRELEERTMKL
FHLADEEPSDLQRSVLLFIVPICLIAIISSSAVVFFLKRKSLCACSKKKMNQETVTDAVSTKHEESCQLNPPT

Lungfishes (Class: Sarcopterygii [lobe-finned fishes plus tetrapods]; Subclass: Dipnoi)

>West_African_Lungfish_(*Protopterus_annectens*)_PD-L1

Source: GenBank XP_043917064 (supported by TSA GGXP01073155)

MNGLRFSLTFALYWCSVTALFTVEMTRSSYVAEHNTVATLECKFPIDKKYGIQDLKIFWHKIVNSSLELEVFKFFNGS
EDFLMQDVIYKGRARLRQALLGNGRAVLEINPVKVTDTGVYRCLIELHGADYKQGTLEVKASYRNIEKHVTMIGS
VNGKQQLELFCHSNGFPKAEVLWNSGTQNLSAQAKTSFKRDPEDLYLMTSFIRVIPSYNDTYSCIFMSNNLRESIS
NFTYLDWQIPDSVDGYRLIYVGAAIAIIIVVMIVVTIILIWRKRKDWSLLHTGIFQGKKKLPCEYRCKSNCTECWTTL
RHLFRSTTHIASHTVLNKEKSDTACHHHNQGKQLEASQLNKLLEEDSQTRVPVQSSRTTVSVHCEKEP

Sharks and Rays (Class: Chondrichthyes [cartilaginous fish]; Subclass: Elasmobranchii)

>Small-spotted_catshark_(*Scyliorhinus_canicula*)_PD-L1

Source: GenBank XP_038660173

MKIISLVLILGVHLPLMTAIFTVTAPRLSYTASYGNNITVECRFPVESNFNSNQIKLYWHHILSDGSSQLVYK
LFNGKPALQDQSQEYSERVFMLLDELRSGRAVLEINRVRVSDAGTYRCVIDLNGVDYKETALEVTASYGE
IETIKTKEKKETELICQSMGYPLAEVVWYYENGSDINETANTTHFTGTNGLYNIRSVIRIKEETDDNYLCM
FWIKELNMNTSAILQNKEMEETEEKKDELLSTNRSYLIAAIAIPVTVLLGVIIIVSILLKSSQYRQGKIKVCET
SLLI

>Nurse_shark_(Ginglymostoma_cirratum)_PD-L1

Source: GenBank GIWU01097433 (a TSA)

MVTAPRSSYIASYGNNITMECQFPVESNFNANQIKVYWHYVLGDGTSRLVYQLINGEPALQAQPREYRERAFLLL
DELQRGRAVLEINQVRVSDAGTYRCLIDLNGVDYKETELEVTASYKNIETIKTKETKETEFVCKSVGYPLAEVIWYYA
NGSDINETANTTHFTSSDGLYNVRSVLRIKAETNDAYLCMFWNKELNMNTSAVLHIKEAEKTGDIFNFPSTKRSYLI
ATIVVPVIILLGFIMIVSCILKSRQSEKGNKKASETSLLL

>Thorny_skate_(*Amblyraja_radiata*)_PD-L1

Source: GenBank XP_032874294

MKIISLLFILGAHLPLDTALFMVTAPRLSYTASYGNNITMECIFPVESNFHMNTLKVYWYHILDNGTSQLVYKLLNG
KAALQDQSFEYRERVFLAMDKLFNGRAVVEITQVRVSDAGTYRCIIALNGVDYKETALKVTASYNDIKTLNTKEQT
ETEFSCQSAGYPLAKVMWYHANGTDLNKTANTTHFTGSNGLYNIRSILRIRAETNETYLCMFWIEELNTSTSAVLQ
IKEIKRKDNDQIPTFTTMRSYLIVAITIPSIVLLGVIMILICVIKSRLCHKGKTYKNEYGLFNA

Bichirs and Reedfish (Class: Actinopterygii [ray-finned fish]; Order: Polypteriformes)

>Gray_bichir_(*Polypterus_senegalus*)_PD-L1

Source: GenBank XP_039615495 (supported by multiple TSAs, including GKLT01349496)

MWASGVHAGPVRVGAAPEKIMKIFQLLVLISQWPALPALFTVEMLKPLYTVEFKDTVRIECRFSINDNFQQDHLS
VFWHQLLPNNTDLEVFRMFRGTESLKSQHSRYKGRASLMTEPLKDGLAMLQISDVQIEDSGRYRCLIDLNGDPD
YKETTLSVKASYNNSAAKFFVQRRPENKSEVELICQSQGYPPVKVLWRDSKGRNLTSHAMSNGSFGTNHLFQVSS
RLNVPASTLETFSCTFWNESLKNPFTAFLTIPDVIQTSGAMETSHLPPLAALFSVLIVTVVTMVVVGVILWKKKHPC
QAHIRKSHRRMSELHIPINSVQDPIQNTEDLQELLKERYMNHGPEECMTWSEEVPLFGQLASSSGQPQRLQEVL
QETDRLHILEEAFQEDQSSLCRALARTWADSNASDPYGVKKYSLILLLQLTDSVWDFFGELVKQLIHRDKISTEELQ
MMLMGAGSILLILDHKGVNSTVSEESLRTILHKYQNLEILLTKHPEGQQIS

>Reedfish_(*Erpetoichthys_calabaricus*)_PD-L1

Source: GenBank XP_028660782

MWAAGIHAGPVRVGAASEKIMKIFQLLVLISQWPALPALFTVEMLKPLYTVEFKDTVRIECRFSINDNFQQDHLSV
FWHQLLPNNTDLEVFRMFRGTESLKSQHTRYKGRASLMTEPLKDGLAVLQISNVQIEDSGRYRCLIDLNGDPDYK
ETTLSVKASYNNSAAKFFVMRRPENKSEVELICQSQGYPPVKVLWRDSKGRNLTSHAMSNGFFGTNHLFQVSSR
LNVPAPTLETFSCTFWNESLKNPFTAFLTIPDVIQTSGAMETSHLPPLVVPFSVVIVTVVTMVVVGVILWKKKRPCQ
AHIGKSHRRMSELHIPINSVKDPIRNTEDLQELLKERYMSHAAEGYMTWSEEVPLFGQLANSSSQPQRLQEVLRE

TERLHVLEEASQEDQSSLCRALARTWADSNASDPYGVKKYSLIFLLQLTDSVWDIFGELIKQLIPRDKSSTEELQVM
LMGAGSILLILDQNGVNNTASEESLRTILHKYQNLEILLTKHLEGQHIS

Sturgeons and Paddlefishes (Class: Actinopterygii [ray-finned fishes]; Order: Acipenseriformes)

>Sterlet_sturgeon_(*Acipenser_ruthenus*)_PD-L1

Source: GenBank XP_033870049 (confirmed by TSA GGQL01027991)

MKIILLVIVLEFHWPVLQALFTVEMAKALYLAEFGNTVKMECRFPTGGSLDSINVYWHRMLSNGSEYEVYTLLNG
NEDLQSQHPEYKGRAHMKPDLLRKGRAELEISNVKISDSGSYRCLIKMGGADYKQATLSVKAPYKIKTHVKELPKN
ADHKDAELSCESEGYPPVQVLWRDGNDQDLSKKASSKHSVTADQLFHISSLLIVNASSNNTYHCILWNETMEETY
NASLRIPADIKTVDPPIADGVSSTAYWATVAILLVTIVMVGLIILMWKKKIKCSQLRAMSQRHPEEENEVHIHINTN
EDDGSLQQIENLRELLKAKYTASSFSDWAIGEWHSGFCEQFLPQRLVNSDGQTQPLTTLIPQPKGALLLEGDPLSG
KSRLCQALAAMWADSTGEDTFGARECELVVLLQCEGSIGNIYEEMIRQLLPEKRFSEADLEAVLMDHFNTLLILDG
YSKGNTTLDESLRSLIREGYSIRILVTSLPEGCEHILDCFGSVLKLQNGVAADIFL

> Paddlefish_(*Polyodon_spathula*)_PD-L1

Source: GenBank XP_041113561

The here presented sequence is encoded by Chr. 1, but there is a similar gene on Chr. 2.

MKMILLVVVLAFHWPVLRALFTVEMIKTLYLAEFGNTVKMECRFPTGGSLDSINVYWHRMLSNGSEYEVYTLVN
GNEDLQSQHPVYKGRAHMKPDLLRKGRAELEISNVKISDSGSYRCLIKMGGADYKHATLSVTAPYKIKTHVKELPK
NADHKDAELSCESEGYPPVKVLWKDGNDQDLSKKATSKHSVTADQLFHISSLLIVSASTNNTYHCILWNETMEET
YNASLRIPADIKTVDPAIADGLRSNAYWAAVVIVLMTIITAGVIILMLKKKIKCSQLRAVLRRYLNEENEAHINEEDV
MGKLNH

Gars (Class: Actinopterygii [ray-finned fishes]; Order: Lepisosteiformes)

>Spotted_gar_(*Lepisosteus_oculatus*)_PD-L1

Source: GenBank XP_015192216 (confirmed by TSA GFIM01002838, although that predicts a longer N-terminus)

MDNSLLIIFQVAMWPMFPALFTVDMTESSFLAEFRGNVTMECRFPTGGGETLSSLRVYWHRILPEPLLEVYKLES
GKEDLSTQHPRYKGRVNLQKDRLKQGQAVLQMSNLTISDSGKYRCIVEQGGADYKEATLNVRAAYTPIKTDIRRT
PDDEKVELTCESEGYPLAKVIWSDASGLNLTSNANESIFLLPDQRYHIKSCVIVNNTVNNTYTCTFWNESMINYAT
NLTIPDDMKKTEEQSPAGRKSSTIVWAIMVVAAIVVTVAILVNQRFKDVKGLKRSKRKEEDFDALSTALTINSEVPT
SVVTHEEGNLKA

Teleost fish (Class: Actinopterygii [ray-finned fishes]; Infraclass: Teleostei)

>Asian_bonytongue_ (*Scleropages_formosus*)_PD-L1

Source: GenBank XP_018606800

MAHHLLLLQVGFFLPSVQALFTVEVASPSYTAEFSGDVAMECKFGPMDSKSSLSVRWQRILPKPLLRVYNLENGQ
EDLSFQDSQYRGRVQLMKDKLSSGRAILNISNVKINDSGTYECLVEMVGADFKRTTLTVKASYKSVKKTVRRTGN
DEVELSCGSEGYPLAAFVWSDQSGRRFDELNASSTVTSDKLFHITSSVIVDTSTNNYTCTILEENHWGPSAAFSIPA
EIPGTKSGKPPVRVAWLTLIVVAAMVVAFLFYHRRKGLKRNRAPRMKHCLLNDSHAAACLPQGRNDDVQVGIE
DEVEVEGLRKVLMSRYEVLSTNTEVSNLRAFCENELPRRLRNREGLTVGAADLLPGVRETVLLEEENGNDKTAVAR
SLASAWAGNSQWDPFGVRQLPLLVLVACEGAVDDLFREAALQVDQKSQFTAGDLQKLLTETIDSLLVLDGYREGS
RELDESLTTFLSSRKMCRVLITARSGQCDSLRGSCRTMLKLCSASTGNEGLS

>Tarpon_(*Megalops_atlanticus*)_PD-L1

Source: GenBank KAG7477045

The underlined sequence was used for structure modeling.

MDTALLILQIVLWPGVPALFTVEVTKPFHLAEFRGNVTMECIFLPGGSEESLSVFWRRILPEPPMEVYKLENGKED
LSSQAPQYRDRVRLLRDELRRGRAALQISHLRINDSGTYQCLVEMGGADYKQTTLTVKASYKSINKSVRRSGRDEV
ELLCQSQGYPQASVVWTDGRGRNLTEEANTTAVISSDQLFHVTSGITVRTSNNTYTCAFVKEGLMGQSVTFHIPE
EIPWKQTGKGSFVVAVVLITVTFAVCVASILIYRRRAGYKGRNTAETMDCLIQKDPFPARMPLETSIEVQTSVEENK
GAVEILREVLRRRYATLSSDAEEAESRRSFCEKELPQRLRNRDGLPLGVTALLPDVGETILLEGEPGSGKTSVAVSLAS
AWAQNSERDPFGVKQIDLLILVTCQESRGGLFQEMMSQLSLGREFTADALREVVTGPVETLLVLDGYKEGNGELD
ESLRRFLRERQTCRVLVMAQPGQCDNMTECLRTVLMLCSEVKEGQGLC

>Oriental_weatherfish_(*Misgurnus_anguillicaudatus*)_PD-L1

Source: GenBank XP_055036527 (confirmed by TSA GGUH01057907, including the large exon; no TSAs were found that suggest a shorter C-terminus)

MKGPLLLMCQALLCSAVSGLFTVDVEQKSYESELHGDVKLVCLFSQVKSLSDLLVIWHRIEPLPEINVYRYERGKEK
QNYTNVVFGDRAQLIHEQLSQSRAVLHLKKLRIKDSGTYQCVVKYQQDDVDYKHITLSVTALNTPIKKSVRKTEVE
DEVELSCEFAGYPLAEVLWSDGQTVNLTEKSSKRTRVTDEDLINITSRLTVRRDLVRNYTCSFLTEGKVRQTTTFIIPH
EIPASSSHHYVWIGSVIVVLLVIIFISIIYHKRKNGQKDKRSKSSKCPPPFPQTAANNDCLLTIYENTPHPCDITREEHA
EKLQTLRDALIQRYSQPITDTEMNTRLMSYCSNVLPHVLHLREGQAVNVSSIFPDKRQTILLLGKPGSGKTTTAQIL
SSCWAQSLTIDPWNIKDLQLVVSVNCRGTNGDFFQLVKSNIPNETPLDVSDIRETLLGSTDCLVILDGYKEGNRDL
DETLGMFLKERQTCRILVMSHPGECPSLENKVGTVLNLIHKSDDKSET

>Zebrafish_(*Danio_rerio*)_PD-L1

Source: GenBank WIB11275 (Supported by TSA GFIL01036161, except that this TSA does not have the extra intron at the N-terminus and its reading frame starts with encoding MQGSMKRT)

MCSIHQGSMKRTLVIIFQALLWPAVLSASFTVNVPRSTYEAELNGDVRLECVFSALKRSSDITVIWSRVHPKPDVNI
YWLDKGKEIHNHTSSAFHKRAQLISHLLRENRAVLHLKKLRIKDSGTYQCIVEGDEVDYKQITLNVTAPFSPVRKSL
RKAGEDEVELSCESQGFPSAQVYWSDGQKLNLTLFSNTSVSSTDEDLILIVSKLKVERELVNNYTCTFIVKGEIQQTA
TFSIPEEIPLHGSALFVWIGAVIVVLLAVIFISIILHRRKYGQKNRRNEASKCAYLFPQSSSVNTDSLLTVNENRTHARE
KSSTEKTASLRDSLTQQYSQLYTESEMKRKLRSYELHSRDGHCVNISSVIPEKGQILLLLGDSGCGKTTFTQILSYSWA
SRSQTDPFNTRRLRLLLLLHCSQNKGNLNQIINSSVQHERPVDVKQSLKGPEDCLLILDDYQEGNKDLEEFLKDHQ
TCRVLITSRPGVCPNLEKTVRTVLHLIHKPEESST

>Atlantic_salmon_(*Salmo_salar*)_PD-L1a

Source: GenBank XP_013980930 (confirmed by TSA GEGY01086932; no indications were found for PD-L1 in salmonid fish with a longer C-terminus). The gene is located on Chr. 1. The Atlantic salmon PD-L1a and PD-L1b molecules share 79% amino acid identity.

MEQAFLLVLQVVLWPTLAALFTVEVDSPFHVAEFHGVVTMGCRFQPGGQGPNLSVIWHRIWPPPVVEVYRLEN
RQEDLTSQNPQYRGRVRLVTEEMTNGWAKLEVSMLRINDSGTYQCLVEMSGADYKQTTLTVKASYKTIVKSMKR
RRGDEVELVCESEGYPLATMTWKDRSLRNIKSNDTTVRTPDQLFQVTSKITVKSSDKNNYTCALVEKGEVPKGPSA
RFDIPDEIDLPAIHSKCNTLSIVLGTSLTVALVIAAAIFGCRRWRGRPRDPSTPSTNTLLD

>Atlantic_salmon_(*Salmo_salar*)_PD-L1b

Source: GenBank NM_001141351 (confirmed by TSA GGAQ01013916; no indications were found for PD-L1 in salmonid fish with a longer C-terminus). The gene is located on Chr. 13. The Atlantic salmon PD-L1a and PD-L1b molecules share 79% amino acid identity.

MEQTFLLVLHVVLWPTLAALFTVEVDSLSHVAEFYGDVTMGCRFQPGSWDPNLSVIWQRVQPLPDVEVYRLDN
GQENLTSQNFQYRGRARLVSEELTNGWAKLHVSRLRINDSGVYRCLVEMGGADYKQTTLTVKATYKTIIKSMQRR

GGGEVELACESEGYPLATINWRDKSLRNIKSNDTVVKTPNQLFHVTSKITVKYSEKNNYTCAFVEKGEAPKGPSAR
FDIPDEIPVIESKPNTLSIVLGTTLTVAMIIVATIFGYRRQKGRLRTLKL

>Barramundi_perch_(*Lates_calcarifer*)_PD-L1

Source: GenBank XP_018525925 (supported by TSA GAQL01310075)

MDWALFVILQVIFQPSLSVLFTVEAERPTYTSEFGGDVVMGCRFQPKPSNPQADLKVSWRWLGSTSPWEVYQ
MNNGKEHSASPEYQGRVRLLTEQLNEGWAKLQVSRLRINDSGVYQCLVQTGEGADYKAITLSVIAPYKTLTKRIVK
AAEGDKVLLTCQSEGYPESPVVWQDGRLQSLKSNTTTVSTPEQLFKVTSQIHVNSSDKDNYTCHFLNGGQSATF
HIPDEIPVPQVKNDALIVVLSIGLIMVVIIVAVLMYRRRKGSNTPSTRNLLVDGRGGPVSAVACSQTDKENEEERTIF
NEERIEENLGAILKAHYSDVSFSTEVRHHSGSFDVEELPHRLQNNEGQAMKPQALLPEAGEILLLQGPPGSGKTTV
AHILVSSWVEGPAHSLANVLDLSILRLLLYIDCSAAKGDLFQEVVTQLSLMEKTSTEDELRTLLTRSSDTLLLLDGYRE
GNQFFDESLKRFLCERGGCRVLVVTCPEHCPTLKETIGTRGVLKLQTQTVKY

>Medaka_(*Oryzias_latipes*)_PD-L1

Source: GenBank XP_020563390 (supported by TSA GFIO01030254).

MDWVFAIILQVMIQPSLSVLFTVEAEQTMYSSEFGGEVVMGCRFSKKATQPNSDLKVTWHWTSSGLHQELIRLD
NTADYSVPPKYQGRVKLLTEELKNGWAKLQLSNLRINDSGTYQCLVQTTDGTDYKTMTLSVKAPYKTVTKRIERAE
QNKVLLTCESEGYPKSEVVWTDGNLQNQHPNTSFVSTPEQLFKITSQILVSSSEENNYTCSFKSDKKSTTFYIPDDL
PTPPEKSDAVIIVVLTVLIIVIMAAGGILYYKRKAFRTIGTTKCLPVDPDSSVSGACLQKEKEIKNVEIDMPEDTGLY

From the genomic sequence, with help of FGENESH software (http://www.softberry.com/), also a longer sequence can be predicted as shown below, but for this sequence no TSA support could be found.

MDWVFAIILQVMIQPSLSVLFTVEAEQTMYSSEFGGEVVMGCRFSKKATQPNSDLKVTWHWTSSGLHQELIRLD
NTADYSVPPKYQGRVKLLTEELKNGWAKLQLSNLRINDSGTYQCLVQTTDGTDYKTMTLSVKAPYKTVTKRIERAE
QNKVLLTCESEGYPKSEVVWTDGNLQNQHPNTSFVSTPEQLFKITSQILVSSSEENNYTCSFKSDKKSTTFYIPDDL
PTPPEKSDAVIIVVLTVLIIVIMAAGGILYYKRKAFRTIGTTKCLPVDPDSSVSGACLQKEKEIKNVEIDMPEVVKRHH
KKTTDSCKKDIRCLLFGKKKYRPLLDLLPEAAEMHLLEGHPHSIQGTDGNPHPTQPSSRTDGPSCASARHAFSPCA
SRKPWWRKTSCRNEKWKKARGTDDFAVQTK

>Mummichog_(*Fundulus_heteroclitus*)_PD-L1

Source: GenBank XP_012726531 (supported by TSA GCES01014700)

MDWVIAVVVVVVLQSFLQPSVAALFTVEAEQATYMSEFGGNVVMGCKFSSNPANPHRDLKVNWHRKTNGIYE
EVIRLEDNLENSASPKYQGRVELLTGELKNGWAKLKISHLKMNDSGTYQCLVQTAEGNDYKEIALSVEAPYKSVSK
RIERRAEGDKVVLTCHSQGYPKSSVVWHDGHLQKHNSSTTATATPDGLYNVTSRIEVSSSAKNNYTCNFTHDGYS

ATFHIPDDITLPKGKNDALITVLCIGLILTAIGLGVVTYRRRKGARTPSTRNCLVNDEERSLSAAACLGMDKESAVEEK
VITEEENLRSHLKAHYSEFSLTTKTKHHCDSFAAEELPHRLQNNEGLPVRLQDLLPNAGEILLLEGPPRSGKTTAAHI
LLSSWTGADSGFLDAGFLDLLVYVNCDTMKGDLFQEATAQLALSEKISAEQLRTVLSRSNKTLLLLDGYKEGNHFC
DETLRRFLSERGSCRVLVTSCLGDCPVLKQTLKTEGTLTLQMQSAKY

Src homology region 2 domain-containing phosphatase-1 (SHP-1)

Tetrapods (Class: Sarcopterygii [lobe-finned fishes plus tetrapods]; Infraclass: Tetrapoda)

>Human_(*Homo_sapiens*)_SHP-1

Source: GenBank BAC81775

MVRWFHRDLSGLDAETLLKGRGVHGSFLARPSRKNQGDFSLSVRVGDQVTHIRIQNSGDFYDLYGGEKFATLTEL
VEYYTQQQGVLQDRDGTIIHLKYPLNCSDPTSERWYHGHMSGGQAETLLQAKGEPWTFLVRESLSQPGDFVLSV
LSDQPKAGPGSPLRVTHIKVMCEGGRYTVGGLETFDSLTDLVEHFKKTGIEEASGAFVYLRQPYYATRVNAADIEN
RVLELNKKQESEDTAKAGFWEEFESLQKQEVKNLHQRLEGQRPENKGKNRYKNILPFDHSRVILQGRDSNIPGSD
YINANYIKNQLLGPDENAKTYIASQGCLEATVNDFWQMAWQENSRVIVMTTREVEKGRNKCVPYWPEVGMQ
RAYGPYSVTNCGEHDTTEYKLRTLQVSPLDNGDLIREIWHYQYLSWPDHGVPSEPGGVLSFLDQINQRQESLPHA
GPIIVHCSAGIGRTGTIIVIDMLMENISTKGLDCDIDIQKTIQMVRAQRSGMVQTEAQYKFIYVAIAQFIETTKKKLE
VLQSQKGQESEYGNITYPPAMKNAHAKASRTSSKHKEDVYENLHTKNKREEKVKKQRSADKEKSKGSLKRK

>Mouse_(*Mus_musculus*)_SHP-1

Source: GenBank AAD00152

MVRWFHRDLSGPDAETLLKGRGVPGSFLARPSRKNQGDFSLSVRVDDQVTHIRIQNSGDFYDLYGGEKFATLTEL
VEYYTQQQGILQDRDGTIIHLKYPLNCSDPTSERWYHGHISGGQAESLLQAKGEPWTFLVRESLSQPGDFVLSVL
NDQPKAGPGSPLRVTHIKVMCEGGRYTVGGSETFDSLTDLVEHFKKTGIEEASGAFVYLRQPYYATRVNAADIEN
RVLELNKKQESEDTAKAGFWEEFESLQKQEVKNLHQRLEGQRPENKSKNRYKNILPFDHSRVILQGRDSNIPGSD
YINANYVKNQLLGPDENSKTYIASQGCLDATVNDFWQMAWQENTRVIVMTTREVEKGRNKCVPYWPEVGTQ
RVYGLYSVTNSREHDTAEYKLRTLQISPLDNGDLVREIWHYQYLSWPDHGVPSEPGGVLSFLDQINQRQESLPHA
GPIIVHCSAGIGRTGTIIVIDMLMESISTKGLDCDIDIQKTIQMVRAQRSGMVQTEAQYKFIYVAIAQFIETTKKKLEI
IQSQKGQESEYGNITYPPAVRSAHAKASRTSSKHKEEVYENVHSKSKKEEKVKKQRSADKEKNKGSLKRK

> Philippine_flying_lemur_(Cynocephalus_Volans)_SHP-1

Source: GenBank XP_062960725

MLSHGWFHRDLGGLDAETLLKSRGVHGSFLARPSRKNQGDFSLSVRVGDQVTHIRIQNSGDFYDLYGGEKFATLT
ELVEYYTQQQGVLQDRDGTVIHLKYPLNCSDPTNERWYHGHMSGGQAEILLQAKGEPWTFLVRESLSQPGDFV
LSVLSDQPKAGPGSPLRVTHIKVMCEGERYTVGGSETFDSLTDLVEHFKKTGIEEASGAFVYLRQPYYATRVNAADI
ENRVLELNKKQESEDTAKAGFWEEFESLQKQEVKNLHQRLEGQRPENKSKNRYKNILPFDHSRVILQGRDSNSPG
SDYINANYVKNQLLGPDENAKTYIASQGCLEATVNDFWQMVWQENTRIIVMTTREVEKGRNKCVPYWPEVGT
QRVYGLYSVTNCGEHDTAEHKLRTLQVSPLDNGDLVREIWHYQYLSWPDHGVPSDPGGVLSFLDQINQRQESLP
HAGPIIVHCSAGIGRTGTIIVIDMLMENISTKGLDCDIDIQKTIQMVRAQRSGMVQTEAQYKFIYVAIAQFIETTKK
KLEVMQSQKGQESEYGNINYPPAMKNAHAKAARTSSKHKEDVYENLQSKNRKEEKVRKQRSADKEKSKGSLKRK

>Tupaia_(*Tupaia_chinensis*)_SHP-1

We are not showing the tupaia SHP-1 sequence because we suspect there is an error in the GenBank XP_027625017 and ELV09744 sequences in regard to the exon 11 region.

>Cattle_(*Bos_taurus*)_SHP-1

Source: GenBank XP_005207188

MVRWFHRDLSGLDAETLLKGRGVHGSFLARPSRKNQGDFSLSVRVGDQVTHIRIQNSGDFYDLYGGEKFATLTEL
VEYYTQQQGVLQDRDGTIIHLKYPLNCSDPTSERWYHGHMSGGQAETLLQAKGEPWTFLVRESLSQPGDFVLSV
LSDQPKAGPGSPLRVTHIKVMCEGGRYTVGGSETFDSLTDLVEHFKRTGIEEASGAFVYLRQPYYATRVNAADIEN
RVLELNKKQESEETAKAGFWEEFESLQKQEVKNLHQRLEGQRPENKSKNRYKNILPFDHSRVVLQGRDSNIPGSD
YINANYVKNQLLGPDENAKTYIASQGCLEATVNDFWQMVWQENTCVIVMTTREVEKGRNKCVPYWPEVGSQ
RVYGPYTVTNCGEHDTTEYKLRNLQVSPLENENLIREIWHYQYLSWPDHGVPSEPGGVLSFLDQINQRQESLPHA
GPIIVHCSAGIGRTGTIIVIDMLMESISTKGLDCDIDIQKTIQMVRAQRSGMVQTEAQYKFIYVAIAQFIETTKKKLE
VMQSQKGRESEYGNITYPPAMKNAHAKASRTSSKHREDVYENVHSKNKKEEKVKKQRSADKEKNKGSLKRK

>Platypus_(*Ornithorhynchus_anatinus*)_SHP-1

Source: GenBank XP_028910310

MVRWFHRDLSGLDAETLLKGRGVHGSFLARPSRKNQGDFSLSVRVGDQVTHIRIQNSGDFYDLYGGEKFATLTEL
VEFYTQQQGVLQDRDGTIIHLKYPLNCSDPTSERWYHGHISGAQAEALLQAKGEPWTFLVRESLSQPGDFVLSVL
SDQPKAGPGSPPKVTHIKVMCEGGRYTVGGSETFDSLTDLVEHFKRTPIEEASGAFVYLRQPYYATRVNAADIENR
VLELNKKHEAQETAKAGFWEEFESLQKQEVKNLYERQEGQRPENKGKNRYKNILPFDHTRVVLQGRDSSIPGADY
INANYVKNQLVSVDENAKTYIASQGCLDATVNDFWQMVWQENSRVIVMTTREVEKGRNKCVPYWPEVGSCKP
YGLYSVQNCAEHDAPEYKLRSLQVSPLDNGDLVREIWHYQYLSWPDHGVPSEPGGVLSFLDQINQRQESLPHPG

PIVVHCSAGIGRTGTIIVIDMIMESISTKGLDCDIDIQKTIQMVRAQRSGMVQTEAQYKFIYMAIAQFIETTKQKLDI
LQAQKGRVESEYGNIGYPPAMKNAHAKACRTSSRQKEDVYENLQGKAKKEVKKQRSADKEKNKGSLRKK

>Chicken_(Gallus_gallus)_SHP-1

Source: GenBank NP_001026655

MVRWFHRDLSGLEAEALLKGRGVHGSFLARPSRKNQGDFSLSVRVGDQVTHIRIQNTGDFYDLYGGEKFATLSEL
VEYYTQQQGSLQDKDGTIIDLRYPLNCSDPTTERWYHGHLSGVAAESLLQAKATPWTFLVRESLSKPGDFVLSVLT
DQPKPGSDAPPAGATSASGARLKVTHIKIMCENGRYTVGGSEKFDSLADLVEHFKKTGIEEVSGSFVYLKQPYYAT
RVNAADIENRVHELNKKSLSEETSKAGFWEEFDSLQKQEAKQLFDRHEGQRPENKSKNRYKNILPFDHSRVILQG
RDPNIPGSDYINANYVKNNLISPDECPKTYIASQGCLDTTVNDFWQMVWQENTHIIVMTTREVEKGRNKCVPY
WPEAGSTKEYGPYLVENVGEHDALEYKLRHLCVCPINDSEAVREIWQYQYLSWPDHGVPSEPGGVLSFLDQINQ
KQESIPNAGPILVHCSAGIGRTGTIIVIDMIVETISTKGLDCDIDIQKTIQMVRAQRSGMVQTEAQYKFIYMAICQFI
ETTKRKLEVIQSQKSKQNESEYGNITYPPAVKNMHAKVSRKSSKQKETIYENLGKKEEKVRKQLSSEKKLKSSLKKK

>Swan_goose_(*Anser_cygnoides_domesticus*)_SHP-1

Source: GenBank XP_013054968

MVRWFHRDLSGLEAEALLKGRGVHGSFLARPSRKNQGDFSLSVRVGDQVTHIRIQNTGDFYDLYGGEKFATLSEL
VEYYTQQQGSLQDKDGTIIDLRYPLNCSDPTTERWYHGHLSGAAAESLLQVKATPWTFLVRESLSKPGDFVLSVLT
DQLKPGPDAPSAGASSASGARLKVTHVKIMCENGRYTVGGAEKFDSLADLVEHFKKTGIEEVSGSFVYLKQPYYAT
RVNAADIENRVHELNKKSVSEETSKAGFWEEFDSLQKQEAKQLFDRHEGQRPENKSKNRYKNILPFDHSRVILQG
RDPNIPGSDYINANYVKNNLISPDECTKTYIASQGCLDATVNDFWQMVWQENTRIIVMTTREVEKGRNKCVPY
WPEVGSTKEYGPYLVENTGEHDALEYKLRHLCVCPKNNGDAVREIWHYQYLSWPDHGVPSEPGGVLSFLDQIN
QKQESIPAAGPILVHCSAGIGRTGTIIVIDMIVETISTKGLDCDIDIQKTIQMVRSQRSGMVQTEAQYKFIYMAICQF
IETTKKKLEVIQSQKSKQNESEYGNIAYPPAMKNAHAKASRKASNRQKEESTVYENLGKKEEKVRKQLSSDKKLKG
SLKKK

>Green_anole_lizard_(*Anolis_carolinensis*)_SHP-1

Source: GenBank XP_062827862

MSRWFHRDLSGIDAEALLKARGIHGSFLARPSRKNKGDFSLSVRVGDQVTHIRIQNTGDFYDLYGGEKFATLSELV
EYYTQQQGCLQDKDGTIIDLRYPLNCSDPTTERWYHGHLSGPAAETLLQTKATPWTFLVRESLSKPGDFVLSVLTD
QPKAGSDATPAGATSTPKEQFKVTHVKVMCEKGKYTIGGPEKFSNLSDLVDHFKKAGIEEASGSYVYLRQPFNATR
VNAADIEDRVQMLNKRSQIEEAAKGGFWEEFDSLQKQETKNLHERNEGQRPENKCKNRYKNILPFDHSRVVLQ
DRDGNVAGSDYINANYIKNTMVSPEECTKTYIASQGCLEATINDFWQMVWQENSRIIVMTTREVEKGRNKCVP
YWPEVGSSKEYRPYIVENFGEHDALEYKLRQLRISPIDDGEAVRDIWHYQYLSWPDHGVPSEPGGVLGFLDQINQ
KQESIPAAGSIVVHCSAGIGRTGTIIVIDMIVDMISTKGLDCDIDIPKVIQMVRSQRSGMVQTEAQYKFIYMAICQFI

ETTKKKLDVMQSQKGRPNESEYGNITYPPAQKTSHAKASRKSSKQKEEPTLYENLDKAKAKKEEKGTKPLGKEKKP
KGSLKKK

>Green_sea_turtle_(Chelonia_mydas)_SHP-1

Source: GenBank XP_027687584

MVRWFHRDLSGLQAEALLKGRGVHGSFLARPSRKNQGDFSLSVRVGDQVTHIRIQNTGDYYDLYGGEKFATLSE
LVEYYTQQQGSLQDKDGTVIDLRYPLNCSDPTTERWYHGHLSGPAAESLLQSKATPWTFLVRESLSKPGDFVLSVL
TDQLKAGPEAPPTGAAGTPGARLKVTHVKIMCENGRYTVGGAEMFDSLADLVEHFKKTGIEEVSGSYVYLKQPY
YATRVNAADIENRVHELNKQSLAEETAKAGFWEEFDSLQKQEAKHLYDRHEGQRPENKGKNRYKNILPFDHSRVI
LQGRDPNIPGADYINANYIKNQLISPDEFTKTYIASQGCLDATVNDFWQMVWQEKSRIIVMTTREVEKGRNKCV
PYWPEEGSTKEYGPYIVENIGEHDALEYKLRQLSLSPVDNCKAVREIWQYQYLSWPDHGVPSEPGGVLSFLDQIN
QKQESIPEAGPIVVHCSAGIGRTGTIIVIDMIVETISTKGLDCDIDIQKTIQMVRAQRSGMVQTEAQYKFIYMAICQ
FIETTKKKLDVMQSQKGRSKESEYGNIAYPPALKNQHAKASRKSSKQKEEPTVYENLENTRGKKEERVRKQRSSEK
EKLKGSLKKK

>Tropical_clawed_frog_(*Xenopus_tropicalis*)_SHP-1

Source: GenBank NP_001116928

MVRWLHQNISGLEAEARLKSRGVHGSFLVRPSKKKPEDFSLSVRVEDSVTHIRIQNTGDYYDLYGGEQFASLSELV
EFYTGQAECLQDTSGNRIELKYPLNCSDPTNERWYHGYLSGPDAEKLLQKTGEPWTFLVRESRSNPGDFVISILTPE
QKDGVHKVTHVKINTEKNKDTYNIGREKFESLTDLIEWHKKKPIEEATGSHLFLKKPCYSTRVNAADIENRMKELN
KKSEQDEAAKAGFWEEFDALQKQENKVHNDRKEGQRPENKSKNRYKNILPYDHTRVILKCGDNNTPGSDYINA
NYVNNLLWAEGEPPKRFIACQGCLNSTTGDFWEMVWQENSRVIVMTTKEIEKGRTKCVPYWPDVGTNMKEFG
RFLVQLMSERDTKEYKVRLLRVSLTESTEKSRDIHHYQYLTWPDHGVPADPGGVLSFLEEVNSMQESMPRAGAIV
VHCSAGIGRTGTIIAIDMLLDLIQMKGVDSDIDVQKTIQMVRNQRSGMVQTEAQYKFIYSAIAQFIDSTKHKLKA
MENRNATESEYGNLGASLTKMKVTRSSSKNQPEGTVYENLNAGKQKEEVVKKKSSEKQRSSVKKK

Lungfishes (Class: Sarcopterygii [lobe-finned fishes plus tetrapods]; Subclass: Dipnoi)

>West_African_Lungfish_(*Protopterus_annectens*)_SHP-1

Source: GenBank XP_043935559

MVRWFHRDISGIDAETLLKSRGVPGSFLARPSRKNQGDFSLSVRLTDSVTHIRIQNTGDYYDLYGGEKFATLSELVE
YYTTQHGTLQDKDGTLIQLKYPLNCSDPTSERWYHGHLSGSNAEVLLKENGFSGSFLVRESLSKPGDFVLSVVTDE

LKEVNGEKKPRVTHIKILSEDGKYTLGGKDRFDCLPDLVEFYKKECIEEASGAKIRLRQPCYATRINAADIESRVQVLA
KSSEDTESAKAGFWEEFDRLQKQETKNLLSRAEGQRPENKTKNRYKNILPFDETRVILKDRDPNVLGSDYINANYV
ENRLFGGGKMYIATQGCLLATVNDFWQMVWQENTRVIIMTTMEVEKGRNKCVPYWPEMDGAKDFEQYYVR
CTQENILKEYKIRVLEVCPMDDGDNIREIWHYQYTAWPDHGVPSEPGGVLGFLEVVNKKQQELGSVGPMVVHC
SAGIGRTGTIIVIDMLVDTIDLKGVDCDIDIQKTIQMVRSQRSGMVQTEAQYKFIYMSVAHYIETVKKRLEAGQNP
SNESEYGNIRTLGLKNPHAKAARKSSHQKEESQVYENLGSLKGKKEDKMKKQKSSEDKVKASSKTSSIKKK

Sharks and Rays (Class: Chondrichthyes [cartilaginous fish]; Subclass: Elasmobranchii)

>Small-spotted_catshark_(*Scyliorhinus_canicula*)_SHP-1

Source: GenBank XP_038678278

MVRWFHREISGNDAEKLLKARGVHGSFLARPSRQNPGDFSLSIRVGEQVTHIRIQNTGDYYDLYGGEK
FATLSELVQYYTEQENVLQDKDGTIIELKYPLNCSDPTNERWYHGHLSGSAAERLLSDKKTPWVYLVRES
LSQPGDFVLSVANDGEKTQDGNKKVTHIKIMCQDGRYTVGGPQKFDTLTDLVEYYKRSVIEQIDGTKIH
LKQPYYATRINAADIENRVQELNKTENPDVAKGGFWEEFDALQKQEDTKKQSRTEGQRPENKSKNRYK
NILPFDNTRVILQIKEEDPPFSDYINANYIKSETMVNRLGKIIPKTCIATQGCLLATVNDFWQMVWQQNS
RIIVMTTREVEKGRNKCVPYWPELNSENVYGPFTVRHVLEREAIDYKIRELDVTLTEKPNSKPRKIFHYQY
LSWPDHGVPSEPGGVLSFLEQVNNKQDEIRDAGPIVVHCSAGIGRTGTIIVIDMLVDKIDRQGVDCDIDI
QKTIQTVRNQRSGMVQTEAQYKFIYMAVSQYIETTQKKLALMENDKQAESEYGNIQYPAQQKAQHSKI
SRKTSNQGEDPTLYENISKAKKDESVQKQRSDEKGKSGKQKSASVKKK

>Nurse_shark_(Ginglymostoma_cirratum)_SHP-1

Source: GenBank GIWU01179041 (a TSA)

MVRWFHRDISGTEAEKLLKARGVHGSFLARPSKQNPGDFSLSIRVGEQVTHIRIQNTGDYYDLYGGEKFATLSELV
QYYTEQENILQDKDGTIIDLKYPMNCSDPTNERWYHGHVSGSTAEKLLGQKKVPWMYLVRESLSNPGDFVLSVA
IDGQKESDGEMRQKVTHIKIMCQDDKYTVGGPNKFDSLTDLVEYYKQSVIEQMDGTKVHLKQPYYATRINAADIE
NRVQELNKTQSPEVGLKGGFWEEFDALQKQDDAKHQTRTEGQRPENKSKNRYKNILPFDSTRVVLQTQEGSPPF
SDYINANYIQYETLAEKEGRMVPKTCIATQGCLLATVNDFWQMVWQENSRIIVMTTREVEKGRNKCVPYWPEP
NTEKEFGPYTVRSVEEREAIDYKIRELDISITGKGSKARRVYQYQYLSWPDHGVPTEPGGVLSFLEQVNSQQAKLR
NAGPIVVHCSAGIGRTGTIIIDMLVDRIDRQGVDCDIDIQKTIQAVRSQRSGMVQTEAQYKFIYMAVSQYIETTQR
KLQLMQETSDAGSEYGNIRYQAQKNSQHGRLSRKVSNNHSEDPTVYENLSKAKKDDRVRKQKSDEKAKSGKPK
PGSVKKK

> Pacific_electric_ray _(*Tetronarce_californica*)_SHP-1

(We could not find SHP-1 for thorny skate)

Source: GenBank GFBV01005387 (a TSA; absence of the last YEN may be common in rays and skates, as suggested by tblast similarity searches in the GenBank TSA dataset. For *SHP-1* in this species we could not determine the intron positions because we could not find a genomic sequence report.)

MRWFHRDISGSQAEKVLKTRGVHGSFLARPSKQNPGDFTLSIRVSDQVTHIRIQNTGDYYDLYGGEKFATLSELVQ
FYTDQSNVLQDKDGTVIELKYPLNCSDPTSERWYHGHLSGTAAETLLAEKQVPWTFLVRESLSHPGDFVISVATDG
EREVEGQKRLKVTHIKIMFKDERFTVGGALNFETLSDLVEYYRNSAIEQLDGMKVLLKQPYHATRINAADIENRVE
QLNKPHSKEGAKAGFWEEFDALQKQDDLKTLSREEGQRPENKSKNRYKNILPFDKTRVILKTTPDSPPFTDYINAN
YIQSETTVLKGGKVVPKTCIATQGCLNATVNEFWQMIWQENSSIIVMTTREVEKGRNKCVLYWPAVDEEKEFGV
YYVRNLGEREAVDYKVRNLQVWPSDKSASPRNIYHYQYMSWPDHGVPSEPGGVLSFLEQVNQRQESLPDAGPII
IHCSAGIGRTGTIIVIDMLIDKINRQGLDCDIDIQKTIQSVRSQRSGMVQTEAQYKFIYMAVSQYIETTQRKLQFVE
GNKAVESEYGNISYPPQKSTHTKVSQRASNPGADESGRTRKEEKVRKQRSAEERKAGSVRKK

Bichirs and Reedfish (Class: Actinopterygii [ray-finned fish]; Order: Polypteriformes)

>Gray_bichir_(*Polypterus_senegalus*)_SHP-1

Source: GenBank XP_039619695

MVRWFHRDLSGIEAETLLKSRGVHGSFLARPSKKNAGDFSLSVKVGEVVTHIRIQNTGDYYDLYGGEKFATLSELV
EYYTTEHGTLQDKDGTIIELKYPLNCSDPTNERWYHGHLSGPAAEKLLWEKGEPGTFLVRESLSKPGDFVLSILSDEI
REINKEQKYRVSHVKIMCQNDRYTVGGTESFETLTDLVEHFKKVGIEEVSGAWVYLKQPYYSTRVNAGDIDKRVQI
LAETGEVANKAGFWEEFDALQKQEAKVKKSREEGMRPENKSKNRYKNILPFNETRVILKSGDPNIIGSDYINANYV
KNLLTESNFRKVYIATQGCLMTTVNDFWQMIWQERSRVIVMTTREVEKGRNKCVPYWPEVETMKEYGKYIVHH
ISEHDALDYKIRILEISPVDNSDDTREIWHYQYLSWPDHGVPEEPGGVLSFLEQVNAKQQEILGAGPIVVHCSAGI
GRTGTIIVIDMLVDTINIKGVDCDIDIQKSIQMVREQRSGMVQTEAQYKFIYMAVSQYIETTKRKLEEIGKTETEYG
NLKYPTAQHGKASRKSSRNKDDTYENAGLKGGKKDDKMKKQKSEEKGKAGNPGSVRKR

>Reedfish_(*Erpetoichthys_calabaricus*)_SHP-1

Source: GenBank XP_028665778

MVRWFHRDLSGIEAETLLKSRGVHGSFLARPSKKNAGDFSLSVKVGEVVTHIRIQNTGDYYDLYGGEKFATLSELV
EYYTTEHGTLQDKDGTIIELKYPLNCSDPTNERWYHGHLSGPAAEKLLWEKGEPGTFLVRESLSKPGDFVLSILSDEI
REINKEQKYRVSHVKIMCQNDRYTVGGTESFETLTDLVEHFKKAGIEEVSGAWVYLKQPYYSTRVNAGDIDKRVQI

LAETGEVANKAGFWEEFDALQKQEAKVKKSREEGMRPENKSKNRYKNILPFNDTRVSLESGDPNIIGSDYINANY
VKNLLTESNFRKVYIATQGCLMTTVNDFWQMIWQERSRVIVMTTREVEKGRNKCVPYWPEVETMKEYGKYIVH
HLSEHDALDYKIRILEISPIDNSDDTREIWHYQYLSWPDHGVPEEPGGVLSFLEQVNAKQQEILGAGPIVVHCSAGI
GRTGTIIVIDMLVDTINIKGVDCDIDIQKSIQMVREQRSGMVQTEAQYKFIYMAVSQYIETTKRKLEEIGKTETEYG
NLKYPTGQHGKASRKSSRNKDDTYENAGLKGGKKDDKVKKQKSEEKGKAGKPGSVRKR

Sturgeons and Paddlefishes (Class: Actinopterygii [ray-finned fishes]; Order: Acipenseriformes)

>Sterlet_sturgeon_(*Acipenser_ruthenus*)_SHP-1

Source: GenBank GKEF01314296 (a TSA; the N-terminus is missing in this TSA)

ELVTHIRIQNTGDFYDLYGGEKFATLSELVEYYTTEHGTLQDRDGTLIELRYPLNCSEPTSERWYHGHLSGQAAEKLL
WQKGEEGTFLVRESLSKPGDFVLSILTGESKEVNGEQRNRVSHVKIMCQDGRYTVGGAEQFENLTDLVEHFKQSG
IEEVSGTRVYLKQPYYSTRINAADIDSRVQVLTKTNDTNEASKSKAGFWEEFDALQKQEAKLKKSRNEGMRPENK
SKNRYKNILPFDETRVILQSGDPNVVGSDYINANYISNMLMESCDRKKFIASQGCLATTVNDFWEMVWQERSCV
IVMTTREVEKGRNKCVRYWPEPEEMKEFGSYQVRHVSEHDAVDYTLRVLEISPLNQSDDLREIWHYQYLSWPDH
GVPAEPGGVLSFLEQVNSKQSELRSAGPIVVHCSAGIGRTGTIIVIDMVINTINTKGLDCDIDIQKSIQMVREQRSG
LVQTEAQYKFIYMAVSQYIETTKRKLDEMKKTETEYGNLHFPMAQHSKASRKLSKNKEEVYENLGSKGGKKEDKV
KKQKSEEKDKAKSGKSGSVRKR

> Paddlefish_(*Polyodon_spathula*)_SHP-1

Source: GenBank XP_041124975

MVRWFHRDLSGIEAEALLKSRGIHGSFLCRPSRKNQGDFSLSVRVGEMVTHIRIQNTGDFYDLYGGEKFATLSEL
VEYYTTEHGTLQDQDGTLIELRFPLNCSEPTSERWYHGHLSGQAAEKLLWQKGEEGTFLVRESLSKPGDFVLSILT
GESREVNGEQRNRVSHVKIMCQDGCYTVGGVERFENLTDLVEHFKQSGIEEVSGTRVYLKQPYYSTRINAADIDS
RVQVLTKTNDADEASKSKAGFWEEFDALQKQEAKLKKSRNEGMRPENKSKNRYKNILPFDETRVILQSGDPNVI
GSDYINANYVSNLLMESCDRKKFIASQGCLATTVNDFWEMVWQERSRVIVMTTREVEKGRNKCVRYWPEPED
TKEFGSYLVRHVSEHDAVDYTLRVLEISPLNQSDDQREIWHYQYLSWPDHGVPTEPGGVLSFLEQVNSKQRELR
GAGPIIVHCSAGIGRTGTIIVIDMVINTINTKGLDCDIDIPKSIQMVREQRSGLVQTEAQYKFIYMAVSQYIETTKRK
LEEMKKTETEYGNLHFPMAQHSKASRKLSKKKEDVYENLSLKGGKKEDKVKKQKSEEKDKAKSGKSGSVRKR

Gars (Class: Actinopterygii [ray-finned fishes]; Order: Lepisosteiformes)

>Spotted_gar_(*Lepisosteus_oculatus*)_SHP-1

Source: GenBank GFIM01033722 (a TSA)

MACGWFHRDLSGIDAEALLKARGVHGSFLARPSKKNQGDFSLSVRVGELVTHIRIQNTGDYYDLYGGEKFATLSEL
VEYYTGEHGTLQDKDGTLIELKYPLNCSDPTTERWYHGHLSGSNAEKLLWEKGEPGTFLVRESLSKPGDFVLSVLT
DEKKDVGGQIRGRVSHIKIMCENDRYTVGGPDMFDTLTDLVEHFKRKGIEELNGTMIYLKQPYYSTRVNAADIDS
RVQVLEQTAGREDAQSSNKAGFWEEFDALQKQEAKLRKSRDEGMRPENKSKNRYKNILPFNDTRVVLESGDPN
VIGSDYINANYVKNLVREPGDQKVYIATQGCLQTTVNDFWQMVWQERSRVIVMTTREVEKGRNKCVPYWPEP
EGTKEYGNYLVRNVSEHDATDYKIRILEISPQDESEDTREIWHYQYLSWPDHGVPLEPGGVLSFLTQVNNKQLEIR
GAGPMIVHCSAGIGRTGTIIVIDMLVETIDTKGLDCDIDIQKCIQMVREQRSGMVQTEAQYKFIYLAVSQYIETTKK
KLEALGSAEMEYGNLSFPNKHTKASRKVSKNKEEVYENLGGKGKKDVKKQKSEEKKSGSVRKR

Teleost fish (Class: Actinopterygii [ray-finned fishes]; Infraclass: Teleostei)

>Asian_bonytongue_ (*Scleropages_formosus*)_SHP-1

Source: GenBank XP_018593687

MRWFHRDLSGLEAEALLKARGVHGSFLARPSKKNQGDFSLSVRVGDMVTHIRIQNTGDFYDLYGGEKFATLSELV
EYYTTEHGTLQDKDGTVIELKYPLNSSDPTTERWFHGHLSGPNAEKLLVERDEPGTFLVRESLSKPGDFVLSALTEE
RTKGPDGPRRVSHIKIMCQNYKYTIGGSEKFDSLSDLVEHYKRKGIEELSGTWVYLKQPYYSTRVNAADIENRVKV
LDQTAARENGGEGEKKSKAGFWEEFDALQKHETKVKKSRSEGMRPENKSKNRYKNILPFDDTRVILQSADPSVV
GSDYINGNYVKNKLSDSGHQKVYIATQGCLATTVNDFWQMVWQENSRVIVMTTREVEKGRNKCVPYWPELEG
TKEVGSYLVTCLSERDAADYKVRVLEIIPMDQSDCPRTIWHYQYLSWPDHGVPEEPGGVLSFLTQVNSKQMEFAD
AGPMIIHCSAGIGRTGTIVVIDMLVEIIDTKGLDCDIDIPKFIQMVREQRSGMVQTEAQYKFIYLAVLQYIETTKSKM
QACENLETEYGNLSLQPKHQKVSRKVSKNKEDVYENLGLKGKKDVKKQKSEEKKSGSVRKR

>Tarpon_(*Megalops_atlanticus*)_SHP-1

Source: GenBank KAG7469368

MVRWFHRDLSGLEAEAMLKARGVHGSFLARPSKKNQGDFSLSVRVGEMVTHIRIQNTGDYYDLYGGEKFATLSE
LVEYYTVEHGTLQDKDGTIIELKYPFNCSDPTTERWYHGHLSGSNAEKMLGERDEPGTFLVRESLSKPGDFVLSVL
TEERSRGPNSPRRVSHIKIMCQNDRYTIGGSEMFDSLTDLVEHFKRKGIEEMSGTWVYLRQPYYSTRVNAADIDS
RVRVLDKTTDRDEEGGGEKKSKAGFWEEFDYLQKQEAKVKKSRDEGMRPENKSKNRYKNILPFDETRVALQSGD
PSIIGSDYINANYVKNKLREPGDQKVYIATQGCLATTVNDFWQMVWQEQSRVIVMTTREVEKGRNKCVPYWPE
LEGSKEVGRYIVTCLSERDATDYKIRILEITPLDQSDAPREIWHYQYLSWPDHGVPHEPGGVLSFLSQVNSKQMEF
PNAGPMIIHCSAGIGRTGTIVVIDMLIETIDTKGLDCDIDIQKSIQMVREQRSGMVQTEAQYKFIYLAVSQYIETTKT
KLQALGNTETEYGNLSLQPKQKASRKVSKNKEDIYENLGAKGKKDVKKQKSEEKKSGSVRKR

>Oriental_weatherfish_(*Misgurnus_anguillicaudatus*)_SHP-1

Source: GenBank XP_055066874

MVRWFHRDLSGLEAEAVLKTRGFHGSFLARPSKKNVGDFSLSVRVGNMITHIRIQNTGDYYDLYGGEKFATLAEL
VEYYTADHGVLQDKDGTVIELKYPLNCSDPTTERWYHGHLSGPNAEKLLRERNEPGTFLVRESLSKPGDFVLSALT
DEQTSAGGRVSHIKIMCNNDRYTVGGKDIFDSLTDLVEHFKRTGIEELSGTMVYLKQPYYSTRLNAADIESRVKQL
DQTSEREEKEGSDKKIKAGFWEEFDALQKLETKVTKSRDEGMRPENKSKNRYKNILPFDETRVILANADPNVVGS
DYINANYVINKLMEIDHQKVYIACQGCLATTVNDFWQMVWQEKTGVIVMTTREVEKGRNKCVPYWPTTQGER
KEAGRYVVTLLSEMDATDYKVRVMELSATHRKEAPRTIWHYQYLSWPDHGVPQEPGGVLSFLEQVNGKQHELS
SNGPMIIHCSAGIGRTGTIVVIDMLIDTIDAKGLDCDIDIQKCIQMVRDQRSGMVQTEAQYKFIYMAVLHYIESTK
VTHKAVLETETEYGNLSIQPKHQKASRKTSSKKNEDVYENLGAKGKKDVKKQKSEDKKSGSVRKR

>Zebrafish_(*Danio_rerio*)_SHP-1

Source: GenBank NP_956254

MVRWFHRDLSGLDAEAVLKSRGVHGSFLARPSKKNVGDFSLSVRVGEIITHIRIQNTGDYYDLYGGEKFATLAELV
EYYTGDHGTLQDKDGTVIELKYPLNCSDPTTERWYHGHLSGPNAEKLLRERNEPGTFLVRESLSKPGDFVLSALTD
DQTSSGRRVSHIKIMCNNDRYTVGGKDQFDNLTDLVEHFKRVGIEELSGTMVYLKQPYYSTRLNAADIQSRVNQL
DQTSEREKMDGADKKIKAGFWEEFDALQKLETKVTKSRDEGMRPENKSKNRYKNILPFDETRVILENADPNVVG
SDYINANYVINKLMVTNPQKTYIACQGCLATTVDDFWQMMWQEDSRVIVMTTREVEKGRNKCVPYWPTTEG
ESKEVGRYVVKLLSEMDAADYKVRVVELTAPHRNEAPRKIWHFQYLSWPDHGVPQEPGGVLSFLDQVNRKQEE
LRSSAPIVIHCSAGIGRTGTIVVIDMLIDSIDAKGLDCDIDIQKCIMMVRDQRSGMVQTEAQYKFIYLAVLQYVEST
KVTRRAIMETETEYGNLSIQSKHPKASRKASSKKNEDVYENLGAKGKKDVKKQKSEEKKGGGSVRKR

>Atlantic_salmon_(*Salmo_salar*)_SHP-1

Source: GenBank NP_001133922

MVRWFHRDISGLDAESVLKSRGIHGSFLARPSRKNQGDFSLSVRVGELVTHIRIQNTGDFYDLYGGEKFATLSELVE
YYTAENGILQDKDGTIIELKYPFNCSDPTTERWYHGHLSGPNAEKLLWERDEPGTFLVRESLSKPGDFVLSVLTEEK
SKASSGGRRVSHIKIMCQNDRYTVGGTEMFDTLADLMEHYKRKGIEEMSGTWVHLKQPYFSTRVNAADIDSRV
RLLDQTAERENEGDKKSKAGFWEEFDALQKQETKVKKSREEGMRPENKSKNRYKNILPFDETRVILSSGDPNIIGS
DYINANYVTNKLQESGDQKVYIACQGCLATTVNDFWQMVWQEKTRVIVMTTREVEKGRNKCVPYWPEMQGS
KEVGPYVVTCVSERDATDYKVRVMEISPLDQSDSVRTIWHYQYLSWPDHGVPEEPGGVLSFLSQVNLKQAEFTN
AGPMIIHCSAGIGRTGTIVVIDMIIKTIDTKGLDCDIDIQKSIQMVRDQRSGMVQTEAQYKFIYLAVSEYIEASKTYN
KGAETEYGNLQFKHQPASRKVSKNKEQELYENLAKGKKDVKKQKSEEKKSGSVRKR

>Barramundi_perch_(*Lates_calcarifer*)_SHP-1

Source: GenBank XP_018527957

MVRWFHRDITGLQAEEMLKSRGIHGSFLARPSKKNVGDFSLSVRVGELVTHIRIQNTGDFYDLYGGEKFATLSELV
DYYTAENGILQDRDGTVIELKYPLNCSDPTTERWYHGHLSGPNAEKLLSARDEPGTFLVRESLSKPGDFVLSVQTD
ERSKTGGKRVSHIKIMCQNDRYTVGGSEMFDTLTDLVEHYKRKGIEEISGNWIYLKQPYYSTRVNAADIDNRVKEL
DQTKQQQEGEGEKSKAGFWEEFDALQKLEAKVKKSREEGQRPENKSKNRYKNILPFNDTRVILQDADPNVVGSD
YINANYVKNTLWESGDQKVYIATQGCLATTVNDFWQMVWQENTSVIVMTTREVEKGRNKCVPYWPELHSSKE
MGPYVVTCESEREAADYKVRVLEIALMDKPKQSRQVWHYQYLSWPDHGVPQQPGGVLSFLTQVNAKQAEYPH
AGPMIIHCSAGIGRTGTILVIDMILETIDTLGLDCDIDIPKYIQMVREQRSGMVQTEAQYKFIYLAVSEYIQTTKAKD
SASMETETEYGNLQLKQPASRKVSKNKEDVYENLSKGKKDAKKSKTDKKSGSVRKR

>Medaka_(*Oryzias_latipes*)_SHP-1

Source: GenBank XP_023820218

MVRWFHRDITGLQAEEILKLRGIDGSFLARPSKKNVGDFSLSVRVGDQVTHIRIQNTGDYYDLYGGEKFATLSELV
DYYTSDSGTLQDKDGTLIQLKYPLNCSDPTTERWYHGHLSGPNAEKLLTVRDEPGTFLVRESLSKPGDFVLSALTDE
ISKSGTKRVSHIKIMCQNDRYTVGGSETFDTLTDLVDYYKRKGIEEVSGNWVYLKQPYYSTRVNAADIDSRVRQLD
QTPQEQLEEEGQKSKAGFWEEFDALQKLEAKVKKSREEGQRPENKSKNRYKNILPFNDTRVILQDTDPNVVGSD
YINANYVKNKLQELGDQKVYIATQGCLATTVNDFWQMVWQENTRVIVMTTREVEKGRNKCVPYWPELHSSKE
VGPYIVTCDTEREAVDYKVRVLEISPMAKPQLSRTIWHYQYLSWPDHGVPQEPGGVLSFLIQVNGKQAEYPEAGP
IIVHCSAGIGRTGTILVIDMIIDTIDTLGLDCDIDIPKYIQMVREQRSGMVQTEAQYKFIYLAVSEYIQTTKTKDCAYM
ETDTEYGNLQLKHQPASRKVSKNKEDVYENLSKAKKEGKKAKPDKKSGSVKKR

>Mummichog_(*Fundulus_heteroclitus*)_SHP-1

Source: GenBank XP_012725752

MVRWFHRDISGLEAEDILKSRGIHGSFLARPSKKNVGDFSLSVRVGDGVTHIRIQNTGDFYDLYGGEKFATLSELVE
YYTAENGILQDKDGTIIELKYPVNCSDPTTERWYHGHLSGPNAEKLLSTREEPGTFLVRESLSKPGDFVLSVLTDEIN
KNGSKRVSHIKIMCQNDRYTVGGSDLFDTLTDLVEFYKRKGIEEISGNWVHLKQPYFSTRVNAADIDSRVRQLDLT
SDKPVESEGEKSKAGFWEEFDALQKMEAKVKKSREEGQRPENKSKNRYKNILPFNDTRVILQNADPNVVGSDYI
NANYVKDKLWESGARKVYIATQGCLATTVNDFWQMVWQENTHVIVMTTREVEKGRNKCVPYWPDLHASQES
GAYVVSCESEREATDYKVRLLHIAPVNQAKLSRPIWHYQYLSWPDHGVPQEPGGVLSFLTQVNAKQAEYPEAGP
MIIHCSAGIGRTGTIVVIDMILETIDTIGLDCDIDIPKYIQMVREQRSGMVQTEAQYKFIYLAVSEYIQTTKAKESASK
ETETEYGNLQLKHQPASRKVSKNKEDVYENLSKGKKDMKKSKSEKKTGSVKKK

Src homology region 2 domain-containing phosphatase-2 (SHP-2)

Tetrapods (Class: Sarcopterygii [lobe-finned fishes plus tetrapods]; Infraclass: Tetrapoda)

>Human_(*Homo_sapiens*)_SHP-2

Source: GenBank NP_002825

MTSRRWFHPNITGVEAENLLLTRGVDGSFLARPSKSNPGDFTLSVRRNGAVTHIKIQNTGDYYDLYGGEKFATLAE
LVQYYMEHHGQLKEKNGDVIELKYPLNCADPTSERWFHGHLSGKEAEKLLTEKGKHGSFLVRESQSHPGDFVLSV
RTGDDKGESNDGKSKVTHVMIRCQELKYDVGGGERFDSLTDLVEHYKKNPMVETLGTVLQLKQPLNTTRINAAEI
ESRVRELSKLAETTDKVKQGFWEEFETLQQQECKLLYSRKEGQRQENKNKNRYKNILPFDHTRVVLHDGDPNEP
VSDYINANIIMPEFETKCNNSKPKKSYIATQGCLQNTVNDFWRMVFQENSRVIVMTTKEVERGKSKCVKYWPDE
YALKEYGVMRVRNVKESAAHDYTLRELKLSKVGQGNTERTVWQYHFRTWPDHGVPSDPGGVLDFLEEVHHKQ
ESIMDAGPVVVHCSAGIGRTGTFIVIDILIDIIREKGVDCDIDVPKTIQMVRSQRSGMVQTEAQYRFIYMAVQHYIE
TLQRRIEEEQKSKRKGHEYTNIKYSLADQTSGDQSPLPPCTPTPPCAEMREDSARVYENVGLMQQQKSFR

>Human_(*Homo_sapiens*)_SHP-2L-pseudogene

Source: GenBank NC_000001.11 (Chr. 1; the depicted fragment is encoded from Chr. 1 positions 1063077-1063202 located at the 5' side of the AGRN gene)

RWFHPNISRVEAEKLFLSRGQRGDFLARPSESSPGGFTLSVR

There are multiple expressed sequence tag (EST) cDNA reports that include this region but do not seem to encode a functional protein, such as the 667 and 802 nt ESTs reported a Genbank accessions BG945396 and BI601978. It is clear from our (homology search) investigation that this region does not include a consensus-type intact *SHP-2L* gene, but we have not investigated in detail how many *SHP-2L* gene fragment remnants are situated in this region, or how many different transcripts have been reported for this region.

>Mouse_(*Mus_musculus*)_SHP-2

Source: GenBank NP_001103462

MTSRRWFHPNITGVEAENLLLTRGVDGSFLARPSKSNPGDFTLSVRRNGAVTHIKIQNTGDYYDLYGGEKFATLAE
LVQYYMEHHGQLKEKNGDVIELKYPLNCADPTSERWFHGHLSGKEAEKLLTEKGKHGSFLVRESQSHPGDFVLSV
RTGDDKGESNDGKSKVTHVMIRCQELKYDVGGGERFDSLTDLVEHYKKNPMVETLGTVLQLKQPLNTTRINAAEI
ESRVRELSKLAETTDKVKQGFWEEFETLQQQECKLLYSRKEGQRQENKNKNRYKNILPFDHTRVVLHDGDPNEP
VSDYINANIIMPEFETKCNNSKPKKSYIATQGCLQNTVNDFWRMVFQENSRVIVMTTKEVERGKSKCVKYWPDE
YALKEYGVMRVRNVKESAAHDYTLRELKLSKVGQGNTERTVWQYHFRTWPDHGVPSDPGGVLDFLEEVHHKQ
ESIVDAGPVVVHCSAGIGRTGTFIVIDILIDIIREKGVDCDIDVPKTIQMVRSQRSGMVQTEAQYRFIYMAVQHYIE
TLQRRIEEEQKSKRKGHEYTNIKYSLVDQTSGDQSPLPPCTPTPPCAEMREDSARVYENVGLMQQQRSFR

(in mouse we have not found *SHP-2L* or its remnants, but we only screened superficially with TBLASTN analysis and cannot completely exclude the possibility that some pseudogene fragments are left)

> Philippine_flying_lemur_(Cynocephalus_Volans)_SHP-2

Source: GenBank XP_062941402

MTSRRWFHPNITGVEAENLLLTRGVDGSFLARPSKSNPGDFTLSVRRNGAVTHIKIQNTGDYYDLYGGEKFATLAE
LVQYYMEHHGQLKEKNGDVIELKYPLNCADPTSERWFHGHLSGKEAEKLLTEKGKHGSFLVRESQSHPGDFVLSV
RTGDDKGESNDGKSKVTHVMIRCQELKYDVGGGERFDSLTDLVEHYKKNPMVETLGTVLQLKQPLNTTRINAAEI
ESRVRELSKLAETTDKVKQGFWEEFETLQQQECKLLYSRKEGQRQENKNKNRYKNILPFDHTRVVLHDGDPNEP
VSDYINANIIMPEFETKCNNSKPKKSYIATQGCLQNTVNDFWRMVFQENSRVIVMTTKEVERGKSKCVKYWPDE
YALKEYGVMRVRNVKESAAHDYTLRELKLSKVGQGNTERTVWQYHFRTWPDHGVPSDPGGVLDFLEEVHHKQ
ESIMDAGPVVVHCSAGIGRTGTFIVIDILIDIIREKGVDCDIDVPKTIQMVRSQRSGMVQTEAQYRFIYMAVQHYIE
TLQRRIEEEQKSKRKGHEYTNIKYSLADQTSGDQSPLPPCTPTPSCAEMREDNARVYENVGLMQQQKSFR

> Philippine_flying_lemur_(Cynocephalus_Volans)_SHP-2L

Source: Modified from XP_062960308

MTSRRWFHPNISGVEAEKLLLSRGQHGSFLARPSKSSPGGFTLSVRRHEEVTHIKIQNTGDYYDLYGGEKFATLAE
LVQHYTGRHGGPLRERSGAPVELRHPLGCQDPTSERWYHGHLSGKEAEKLLMEKGRPGSFLVRESQSKPGDFV
LSVLTQELDKVQGVDRRARVTHIMIRFQSDGKYDVGGGERFDTLRELVEHYRKNPMVEKSGAVVHLKQPLKAT
RITAESIESRVRELSAAAAASEAKQGFWEEFEMLQQQECRLLYPRKEGQRPENKPKNRYKNILPFDTTRVILRDVD
DSVPGADYINANYIRSDPEEKPGHERGKVYIATQGCLQTTVAAFWAMVYQENTHVIVMTTREVERGRNKCFRY
WPELHGSQEYGHVHVRNVAERQAQGYCVRELQVWQPDQEEPPRKVKHYQYFSWPDHGVPAEPAGVLGFLD
EVNRAQSSVPEAGPMVVHCSAGIGRTGTIIVIDILVGVVRRKGLDCDIDIPKTIQLVRRQRSGMVQTEAQYKFVYL
ALQRYIQGEQLRLREQVGPGRGRSGACAGVPVVTRGPCPQREPPEGRDYLNVRAAPAGPGRSRRPAP

>Tupaia_(*Tupaia_chinensis*)_SHP-2

Source: GenBank XP_006148032

MTSRRWFHPNITGVEAENLLLTRGVDGSFLARPSKSNPGDFTLSVRRNGAVTHIKIQNTGDYYDLYGGEKFATLAE
LVQYYMEHHGQLKEKNGDVIELKYPLNCADPTSERWFHGHLSGKEAEKLLTEKGKHGSFLVRESQSHPGDFVLSV
RTGDDKGESNDGKSKVTHVMIRCQELKYDVGGGERFDSLTDLVEHYKKNPMVETLGTVLQLKQPLNTTRINAAEI
ESRVRELSKLAETTDKVKQGFWEEFETLQQQECKLLYSRKEGQRQENKNKNRYKNILPFDHTRVVLHDGDPNEP
VSDYINANIIMPEFETKCNNSKPKKSYIATQGCLQNTVNDFWRMVFQENSRVIVMTTKEVERGKSKCVKYWPDE
YSLKEYGVMRVRNVKESAAHDYTLRELKLSKVGQGNTERTVWQYHFRTWPDHGVPSDPGGVLDFLEEVHHKQ
ESIMDAGPVVVHCSAGIGRTGTFIVIDILIDIIREKGVDCDIDVPKTIQMVRSQRSGMVQTEAQYRFIYMAVQHYIE
TLQRRIEEEQKSKRKGHEYTNIKYSLTDQTSGDQSPLPPCTPTPPCAEMREDNARVYENVGLMQQQKSFR

>Tupaia_(*Tupaia_chinensis*)_SHP-2L

Source: GenBank XP_014437880 (the reported region sequence of exons 5-to-7 has unclarities, and the actual sequence may encode a molecule more similar to SHP-2L in other mammals)

MTSRRWFHPNISGIEAEKLLLSRGQHGSFLARPSKSSPGGFTLSVRRHEEVTHIKIQNTGDYYDLYGGEKFATLAEL
VQHYTGQRGGLLRERSGAPVELRHPLGCQDPTSERWYHGHLSGKEAEKLLMEKGRLGSFLVRESQSKPGDFVLS
ALTQEPNKAQGTDHRPRPTHIMIRFQPDGKYDVGGGERFDTLRDLVEHYKKNPMVEKSGAVVHLKQPLKATRIN
ADSIESRVQELAQAADTSGKATQGFWEEFEVPSACCHPHTQTSGARAQEESERGRNRYKNILPFDTTRVVLHDV
DHGTPGADYINANYIRSDPEEKPGHGLGKVYIATQGCLQTTVAAFWAMVHQENTCVIVMTTKEVERGRNKCFR
YWPELYSSQEYGQVHVSNIAEHQAQGYCVRELQVWRPDQVEPPRTVKHYQYFSWPDHGVPAEHAGVLGFRRL
DEVNRTQSSVPGAGPMVVHCSAGIGRTGTIIVIDILVDVIRKQGLNCDIDIPKTIQMVRRQRSGMVQTEAQYKFV
YLALQRYIQSEQLRLREQREPPEERGEYLNVGPAPANPSSSPRLQQSRPAATQEISGGVYENLLTLRR

>Cattle_(*Bos_taurus*)_SHP-2

Source: GenBank XP_002694636

MTSRRWFHPNITGVEAENLLLTRGVDGSFLARPSKSNPGDFTLSVRRNGAVTHIKIQNTGDYYDLYGGEKFATLAE
LVQYYMEHHGQLKEKNGDVIELKYPLNCADPTSERWFHGHLSGKEAEKLLTEKGKHGSFLVRESQSHPGDFVLSV
RTGDDKGESNDAKSKVTHVMIRCQELKYDVGGGERFDSLTDLVEHYKKNPMVETLGTVLQLKQPLNTTRINAAEI
ESRVRELSKLAETTDKVKQGFWEEFETLQQQECKLLYSRKEGQRQENKNKNRYKNILPFDHTRVVLHDGDPNEP
VSDYINANIIMPEFETKCNNSKPKKSYIATQGCLQNTVNDFWRMVFQENSRVIVMTTKEVERGKSKCVKYWPDE
YALKEYGVMRVRNVKESAAHDYTLRELKLSKVGQGNTERTVWQYHFRTWPDHGVPSDPGGVLDFLEEVHHKQ
ESIVDAGPVVVHCSAGIGRTGTFIVIDILIDIIREKGVDCDIDVPKTIQMVRSQRSGMVQTEAQYRFIYMAVQHYIE
TLQRRIEEEQKSKRKGHEYTNIKYSLTDQASGDQSPLPPCTPTPSCAEMREDNARVYENVGLMQQQKSFR

>Cattle_(*Bos_taurus*)_SHP-2L

Source: GenBank XP_015330718 (supported by TSAs such as GGUZ01039644)

MTSRRWFHPNISGVEAEQLLMSSGQHGSFLARPSKSCPGGFTLSVRRHNEVTHIKIQNTGDYYDLYGGEKFATLA
ELVQHYTGQHGGLLRERGGAPVELRHPLGCQDPTSERWYHGHLSGKEAEKLLMEKGRPGSFLVRESQSKPGDFV
LSVLTQQLDRVDRQPRVTHIMIHFQPDGKYDVGGGEQFDTLGDLVERYRKNPMVERSGVVVHLRQPLKATRISA
ASIESRVQELSEATDASEKAKQGFWEEFEMLQQQECRLLYPRKEGQRPENKPKNRYKNILPFDTTRVILHDVDDR
VPGADYINANYIRSDPEEKPGHGLGKVYIATQGCLPTTVAAFWAMVHQENTHVIVMTTREVERGRNKCFRYWP
ELHGSQEYGHLHVCNMAEHWAQGYCVRELQVWRPDQEESPHTVKHYQYFSWPDHGVPAEPTGVLSFLEEVN
RTHSSMPGAGPMVVHCSAGIGRTGTIIVIDILVDVIRRQGLDCDIDVPKTIQLVRRQRSGMVQTEAQYKFVYLALQ
RYIQGEQLRLRKPVGAGPGRGGACAGVTMVTRGPSPQRERQEERGSLNLGVSPVDPGCSPGPAPSRAPAATRE
APRHEYENLQGLEP

>Platypus_(*Ornithorhynchus_anatinus*)_SHP-2

Source: GenBank XP_028907739

MTSRRWFHPNITGVEAENLLLTRGVDGSFLARPSKSNPGDFTLSVRRNGAVTHIKIQNTGDYYDLYGGEKFATLAE
LVQYYMEHHGQLKEKNGDVIELKYPLNCADPTSERWFHGHLSGKEAEKLLTEKGKHGSFLVRESQSHPGDFVLSV
RTGDDKGESNDGKSKVTHVMIRCQDLKYDVGGGEKFDSLTDLVEHYKKNPMVETLGTVLQLKQPLNTTRINAAE
IESRVRELSKLAETTDKVKQGFWEEFETLQQQECKLLYSRKEGQRQENKNKNRYKNILPFDHTRVVLHDGDPNEP
VSDYINANIIMPEFETKCNNTKPKKSYIATQGCLQNTVNDFWRMVFQENSRVIVMTTKEVERGKSKCVKYWPDE
YALKEYGVMRVRNVKESAAHDYTLRELKLSKVGQGNTERTVWQYHFRTWPDHGVPSDPGGVLDFLEEVHHKQ
ENITDAGPVVVHCSAGIGRTGTFIVIDILIDIIREKGVDCDIDVPKTIQMVRSQRSGMVQTEAQYRFIYMAVQHYIE
TLQRRIEEEQKSKRKGHEYTNIKYSLADQTSGDQXPLPPCTPTPTCPEMRDDSARVYENVGLMQQQKSFR

>Platypus_(*Ornithorhynchus_anatinus*)_SHP-2L

Source: GenBank XP_028922623

MTSRRWFHPNINGIEAEKLLLTRGGHGSFLARPSKSSLGDFTLSVRRNEEVTHIKIQNTGDYYDLYGGEKFATLAEL
VQYYTEQQGLLREKNGDVIELKFPLNCQDPTSERWYHGHLFGKEAEKLLTEKGKAGSFLVRESQSKPGDFVLSVLT
HEDRTDTGDRKARVTHVMIRFQADGKYDVGGGERFDTLTDLVEHYKKNPMVEKSGAVVHLKQPFNATRINAAN
IENRVKELNKTADQSEKAKQGFWEEFEMLQQQECKLLYPRKEGQRPENKAKNRYKNILPFDTTRVTLQEVDHRV
PGADYINANYIQNIGEDGQSSEQDKVYIATQGCLQTTVNDFWAMVYQENCHVIVMTTKEMERGRNKCFRYWP
DRDSSKEFGCICVRNTEEREAQGYLLRELEIRRTDREEQPRKVKHYQYFSWPDHGVPTEPGGVLSFLDQVNRAQR
GYVNTGPIIVHCSAGIGRTGTIIVIDILVDVIHRQGLDCDIDIPKTIQMVRRQRSGMVQTEAQYKFVYMALQQFIET
ERKRLEEEQRSKRKEREYLNIRYQPLDRARGKGNLPPARAQAGMDDDPASVYENLSLQGTKVFGAGNTGR

>Chicken_(*Gallus_gallus*)_SHP-2

Source: GenBank NP_990299

MTSRRWFHPNITGVEAENLLLTRGVDGSFLARPSKSNPGDFTLSVRRTGAVTHIKIQNTGDYYDLYGGEKFATLAE
LVQYYMEHHGQLKEKNGDVIELKYPLNCADPTSERWFHGHLSGREAEKLLTEKGKHGSFLVRESQSHPGDFVLSV
RTGDDKGESNDGKSKVTHVMIHCQDLKYDVGGGEKFDSLTDLVEHYKKNPMVETLGTVLQLKQPLNTTRINAAE
IESRVRELSKLAETTDKVKQGFWEEFETLQQQECKLLYSRKEGQRQENKNKNRYKNILPFDHTRVVLHDGDPNEP
VSDYINANIIMPEFETKCNNSKPKKSYIATQGCLQNTVNDFWRMVFQENSRVIVMTTKEVERGKSKCVKYWPDE
YSLKEYGVMRVRNVKESAAHDYTLRELKLSKVGQGNTERTVWQYHFRTWPDHGVPSDPGGVLDFLEEVHHKQ
ESISDAGPVVVHCSAGIGRTGTFIVIDILIDIIREKGVDCDIDVPKTIQMVRSQRSGMVQTEAQYRFIYMAVQHYIET
LQRRIEEEQKSKRKGHEYTNIKYSLSDQTSGDQSPLPPCTPTPTCPEMREDSARVYENVGLMQQQKSFR

>Chicken_(*Gallus_gallus*)_SHP-2L

Source: GenBank NP_001026046

MVRWFHPNISGIEAEKLLLTRGVHGSFLARPSKSNPGDFTLSVRRNDEVTHIKIQNTGDYYDLYGGEKFATLAELV
QYYTEQQGLLREKNSNVIELKYPLNCQDPTSERWYHGHLTGKEAEKLLTEKGKPGSFLVRESQSKPGDFVLSVLTN
EDKMETGDRKPHVTHVMIHYQPDGKYDVGGGERFDTLTDLVEHYKKNPMVEKSGAVVHLKQPFNATRINAANI
ENRVRELNKMADHSEKAKQGFWEEFEMLQQQECKLLYPRKEGQRPENKAKNRYKNILPFDTTRVALRDVDESV
PGSDYINANYIKSIPEDGRNSEHCKIYIATQGCLQTTVNDFWTMVYQENTHVIVMTTKEVERGRNKCFRYWPDK
GCAKEYGCICVRNISEREAQGYYLRELEILHTDRDERPRVVKHYQYFSWPDHGVPNEPGGVLSFLDQVNRTQRSI
PDTGPIIVHCSAGIGRTGTIIVIDILVDIIHRQGLDCDIDIPKTIQMVRRQRSGMVQTEAQYKFVYMAVQQFIEAEQ
KRLEEEQRSKRKERDYLNIGYSPVEKGRAKGQPPSPRAQPVADDESASVYENLNIKSPKVSGMSNTGR

>Swan_goose_(*Anser_cygnoides_domesticus*)_SHP-2

Source: GenBank XP_047929871 modified based on NW_025927732 (we could not predict the N-terminus with certainty)

WFHPNITGVEAENLLLTRGVDGSFLARPSKSNPGDFTLSVRRTGAVTHIKIQNTGDYYDLYGGEKFATLAELVQYY
MEHHGQLKEKNGDVIELKYPLNCADPTSERWFHGHLSGREAEKLLTEKGKHGSFLVRESQSHPGDFVLSVRTGD
DKGESNDGKSKVTHVMIHCQDLKYDVGGGEKFDSLTDLVEHYKKNPMVETLGTVLQLKQPLNTTRINAAEIESRV
RELSKLAETTDKVKQGFWEEFETLQQQECKLLYSRKEGQRQENKNKNRYKNILPFDHTRVVLHDGDPNEPVSDYI
NANIIMPEFETKCNNSKPKKSYIATQGCLQNTVNDFWRMVFQENSRVIVMTTKEVERGKSKCVKYWPDEYSLKE
YGVMRVRNVKESAAHDYTLRELKLSKVGQGNTERTVWQYHFRTWPDHGVPSDPGGVLDFLEEVHHKQESISD
AGPVVVHCSAGIGRTGTFIVIDILIDIIREKGVDCDIDVPKTIQMVRSQRSGMVQTEAQYRFIYMAVQHYIETLQRR
IEEEQKSKRKGHEYTNIKYSLSDQTSGDQSPLPPCTPTPTCPEMREDSARVYENVGLMQQQKSFR

>Swan_goose_(*Anser_cygnoides_domesticus*)_SHP-2L

Source: GenBank XP_047923286

MVRWFHPNISGIEAEKLLLTRGVHGSFLARPSKSNPGDFTLSVRRNDEVTHIKIQNTGDYYDLYGGEKFATLAELV
QYYTEQQGLLREKNSNVIELKYPLNCQDPTSERWYHGHLTGKEAEKLLTEKGKPGSFLVRESQSKPGDFVLSVLTN
EDKMETGDRKPHVTHVMIHYQPDGKYDVGGGERFDTLTDLVEHYKKNPMVEKSGAVVHLKQPFNATRINAANI
ENRVKELNKMADHSEKAKQGFWEEFEMLQQQECKLLYPRKEGQRPENKAKNRYKNILPFDTTRVALRDVDESV
PGSDYINANYIKSIPEDGRNSEHCKIYIATQGCLQTTVNDFWTMVYQENSHVIVMTTKEVERGRNKCFRYWPDK
GCAKEYGCICVRNVSEREAQGYYLRELEIVRTDRDERPRVVKHYQYFSWPDHGVPNEPGGVLSFLDQVNRAQRS
IPDTGPIIVHCSAGIGRTGTIIVIDILVDIIHRQGLDCDIDIPKTIQMVRRQRSGMVQTEAQYKFVYMAVQQFIEAEQ
KRLEEEQRNKRKERDYLNIGYSPMEKGRAKGQPPSPRAQSVVDDESASVYENLNIKSPKVSGMSNTGR

>Green_anole_lizard_(*Anolis_carolinensis*)_SHP-2

Source: GenBank XP_062814411

MTSRRWFHPNITGVEAENLLLTRGVDGSFLARPSKSNPGDFTLSVRRNGAVTHIKIQNTGDYYDLYGGEKFATLAE
LVQYYMEHHGQLKEKNGDVIELKYPLNCADPTSERWFHGHLSGKEAEKLLTEKGKHGSFLVRESQSHPGDFVLSV
RTGDDKGESNDGKSKVTHVMIRCQDMKYDVGGGEKFDSLTDLVEHYKKNPMVETLGTVLQLKQPLNTTRINAA
EIESRVRELSKLAETTDKVKQGFWEEFETLQQQECKLLYSRKEGQRQENKNKNRYKNILPFDHTRVVLHDGDPNE
PVSDYINANIIMPEFETKCNNTKPKKSYIATQGCLQNTVNDFWRMVFQENSRVIVMTTKEVERGKSKCVKYWPD
EHALKEYGVMRVRNVKESAAHDYTLRELKLSKVGQGNTERTVWQYHFRTWPDHGVPSDPGGVLDFLEEVHHK
QEGIADAGPVVVHCSAGIGRTGTFIVIDILIDIIREKGVDCDIDVPKTIQMVRSQRSGMVQTEAQYRFIYMAVQHYI
ETLQRRIEEEQKSKRKGHEYTNIKYSLADQAGGDQSPLPPCTPTPTCPEMREDNAMRVYENVGLMQQQKSFR

>Green_anole_lizard_(*Anolis_carolinensis*)_SHP-2L

Source: GenBank XP_062821653

MRWFHPNINGIEAEKLLLTRGVHGSFLARPSKSNPGDFTLSVRRNDEVTHIKIQNTGDYYDLYGGEKFATLSELVQ
YYTEQQGLLREKNSNIIELKFPLNCQDPTSERWYHGHLTGKEAEKLLTEKGKPGSFLVRSSQSKPGDFVLSVLTNED
KADTGDRKPRVTHIMIRYQPDGKYDVGGGERFDTLTDLVEHYKKNPMVEKSGAVVHLKQPFNATRIIAANIENRV
KELNKMADHSEKAKQGFWEEFEMLQQQECKFLYPRKEGQRMENKAKNRYKNILPFDTTRVALREVDESLPGSD
YINANYIRNIAEDGQSSEHSKVYIATQGCLQTTVNDFWAMVYQENTHVIVMTTKEVERGRNKCFHYWPDKNCT
KDYGYISVRNVSELEAQGYHIRELEIARLDREEQPRLIWHFQYISWPDHGVPNEPGGVLSFLDQVNKAQRSVPGT
GPIVVHCSAGIGRTGTIIVIDILVDTIHRQGLDCDIDIPKTIQMVRRQRSGMVQTEAQYKFVYMAVQHYIATEQKEL
EEEQRSMRKEKEYLNIRYPPMEQPRGKARPPSPRVQMGVDDESAAVYENLNVKAAKVSGITHSGR

>Green_sea_turtle_(Chelonia_mydas)_SHP-2

Source: GenBank XP_037735106

MTSRRWFHPNITGVEAENLLLTRGVDGSFLARPSKSNPGDFTLSVRRNGAVTHIKIQNTGDYYDLYGGEKFATLAE
LVQYYMEHHGQLKEKNGDVIELKYPLNCADPTSERWFHGHLSGKEAEKLLTEKGKHGSFLVRESQSHPGDFVLSV

RTGDDKGESNDGKSKVTHVMIRCQELKYDVGGGEKFDSLTDLVEHYKKNPMVETLGTVLQLKQPLNTTRINAAEI
ESRVRELSKLAETTDKVKQGFWEEFETLQQQECKLLYSRKEGQRQESKNKNRYKNILPFDHTRVVLHDGDPNEPV
SDYINANIIMPEFESKCNNSKPKKSYIATQGCLQNTVNDFWRMVFQENSRVIVMTTKEVERGKSKCVKYWPDEY
ALKEYGVMRVRNVKESAAHDYTLRELKLSKVGQGNTERTVWQYHFRTWPDHGVPSDPGGVLDFLEEVHHKQE
SIADSGPVVVHCSAGIGRTGTFIVIDILIDIIREKGVDCDIDVPKTIQMVRSQRSGMVQTEAQYRFIYMAVQHYIETL
QRRIEEEQKSKRKGHEYTNIKYSLSDQTSGDQSPLPPCTPTPTCPEMREDSARVYENVGLMQQQQKSFR

>Green_sea_turtle_(Chelonia_mydas)_SHP-2L

Source: GenBank XP_027688190

MVRWFHPNINGIEAEKLLLTRGVHGSFLARPSKSNPGDFTLSVRRNDEVTHIKIQNTGDYYDLYGGEKFATLAELV
QYYTEQQGLLREKNSNVIELKYPLNCQDPTSERWYHGHLTGKEAEKLLTEKGKPGSFLVRESQSKPGDFVLSVLTN
EDKMETGDRKPRVTHVMIRYQPDGKYDVGGGERFDTLTDLVEHYKKNPMVEKSGAVVHLKQPFNATRINAANI
ENRVKELNKMADHSEKAKQGFWEEFEMLQQQECKLLYPRKEGQRVENKAKNRYKNILPFDTTRVALRDVDESLP
GSDYINANYIKSIPEDGRSSEHCKVYIATQGCLQTTVNDFWAMVYQENTHVIVMTTKEVERGRNKCFRYWPDKN
CTKEYGHICVRIVSEREAQGYYLRELEITRTDREEHPRQVKHYQYFSWPDHGVPNEPGGVLSFLDQVNRAQRSIP
DSGPIVVHCSAGIGRTGTIIVIDILVDTIHRQGLDCDIDIPKTIQMVRRQRSGMVQTEAQYKFVYMAVQQYIETEQ
KRLEEEQRNKRKERDYLNIRHPPMDKARAKGQPPPPRVSVVDDDDSATVYENLNIKSSKVSGMNNAGR

>Tropical_clawed_frog_(*Xenopus_tropicalis*)_SHP-2

Source: GenBank XP_012818337

MTSRRWFHPNITGVEAENLLLTRGVDGSFLARPSKSNPGDFTLSVRRNGAVTHIKIQNTGDYYDLYGGEKFATLAE
LVQYYMEHHGQLKEKNGDVIELKYPLNCADPTSERWFHGHLSGKEAEKLLTEKGKHGSFLVRESQSHPGDFVLSV
RTGDDKAESNDGKSKVTHVMIRCQDLKYDVGGGEKFDSLTDLVEHYKKNPMVETLGTVLQLKQPLNTTRINAAE
IESRVKELSKPAETADKFKQGFWEEFETLQQQECKLLYSRKEGQRQENKNKNRYKNILPFDHTRVELHDGDPNEQ
VSDYINANIIMPEFETKCNNPKPKKRYIATQGCLQNTVNDFWRMVFQENSRVIVMTTKEVERGKSKCVKYWPDE
FALKEYGVMRVRNVKETPAHDYILRELKLSKVGQGNTERTVWQYHFKTWPDHGVPTDPGGVLDFLEEVHHKQD
GIADAGPVVVHCSAGIGRTGTFIVIDILIDVIREKGVDCDIDVPKTIQMVRSQRSGMVQTEAQYRFIYMAVQHYIE
TLQRRIEEEQKSKRKGHEYTNIKYSLSEQAGDQSPLPPCTPTPLPTPTLPEMREDAGRVYENVGLMQQQKSFR

>Tropical_clawed_frog_(*Xenopus_tropicalis*)_SHP-2L

Source: GenBank NP_001015717

MTSRRWFHPNISGQEAETLLLTNGGHGSFLARPSKSNPGDFTLSVRRHNEVTHIKIQNTGDYYDLYGGETFATLAE
LVQYYTEQQGLLREKNGDVIELKYPLNSQDPTSERWYHGHLSGKEAEKLLLDKGKPGSFLVRESQSKPGDFVLSVL
TSEEKLENGERKSRVTHVMIRFQPDGKYDVGGGERFDTLTDLLDHYKKNPMVEKSGAVVHLKQPFNATRINAAN
IETRVRELNKTADNTEKAKQGFWEEFEMLQQQECKLLYPRKEGQRPENKSKNRYKNILPFDTTRVTLRDTDESVA

GSDYINANYIKSTLDEARGSQHYKVYIATQGCLQNTVCDFWAMVYQENTHVIVMTTKEVERSRKKCFRYWPDE
NCTKDYGCIRVQSLEVQQKKDYILRELQISRMDKVESPRPIWHYQYLSWPDHGVPIEPGGVLSFLEEINAAQKYIP
HSGPIVVHCSAGIGRTGTIIVIDMLVDIINRQGLDCDIDIPKTIQMVRQQRSGMVQTEAQYKFIYMAVQKYIEVRK
RHLEEEQMGKLKEREYTNIQYSVGERARTKAQPAAAQSQHRVSDDAACVYENLNIRSTHMSSTSNAGR

Lungfishes (Class: Sarcopterygii [lobe-finned fishes plus tetrapods]; Subclass: Dipnoi)

>West_African_Lungfish_(*Protopterus_annectens*)_SHP-2

Source: GenBank GGXP01000072 (a TSA)

MTSRRWFHPNITGVEAENLLLTRGVDGSFLARPSKSNPGDFTLSVRRNAAVTHIKIQNTGDYYDLYGGEKFATLAE
LVQFYMEHHGQLKEKNGDVIELKYPLNCADPTSERWFHGHLSGKEAEKLLSEKGKNGSFLVRESQSHPGDFVLSV
RTGDDKGECYDGKPKVTHVMIRCQDMKYDVGGGEKFDSLTDLVEHYKKNPMVEKLGTVLQLKQPLNTTRINAA
EIESRVKELSKLAETTDKVKQGFWEEFETLQQQECKLLYSRKEGQRPENKNKNRYKNILPFDHTRVVLHDGDPNE
PVSDYINANIITPESETRSNNSKSKKCYIATQGCLQNTVNDFWRMVFQENSMVIVMTTKEVERGKSKCVKYWPD
EYQLKEYGVMRVRNVRETPAHDYILRELKLSKVGQGNTERTVWQYHFKTWPDHGVPADPGGVLDFLEEVHLKQ
ENISEVGPIVVHCSAGIGRTGTFIVIDILIDIIREKGVDCDIDVPKTIQMVRSQRSGMVQTEAQYRFIYMAVQHYIET
LQRRIEEEQKTKMKGREYTNIKYSLSDLTGDLSPLPPGTPTSACTEMRDDSARVYENVGVMQQQKSFR

>West_African_Lungfish_(*Protopterus_annectens*)_SHP-2L

(information for the N-terminus is lacking)

Source: GenBank XP_043919015 (modified)

WFHPNITGVEAEQVLLTRGVHGSFLARPSKSNPGDFTLSVRRNDEVTHIKIQNTGDFYDLYGGEKFATLAELVQYY
TEQQGLLREKNGDVIELKYPLNCKDPTSERWYHGHLSGKEAEKLLIDKGKPGSFLVRESQSKPGDFVLSVLTSEEKL
ENGDRRPKVTHVMIRFQDGKYDVGGGERFDTLTDLVEHYKKNPMVEISGIVVHLKQPFNATRINAANIENRVKEL
NKTAEHSEKAKQGFWEEFEVLQQQECKFLYPRKEGQRLENKSKNRYKNILPFDTTRVKLTELDETIMNYDYINANY
IRNIPEDGKSAEECKSYIATQGCLQNTVNDFWRMVYQENTHVIVMTTKEVERGRNKCVRYWPEENSFKEYGNICI
RNVEEHTAQDYVLRILELTRLDRHEVPRQIWQYQYLSWPDHGVPNEPGGVLSFLDQVNQEQQSIPHAGPIIVHC
SAGIGRTGTIIVIDILIDIINRQGLDCDIDIPKTIQMVRRQRSGMVQTEAQYKFIYMAVQQYIETVQKRLEEEQRNKT
KEREYSNIRYPPMEKPKSRPQTPSARTSPMVNEDPGCVYENLNIKNPKVVGISSARR

Sharks and Rays (Class: Chondrichthyes [cartilaginous fish]; Subclass: Elasmobranchii)

>Small-spotted_catshark_(*Scyliorhinus_canicula*)_SHP-2

Source: GenBank XP_038675925 (modified)

MTSRRWFHPNITGVEAENLLLTRGVDGSFLARPSKSNPGDFTLSVRRNGAVTHIKIQNTGDYYDLYGGE
KFATLAELVQYYMEHHGQLKEKNGDVIELKYPLNCADPTSERWFHGHLSGKEAEKLLTEKGKPGSFLVR
ESQSHPGDFVLSVRTGDDKGESSEGKPKVTHVMIRCQDGKYDVGGGEKFDSLTDLVEHYKKNPMVET
LGTVLQLKQPLNTTRINAAEIESRVRELSKPAEMMDKVKQGFWEEFETLQQQECKLLYSRKEGQRPENK
NKNRYKNILPFDHTRVVLHDGDVNESGSDYINANFITHDIESNRTTTKPKKSYIATQGCLQNTVNDFWR
MVFQENSSVIVMTTKEVERGKSKCVKYWPDEMSLKEYGGMRVRNVRESPAHDYILRELKLSKVGQGN
TERTVWQYHFKTWPDHGVPSDPGGVLDFLEEVNIKQENIPETGPIVVHCSAGIGRTGTFIVIDILIDIIRE
KGVDCDIDVPKSIQMVRSQRSGMVQTEAQYRFIYMAVQHYIETLQRRIEEEQKSKIKGREYTNIKYSLSD
LTCGGDQSPFPPCTPNPACTELKDEGGRIYENVGLMQAQKGHR

>Small-spotted_catshark_(*Scyliorhinus_canicula*)_SHP-2L

Source: GenBank XP_038677677

MTSRRWFHPNITGIEAEQLLLTRGVHGNFLARPSKSNPGDFTLSVRRNEEVTHIKIQNTGDYYDLYGGEKFATLAE
LVQYYTEQQGLLREKNGDIIELKYPLNCQDPTSERWYHGHLSGKDAEKLLTDKGKPGSFLVRESLSKPGDFVLSVLT
NEEKVENGDRKPRVTHVMIRYQDGKYDVGGGERFDTLTDLVEHYKKNPMVEVSGIVVHLKQPFNATRINAANIE
NRVKELNKTADNTEKPKQGFWEEFELLQQQEYKLLYSRKEGQRMENKSKNRYKNILPFDNTRVTLKEVDETILGS
DYINANNITMKLKNGKHGDESKTYIATQGCLQNTINDFWKMIYQENTHVIVMTTKEVERGRNKCFRYWPDLEA
SKQYGTISVRNLKERMAQDYIVRELEVTQTAQNEPPRHIWHYQYLSWPDHGVPNEPGGVLSFLDQVNRAQQSIP
DTGPIIVHCSAGIGRTGTIIVIDMLVADINRQGLDCDIDIPKTIQMVRKQRSGMVQTEAQYKFIYMAVQQYIETVQ
KRLLEEQKNKTTEREYSNIHYLRMEKAKTKRHAAPPRVFPALKEEPSCLYENLNIKSSKSSGGCNTKR

>Thorny_skate_(*Amblyraja_radiata*)_SHP-2

Source: GenBank XP_032899632

MTSRRWFHPNITGVEAENLLLTRGVDGSFLARPSKSNPGDFTLSVRRNGAVTHIKIQNTGDYYDLYGGEKFATLAE
LVQYYMEHHGQLKEKNGDVIELKYPLNCADPTSERWFHGHLSGKEAEKLLTEKGKPGSFLVRESQSHPGDFVLSV
RTGDDKGESSEGKSKVTHVMIRCQDGKYDVGGGEKFDSLTDLVEHYKKNPMVETLGTVLQLKQPLNTTRINAAEI
ESRVRELSKPAEMMDKVKQGFWEEFETLQQQECKLLYSRKDGQRQENKNKNRYKNILPFDHTRVVLHDGDLTE
AGSDYINANFITHDIDSNRTTTRPKKSYIATQGCLQNTVNDFWRMVFQENSRVIVMTTKEVERGKSKCVKYWPD
EMSLKEYGAMRVRNVRENPAHDYILRELKLSQVGQSTERTVWQYHFKTWPDHGVPSDPGGVLDFLEEINIKQE
NIPETGPSVVHCSAGIGRTGTFIVIDILIDIIREKGVDCDIDVPKSIQMVRSQRSGMVQTEAQYRFIYMAVQHYIETL
QRRIEEEQKSKIKGREYTNIKYSLSDLSCGGDQSPFPPCTPNPVCTEMKDEGGRIYENVGLMQQQKGHR

>Thorny_skate_(*Amblyraja_radiata*)_SHP-2L

Source: GenBank XP_032903735

MTSRRWFHPNITGIEAEQVLLCRGVHGSFLARPSKSNPGDFTLSIRRNVEVTHIKIQNSGDYYDLYGGEKFATLAEL
VQHYTEQEGLLREKNGDVIELKYPLNCRDPTSERWYHGHLSGKDAEKLLTDKGKPGSFLVRESLSKPGDFVLSVLT
NEEKVENGDRKPRVTHVMIGYQAGKYDVGGGERFDTLTDLVEHYKKNPMVEVSGIVVHLKQPFNATRINAANIE
NRVKELNKTADNTEKAKQGFWEEFELLQQQEHKLLYSRKEGQRIENKNKNRYKNILPFDNTRVSLKEVDESVVGA
DYINANDITNGKSGDDCKNYIATQGCLQNTINDFWKIIYQENAHVIVMTTKEIERGKNKCVRYWPDLETSKEYGLI
SVRNLEERMAQDYIVRDLEVTRIDQNDLPRHIWHYQYLSWPDHGVPNEPGGVLSFLDQVNRAQQSIPDMGPII
VHCSAGIGRTGTIIVIDMLISDISRQGLDCDIDIPKTIQIVRKQRSGMVQTEAQYKFIYMAVQQYIETVQRRLQEEQ
KNKTKDREYSNIRYLPTEVKTKRQAGTSRDFSLSKEESSCLYENLNIKTSKASGGSNAKR

Bichirs and Reedfish (Class: Actinopterygii [ray-finned fish]; Order: Polypteriformes)

>Lapradei_bichir_(*Polypterus_bichir_lapradei*)_SHP-2

Source: GenBank GKOV011119657 (a TSA; for *Polypterus senegalus* see NW_024382475 and NC_053165)

MTSRRWFHPNITGVEAENLLLTRGVDGSFLARPSKSNPGDFTLSVRRNGAVTHIKIQNTGDYYDLYGGEKFATLAE
LVQYYMEHHGQLKEKNGDVIELKYPLNCADPTSERWFHGHLSGREAEKLLTEKGKNGSFLVRESQSHPGDFVLSV
RTGDDKGESSDGKSKVTHVMIRCQDLKYDVGGGEKFDSLTDLVEHYKKNPMVETLGTVLQLKQPLNTTRINAAEI
ESRVRELSKLAEATDKFKQGFWEEFETLQQQECKLLYSRKEGQRPENKNKNRYKNILPFDHTRVVLNDGDPNEPG
SDYINANKIMPEFDSKCNNTKLKKCYIATQGCLQNTISDFWRMVFQENSRVIVMTTKEVERGKSKCLRYWPDVS
ALKEYGAMRVRNVKETLAHDYILRELKLSKVGQGNTERTVWQYHFRTWPDHGVPGDPGGVLDFLEEVNHKQE
SIFEAGPIVVHCSAGIGRTGTFIVIDILIDIIREKGVDCDIDVPKTIQMVRSQRSGMVQTEAQYRFIYMAVQHYIETL
QRRILEEQKSKIKGREYTNIKYSLSDMTAGDQSPIPPCTPTPSCTDMRDDNSRVYENVGLMAQQKR

>Gray_bichir_(*Polypterus_senegalus*)_SHP-2L

Source: GenBank XP_039611514

MTSRRWFHPNISGIEAEELLLTRGVHGSFLARPSKSNPGDFTLSVRRGDEVTHIKIQNSGDYYDLYGGEKFATLAEL
VQYYTEQQDLLREKNGDVIELKYPLNCKDPTSERWYHGHLSGKDAEKLLMEKGKAGSFLVRESQSKPGDFVLSVL
TNEEKHENVERKTKVTHVMIRYQNGKYDVGGGEQFDTLTDLVEHYKKNPMVEKSGIVVHLKQPFNTTRINAANI
ENRVKELNKVADNMEKPKQGFWEEFEMLQQQECKLLYPRKEGQRPENKSKNRYKNILPFDTTRVELKETDPEILG
SDYINANYIQSVQEDSRQLCKGKVFIATQGCLQNTVKDFWKMVYQENTHVIVMTTKEVERGRNKCVRYWPDM

EATKEYGSLQVRNIEERTAQDYVLRKLEVSCLNRDEPHRYIWHYQYLSWPDHGVPNEPGGVLSFLEQVNRTQQSI
PDTGPIVVHCSAGIGRTGTIIVIDILIDIINRQGLDCDIDIPKTIQMVRRQRSGMVQTEAQYKFIYMAVQQYIDTAQK
RLEEEQRNKIKEREYSNIRYPPMENTKLRPQTPTPRGTSALEDDSTGVYENLNLKNPKVPGISNTRR

>Reedfish_(*Erpetoichthys_calabaricus*)_SHP-2

Source: GenBank XP_028680134

MTSRRWFHPNITGVEAENLLLTRGVDGSFLARPSKSNPGDFTLSVRRNGAVTHIKIQNTGDYYDLYGGEKFATLAE
LVQYYMEHHGQLKEKNGDVIELKYPLNCADPTSERWFHGHLSGREAEKLLTEKGKNGSFLVRESQSHPGDFVLSV
RTGDDKGESSDGKSKVTHVMIRCQQDLKYDVGGGEKFDSLTDLVEHYKKNPMVETLGTVLQLKQPLNTTRINAA
EIESRVRELSKLAEATDKFKQGFWEEFETLQQQECKLLYSRKEGQRPENKNKNRYKNILPFDHTRVVLNDGDPNEP
GSDYINANKIMPEFDSKCNNTRLKKSYIATQGCLQNTISDFWRMVFQENSRVIVMTTKEVERGKSKCLRYWPDV
SALKEYGAMRVRNVKETLAHDYILRELKLSKVGQGNTERTVWQYHFRTWPDHGVPGDPGGVLDFLEEVNHKQ
ESILEAGPIVVHCSAGIGRTGTFIVIDILIDIIREKGVDCDIDVPKTIQMVRSQRSGMVQTEAQYRFIYMAVQHYIETL
QRRILEEQKSKIKGREYTNIKYSLSDMTAGDQSPIPPCTPTPSCTDIRDDNSRVYENVGLMAQQKR

>Reedfish_(*Erpetoichthys_calabaricus*)_SHP-2L

Source: GenBank XP_028663680

MTSRRWFHPNISGIDAEELLLTRGVHGSFLARPSKSNPGDFTLSVRRSDEVTHIKIQNSGDYYDLYGGEKFATLAEL
VQYYTEQQDLLREKNGDVIELKYPLNCKDPTSERWYHGHLSGKDAEKLLMEKGKAGSFLVRESQSKPGDFVLSVL
TNEEKHENMERKTKVTHVMIRYQNGKYDVGGGEQFDTLTDLVEHYKKNPMVEKSGIVVHLKQPFNTTRINAAN
IENRVKELNKVADNMEKPKQGFWEEFEMLQQQECKLLYPRKEGQRPENKSKNRYKNILPFDTTRVELKETDPEIL
GSDYINANYIQSVQEDSRQLCKGKVFIATQGCLQNTVKDFWKMVYQENTHVIVMTTKEVERGRNKCVRYWPDL
ETTKEYGSLQVRNIEERTAQDYVLRKLEVSCLNGDEPHRYIWHYQYLSWPDHGVPNEPGGVLSFLEQVNRTQQSI
PDTGPIVVHCSAGIGRTGTIIVIDILIDIINRQGLDCDIDIPKTIQMVRRQRSGMVQTEAQYKFIYMAVQQYIDTAQK
RLEEEQRNKIKEREYSNIRYPPMENTKLRPQTPTPRGTSALEDDSTGVYENLNLKNPKVPGISNTRR

Sturgeons and Paddlefishes (Class: Actinopterygii [ray-finned fishes]; Order: Acipenseriformes)

(in sterlet and paddlefish, there are even two more SHP-2 genes, but only two are shown here)

>Sterlet_sturgeon_(*Acipenser_ruthenus*)_SHP-2

Source: GenBank XP_033902829

MTSRRWFHPNITGVEAENLLLTRGVDGSFLARPSKSNPGDFTLSVRRNGAVTHIKIQNTGDYYDLYGGEKFATLAE
LVQYYMEHHGQLKEKNGDVIELKYPLNCADPTSERWFHGHLSGREAEKLLTEKGKNGSFLVRESQSHPGDFVLSV
RTGDDKGESSDGKPRVTHVMIRCQPDLKFDVGGGEKFDSLTDLVEHYKKNPMVETLGTVLQLKQPLNTTRINAA
EIESRVRELSKLAETTDKVKQGFWEEFETLQQQECKLLYSRKEGQRAENKNKNRYKNILPFDHTRVMLNDKEINEL
GSDYINANIIMPEVEVKCNNTKPKKSYIATQGCLQNTISDFWRMVFQENSRVIVMTTKEVERGKSKCVKYWPDV
TVLKEYGAMRVRNARETSAHDYILRELRMSKVGQGPDDERTVWQYHFRAWPDHGVPGDPGGVLDFLEEVNM
KQESILEAGPIVVHCSAGIGRTGTFIVIDMLIDVIREKGVDCDIDVPKSIQMVRSQRSGMVQTEAQYRFIYMAVQH
YIETLQRRIEEEQKSKIKGREYTNIKYSLTDLSGGDQSPLPPCTPTPVYADVRDDSSRVYENVGLMQQQKSFR

>Sterlet_sturgeon_(*Acipenser_ruthenus*)_SHP-2L

Source: GenBank XP_033899126

MTSRRWFHPNITGIEAEQLLLTRGVHGSFLARPSKSNPGDFTLSVRRNDEVTHIKIQNSGDYYDLYGGEKFATLAEL
VQYYTEQQDLLREKNGDVIELKYPLNCKDPTSERWYHGHLSGKDAEKLLTEKGKPGSFLVRESQSKPGDFVLSVLT
NEEKHENVDRKTKVTHVMIRYQDGKYDVGGGERFDTLTDLVEHYKKNPMVEKSGIVVHLKQPFNATRINAANIE
NRVKELNKVADHTEKPKQGFWEEFEMLQQQECKLLYPRKEGQKPENKTKNRYKNILPFDTTRVELRDADADVPG
AEYINANYIRSVHEDGRRPDECKVYIATQGCLQNTVNDFWNMVYQENTHVIVMTTKEVERGRNKCVRYWPDV
DSQKEYGLLCVRNMEERPAQDYVLRELEITRLDREEPPRYIWHYQYLSWPDHGVPNEPGGVLSFLDQVNQAQQ
SIPDTGPIVVHCSAGIGRTGTIIVIDILVDIISRQGLDCDIDVPKTIQMVRRQRSGMVQTEAQYKFIYMAVQQYIDTV
QKRLEEEQRNKTKEREYSNIRYPPTENTKLWPHTPTSRTSSMVNEDAAGVYENLNIKNPKGPGISNTRR

> Paddlefish_(*Polyodon_spathula*)_SHP-2

Source: GenBank XP_041083297

MTSRRWFHPNITGVEAENLLLTRGVDGSFLARPSKSNPGDFTLSVRRNGAVTHIKIQNTGDYYDLYGGEKFATLAE
LVQYYMEHHGQLKEKNGDVIELKYPLNCADPTSERWFHGHLSGREAEKLLTEKGKNGSFLVRESQSHPGDFVLSV
RTGDDKGESSDGKPRVTHVMIRCQPDLKFDVGGGEKFDSLTDLVEHYKKNPMVETLGTVLQLKQPLNTTRINAA
EIESRVRELSKLAETTDKVKQGFWEEFETLQQQECKLLYSRKEGQRAENKNKNRYKNILPFDHTRVMLNDKEINEL
GSDYINANIIMPEVEVKCNNTKPKKSYIATQGCLQNTISDFWRMVFQENSRVIVMTTKEVERGKSKCVKYWPDV
TVLKEYGAMRVRNARETSAHDYILRELRMSKVGQGPDDERTVWQYHFRAWPDHGVPGDPGGVLDFLEEVNM
KQESILEAGPIVVHCSAGIGRTGTFIVIDMLIDVIREKGVDCDIDVPKSIQMVRSQRSGMVQTEAQYRFIYMAVQH
YIETLQRRIEEEQKSKIKGREYTNIKYSLTDLSGGDQSPLPPCTPTPVYADVRDDSSRVYENVGLMQQQKSFR

> Paddlefish_(*Polyodon_spathula*)_SHP-2L

Source: GenBank XP_041130142

MTSRRWFHPNITGIEAEQLLLTRGVHGSFLARPSKSNPGDFTLSVRRNDEVTHIKIQNSGDYYDLYGGEKFATLAEL
VQYYTEQQDLLREKNGDVIELKYPLNCKDPTSERWYHGHLSGKDAEKLLTEKGKPGSFLVRESQSKPGDFVLSVLT
NEEKHENVDRRTKVTHVMIRYQDGKYDVGGGERFDTLTDLVEHYKKNPMVEKSGIVVHLKQPFNATRINAANIE
NRVRELNKVADHTEKPKQGFWEEFEMLQQQECKLLYPRKEGQKPENKSKNRYKNILPFDTTRVELRDADADIPG
AEYINANYIRSVHEDGRRPDECKVYIATQGCLQNTVNDFWNMVYQENTHVIVMTTKEVERGRNKCVRYWPDV
DSRKEYGLLCVRNMEERPAQDYVLRELEITRLDREEPPRYIWHYQYLSWPDHGVPNEPGGVLSFLDQVNQAQKS
IPDTGPIVVHCSAGIGRTGTIIVIDILIDIIGRQGLDCDIDVPKTIQMVRRQRSGMVQTEAQYKFIYMAVQQYIDTV
QKRLEEEQRNKTKEREYSNIRYPPTENTKLWPHTPTSRTSSMVNEDTAGVYENLNIKNPKCPGISNTRR

Gars (Class: Actinopterygii [ray-finned fishes]; Order: Lepisosteiformes)

>Spotted_gar_(*Lepisosteus_oculatus*)_SHP-2

Source: GenBank XP_015221631

MTSRRWFHPNITGVEAENLLLTRGVDGSFLARPSKSNPGDFTLSVRRNGAVTHIKIQNTGDYYDLYGGEKFATLAE
LVQYYMEHHGQLKEKNGDVIELKYPLNCADPTSERWFHGHLSGREAEKLLTEKGKNGSFLVRESQSHPGDFVLSV
RTGDDKGDSSEGKPKVTHVMIRCQQDLKYDVGGGEKFDSLTDLVEHYKKNPMVETLGTVLQLKQPLNTTRINAA
EIESRVKELSKLAEATDKVKQGFWEEFETLQQQECKLLYSRKEGQRAENKNKNRYKNILPFDHTRVVLNDGDVNE
PGSDYINANIIMPEFETKCNNSKSKKCYIATQGCLQNTISDFWRMVFQENSRVIVMTTKEVERGKSKCVKYWPDV
SALKEYGAMRVRNVKENSAHDYILRELKLSKVGQGNTERTVWQYHFRAWPDHGVPGDPGGVLDFLEEVNLKQ
ESILEAGPIVVHCSAGIGRTGTFIVIDILIDIIREKGVDCDIDVPKTIQMVRSQRSGMVQTEAQYRFIYMAVQHYIETL
QRRILEEQKSKIKGREYTNIKYSLSDLTGGDQSPLPPCTPTSACAEMRDDSSRVYENVGLMQQQKSFR

>Spotted_gar_(*Lepisosteus_oculatus*)_SHP-2L

Source: GenBank GFIM01033729 (a TSA)

MTSRRWFHPNITGIEAEQLLLTRGVHGSFLARPSKSNPGDFTLSVRRNDEVTHIKIQNSGDYYDLYGGEKFATLAEL
VQYYTEQHDLLREKSGDIIELKYPLNCKDPTSERWYHGHLSGRDAEKLLTEKGKPGSFLVRESQSKPGDFVLSVLTH
EEKHEYVERKTKVTHVMIRCQAGKYDVGGGERFDTLADLVEHYKKNPMVEKNGIVVHLKQPFNATRINAANIEN
RVRELNKVADNSEKPKQGFWEEFEMLQQQECKLLYPRKEGQRPANKSKNRYKNILPFDTTRVILKEVDPEVPGSD
YINANYIQSVQDDGRLVGVSKMYIATQGCLQNTVEDFWKMVYQENTHVIVMTTKELERGRNKCVRYWPDCDC
SQDFGRVCVRNVEERPAQDYILRILNVTHLDRNEPPRYIWHYQYLSWPDHGVPNEPGGVLSFLDQVNRAQNSV
QDTGPIVVHCSAGIGRTGTIIVIDILIDIINRQGLDCDIDIPKTIQMVRCQRSGMVQTEAQYKFIYMAVQQYIDTVQ
KRLEEEQRNKTKEREYSNIRYPPTENAKQKPGVPASQTPPMVYDDSASVYENLNIKKPKASGISNARR

Teleost fish (Class: Actinopterygii [ray-finned fishes]; Infraclass: Teleostei)

>Asian_bonytongue_ (*Scleropages_formosus*)_SHP-2

(this species also has two more SHP-2 genes that are not shown here)

Source: GenBank XP_018610641

MTSRRWFHPNITGVEAENLLLTRGVDGSFLARPSKSNPGDFTLSVRRNGAVTHIKIQNTGDYYDLYGGEKFATLAE
LVQYYMEHHGQLKEKNGDVIELKYPLNCADPTSERWFHGHLSGREAEKLLTEKGKNGSFLVRESQSHPGDFVLSV
RTGDDKTDGSDGKPKVTHVMIRCQHDQKYDVGGGEKFDSLTDLVEHYKKNPMVETLGTVLQLKQPLNTTRINA
AEIESRVRELSKLAETTDKVKQGFWEEFETLQQQECKLLYSRKEGQRPENKNKNRYKNILPFDHTRVVLRDGDPN
EPGSDYINANIIMPELEFKCNNAKVKKCYIATQGCLQNTVSDFWRMVFQENSCVIVMTTKEVERSKSKCVKYWP
DVSALKEYGAMRVRNVKETLAHDYILRELKLSKVGQGNTERTVWQYHFRAWPDHGVPTDPGGVLDFLEEVNLK
QESILEAGPIVVHCSAGIGRTGTFIVIDILIDVIREKGVDCDIDVPKSIQMVRSQRSGMVQTEAQYRFIYMAVQHYIE
TLQRRIEEEQKSKIKGREYTNIKYSLSDLTSGDQSPLPPCTPTPTCADFRDEGSRVYENVGLMQQQKSFR

>Asian_bonytongue_ (*Scleropages_formosus*)_SHP-2L

Source: GenBank XP_018606817

MMMSRRWFHPNITGVEAEQLLLTRGVHGSFLARPSKSNPGDFTLSVRRKNEVTHIKIQNSGDYYDLYGGEKFATL
AELVQYYTEQHDLLRERNGDVIELKYPLNCKDPTSERWYHGHLSGRDAEKLLMEKGKPGSFLVRESQSKPGDFVL
SVLTHEEKHENADRRTKVTHVMIRCQDGKYDVGGGERFDTLADLVEHYKKNPMVEKSGIVVHLKQPFNATRINA
ANIENRVRELNKVADNSEKPKQGFWEEFEVLQQQECKLLYPRKEGQRLENKSKNRYKNILPFDTTRVVLREADRD
VPGADYINANYIQSKHEEGHLADECKVYIATQGCLQNTVTDFWQMVYQENTHVIVMTTKEMERGRNKCVRYW
PDVDTTKDFGKLSVRNIEERPAQDYVLRELEITRLDTREPPRYIWHYQYLSWPDHGVPNEPGGVLSFLDQVNRAQ
NSIPNTGPIVVHCSAGIGRTGTIIVIDILIDIINRQGLDCDIDIPKTIQMVRRQRSGMVQTEAQYKFIYMAVQQYIDT
AQKRLDEEQRNKMKEREYSNIKYPQMANAKVKANVGSSRSSNSVMNDDPSNVYENLNVKTPKASGSSNTRR

>Tarpon_(*Megalops_atlanticus*)_SHP-2

Source: GenBank KAG7476614

MTSRRWFHPNITGVEAENLLLTRGVDGSFLARPSKSNPGDFTLSVRRNGAVTHIKIQNTGDYYDLYGGEKFATLAE
LVQYYMEHHGQLKEKNGDVIELKYPLNCADPTSERWFHGHLSGREAEKLLTEKGKNGSFLVRESQSHPGDFVLSV
RTGDDKTDSSDSKPKVTHVMIRCQHDLKYDVGGGEKFDSLTDLVEHYKKNPMVETLGTVLQLKQPLNTTRINAA
EIESRVRELSKLAEATDKVKQGFWEEFETLQQQECKLLYSRKEGQRAENKNKNRYKNILPFDHTRVVLNDGDTNE
PGSDYINANIIMPELESKCNNAKLKKSYIATQGCLQNTISDFWRMVFQENSRVIVMTTKEVERGKSKCVKYWPDV
SALKEYGAMRVRNVKETSAHDYILRELKLSKVGQGNTERTVWQYHFRAWPDHGVPTDPGGVLDFLEEVKLKQE
SILEAGPIVVHCSAGIGRTGTFIVIDILIDVIREKGVDCDIDVPKTIQMVRSQRSGMVQTEAQYRFIYMAVQHYIETL
QRRIEEEQKSKIKGREYTNIKYSLSDLSGGDQSPLPPCTPTPTCADMRDDSSRVYENVGLMQQQKSFR

>Tarpon_(*Megalops_atlanticus*)_SHP-2L

Source: GenBank KAG7472747

MTSRRWFHPNITGMEAEQLLLARGVHGSFLARPSKSNPGDFTLSVRRNDEVTHIKIQNSGDYYDLYGGEKFATLA
ELVQYYTEQHDLLRERNGDVIELKYPLNCKDPTSERWYHGHLSGRDAEKLLMEKGKPGSFLVRESQSKPGDFVLS
VLTNEEKHENVDRRTKVTHVMIHYQDGKYDVGGGERFDTLADLVEHYKKNPMVEKSGIVVHLKQPFNATRINA
ANIENRVRELNKVADNSEKPKQGFWEEFEVLQQQECKLLYPRKEGQRAENKSKNRYKNILPFDTTRVELKEADPG
VPGSDYINANYIRNLHEDGRYVDEGKVFIATQGCLQNTVVDFWKMVYQENTHVIVMTTKEMERGRNKCVRYW
PDIDCTKEFGKVRVKNIEERPAQDYVLRELEVTRTDRNDPPRYIWHYQYLSWPDHGVPNEPGGVLSFLDQVNRA
QSAFPQTGPIVVHCSAGIGRTGTIIVIDLLIDIINRQGLDCDIDIPKTIQMVRQQRSGMVQTEAQYKFIYMAVQQYI
DTAQKRLEEEQRNKMKEREYSNIKYPQMTNAKPKPNMTISRSSSSVMNDDSASVYENLNIKNPKTSGSTNSRR

>Oriental_weatherfish_(*Misgurnus_anguillicaudatus*)_SHP-2

Source: GenBank XP_055063481

MTSRRWFHPNITGVEAENLLLTRGVDGSFLARPSKSNPGDFTLSVRRNGAVTHIKIQNTGDYYDLYGGEKFATLAE
LVQYYMEHHGQLKEKNGDVIELKYPLNCADPTSERWFHGHLSGREAEKLLTEKGKNGSFLVRESQSHPGDFVLSV
RTGDDKTDTSDGKPKVTHVMIRCQHDLKYDVGGGEKFDSLTDLVEHYKKNPMVETLGTVLQLKQPLNTTRINAA
EIESRVRELSKLAEATDKVKQGFWEEFETLQQQECKLLYSRKEGQRPENKNKNRYKNILPFDHTRVMLIDGDANE
PGSDYINANIIEPELENKGNNKLKRSYIATQGCLQNTISDFWRMVFQENTRVIVMTTKEVERGKSKCVKYWPDAT
ALKEYGAMRVRNVKETQAHDYILRELKLSKVGQGNTERTVWQYHFRAWPDHGVPGDPGGVLDFLEEVKLKQE
GITGAGPIVVHCSAGIGRTGTFIVIDILIDIIREKGVDCDIDVPKTIQMVRSQRSGMVQTEAQYRFIYMAVQHYIETL
QRRIEEEQKSKIKGREYTNIKYSLSDLSGGDQSPLPPCTPTPTCVEMRDDSSRVYENVGLMQQQKSHR

>Oriental_weatherfish_(*Misgurnus_anguillicaudatus*)_SHP-2L

Source: GenBank XP_055043582

MVRWFHPNITGIEAEHLLLTRGVHGSFLARPSKSNPGDFTLSVRRNDEVTHIKIQNSGDYYDLYGGEKFATLAELV
QYYTEQHDSLRERNGDVIELKYPLNCKDPTSERWYHGHLSGKDAEKLLLEKGKSGSFLVRESQSKPGDFVLSVLTN
EEKHENVDRKTKVTHVMIRFQDGKYDVGGGERFDTLADLVEHYKKNPMVEKSGIVVHLKQPFNATRINAANIEN
RVRELNKVADNSEKPKQGFWEEFEVLQQQECKLLYPRKEGQRPENKNKNRYKNILPFDTTRVQIKEVDPDVLGSD
YINANYIRSTPEEGRHTDEGKVFIATQGCLQNTVLDFWKMVYQENTHVIVMTTKEMERGRNKCVRYWPDLNST
REFGKLSVKNIEEHTAQDYIRRELEVTRLDRREPPRCIWHYQYLSWPDHGVPNEPGGVLSFLEQVNRTQSAIPESG
PIVVHCSAGIGRTGTIIVIDILINIINRQGLDCDIDIPKTIQRVRQQRSGMVQTEAQYKFIYMAVQQYIDTAQKRLEE
EQRNKTKEREYSNIRYPQMTNARAKPNMSSSRTSSVMNDDSSVYENLNIKNPKGSASANTRR

>Zebrafish_(*Danio_rerio*)_SHP-2

Source: GenBank XP_005162214

MTSRRWFHPNITGVEAENLLLTRGVDGSFLARPSKSNPGDFTLSVRRNGAVTHIKIQNTGDYYDLYGGEKFATLAE
LVQYYMEHHGQLKEKNGDVIELKYPLNCADPTSERWFHGHLSGREAEKLLTEKGKNGSFLVRESQSHPGDFVLSV
RTGDDKTDTSEGKPKVTHVMIRCQHDLKYDVGGGEKFDSLTDLVEHYKKNPMVETLGTVLQLKQPLNTTRINAA
EIESRVRELSKLAEATDKVKQGFWEEFETLQQQECKLLYSRKEGQRPENKNKNRYKNILPFDHTRVVLTDGDVNE
QGSDYINANLIMPDNEAKSNNSKLKRSYIATQGCLQNTISDFWRMVFQENSRVIVMTTKEVERGKSKCVKYWPD
VSALKEYGAMRVRNVKETMAHDYILRELKLSKVGQGNTERTVWQYHFRAWPDHGVPGDPGGVLDFLEEVKLK
QEGITGAGPIVVHCSAGIGRTGTFIVIDILIDIIREKGVDCDIDVPKTIQMVRSQRSGMVQTEAQYRFIYMAVQHYI
ETLQRRIEEEQKSKIKGREYTNIKYSLSDLSGGDQSPLPPCTPTPTCADMRDDSSRVYENVGLMQQQKSHR

>Zebrafish_(*Danio_rerio*)_SHP-2L

Source: GenBank XP_005162214

MVRWFHPNITGIEAEHLLLTRGVHGSFLARPSKSNPGDFTLSVRRNDEVTHIKIQNSGDYYDLYGGEKFATLAELV
QYYTEQHDLLRERNGDVIELKYPLNCKDPTSERWYHGHLSGRDAEKLLTEKGKSGSFLVRESQSKPGDFVLSVLTN
EEKHENVDRKTKVTHVMIRYQDGKYDVGGGERFDTLADLVEHYKKNPMVEKSGIVVHLKQPFNATRINAANIEN
RVHELNKVADNSEKPKQGFWEEFEVLQQQECKLLYPRKEGQRPENKNKNRYKNILPFDTTRVQIKEADPDVPGS
DYINANYIRSVNEEGRHMDEGKVFIATQGCLQNTVLDFWKMVYQENTHVIVMTTKEMERGRNKCVRYWPDLN
STKEFGKVCVKNIEEHTAQDYIRRELEVTRLDRREPPRCIWHYQYLSWPDHGVPNEPGGVLSFLEQVNRTQSAIPE
SGPIVVHCSAGIGRTGTIIVIDILIDIINRQGLDCDIDIPKTIQRVRQQRSGMVQTEAQYKFIYMAVQQYIDTAQKRL
EEEQRNKTKEREYSNIKYPQMSNARAKPNMSSSRSSSVMNDDSSVYENLNIKNPKGSTSSNTRR

>Atlantic_salmon_(*Salmo_salar*)_SHP-2

Source: GenBank XP_045548428 (modified based on TSA GIYK01034503)

MTSRRWFHPNITGVEAENLLLTRGVDGSFLARPSKSNPGDFTLSVRRNGAVTHIKIQNTGDYYDLYGGEKFATLAE
LVQYYMEHHGQLKEKNGDVIELKYPLNCADPTSERWFHGHLSGREAEKLLTEKGKNGSFLVRESQSHPGDFVLSV
RTGDDKTDSSDNKPKVTHVMIRCQHDLKYDVGGGEKFDSLTDLVEHYKKNPMVETLGTVLQLKQPLNTTRINAA
EIDSRVRELSKLAEATDKVKQGFWEEFETLQQQECKLLYSRKEGQRAENKNKNRYKNILPFDHTRVVLNDGDGSE
AGSDYINANLMPELEWKCNSTKLKKSYIATQGCLQNTISDFWRMVFQENSRVIVMTTKEVERGKSKCVKYWPD
MSSLKEYGIMRVRNVKETSAHDYILRELKLSKVGQGNTERTVWQYHFRAWPDHGVPTDPGGVLDFLEEVNLKQ
ESILEAGPIVVHCSAGIGRTGTFIVIDILIDVIREKGVDCDIDVPKSIQMVRSQRSGMVQTEAQYRFIYMAVQHYIET
LQRRIEEEQKSKIKGREYTNIKYSLSDLTGGDQLQSPLPPCTPSPTCAEMREDCSRVYENVGLMQQQKSYR

>Atlantic_salmon_(*Salmo_salar*)_SHP-2La

Source: GenBank XP_014001604

Atlantic salmon SHP-2La and SHP-2Lb share 96% amino acid identity. This gene is located on Chr. 15.

MVRWFHPNITGIEAEQLLLTRGVHGSFLARPSKSNPGDFTLSVRRSDEVTHIKIQNSGDYYDLYGGEKFATLAELV
QYYTEQHDLLRERNGDVIELKYPLNCKDPTSERWYHGHLSGRDAEKLLTDKGKPGSFLVRESQSKPGDFVLSVLTN
EEKHENVDRKTKVTHVMIRYQQDGKYDVGGGERFDTLADLVDHYKKNPMVEKSGIVVHLKQPFNATRINAANI
ENRVKELNKVADNSEKPKQGFWEEFEVLQQQECKLLYPRKEGQTAENKSKNRYKNILPFDTTRVEIREADADVPG
SDYINANYIRSMHEEGCHVEEGKVFIATQGCLQNTVVDFWKMVYQENTHVIVMTTKEMERGRNKCVRYWPDL
NATKEFGKVCVKNVEERPAQDYILRELEVTRLDRREPMRYIWHYQYLSWPDHGVPNEPGGVLSFLEQVNRTQSTI
QDTGPIVVHCSAGIGRTGTIIVIDILIDIINRQGLDCDIDIPKTIQRVRQQRSGMVQTEAQYKFIYMAVQQYIDTAQK
RLEEEQRNKTKEREYSNIKYPQMTNARSKPNMTCVSRSSSVVTNDDPSAVYENLNIKNPSTSGNSSNTRK

>Atlantic_salmon_(*Salmo_salar*)_SHP-2Lb

Source: GenBank XP_013991103

Atlantic salmon SHP-2La and SHP-2Lb share 96% amino acid identity. This gene is located on Chr. 13.

MTSRRWFHPNITGIEAEQLLLTRGVHGSFLARPSKSNPGDFTLSIRRNNEVTHIKIQNSGDYYDLYGGEKFATLAEL
VQYYTEQHDLLRERNGDVIELKYPLNCKDPTSERWYHGHLSGRDAEKLLMDKGKPGSFLVRESQSKPGDFVLSVL
TNEEKHENVDRKTKVTHVMIRYQQDGKYDVGGGERFDTLADLVDHYKKNPMVEKSGIVVHLKQPFNATRINAA
NIENRVKELNKVADNSEKPKQGFWEEFEVLQQQECKLLYPRKEGQRAENKSKNRYKNILPFDTTRVEIREADTDV
PGSDYVNANYIRSMHEEGRHLEEGKVFIATQGCLQNTVVDFWKMVYQENTHVIVMTTKEMERGRNKCVRYW
PDLNATKEFGKVSVKNVEECPAQDYILRELEVTRLDRRELVRYIWHYQYLSWPDHGVPNEPGGVLSFLEQVNRTQ
SAIPDTGPIVVHCSAGIGRTGTIIVIDILIDIINRQGLDCDIDIPKTIQRVRQQRSGMVQTEAQYKFIYMAVQQYIDTA
QKRLEEEQRNKMKEREYSNIKYPQMTNTRSKSNMTCVSRSSSVVTNDNPSSVYENLNIKNPKTSGSSSNTRR

>Barramundi_perch_(*Lates_calcarifer*)_SHP-2

Source: GenBank XP_018552564

MTSRRWFHPNITGVEAENLLLTRGVDGSFLARPSKSNPGDFTLSVRRNGAVTHIKIQNTGDYYDLYGGEKFATLAE
LVQYYMEHHGQLKEKNGDVIELKYPLNCADPTSERWFHGHLSGREAEKLLTEKGKNGSFLVRESQSHPGDFVLSV
RTGDDKTDSSDSKPKVTHVMIRCQHDLKYDVGGGEKFDSLTDLVEHYKKNPMVETLGTVLQLKQPLNTTRINAA
EIESRVRELSKLAEATDKVKQGFWEEFETLQQQECKLLYSRKEGQRAENKNKNRYKNILPFDHTRVVLNDGDLNE
PGSDYINANIIMPELDSKCNSTKVKKSYIATQGCLQNTISDFWRMVFQENSRVIVMTTKEVERGKSKCVKYWPD
MNALKEYGAMRVRNVRETAAHDYILRELKLSKVGQGNTERTVWQYHFRAWPDHGVPTDPGGVLDFLEEVNLK
QESILDAGPIAVHCSAGIGRTGTFIVIDILIDVIREKGVDCDIDVPKTIQMVRSQRSGMVQTEAQYRFIYMAVQHYI
ETLQRRIEEEQKSKIKGREYTNIKYSLSDLTGGEQSPLPPCTPIPTPTCTEMREDSSRVYENVGLMQQQKSYR

>Atlantic_salmon_(*Salmo_salar*)_SHP-2L

Source: GenBank XP_014001604

MVRWFHPNITGIEAEQLLLTRGVHGSFLARPSKSNPGDFTLSVRRSDEVTHIKIQNSGDYYDLYGGEKFATLAELV
QYYTEQHDLLRERNGDVIELKYPLNCKDPTSERWYHGHLSGRDAEKLLTDKGKPGSFLVRESQSKPGDFVLSVLTN
EEKHENVDRKTKVTHVMIRYQQDGKYDVGGGERFDTLADLVDHYKKNPMVEKSGIVVHLKQPFNATRINAANI
ENRVKELNKVADNSEKPKQGFWEEFEVLQQQECKLLYPRKEGQTAENKSKNRYKNILPFDTTRVEIREADADVPG
SDYINANYIRSMHEEGCHVEEGKVFIATQGCLQNTVVDFWKMVYQENTHVIVMTTKEMERGRNKCVRYWPDL
NATKEFGKVCVKNVEERPAQDYILRELEVTRLDRREPMRYIWHYQYLSWPDHGVPNEPGGVLSFLEQVNRTQSTI
QDTGPIVVHCSAGIGRTGTIIVIDILIDIINRQGLDCDIDIPKTIQRVRQQRSGMVQTEAQYKFIYMAVQQYIDTAQK
RLEEEQRNKTKEREYSNIKYPQMTNARSKPNMTCVSRSSSVVTNDDPSAVYENLNIKNPSTSGNSSNTRK

>Barramundi_perch_(*Lates_calcarifer*)_SHP-2L

Source: GenBank XP_050927024

MVRWFHPNITGIEAEQLLLTRGVHGSFLARPSKSNPGDFTLSVRRNDEVTHIKIQNSGDYYDLYGGEKFATLAELV
QYYTEQQDLLRERNGHVIELKYPLNCKDPTSERWYHGHLSGRDAEKLLTDKGKAGSFLVRESQSKPGDFVLSVLT
NEEKHENVDRKTKVTHVMIRYQQDGKYDVGGGERFDTLTDLVDHYKKNPMVEKSGIVVHLKQPFNATRINAAN
IENRVRELNKVADNSEKPKQGFWEEFEVLQQQECKLLYPRKEGQKPENKSKNRYKNILPFDTTRVEIREKDPDVP
GSDYINANYIRSMHEEGRHMEEGKVFIATQGCLQNTVIDFWKMVYQENTHVIVMTTKEIERGRNKCVRYWPDL
NATKEFGKVLVRNVDERPAQDYILRKLEVTRLDRLKEHSRYIWHYQYLSWPDHGVPNEPGGVLWFLEEVNRTQS
TIPDAGPIVVHCSAGIGRTGTIIVIDILINIINRQGLDCDIDIPKTIQRVRQQRSGMVQTEAQYKFIYMAVQQYIDTA
QKRLEEEQRNKMEREYSNIKYPQMTNSRSKPNMASSRSSSVMTNDDSSSVYENINFKSPQTSFSSNTRR

>Medaka_(*Oryzias_latipes*)_SHP-2

Source: GenBank XP_023816374

MTSRRWFHPNITGVEAENLLLTRGVDGSFLARPSKSNPGDFTLSVRRNGAVTHIKIQNTGDFYDLYGGEKFATLAE
LVQYYMEHHGQLKEKNGDVIELKYPLNCADPTSERWFHGHLSGREAEKLLTEKGKNGSFLVRESQSHPGDFVLSV
RTGDDKTDSSDGKPKVTHVMIRCQHDLKYDVGGGEKFDSLTDLVEHYKKNPMVETLGTVLQLKQPLNTTRINAA
EIESRVRELSKLAEATDKVKQGFWEEFETLQQQECKLLYSRKEGQRAENKNKNRYKNILPFDHTRVVLNDGDPNE
PGSDYINANIIMPELDAKCNSHKLKKSYIATQGCLQNTISDFWRMVFQENSRVIVMTTKEVERGKSKCVKYWPD
MSALKEYGAMRVRNVRETSAHDYILRELKLSKVGQGNTERTVWQYHFRAWPDHGVPTDPGGVLDFLEEVNLK
QESIADAGPIVVHCSAGIGRTGTFIVIDILIDVIREKGVDCDMDVPKTIQMVRSKRSGMVQTEAQYRFIYMAVKHY
IETLQRRIEEEQKSKIKGREYTNIKYSLSDLTAGEQGPLTPCTPLPTPTCTEMRDDSSRVYENVGLMQQQKSFR

>Medaka_(*Oryzias_latipes*)_SHP-2L

Source: GenBank XP_011474971

MTSRRWFHPNIIGVEAEQLLLTRGVHGSFLARPSKSNPGDFTLSVRRSDEVTHIKIQNSGDYYDLYGGEKFATLAEL
VQYYTEQQDLLRERNGHVIELKYPLNCKDPTSERWYHGHLSGKEAEKMLTDKGKAGSFLVRESQSKPGDFVLSVL

TNEEKHENVDRKTKVTHVMIRFQDGKYDVGGGEKFDTLTDLVEHYKKNPMVEKSGIVVHLKQPFNATRINAANI
ENRVRELNKVADNTEKPKQGFWEEFEVLQQQECKLLYPRKEGQKPENKSKNRYKNILPFDTTRVVIRDADPEVAG
SDYINANYIRQSMLEDGRHVVEGKVFIATQGCLQNTVVDFWKMVHQENTQVIVMTTKETERGRNKCARYWPD
LHCTKEFGRLLVRNVDERPAQDYILRKLEVTCSDREPLRYIWHYQYLSWPDHGVPNEPGGVLWFLEEINRTQNSV
KEAGPIVVHCSAGIGRTGTIIVIDILIDIISRQGLDCDIDIPKTIQRVRQQRSGMVQTEAQYKFIYMAVQQHIDTAQK
RLEEEQRNKMKEREYSNIKYPQMTNSRSKQNTASSRSSSVMTNEDSTGVYENINFKSPQASFSSNTRR

>Mummichog_(*Fundulus_heteroclitus*)_SHP-2

Source: GenBank XP_035996548

MTSRRWFHPNISGVEAENLLLTRGVDGSFLARPSKSNPGDFTLSVRRNGAVTHIKIQNTGDYYDLYGGEKFATLAE
LVQYYMEHHGQLKEKNGDVIELKYPLNCADPTSERWFHGHLSGRDAEKLLTEKGKNGSFLVRESQSHPGDFVLS
VRTGDDKTDTNDGKPKVTHVMIRCQGDLKYDVGGGEKFDSLTDLVEHYKKNPMVETLGTVLQLKQPLNTTRIN
AAEIESRVRELSKLAEATDKVKQGFWEEFETLQQQECKLLYSRKEGQRPENKNKNRYKNILPFDHSRVVLDDGDPS
EAGSDYINANIIMPEADIKCNNAKPKKSYIATQGCLQNTISDFWRMVFQENSRVIVMTTKEVERGKSKCVKYWPE
MSALKEYGAMRVRNVRETNAHDYILRELKLSKVGQGNTERTVWQYHFRTWPDHGVPTDPGGVLDFLEEVNLK
QEGILDAGPITVHCSAGIGRTGTFIVIDILIDVIREKGVDCDIDVPKTIQMVRSQRSGMVQTEAQYRFIYMAVQHYI
ETLQRRIEEEQKSKIKGREYTNIKYSLSDLSGGEQSPLPLCSPIPTPVCTEMRDDSSRVYENVGLMQQQKTFR

>Mummichog_(*Fundulus_heteroclitus*)_SHP-2L

Source: GenBank XP_035984821

MVRWFHPNITGIEAEQLLLTRGVHGSFLARPSKSNPGDFTLSVRRNDEVTHIKIQNSGDYYDLYGGEKFATLAELV
QYYTEQQDLLRERNGHVIELKYPLNCKDPTSERWYHGHLSGRDAEKLLTEKGKAGSFLVRESQSKPGDFVLSVLTN
EEKHDNVDRKTKVTHVMIRFQEGKYDVGGGERFDTLADLVEHYKKNPMVEKSGIVVHLKQPFNATRINAANIEN
RVRELNKVADNSEKPKQGFWEEFEVLQQQECKLLYPRKEGQKPENKSKNRYKNILPFDTTRVVIKDSESDVPGSD
YINANYIRNMNEDGRHVEESKVFIATQGCLQNTVIDFWKMVYQENAHVIVMTTKEMERGRNKCVRYWPDLHG
TKEFGKVLLRNVEERPAQDYVLRKLEVTRLDRKEPQRHIWHYQYLSWPDHGVPNEPGGVLWFLEEVNRTQGTIK
EAGPIIVHCSAGIGRTGTIIVIDILIDVINRQGLDCDIDIPKTIQMVRQQRSGMVQTEAQYKFIYMAVQQYIDTAQK
RLEEEQRNKMKEREYSNIKYPQMTNSRSKHNMASSRSSSVMTNDDSSSVYENINFRTPQTSFSSNTRR
