# Supplementary material for: PD-1 is conserved from sharks to humans: new insights into PD-1, PD-L1, PD-L2, and SHP-2 evolution
Source: Front Immunol. 2025 May 28;16:1573492. doi: 10.3389/fimmu.2025.1573492 (PMC12151841; doi:10.3389/fimmu.2025.1573492)

Supplementary file 2

Sequence alignment (A) and phylogenetic tree (B) of deduced PD-L1 and PD-L2 amino acid sequences in representative species

(A) Sequence alignment.

Deduced amino acid sequences of PD Ligand 1 (PD-L1) and ligand 2 (PD-L2) sequences in representative species are aligned, with a highlighting of the conserved F19 and D122 residues involved in PD-1 binding, and of the (rather) PD-L2 specific residues L150, aromatic residue at 166, G172, Y174, N189, and S191. PD-L2 sequences are only found in tetrapod species. We deem most of the alignment as quite reliable in regard to the proper matching of evolutionary-related stretches, but the cytoplasmic regions have so rapidly diverged that their alignment is largely speculative. In most but not all of the ray-finned fish that we investigated, a large cytoplasmic tail extension was found with a well-conserved sequence, encoded by exon 8.

In all investigated tetrapod species we found the assigned *PD-L2* gene downstream of *PD-L1*, including in amphibians. Therefore, although the phylogenetic tree analysis (see [B]) is only weakly supportive of all tetrapod *PD-L2* genes being orthologous, the most parsimonious explanation is that they are the same gene indeed.

Residue numbering above the alignment follows the human PD-L1 mature protein. The numbers between brackets refer to introns and to their phases at the indicated position (0) or in the preceding codon (1, 2). Cysteines are in purple and, based on Hopp and Woods, 1981 (doi: 10.1073/pnas.78.6.3824) : red font is used for basic residues, blue for acidic residues, and of the other residues (green and orange) the more hydrophilic ones are in green.

The sequences and their sources are shown in Supplementary file 1. The species are human (*Homo sapiens*), mouse (*Mus musculus*), cattle (*Bos taurus*), Platypus (*Ornithorhynchus anatinus*), chicken (*Gallus gallus*), goose (swan goose; *Anser cygnoides domesticus*), lizard (green anole lizard; *Anolis carolinensis*), turtle (green sea turtle; *Chelonia mydas*), frog (tropical clawed frog; *Xenopus tropicalis*), lungfish (West African Lungfish (*Protopterus annectens*), shark (small-spotted catshark; *Scyliorhinus canicula*), skate (Thorny skate; *Amblyraja radiata*), bichir (gray bichir (*Polypterus senegalus*), reedfish (reedfish; *Erpetoichthys calabaricus*), sturgeon (sterlet sturgeon; *Acipenser ruthenus*), paddlefish (Mississippi paddlefish; *Polyodon spathula*), gar (spotted gar; *Lepisosteus oculatus*), bonytongue (Asian bonytongue ; *Scleropages formosus*), tarpon (*Megalops atlanticus*), weatherfish (oriental weatherfish *Misgurnus anguillicaudatus*), zebrafish (*Danio rerio*), salmon (Atlantic salmon; *Salmo salar*; the PD-L1a form), perch (Barramundi perch; *Lates calcarifer*), medaka (*Oryzias latipes*), mummichog (*Fundulus heteroclitus*).

Exon-1

Leader peptide

```
                                          .1              .10
Human PD-L1                               MRIFAVFI-----FMTYW-HLL-NA(1)
Mouse PD-L1                               MRIFAGII-----FTACC-HLL-RA(1)
Cattle PD-L1                              MRIYSVLT-----FMAYC-CLL-KA(1)
Platypus PD-L1                            MKILPVFT-----FMLDW-QLL-NA(1)
Chicken PD-L1                             MEKLLLLHI----FLFCW-RSL-NA(1)
Goose PD-L1                               MEKPLLLCM----FSFYW-HFL-NA(1)
Turtle PD-L1                              MENALLLCI----FVSHW-HFL-NA(1)
Frog PD-L1                              MAMHRRLIIAV---LFLCHC-GVL-SA(1)
Newt PD-L1                              MGKMEKTFLVF---TCIFKW-NYI-NA(1)
Human PD-L2                               MIFLLLMLS----LELQL-HQI-AA(1)
Mouse PD-L2                               MLLLLPILN----LSLQL-HPV-AA(1)
Cattle PD-L2                MMRLPQSD(1)PVQSMFLLLILS-----LGLQL-QQT-VA(1)
Platypus PD-L2                            MCLFLLVLI----LEMQL-HLM-PA(1)
Chicken PD-L2                                                MLL-RG(1)
Goose PD-L2                               MFQILTMLL----LEMQL-WVV-SG(1)
Lizard PD-L2                              MFGPLLVVL----VQIQL-HLV-SA(1)
Turtle PD-L2                              MFRILPILI----LEVQL-YLI-RA(1)
Frog PD-L2                       MLVMKEEK(0)MERKIFCLL-CILLMILDGHSV-LA(1)
Newt PD-L2                                MSPLIFMIV----MVNLL-HPQ-TA(1)
Lungfish PD-L1                            MNGLRFSLT----FALYW-CSV-TA(1)
Shark PD-L1                               MKIISLVLI----LGVHL-PLM-TA(1)
Skate PD-L1                               MKIISLLFI----LGAHL-PLD-TA(1)
Bichir PD-L1        MWASGVHAGPVRVGAAPE(1)KIMKIFQLLV-----LISQW-PAL-PA(1)
Reedfish PD-L1      MWAAGIHAGPVRVGAASE(1)KIMKIFQLLV-----LISQW-PAL-PA(1)
Sturgeon PD-L1                            MKIILLVIV----LEFHW-PVL-QA(1)
Paddlefish PD-L1                          MKMILLVVV----LAFHW-PVL-RA(1)
Gar PD-L1                                 MDNSLLIIF----QVAMW-PMF-PA(1)
Bonytongue PD-L1                          MAHHLLLL-----QVGFFLPSV-QA(1)
Tarpon PD-L1                              MDTALLIL-----QIVLW-PGV-PA(1)
Weatherfish PD-L1                         MKGPLLLMC----QALLC-SAV-SG(1)
Zebrafish PD-L1                  MCSIHQ(0)GSMKRTLVIIF----QALLW-PAVLSA(1)
Salmon PD-L1                              MEQAFLLVL----QVVLW-PTL-AA(1)
Perch PD-L1                               MDWALFVIL----QVIFQ-PSL-SV(1)
Medaka PD-L1                              MDWVFAIIL----QVMIQ-PSL-SV(1)
Mummichog PD-L1                           MDWVIAVVVVVVLQSFLQ-PSV-AA(1)
```

Exon-2

IgSF domain 1

```
                      .F19      .30       .40       .50         .60            .70       .80       .90       .100         .110         .D122   .130
Human PD-L1            FTVTVPKDLYVVEYGSNMTIECKFPVEKQLDL-AALIVYWEMED------KNIIQFVHGEEDLKVQHSSYRQRARLLKDQLSLGNAALQ---ITDVKLQDAGVYRCMISYGGA--DYKRITVKVNA(1)
Mouse PD-L1            FTITAPKDLYVVEYGSNVTMECRFPVERELDL-LALVVYWEKED------EQVIQFVAGEEDLKPQHSNFRGRASLPKDQLLKGNAALQ---ITDVKLQDAGVYCCIISYGGA--DYKRITLKVNA(1)
Cattle PD-L1           FTITVSKDLYVVEYGSNVTLECRFPVDKQLNL-LVLVVYWEMED------KKIIQFVNGKEDPNVQHSSYHGRAQLLKDQLFLGKAALQ---ITDVKLQDAGVYCCLISYGGA--DYKRITLKVNA(1)
Platypus PD-L1        LFEVEVLKESFTVVYGSNVTMECSFPFKDRLDL-EALSVYWDTEDD-----KHIVKFVKGKVDLKIQHHSYRGRATLLKDKLLLGKAMLQ---ITNVQLTDAGVYRCLIGYEGA--DYKWITLTVQA(1)
Chicken PD-L1 )       LFTVEAPKSLYTAELGSNVTMECVFPVNGKLKF-RDLSVIWEKKDEVR---KDVYILLKGKEDSGSQHSDFQGRIKLLKENLDFGQSLLQ---ISNVKLRDAGLYHCLIEYGGA--DYKTINLKVQA(1)
Goose PD-L1           LFTVEAPQSLYTVELGNNVTMECTFPVNGKLKF-GDLSVSWEKKEELG---KDVYVLLKGEEDFKSQHSDFRGRIKLLKENLKLGQSLLQ---IMDVKLRDAGFYRCLIDYGGA--DYKTINLKVQA(1)
Turtle PD-L1          LFTVEVPQPQYIVEYGSNVTMECRFRVNGQLKL-QDLSVIWEKKEEHT---KEVYKLHKGKENFNNQHSSYSGRVQLLKDKLQFGRSMLQ---VTSVKFTDAGTYLCLIGYEGA--DYKMIALQVKA(1)
Frog PD-L1            LFTVKAGKSHYTAEYGGKVNMECHFQVGKGTKA-DDVEVYWEYIAAEGG-RKEVIKLIRGTENLSAQHEDYRGRVRILKEELHKGHAVLQ---ISNVELTDSGRYICIISAQGS--DYKSMGLTVQA(1)
Newt PD-L1            LFSVKVPESSYTVQIGTTVDLECNFPVIDTLRI-KDLTITWLWRKSPETDQQEVYVFHNGKEDISKQDSRYKGRATLVKDTLFTGRVVLQ---ISNIRLSDRGTYQCLIGYGGA--DYRHIDLLVEA(1)
Human PD-L2           LFTVTVPKELYIIEHGSNVTLECNFDTGSHVNL-GAITASLQK--------------------VENDTSPHRERATLLEEQLPLGKASFH---IPQVQVRDEGQYQCIIIYGVAW-DYKYLTLKVKA(1)
Mouse PD-L2           LFTVTAPKEVYTVDVGSSVSLECDFDRRECTEL-EGIRASLQK--------------------VENDTSLQSERATLLEEQLPLGKALFH---IPSVQVRDSGQYRCLVICGAAW-DYKYLTVKVKA(1)
Cattle PD-L2          LFTVTIPKEMYMVDYGSNVTLECDFDTGGPVEL-GILKASLQK--------------------VENDTVLLSERATLLEEQLPLGKALFL---IPRIQLKDAGQYRCLIIYGIAW-DYKYLTLKVKA(1)
Platypus PD-L2        LFTVQIPKDFYTVDYGSNVTMECNFNVENQMDF-NILLVFWDKDEKN------IVKFAKGQEDLKTQDEHYRGRATLLREELSSGKALLR---ISDVKITDAGQYRCLISYGGA--DYKYITLKVKA(1)
Chicken PD-L2      AEELFTVEVPQQLYVVEYGSNVTMECRFPVNGSLNL-GLLSVVWEQKRQGQLESREVYTLRNGKALTSSQHHDYMGRAALLRNELKLGRAILH---ITSVKITDAGSYLCLIDYQGA--DYKYITLEVKA(1)
Goose PD-L2           LFTVEVPQQLYVAAYGSNVTMECRFPVNGSLNL-GLLSVVWEQKRQGQPESRDVYTLHKGQALPPSQHHDYMGRAALLHDELKSGRAILQ---ITRVKVTDAGSYLCLIDYQGA--DYKYITLEVTA(1)
Lizard PD-L2          LFTVEVLQPRYFAEHGGTVTMGCRFPVHDPFNL-TNLSVLWQRKPSQGNEVKEVYKLSKGQEDLRQQHADYQDRARVAPSELKIGLSMLC---LNNVKIADSGIYVCLVHYEGS--DLKYIYLDVKA(1)
Turtle PD-L2          FFTVEVPQLQYIAEYGSNVTMECRFPVDGQLNL-KDLSVSWEQKGLKEQKPKEVYTLQKGEEDLKSQHRDYRGRATLLRDKLNLGYSVLQ---ITSVKLMDAGSYLCLIDYRGA--DYKYITLEVKA(1)
Frog PD-L2            LFVVTAPRSSYTAQYGDTVQLICSFPPEENVYISKKLKVSWEHIDSFQGKSQDVLMLTDGKLVLEKQSDTFRGRTTLLMEELNNGRAVLE---ITNVKLTDSGKYRCVLQLDGS--DYKTISLKVKA(1)
Newt PD-L2            LFSVEVPQSSFIVRVGSTVALECHFPLSGTLRV-EELIVIWLHSAPSQPDQRDVIAFERGKIDLSVQDASYKGRAILLNEALYKGQAVLQ---ITNVKLSDSGIYSCIIGYGGA--DYKHIQLTVGA(1)
Lungfish PD-L1        LFTVEMTRSSYVAEHNTVATLECKFPIDKKYGI-QDLKIFWHKIVNSSLE-LEVFKFFNGSEDFLMQDVIYKGRARLRQALLGNGRAVLE---INPVKVTDTGVYRCLIELHGA--DYKQGTLEVKA(1)
Shark PD-L1           IFTVTAPRLSYTASYGNNITVECRFPVESNFNS-NQIKLYWHHILSDGSS-QLVYKLFNGKPALQDQSQEYSERVFMLLDELRSGRAVLE---INRVRVSDAGTYRCVIDLNGV--DYKETALEVTA(1)
Skate PD-L1           LFMVTAPRLSYTASYGNNITMECIFPVESNFHM-NTLKVYWYHILDNGTS-QLVYKLLNGKAALQDQSFEYRERVFLAMDKLFNGRAVVE---ITQVRVSDAGTYRCIIALNGV--DYKETALKVTA(1)
Bichir PD-L1          LFTVEMLKPLYTVEFKDTVRIECRFSINDNFQQ-DHLSVFWHQLLPNNTD-LEVFRMFRGTESLKSQHSRYKGRASLMTEPLKDGLAMLQ---ISDVQIEDSGRYRCLIDLNGDP-DYKETTLSVKA(1)
Reedfish PD-L1        LFTVEMLKPLYTVEFKDTVRIECRFSINDNFQQ-DHLSVFWHQLLPNNTD-LEVFRMFRGTESLKSQHTRYKGRASLMTEPLKDGLAVLQ---ISNVQIEDSGRYRCLIDLNGDP-DYKETTLSVKA(1)
Sturgeon PD-L1        LFTVEMAKALYLAEFGNTVKMECRFPTGGSLDS-IN--VYWHRMLSNGSE-YEVYTLLNGNEDLQSQHPEYKGRAHMKPDLLRKGRAELE---ISNVKISDSGSYRCLIKMGGA--DYKQATLSVKA(1)
Paddlefish PD-L1      LFTVEMIKTLYLAEFGNTVKMECRFPTGGSLDS-IN--VYWHRMLSNGSE-YEVYTLVNGNEDLQSQHPVYKGRAHMKPDLLRKGRAELE---ISNVKISDSGSYRCLIKMGGA--DYKHATLSVTA(1)
Gar PD-L1             LFTVDMTESSFLAEFRGNVTMECRFPTGGGETL-SSLRVYWHRILPEPL--LEVYKLESGKEDLSTQHPRYKGRVNLQKDRLKQGQAVLQ---MSNLTISDSGKYRCIVEQGGA--DYKEATLNVRA(1)
Bonytongue PD-L1      LFTVEVASPSYTAEFSGDVAMECKFGP--MDSK-SSLSVRWQRILPKPL--LRVYNLENGQEDLSFQDSQYRGRVQLMKDKLSSGRAILN---ISNVKINDSGTYECLVEMVGA--DFKRTTLTVKA(1)
Tarpon PD-L1          LFTVEVTKPFHLAEFRGNVTMECIFLP--GGSE-ESLSVFWRRILPEPP--MEVYKLENGKEDLSSQAPQYRDRVRLLRDELRRGRAALQ---ISHLRINDSGTYQCLVEMGGA--DYKQTTLTVKA(1)
Weatherfish PD-L1     LFTVDVEQKSYESELHGDVKLVCLFSQ--VKSL-SDLLVIWHRIEPLPE--INVYRYERGKEKQNYTNVVFGDRAQLIHEQLSQSRAVLH---LKKLRIKDSGTYQCVVKYQQDDVDYKHITLSVTA(1)
Zebrafish PD-L1       SFTVNVPRSTYEAELNGDVRLECVFSA--LKRS-SDITVIWSRVHPKPD--VNIYWLDKGKEIHNHTSSAFHKRAQLISHLLRENRAVLH---LKKLRIKDSGTYQCIVE--GDEVDYKQITLNVTA(1)
Salmon PD-L1          LFTVEVDSPFHVAEFHGVVTMGCRFQP--GGQG-PNLSVIWHRIWPPPV--VEVYRLENRQEDLTSQNPQYRGRVRLVTEEMTNGWAKLE---VSMLRINDSGTYQCLVEMSGA--DYKQTTLTVKA(1)
Perch PD-L1           LFTVEAERPTYTSEFGGDVVMGCRFQPKPSNPQ-ADLKVSWRWLGSTSP--WEVYQMNNGKEH--SASPEYQGRVRLLTEQLNEGWAKLQ(0)VSRLRINDSGVYQCLVQTGEGA-DYKAITLSVIA(1)
Medaka PD-L1          LFTVEAEQTMYSSEFGGEVVMGCRFSKKATQPN-SDLKVTWHWTSSGLH--QELIRLDNTADY--SVPPKYQGRVKLLTEELKNGWAKLQ(0)LSNLRINDSGTYQCLVQTTDGT-DYKTMTLSVKA(1)
Mummichog PD-L1       LFTVEAEQATYMSEFGGNVVMGCKFSSNPANPH-RDLKVNWHRKTNGIY--EEVIRLEDNLEN--SASPKYQGRVELLTGELKNGWAKLK(0)ISHLKMNDSGTYQCLVQTAEGN-DYKEIALSVEA(1)
```

```
                    Exon-3
                             IgSF domain 2
                         .140        .150      .160      .170      .180      .190      .200      .210      .220
Human PD-L1          PYNKINQRILVV--DPVTSEHELTCQAEGYPKAEVIWTSSDHQVLSGKTTTTNSKREEKLFNVTSTLRINTTTNEIFYCTFRRLDPEE-NHTAELVI--PE(1)
Mouse PD-L1          PYRKINQRISV---DPATSEHELICQAEGYPEAEVIWTNSDHQPVSGKRSVTTSRTEGMLLNVTSSLRVNATANDVFYCTFWRSQPGQ-NHTAELII--PE(1)
Cattle PD-L1         PYRKIYHTISV---DPVTSEHELTCQAEGYPEADVIWTSSDHQVLSGKTSITSSKREEKLFNVTSTLRINTTADKIFYCTFRRLGHEE-NNTAELVI--PE(1)
Platypus PD-L1       PYSKINQRISR---NPTTLEYEMTCQSEGYPEASVIWKNNYHEDLSDKAITNSSRGPDMLFNVTSTLGINATVNDTFYCFFWNKKAEE-NTTAVLII--PE(1)
Chicken PD-L1        PYRTITQEVV----STGDKEWKLTCQSEGYPKAEVMWQNGECQDLTDKANTSYETGSDQLYRVTSTLTVKNRTCENFRCIFWNKEIQE-NTSANLYI--LD(1)
Goose PD-L1          PYRNITQGVV----STGDKEWKLTCQSEGYPKAEVIWQNGEYEDLTDKADTSYETGSDQLYRVTSTLTIKNRTHENFRCIFWNKELQK-NTSAILYI--AD(1)
Turtle PD-L1         PYRNITKRTVIVQRTAGQNEWELTCQSKGYPKAEVIWHNGEHQDFSDKANTSYERGTDQLYSVTSTLKTNTSINETFHCIFWNKEFKE-NTSAILII--PD(1)
Frog PD-L1        HYKEINIRVTDIMA---SSGDIVKQIECQSIGYPEAEVTWVHGEK-NLSSLVNTSYTVTAAKLFNVTSVVRVSSISNNTFTCMFWNEALQE-ATVLTFTI--PV(1)
Newt PD-L1           PYSTINTKLSTFLTDSGMQERQMECQSQGYPKAEVIWLNEWK-DISEKAITSYTVSTDLLHNVTSVLQIKGSENNTYHCIFWNKELQQ-NTTATFIL--SD(1)
                                              .150                .166  .172             .189
Human PD-L2          SYRKINTHILK---VPETDEVELTCQATGYPLAEVSWPN-----VSVPANTSHSRTPEGLYQVTSVLRLKPPPGRNFSCVFWNTHVRE-LTLASIDL--QS(1)
Mouse PD-L2          SYMRIDTRILE---VPGTGEVQLTCQARGYPLAEVSWQN-----VSVPANTSHIRTPEGLYQVTSVLRLKPQPSRNFSCMFWNAHMKE-LTSAIIDP--LS(1)
Cattle PD-L2         SYKKINTRHLK---VPGTDEVELTCQAEGYPLAEVSWPN-----ISIPTNTSHTKTSEGLYQVTSVLRLKPHPGRNFSCVFWNANVKE-LTSATIV---QG(1)
Platypus PD-L2       SYKPVNTQILA---APGGDEEDLICQAKGFPLAEVSWQN-----VSAPANTSYTRTPDGLYHITSVLRLPANSNKNVSCIFWNKDVNE-RTTANLDT--PA(1)
Chicken PD-L2        SYKIINTQKTR---EWNENKFALICQSEGFPLAEVFWQNEKNFSLSESANTTYALTADGLYNVTSILIINQNMNENYSCIFWNKELNE-NTSADIYS--LA(1)
Goose PD-L2          SYRIINTQVMR---KGNEEKFVFICQSEGFPLAEVFWQNEKNISLTGSANTTYMLTADGLYNVTSILMFKPNTSENYSCIFWNKELNE-ETSAHFST--LA(1)
Lizard PD-L2         PYKRIHFQK-----RKEKTELNLTCQSQGYPLAEVSWQNEKSTNLSKFAFTTHQMTEDGLFNITSTLRVKPRNHGNYSCVFWNRELNE-NTSAHGAVSSLD(1)
Turtle PD-L2         SYTRINVQIMS---EPAEEELVLTCQSEGFPLAEVFWQNEKNLKVNVSVNTTYTLTTDGLYNVTSMLTFKPNASGNYSCTFWSKELNE-ETSYIFT---LA(1)
Frog PD-L2           SYKIIDIY-------RSENEDLLTCQSLGFPIAEVSWQNNGD-NVSLPSNFSQFLRPDGVYNITSTIRISRDVTQNYTCVFWNKELNE-KTQASFH---LEPYQG(1)
Newt PD-L2           SYERIHTQTEV---VTGVKELILTCGAKGFPLPDVFWQCNKV-NISLPSNTSHTVTEDGLYNITSTIRSDLRLGKDCSCLFWNKDLQE-TTSAKLF---TEG(1)
Lungfish PD-L1       SYRNIEKHVTMIGSVNGKQQLELFCHSNGFPKAEVLWNSGTQ-NLSAQAKTSFKRDPEDLYLMTSFIRVIPSYNDTYSCIFMSNNLRE-SISNF-TY--LD(1)
Shark PD-L1          SYGEIETIK-----TKEKKETELICQSMGYPLAEVVWYYENGSDINETANTTHFTGTNGLYNIRSVIRIKEETDDNYLCMFWIKELNM-NTSAILQN--KE(1)
Skate PD-L1          SYNDIKTLN-----TKEQTETEFSCQSAGYPLAKVMWYHANGTDLNKTANTTHFTGSNGLYNIRSILRIRAETNETYLCMFWIEELNT-STSAVLQI--KE(1)
Bichir PD-L1         SYNNSAAKFFVQRRPENKSEVELICQSQGYPPVKVLWRDSKGRNLTSHAMSNGSFGTNHLFQVSSRLNVPASTLETFSCTFWNESLKN-PFTAFLTI--PD(1)
Reedfish PD-L1       SYNNSAAKFFVMRRPENKSEVELICQSQGYPPVKVLWRDSKGRNLTSHAMSNGFFGTNHLFQVSSRLNVPAPTLETFSCTFWNESLKN-PFTAFLTI--PD(1)
Sturgeon PD-L1       PYKIKTHVKELP-KNADHKDAELSCESEGYPPVQVLWRDGNDQDLSKKASSKHSVTADQLFHISSLLIVNASSNNTYHCILWNETMEE-TYNASLRI--PA(1)
Paddlefish PD-L1     PYKIKTHVKELP-KNADHKDAELSCESEGYPPVKVLWKDGNDQDLSKKATSKHSVTADQLFHISSLLIVSASTNNTYHCILWNETMEE-TYNASLRI--PA(1)
Gar PD-L1            AYTPIKTDI---RRTPDDEKVELTCESEGYPLAKVIWSDASGLNLTSNANESIFLLPDQRYHIKSCVIVNNTVNNTYTCTFWNESMIN--YATNLTI--PD(1)
Bonytongue PD-L1     SYKSVKKTV---RRTGNDE-VELSCGSEGYPLAAFVWSDQSGRRFDELNASSTVTSDKLFHITSS-VIVD-TSTNNYTCTILEENHWG--PSAAFSI--PA(1)
Tarpon PD-L1         SYKSINKSV---RRSGRDE-VELLCQSQGYPQASVVWTDGRGRNLTEEANTTAVISSDQLFHVTSGITVR-TSNNTYTCAFVKEGLMG--QSVTFHI--PE(1)
Weatherfish PD-L1    LNTPIKKSV---RKTEVEDEVELSCEFAGYPLAEVLWSDGQTVNLTEKSSKRTRVTDEDLINITSRLTVRRDLVRNYTCSFLTEGKVR--QTTTFII--PH(1)
Zebrafish PD-L1      PFSPVRKSL---RKA-GEDEVELSCESQGFPSAQVYWSDGQKLNLTLFSNTSVSSTDEDLILIVSKLKVERELVNNYTCTFIVKGEIQ--QTATFSI--PE(1)
Salmon PD-L1         SYKTIVKSM---KRR-RGDEVELVCESEGYPLATMTWKDRS--LRNIKSNDTTVRTPDQLFQVTSKITVKSSDKNNYTCALVEKGEVPKGPSARFDI--PD(1)
Perch PD-L1          PYKTLTKRI---VKAAEGDKVLLTCQSEGYPESPVVWQDGR--LQSLKSNTTTVSTPEQLFKVTSQIHVNSSDKDNYTCHFLNGGQ-----SATFHI--PD(1)
Medaka PD-L1         PYKTVTKRI---ERA-EQNKVLLTCESEGYPKSEVVWTDGN--LQNQHPNTSFVSTPEQLFKITSQILVSSSEENNYTCSFKSDKK-----STTFYI--PD(1)
Mummichog PD-L1      PYKSVSKRI---ERRAEGDKVVLTCHSQGYPKSSVVWHDGH--LQKHNSSTTATATPDGLYNVTSRIEVSSSAKNNYTCNFTHDGY-----SATFHI--PD(1)
```

```
                 Exon-4                                                        Exon-5                 Exon-6
                                         Transmembrane region     Cytoplasmic tail
                                   .230      .240      .250      .260               .270      .280           .290
Human PD-L1                       LPLAHPPNERTHLVILGAILLCLGVALTFI-FRLRKG--------(1)RMMDVKKCGIQDTNSKKQSD--(1)THLEET
Mouse PD-L1                       LPATHPPQNRTHWVLLGSILLFLIVVSTVLLFLRKQV--------(1)RMLDVEKCGVEDTSSKNRND--(1)TQFEET
Cattle PD-L1                      PYLD-PAKKRNHLVTLGALFLCLSVTLAVIFCLKRDV--------(1)RMMDVEKCDTRDMNSKQQNA--(1)TQFEET
Platypus PD-L1                    QTKI-PLMNNRNHLATLTAAILLGSLLLHLFCSKICV--------(1)RVADVEKWDCQSVTCEGQRY--(1)PSTGKT
Chicken PD-L1                     SADD-VLWTESRRFVWPVLIVSALVGSVPITVCIRKA--------(1)RASKDCRTRMAKSSIHITK---(1)LSKDKGAHDCRGPSFEDAELKCKHK
Goose PD-L1                       SADD-VLRTESRRFVGAILVVTALVGSVLFILCIRKA--------(1)STSKDKRTCMANSSVNTAN---(1)IQIEKT
Turtle PD-L1       DTVDSQRINSRHYFATAGAALVLFGSLLLFMLW(0)LEKIRTGRCTNNAAVKVIK--------(1)LSTNEEEGDCREISPENEELEN(1)VRIEVT
Frog PD-L1                        SGKSQSTNKRSFQLSLTAIAVAAALCIFVCVYIKKHD--------(1)LENT
Newt PD-L1                       EIDHTLWPNKRGYYGVILAGSLIVTIMLITVGLSQIYG--------(1)SRSTS
Human PD-L2                      QMEPRTHPTWLLHIFIPFCIIAFIFIATV-IALRKQLCQKLYSSKD(1)TTKRPVTTTKREVNSA(0)I
Mouse PD-L2                      RMEPKVPRTWPLHVFIPACTIALIFLAIV-IIQRKRI
Cattle PD-L2                    PLEDPKIPSSSLLHVFILSLIVAPIFIATV-VALRKRLCQKLYSGED(1)STKRSATMVRREVDRA(0)I
Platypus PD-L2                  PTEVPLAEKTSLFYLFIPTCIIAFIFISAL-IFLRRPLCRELSNRKE(1)NRTRGNGLKLQQEKVRHKEDRGKNFFEC(1)LNL
Chicken PD-L2                   LMSTQYGGQKSLILFIAATCVIVLVLLSVLTIFQKRKSFKNFRAKKD(1)RKGKLSPTVTDENR(1)HSSNSQTETSYLSTVAYSGESRNVSL
Goose PD-L2                     LLSTLYSGQKSLILFIIATCVIVTVLLSALIIFQKRKSFKKCHAKKK(0)-TGKLNPSLTDENR(1)DDYSPQTEAAYFSTATFSRDSGNVSL
Lizard PD-L2                    FANKSNTEKKSLTVMVPICLLLLLVLIIFAFMKLRRKQFTNYYPQKE(1)EDLDLQTKNLNAATIPD
Turtle PD-L2                     RQTSVVGKKSLNLFIIPTCVIAVVLISALIIFLKRKSLTKLHAKKD(1)KKRKCLQLQKEDKR(1)HLNHEIQDLIM-TNVTDSRNCTDVCP
Frog PD-L2                        LTKDQFPMNTGKILTIISVILAVMVTMMVIYIKRKHCFRCFRKKG(1)KRNYSTGSVCETMCPRRNAKHEQDSLPDNPRK
Newt PD-L2                          TILFGKSLQLNLRFMFNLVNVGVVAWTAKVMDGLF
Lungfish PD-L1                 WQIPDSVDGYRLIYVGAAIAIIIVVMIVVTIILIWRKRKD--------(1)WSLLHTGIFQGKKKLPCEYRCKSNCTECWTTLRHLFRSTTHIASHTVLN(1)-KEKSDTACHHHNQGKQLEAS(1)
Shark PD-L1              MEETEEKKDELLSTNRSYLIAAIAIPVTVLLGVIIIVSILLKSSQYRQG--------(1)KIKVCETSLLI
Skate PD-L1              IKRKDNDQIPTFTTMRSYLIVAITIPSIVLLGVIMILICVIKSRLCHKG--------(1)KTYKNEYGLFNA
Bichir PD-L1                   VIQTSGAMETSHLPPLAALFSVLIVTVVTMVVVGVILWKKKHPCQA-----(1)HIRKSHRRMSELHIPINSVQD----(1)
Reedfish PD-L1                 VIQTSGAMETSHLPPLVVPFSVVIVTVVTMVVVGVILWKKKRPCQA-----(1)HIGKSHRRMSELHIPINSVKD----(1)
Sturgeon PD-L1                DIKTVDPPIADGVSSTAYWATVAILLVTIVMVGLIILMWKKKIKCSQLRA--(1)MSQRHPEEENEVHIHINTNED--DG(1)
Paddlefish PD-L1              DIKTVDPAIADGLRSNAYWAAVVIVLMTIITAGVIILMLKKKIKCSQLRA--(1)VLRRYLNEENEAHI----NEE--DV(1)MGKLNH
Gar PD-L1                     DMKKTEEQSPAGRKSSTIVWAI--MVVAAIVVTVAILVNQRFKD--------(1)VKGL-----KRSKRK-EEDFD-ALS(1)TALTINSEVPTSVVTHEEG(1)NLKA
Bonytongue PD-L1                    EIPGTKSGKPPVRVAW-LTLIVVAAMVVAFLFYHRRKG--------(1)LKRNRAPRMKHCLLN-DSHAA--AC(1)LPQGRNDDVQ---VGIED(1)
Tarpon PD-L1                        EIPWKQTGKGSFVVAVVLITVTFAVCVASILIYRRRAG--------(1)YKGRNTAETMDCLIQ-KDPFP--AR(1)MPLETSIEVQ---TSVEE(1)
Weatherfish PD-L1                   EIPASSSHHYVWIGSV--IVVLLVIIFISIIYHKRKNG--------(1)QKDKRSKSSKCPPPFPQT-AANNDC(1)LLTIYENTPHPCDITREE(1)
Zebrafish PD-L1                     EIPLHGSALFVWIGAV--IVVLLAVIFISIILHRRKYG--------(1)QKNRRNEASKCAYLFPQSSSVNTDS(1)LLTVNENRTHAREKSSTE(1)
Salmon PD-L1                      EIDLPAIHSKCNTLSIVLGTSLTVALVIAAAIFGCRRWRG--------(1)RPRDPSTPSTNTLLD
Perch PD-L1                         EIPVPQVKNDALI-VVLSIGLIMVVIIVAVLMYRRRKG--------(1)SNTP--STRNLLVDGRGGPVSAVAC(1)SQTDKENEEERTIF-NEE(1)
Medaka PD-L1                        DLPTPPEKSDAVIIVVLTVLIIVIMAAGGILYY-KRKA--------(1)FRTI--GTTKCLPVDPDSSVSG-AC(1)LQKEKEIKNVEIDM-PED(1)TGLY
Mummichog PD-L1                     DITLPKGKNDALITVLCIGLILTAIGLGVVTYR-RRKG--------(1)ARTP--STRNCLVNDEERSLSAAAC(1)LGMDKESAVEEKVI-TEE(1)
```

```
                     Exon-7 (for lungfish exon 8 + exon 9; for bichir, reedfish, and sturgeon exon 6)
Lungfish PD-L1       QLNKLLEEDSQTRVPVQSSRTTVSVHCEKE(1)P
Bichir PD-L1         PIQNTEDLQELLKERYMN-----HGPEECMTWSEEVPLFGQLASS(2)
Reedfish PD-L1       PIRNTEDLQELLKERYMS-----HAAEGYMTWSEEVPLFGQLANS(2)
Sturgeon PD-L1       SLQQIENLRELLKAKYTASSFSDWAIGEWHSGFCEQFLPQRLVNS(2)
Bonytongue PD-L1      EVEVEGLRKVLMSRYEVLS-TNTEVSN-LRAFCENELPRRLRNR(2)
Tarpon PD-L1         NKGAVEILREVLRRRYATLS-SDAEEAESRRSFCEKELPQRLRNR(2)
Weatherfish PD-L1    HAEKLQTLRDALIQRYSQPI-TDTEMNTRLMSYCSNVLPHVLHLR(2)
Zebrafish PD-L1         KTASLRDSLTQQYSQLY-TESEMKRKLRSYE-------LHSR(2)
Perch PD-L1            RIEENLGAILKAHYSDVS-FSTEVRHHSGSFDVEELPHRLQNN(2)
Mummichog PD-L1           ENLRSHLKAHYSEFS-LTTKTKHHCDSFAAEELPHRLQNN(2)
```

```
                  Exon-8 (for bichir, reedfish, and sturgeon exon 7)
Bichir PD-L1      SGQPQRLQEVLQETDRLHILEEAFQEDQSSLCRALARTWADSNASDPYGVKKY---SLILLLQLTDSVWDFFGELVKQLIH-RDKISTEELQMMLMGAGSILLILDHKGVNSTVSEESLRTILHKYQNLEILLTKHPEGQQIS
Reedfish PD-L1    SSQPQRLQEVLRETERLHVLEEASQEDQSSLCRALARTWADSNASDPYGVKKY---SLIFLLQLTDSVWDIFGELIKQLIP-RDKSSTEELQVMLMGAGSILLILDQNGVNNTASEESLRTILHKYQNLEILLTKHLEGQHIS
Sturgeon PD-L1    DGQTQPLTTLIPQPKGALLLEGDPLSGKSRLCQALAAMWADSTGEDTFGAREC---ELVVLLQCEGSIGNIYEEMIRQLLP-EKRFSEADLEAVLMDHFNTLLILDGYSKGNTTLDESLRSLIREGYSIRILVTSLPEGCEHILDCFGSVLKLQNGVAADIFL
Bonytongue PD-L1  EGLTVGAADLLPGVRETVLLEEENGNDKTAVARSLASAWAGNSQWDPFGVRQL---PLLVLVACEGAVDDLFREAALQVDQ-KSQFTAGDLQKLLTETIDSLLVLDGYREGSRELDESLTTFLSSRKMCRVLITARSGQCDSLRGSCRTMLKLCSASTGNEGLS
Tarpon PD-L1      DGLPLGVTALLPDVGETILLEGEPGSGKTSVAVSLASAWAQNSERDPFGVKQI---DLLILVTCQESRGGLFQEMMSQLSL-GREFTADALREVVTGPVETLLVLDGYKEGNGELDESLRRFLRERQTCRVLVMAQPGQCDNMTECLRTVLMLCSEVKEGQGLC
Weatherfish PD-L1 EGQAVNVSSIFPDKRQTILLLGKPGSGKTTTAQILSSCWAQSLTIDPWNIKDL---QLVVSVNCRGTNGDFFQLVKSNIPN-ETPLDVSDIRETLLGSTDCLVILDGYKEGNRDLDETLGMFLKERQTCRILVMSHPGECPSLENKVGTVLNLIHKSDDKSET
Zebrafish PD-L1   DGHCVNISSVIPEKGQILLLLGDSGCGKTTFTQILSYSWASRSQTDPFNTRRL---RLLLLLHCSQNKGNLNQIINSSVQH-ERPVDV---KQSLKGPEDCLLILDDYQEGNKDLEE----FLKDHQTCRVLITSRPGVCPNLEKTVRTVLHLIHKPEESST
Perch PD-L1       EGQAMKPQALLPEAGEILLLQGPPGSGKTTVAHILVSSWVEGPAHSLANVLDLSILRLLLYIDCSAAKGDLFQEVVTQLSLMEKTSTEDELRTLLTRSSDTLLLLDGYREGNQFFDESLKRFLCERGGCRVLVVTCPEHCPTLKETIGTRGVLKLQTQTVKY
Mummichog PD-L1   EGLPVRLQDLLPNAGEILLLEGPPRSGKTTAAHILLSSWTGADSGFLDAGFL----DLLVYVNCDTMKGDLFQEATAQLALSEKISAEQ-LRTVLSRSNKTLLLLDGYKEGNHFCDETLRRFLSERGSCRVLVTSCLGDCPVLKQTLKTEGTLTLQMQSAKY
```

(B) Phylogenetic tree.

The deduced PD-L1 and PD-L2 amino acid sequences shown in (A) were aligned by ClustalW (Thompson et al., 1994, doi: 10.1093/nar/22.22.4673) in MEGA7 (Kumar et al., 2016, doi: 10.1093/molbev/msw054), after which the cytoplasmic tail regions and extended leader regions were removed by hand. Then, in MEGA7, evolutionary history was inferred by using the Maximum Likelihood method based on the JTT matrix-based model (Jones et al., 1992, doi: 10.1093/bioinformatics/8.3.275). The tree with the highest log likelihood (-10502.97) is shown. The percentage of trees in which the associated taxa clustered together is shown next to the branches. Initial tree(s) for the heuristic search were obtained automatically by applying Neighbor-Join and BioNJ algorithms to a matrix of pairwise distances estimated using a JTT model, and then selecting the topology with superior log likelihood value. The tree is drawn to scale, with branch lengths measured in the number of substitutions per site. The analysis involved 39 amino acid sequences. All positions with less than 95% site coverage were eliminated. That is, fewer than 5% alignment gaps, missing data, and ambiguous bases were allowed at any position. There were a total of 185 positions in the final dataset.

Phylogenetic tree by Maximum Likelihood method

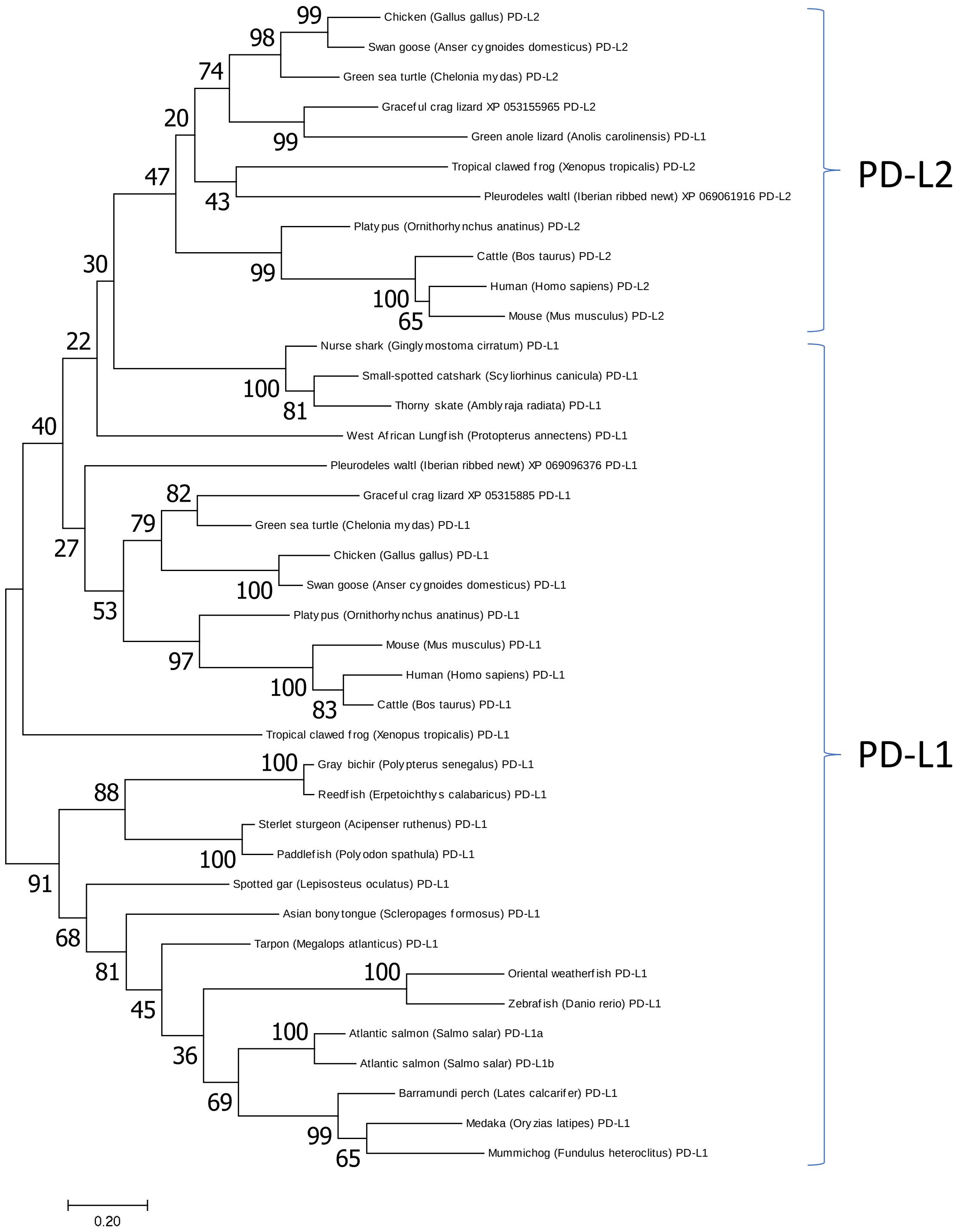

Supplement: Supplementary file 2 [file DataSheet2.pdf]
