# Supplementary material for: PD-1 is conserved from sharks to humans: new insights into PD-1, PD-L1, PD-L2, and SHP-2 evolution
Source: Front Immunol. 2025 May 28;16:1573492. doi: 10.3389/fimmu.2025.1573492 (PMC12151841; doi:10.3389/fimmu.2025.1573492)

Supplementary file 3

Alignment (A) and phylogenetic tree analysis (B) of deduced SHP-1, SHP-2, and SHP-2L amino acid sequences in representative species

(A) Sequence alignment.

Deduced amino acid sequences of Src homology region 2 domain-containing phosphatase-1 (SHP-1), -2 (SHP-2), and -2-like (SHP-2L) in representative species are aligned. All these molecules have two Src homology region 2 (SH2) domains, N-SH2 and C-SH2, a protein tyrosine phosphatase (PTP) domain, and a C-terminus. Region border indications in the figure for the N-SH2, C-SH-2, and PTP domains follow Hof et al., 1998 (doi: 10.1016/s0092-8674(00)80938-1), and the same study is used for identifying residues in the PTP catalytic domain that bind phosphopeptide (gray shaded residues in the PTP domain). The C-termini, encoded by exons 13 and 14 and involved in regulatory functions, are characteristically different between SHP-1, SHP-2, and SHP-2L, whereas the remaining part of the molecules shows only rather subtle differences between SHP-2 and SHP-2L, and more pronounced differences between these two molecules and SHP-1. In almost all SHP-1, SHP-2, and SHP-2L molecules, exon-13 encodes a YxN motif, but this tends to be YGN in SHP-1 at position 536, versus YTN in SHP-2 at 542, and Y(L/S)N in SHP-2L; in SHP-1, phosphorylation of Y536 contributes to SHP-1 activation (Uchida et al., 1994, PMID: 7512963), and phosphorylation of Y542 in SHP-2 can promote the functioning of that protein (Bennett et al., 1994, doi: 10.1073/pnas.91.15.7335). Furthermore, in almost all SHP-1, SHP-2, and SHP-2L molecules, exon-14 encodes a YEN motif (at 564 in SHP-1 and at 580 in SHP-2), whereas the sequences surrounding this motif show characteristic differences between the three types of molecules; phosphorylation of Y564 stimulates SHP-1 activity (Yoshida et al., 1999, doi: 10.1074/jbc.274.49.34663) and phosphorylation of Y580 in SHP-2 can promote the functioning of that protein (Bennett et al., 1994, doi: 10.1073/pnas.91.15.7335). Phosphorylation of S591 of SHP-1 has been described as reducing its phosphatase activity (Liu et al., 2007, doi: 10.1189/jlb.1206736).

Conservation of SHP-2L in eutherian mammals appears to be relatively relaxed, as some mammals like rodents and higher primates lost intact *SHP-2L*, and in the investigated eutherian mammals (cattle, lemur, and tapaia) the available information suggests unusual C-termini and also some other losses of family consensus motifs (in all three species at position 154, and, in tupaia, also the exon-6-encoded stretch and position 433).

Below follow two paragraphs, with the first one containing a more detailed discussion of the SHP-2 binding sites for the PD-1 ITIM and ITSM motifs, and the second one providing general information about the alignment figure.

The SH2 domains of SHP-2 and their binding sites for PD-1 ITIM and ITSM motifs.

SHP-2 residues that were recognized as important for binding the PD-1 cytoplasmic tail ITIM and ITSM stretches (Marasco et al. 2020, doi: 10.1126/sciadv.aay4458; Wang et al. 2020, doi: 10.3389/fchem.2020.597495) are highlighted in yellow shading, and some of these residues are named above the human SHP-2 sequence to help with orientation. Other residues at these

positions—or in residues adjacent to these positions in the amino acid chain—that show characteristic differences between SHP-1, SHP-2, and/or SHP-2L, are highlighted by shading in different colors; we chose to also highlight these adjacent residues as we expect that at least some of them will have an effect on the binding.

For a better understanding of how the different SHP-2 residues highlighted in yellow may contribute to PD-1 binding, we here include direct excerpts from the Marasco et al., 2020, and Wang et al., 2020, articles:

According to Marasco et al., 2020, who experimentally determined the structure of pY-peptides derived from PD-1 ITIM- or ITSM-containing stretches bound to either SH2 domain of SHP-2: "*In all structures* [here referring to N-SH2–ITIM, N-SH2–ITSM, C-SH2-ITSM complexes]*, both ITIM and ITSM adopt the typical extended conformation observed for other phosphopeptides bound to SH2 domains, with the peptide backbone arranged perpendicularly to the central β sheet bundle. The pY moiety is held in place by a network of hydrogen bonds to R32, S34, S36, and T42 of N-SH2 or R138, S140, and S142 of C-SH2. A patch of hydrophobic amino acids provides an interaction surface for the nonpolar C-terminal part of ITSM: In C-SH2–ITSM, residues V170, V181, M202, and L210 surround the ITSM A(+1)–F(+5) stretch; analogously, in N-SH2–ITSM, the peptide C-terminal stretch is in contact with I54, L65, and L88. The packing of the peptide is tighter in C-SH2–ITSM than in N-SH2–ITSM, and C-SH2–ITSM contains two hydrogen bonds from the backbone HN and the side-chain hydroxyl of ITSM-T(+2) to the side-chain carbonyl of C-SH2–E204, which are absent in N-SH2–ITSM.*" Some of those residues with their numbers are indicated above the human SHP-2 sequence to help with orientation. E90 in the SH1 domain corresponds to E204 in the SH2 domain.

According to Wang et al., 2020, who used molecular dynamics simulations (MDS) to analyze all four possible bindings between SHP-2 N-SH2 or C-SH-2 with PD-1 ITIM or ITSM: "*For system N-SH2-ITIM, residues ARG32, SER34, LYS35, SER36, HIS53, LYS55 LYS89 and LYS91 have the largest contribution to the binding due to the electrostatic interaction. Those positive charged residues surround the negative charged PTR, and played a decisive role in the binding. In addition, residues ILE54 and LEU65 also have an important contribution to the binding by van der Waals interaction. For system N-SH2-ITSM, in addition to the above residues, residues GLU17, THR42 and GLU90 also played an important role in the binding. But the contribution of ILE54 and LEU65 to the binding disappeared. For system C-SH2-ITIM, ITIM binds to the C-SH2 domain in the same way that it binds to the N-SH2 domain. Positively charged residues ARG138, ARG173, SER140, SER142, and HIS169 play a major role in the binding. For system C-SH2-ITSM (Figure 3D), there are abundant interactions between ITSM and C-SH2 domain. Residues HIS169, GLY183, ARG186, and THR205 also have key contribution to the binding.*"

General information about the alignment figure.

The sequences here aligned, and their sources, are shown in Supplementary file 1. The species are human (*Homo sapiens*), lemur (Philippine flying lemur; *Cynocephalus volans*), tupaia (*Tupaia chinensis*), mouse (*Mus musculus*), cattle (*Bos taurus*), Platypus (*Ornithorhynchus anatinus*), chicken (*Gallus gallus*), goose (swan goose; *Anser cygnoides domesticus*), lizard (green anole lizard; *Anolis carolinensis*), turtle (green sea turtle; *Chelonia mydas*), frog (tropical clawed frog; *Xenopus tropicalis*), lungfish (West African Lungfish (*Protopterus annectens*), shark (small-spotted catshark; *Scyliorhinus canicula*), skate (Thorny skate; *Amblyraja radiata*), bichir (gray bichir (*Polypterus senegalus*),

reedfish (reedfish; *Erpetoichthys calabaricus*), sturgeon (sterlet sturgeon; *Acipenser ruthenus*), paddlefish (Mississippi paddlefish; *Polyodon spathula*), gar (spotted gar; *Lepisosteus oculatus*), bonytongue (Asian bonytongue; *Scleropages formosus*), tarpon (*Megalops atlanticus*), weatherfish (oriental weatherfish *Misgurnus anguillicaudatus*), zebrafish (*Danio rerio*), salmon (Atlantic salmon (Atlantic salmon; *Salmo salar*; for SHP-2L the SHP-2La form is shown), perch (Barramundi perch; *Lates calcarifer*), medaka (*Oryzias latipes*), mummichog (*Fundulus heteroclitus*). Residue numbering above the alignments follow the human SHP-1 mature protein, and, for some highlighted residues, those of the human SHP-2 mature protein. The numbers between brackets refer to introns and to their phases at the indicated position (0) or in the preceding codon (1,2); intron positions that can only be speculated because genomic DNA sequence information is not available for that species are indicated with "(?)". Cysteines are in purple and, based on Hopp and Woods, 1981 (doi: 10.1073/pnas.78.6.3824): red font is used for basic residues, blue for acidic residues, and of the other residues (green and orange) the more hydrophilic ones are in green. *, the skate SHP-1 sequence is derived from a transcript so the intron positions are only guessed; **, for some molecules the sequence information is not complete.

```
                      Exon-1                                          Exon-2
                       .1           .10          .20          .30          .40          .50          .60          .70          .80          .90         .100
Human SHP-1             MVR(2)WFHRDLSGLDAETLLKGRGVHGSFLARPSRKNQGDFSLSVR(2)VGDQVTHIRIQNSGDFYDLYGGEKFATLTELVEYYTQQQ-GVLQDRDGTIIHLKYPLNCSDPTSER(2)
Lemur SHP-1           MLSHG(2)WFHRDLGGLDAETLLKSRGVHGSFLARPSRKNQGDFSLSVR(2)VGDQVTHIRIQNSGDFYDLYGGEKFATLTELVEYYTQQQ-GVLQDRDGTVIHLKYPLNCSDPTNER(2)
Mouse SHP-1             MVR(2)WFHRDLSGPDAETLLKGRGVPGSFLARPSRKNQGDFSLSVR(2)VDDQVTHIRIQNSGDFYDLYGGEKFATLTELVEYYTQQQ-GILQDRDGTIIHLKYPLNCSDPTSER(2)
Cattle SHP-1            MVR(2)WFHRDLSGLDAETLLKGRGVHGSFLARPSRKNQGDFSLSVR(2)VGDQVTHIRIQNSGDFYDLYGGEKFATLTELVEYYTQQQ-GVLQDRDGTIIHLKYPLNCSDPTSER(2)
Platypus SHP-1           VR(2)WFHRDLSGLDAETLLKGRGVHGSFLARPSRKNQGDFSLSVR(2)VGDQVTHIRIQNSGDFYDLYGGEKFATLTELVEFYTQQQ-GVLQDRDGTIIHLKYPLNCSDPTSER(2)
Chicken SHP-1           MVR(2)WFHRDLSGLEAEALLKGRGVHGSFLARPSRKNQGDFSLSVR(2)VGDQVTHIRIQNTGDFYDLYGGEKFATLSELVEYYTQQQ-GSLQDKDGTIIDLRYPLNCSDPTTER(2)
Goose SHP-1             MVR(2)WFHRDLSGLEAEALLKGRGVHGSFLARPSRKNQGDFSLSVR(2)VGDQVTHIRIQNTGDFYDLYGGEKFATLSELVEYYTQQQ-GSLQDKDGTIIDLRYPLNCSDPTTER(2)
Lizard SHP-1            MSR(2)WFHRDLSGIDAEALLKARGIHGSFLARPSRKNKGDFSLSVR(2)VGDQVTHIRIQNTGDFYDLYGGEKFATLSELVEYYTQQQ-GCLQDKDGTIIDLRYPLNCSDPTTER(2)
Turtle SHP-1            MVR(2)WFHRDLSGLQAEALLKGRGVHGSFLARPSRKNQGDFSLSVR(2)VGDQVTHIRIQNTGDYYDLYGGEKFATLSELVEYYTQQQ-GSLQDKDGTVIDLRYPLNCSDPTTER(2)
Frog SHP-1              MVR(2)WLHQNISGLEAEARLKSRGVHGSFLVRPSKKKPEDFSLSVR(2)VEDSVTHIRIQNTGDYYDLYGGEQFASLSELVEFYTGQA-ECLQDTSGNRIELKYPLNCSDPTNER(2)
Lungfish SHP-1          MVR(2)WFHRDISGIDAETLLKSRGVPGSFLARPSRKNQGDFSLSVR(2)LTDSVTHIRIQNTGDYYDLYGGEKFATLSELVEYYTTQH-GTLQDKDGTLIQLKYPLNCSDPTSER(2)
Shark SHP-1             MVR(2)WFHREISGTEAEKLLKTRGVHGSFLARPSKQNPGDFSLSIR(2)VGEQVTHIRIQNTGDYYDLYGGEKFATLSELVQYYTEQD-NILQDKDGTIIDLKYPLNCSDPTNER(2)
Skate SHP-1*             MR(?)WFHRDISGSQAEKVLKTRGVHGSFLARPSKQNPGDFTLSIR(?)VSDQVTHIRIQNTGDYYDLYGGEKFATLSELVQFYTDQS-NVLQDKDGTVIELKYPLNCSDPTSER(?)
Bichir SHP-1            MVR(2)WFHRDLSGIEAETLLKSRGVHGSFLARPSKKNAGDFSLSVK(2)VGEVVTHIRIQNTGDYYDLYGGEKFATLSELVEYYTTEH-GTLQDKDGTIIELKYPLNCSDPTNER(2)
Reedfish SHP-1          MVR(2)WFHRDLSGIEAETLLKSRGVHGSFLARPSKKNAGDFSLSVK(2)VGEVVTHIRIQNTGDYYDLYGGEKFATLSELVEYYTTEH-GTLQDKDGTIIELKYPLNCSDPTNER(2)
Sturgeon SHP-1**                                                            ELVTHIRIQNTGDFYDLYGGEKFATLSELVEYYTTEH-GTLQDRDGTLIELRYPLNCSEPTSER(2)
Paddlefish SHP-1        MVR(2)WFHRDLSGIEAEALLKSRGIHGSFLCRPSRKNQGDFSLSVR(2)VGEMVTHIRIQNTGDFYDLYGGEKFATLSELVEYYTTEH-GTLQDQDGTLIELRFPLNCSEPTSER(2)
Gar SHP-1              MACG(2)WFHRDLSGIDAEALLKARGVHGSFLARPSKKNQGDFSLSVR(2)VGELVTHIRIQNTGDYYDLYGGEKFATLSELVEYYTGEH-GTLQDKDGTLIELKYPLNCSDPTTER(2)
Bonytongue SHP-1         MR(2)WFHRDLSGLEAEALLKARGVHGSFLARPSKKNQGDFSLSVR(2)VGDMVTHIRIQNTGDFYDLYGGEKFATLSELVEYYTTEH-GTLQDKDGTVIELKYPLNSSDPTTER(2)
Tarpon SHP-1            MVR(2)WFHRDLSGLEAEAMLKARGVHGSFLARPSKKNQGDFSLSVR(2)VGEMVTHIRIQNTGDYYDLYGGEKFATLSELVEYYTVEH-GTLQDKDGTIIELKYPFNCSDPTTER(2)
Weatherfish SHP-1       MVR(2)WFHRDLSGLEAEAVLKTRGFHGSFLARPSKKNVGDFSLSVR(2)VGNMITHIRIQNTGDYYDLYGGEKFATLAELVEYYTADH-GVLQDKDGTVIELKYPLNCSDPTTER(2)
Zebrafish SHP-1         MVR(2)WFHRDLSGLDAEAVLKSRGVHGSFLARPSKKNVGDFSLSVR(2)VGEIITHIRIQNTGDYYDLYGGEKFATLAELVEYYTGDH-GTLQDKDGTVIELKYPLNCSDPTTER(2)
Salmon SHP-1            MVR(2)WFHRDISGLDAESVLKSRGIHGSFLARPSRKNQGDFSLSVR(2)VGELVTHIRIQNTGDFYDLYGGEKFATLSELVEYYTAEN-GILQDKDGTIIELKYPFNCSDPTTER(2)
Perch SHP-1             MVR(2)WFHRDITGLQAEEMLKSRGIHGSFLARPSKKNVGDFSLSVR(2)VGELVTHIRIQNTGDFYDLYGGEKFATLSELVDYYTAEN-GILQDRDGTVIELKYPLNCSDPTTER(2)
Medaka SHP-1            MVR(2)WFHRDITGLQAEEILKLRGIDGSFLARPSKKNVGDFSLSVR(2)VGDQVTHIRIQNTGDYYDLYGGEKFATLSELVDYYTSDS-GTLQDKDGTLIQLKYPLNCSDPTTER(2)
Mummichog SHP-1         MVR(2)WFHRDISGLEAEDILKSRGIHGSFLARPSKKNVGDFSLSVR(2)VGDGVTHIRIQNTGDFYDLYGGEKFATLSELVEYYTAEN-GILQDKDGTIIELKYPVNCSDPTTER(2)
                         <                                        N-SH2 domain                                              >
                                    .E17                .R32        .T42              .I54          .L65                       .E90
Human SHP-2           MTSRR(2)WFHPNITGVEAENLLLTRGVDGSFLARPSKSNPGDFTLSVR(2)RNGAVTHIKIQNTGDYYDLYGGEKFATLAELVQYYMEHH-GQLKEKNGDVIELKYPLNCADPTSER(2)
Lemur SHP-2           MTSRR(2)WFHPNITGVEAENLLLTRGVDGSFLARPSKSNPGDFTLSVR(2)RNGAVTHIKIQNTGDYYDLYGGEKFATLAELVQYYMEHH-GQLKEKNGDVIELKYPLNCADPTSER(2)
Tupaia SHP-2          MTSRR(2)WFHPNITGVEAENLLLTRGVDGSFLARPSKSNPGDFTLSVR(2)RNGAVTHIKIQNTGDYYDLYGGEKFATLAELVQYYMEHH-GQLKEKNGDVIELKYPLNCADPTSER(2)
Mouse SHP-2           MTSRR(2)WFHPNITGVEAENLLLTRGVDGSFLARPSKSNPGDFTLSVR(2)RNGAVTHIKIQNTGDYYDLYGGEKFATLAELVQYYMEHH-GQLKEKNGDVIELKYPLNCADPTSER(2)
Cattle SHP-2          MTSRR(2)WFHPNITGVEAENLLLTRGVDGSFLARPSKSNPGDFTLSVR(2)RNGAVTHIKIQNTGDYYDLYGGEKFATLAELVQYYMEHH-GQLKEKNGDVIELKYPLNCADPTSER(2)
Platypus SHP-2        MTSRR(2)WFHPNITGVEAENLLLTRGVDGSFLARPSKSNPGDFTLSVR(2)RNGAVTHIKIQNTGDYYDLYGGEKFATLAELVQYYMEHH-GQLKEKNGDVIELKYPLNCADPTSER(2)
Chicken SHP-2         MTSRR(2)WFHPNITGVEAENLLLTRGVDGSFLARPSKSNPGDFTLSVR(2)RTGAVTHIKIQNTGDYYDLYGGEKFATLAELVQYYMEHH-GQLKEKNGDVIELKYPLNCADPTSER(2)
Goose SHP-2**                 WFHPNITGVEAENLLLTRGVDGSFLARPSKSNPGDFTLSVR(2)RTGAVTHIKIQNTGDYYDLYGGEKFATLAELVQYYMEHH-GQLKEKNGDVIELKYPLNCADPTSER(2)
Lizard SHP-2          MTSRR(2)WFHPNITGVEAENLLLTRGVDGSFLARPSKSNPGDFTLSVR(2)RNGAVTHIKIQNTGDYYDLYGGEKFATLAELVQYYMEHH-GQLKEKNGDVIELKYPLNCADPTSER(2)
Turtle SHP-2          MTSRR(2)WFHPNITGVEAENLLLTRGVDGSFLARPSKSNPGDFTLSVR(2)RNGAVTHIKIQNTGDYYDLYGGEKFATLAELVQYYMEHH-GQLKEKNGDVIELKYPLNCADPTSER(2)
Frog SHP-2            MTSRR(2)WFHPNITGVEAENLLLTRGVDGSFLARPSKSNPGDFTLSVR(2)RNGAVTHIKIQNTGDYYDLYGGEKFATLAELVQYYMEHH-GQLKEKNGDVIELKYPLNCADPTSER(2)
Lungfish SHP-2        MTSRR(2)WFHPNITGVEAENLLLTRGVDGSFLARPSKSNPGDFTLSVR(2)RNAAVTHIKIQNTGDYYDLYGGEKFATLAELVQFYMEHH-GQLKEKNGDVIELKYPLNCADPTSER(2)
Shark SHP-2           MTSRR(2)WFHPNITGVEAENLLLTRGVDGSFLARPSKSNPGDFTLSVR(2)RNGAVTHIKIQNTGDYYDLYGGEKFATLAELVQYYMEHH-GQLKEKNGDVIELKYPLNCADPTSER(2)
Skate SHP-2           MTSRR(2)WFHPNITGVEAENLLLTRGVDGSFLARPSKSNPGDFTLSVR(2)RNGAVTHIKIQNTGDYYDLYGGEKFATLAELVQYYMEHH-GQLKEKNGDVIELKYPLNCADPTSER(2)
Bichir SHP-2          MTSRR(2)WFHPNITGVEAENLLLTRGVDGSFLARPSKSNPGDFTLSVR(2)RNGAVTHIKIQNTGDYYDLYGGEKFATLAELVQYYMEHH-GQLKEKNGDVIELKYPLNCADPTSER(2)
Reedfish SHP-2        MTSRR(2)WFHPNITGVEAENLLLTRGVDGSFLARPSKSNPGDFTLSVR(2)RNGAVTHIKIQNTGDYYDLYGGEKFATLAELVQYYMEHH-GQLKEKNGDVIELKYPLNCADPTSER(2)
Sturgeon SHP-2        MTSRR(2)WFHPNITGVEAENLLLTRGVDGSFLARPSKSNPGDFTLSVR(2)RNGAVTHIKIQNTGDYYDLYGGEKFATLAELVQYYMEHH-GQLKEKNGDVIELKYPLNCADPTSER(2)
Paddlefish SHP-2      MTSRR(2)WFHPNITGVEAENLLLTRGVDGSFLARPSKSNPGDFTLSVR(2)RNGAVTHIKIQNTGDYYDLYGGEKFATLAELVQYYMEHH-GQLKEKNGDVIELKYPLNCADPTSER(2)
Gar SHP-2             MTSRR(2)WFHPNITGVEAENLLLTRGVDGSFLARPSKSNPGDFTLSVR(2)RNGAVTHIKIQNTGDYYDLYGGEKFATLAELVQYYMEHH-GQLKEKNGDVIELKYPLNCADPTSER(2)
Bonytongue SHP-2      MTSRR(2)WFHPNITGVEAENLLLTRGVDGSFLARPSKSNPGDFTLSVR(2)RNGAVTHIKIQNTGDYYDLYGGEKFATLAELVQYYMEHH-GQLKEKNGDVIELKYPLNCADPTSER(2)
Tarpon SHP-2          MTSRR(2)WFHPNITGVEAENLLLTRGVDGSFLARPSKSNPGDFTLSVR(2)RNGAVTHIKIQNTGDYYDLYGGEKFATLAELVQYYMEHH-GQLKEKNGDVIELKYPLNCADPTSER(2)
Weatherfish SHP-2     MTSRR(2)WFHPNITGVEAENLLLTRGVDGSFLARPSKSNPGDFTLSVR(2)RNGAVTHIKIQNTGDYYDLYGGEKFATLAELVQYYMEHH-GQLKEKNGDVIELKYPLNCADPTSER(2)
Zebrafish SHP-2       MTSRR(2)WFHPNITGVEAENLLLTRGVDGSFLARPSKSNPGDFTLSVR(2)RNGAVTHIKIQNTGDYYDLYGGEKFATLAELVQYYMEHH-GQLKEKNGDVIELKYPLNCADPTSER(2)
Salmon SHP-2          MTSRR(2)WFHPNITGVEAENLLLTRGVDGSFLARPSKSNPGDFTLSVR(2)RNGAVTHIKIQNTGDYYDLYGGEKFATLAELVQYYMEHH-GQLKEKNGDVIELKYPLNCADPTSER(2)
Perch SHP-2           MTSRR(2)WFHPNITGVEAENLLLTRGVDGSFLARPSKSNPGDFTLSVR(2)RNGAVTHIKIQNTGDYYDLYGGEKFATLAELVQYYMEHH-GQLKEKNGDVIELKYPLNCADPTSER(2)
Medaka SHP-2          MTSRR(2)WFHPNITGVEAENLLLTRGVDGSFLARPSKSNPGDFTLSVR(2)RNGAVTHIKIQNTGDFYDLYGGEKFATLAELVQYYMEHH-GQLKEKNGDVIELKYPLNCADPTSER(2)
Mummichog SHP-2       MTSRR(2)WFHPNISGVEAENLLLTRGVDGSFLARPSKSNPGDFTLSVR(2)RNGAVTHIKIQNTGDYYDLYGGEKFATLAELVQYYMEHH-GQLKEKNGDVIELKYPLNCADPTSER(2)
Lemur SHP-2L          MTSRR(2)WFHPNISGVEAEKLLLSRGQHGSFLARPSKSSPGGFTLSVR(2)RHEEVTHIKIQNTGDYYDLYGGEKFATLAELVQHYTGRHGGPLRERSGAPVELRHPLGCQDPTSER(2)
Tupaia SHP-2L         MTSRR(2)WFHPNISGIEAEKLLLSRGQHGSFLARPSKSSPGGFTLSVR(2)RHEEVTHIKIQNTGDYYDLYGGEKFATLAELVQHYTGQRGGLLRERSGAPVELRHPLGCQDPTSER(2)
Cattle SHP-2L         MTSRR(2)WFHPNISGVEAEQLLMSSGQHGSFLARPSKSCPGGFTLSVR(2)RHNEVTHIKIQNTGDYYDLYGGEKFATLAELVQHYTGQHGGLLRERGGAPVELRHPLGCQDPTSER(2)
Platypus SHP-2L       MTSRR(2)WFHPNINGIEAEKLLLTRGGHGSFLARPSKSSLGDFTLSVR(2)RNEEVTHIKIQNTGDYYDLYGGEKFATLAELVQYYTEQQ-GLLREKNGDVIELKFPLNCQDPTSER(2)
Chicken SHP-2L          MVR(2)WFHPNISGIEAEKLLLTRGVHGSFLARPSKSNPGDFTLSVR(2)RNDEVTHIKIQNTGDYYDLYGGEKFATLAELVQYYTEQQ-GLLREKNSNVIELKYPLNCQDPTSER(2)
Goose SHP-2L            MVR(2)WFHPNISGIEAEKLLLTRGVHGSFLARPSKSNPGDFTLSVR(2)RNDEVTHIKIQNTGDYYDLYGGEKFATLAELVQYYTEQQ-GLLREKNSNVIELKYPLNCQDPTSER(2)
Lizard SHP-2L            MR(2)WFHPNINGIEAEKLLLTRGVHGSFLARPSKSNPGDFTLSVR(2)RNDEVTHIKIQNTGDYYDLYGGEKFATLSELVQYYTEQQ-GLLREKNSNIIELKFPLNCQDPTSER(2)
Turtle SHP-2L           MVR(2)WFHPNINGIEAEKLLLTRGVHGSFLARPSKSNPGDFTLSVR(2)RNDEVTHIKIQNTGDYYDLYGGEKFATLAELVQYYTEQQ-GLLREKNSNVIELKYPLNCQDPTSER(2)
Frog SHP-2L           MTSRR(2)WFHPNISGQEAETLLLTNGGHGSFLARPSKSNPGDFTLSVR(2)RHNEVTHIKIQNTGDYYDLYGGETFATLAELVQYYTEQQ-GLLREKNGDVIELKYPLNSQDPTSER(2)
Lungfish SHP-2**              WFHPNITGVEAEQVLLTRGVHGSFLARPSKSNPGDFTLSVR(2)RNDEVTHIKIQNTGDFYDLYGGEKFATLAELVQYYTEQQ-GLLREKNGDVIELKYPLNCKDPTSER(2)
Shark SHP-2L          MTSRR(2)WFHPNITGIEAERLLLTRGVHGNFLARPSKSNPGDFTLSVR(2)RNEEVTHIKIQNTGDYYDLYGGEKFATLAELVQYYTEQQ-GLLREKNGDIIELKYPLNCQDPTSER(2)
Skate SHP-2L          MTSRR(2)WFHPNITGIEAEQVLLCRGVHGSFLARPSKSNPGDFTLSIR(2)RNVEVTHIKIQNSGDYYDLYGGEKFATLAELVQHYTEQE-GLLREKNGDVIELKYPLNCRDPTSER(2)
Bichir SHP-2L         MTSRR(2)WFHPNISGIEAEELLLTRGVHGSFLARPSKSNPGDFTLSVR(2)RGDEVTHIKIQNSGDYYDLYGGEKFATLAELVQYYTEQQ-DLLREKNGDVIELKYPLNCKDPTSER(2)
Reedfish SHP-2L       MTSRR(2)WFHPNISGIDAEELLLTRGVHGSFLARPSKSNPGDFTLSVR(2)RSDEVTHIKIQNSGDYYDLYGGEKFATLAELVQYYTEQQ-DLLREKNGDVIELKYPLNCKDPTSER(2)
Sturgeon SHP-2L       MTSRR(2)WFHPNITGIEAEQLLLTRGVHGSFLARPSKSNPGDFTLSVR(2)RNDEVTHIKIQNSGDYYDLYGGEKFATLAELVQYYTEQQ-DLLREKNGDVIELKYPLNCKDPTSER(2)
Paddlefish SHP-2L     MTSRR(2)WFHPNITGIEAEQLLLTRGVHGSFLARPSKSNPGDFTLSVR(2)RNDEVTHIKIQNSGDYYDLYGGEKFATLAELVQYYTEQQ-DLLREKNGDVIELKYPLNCKDPTSER(2)
Gar SHP-2L            MTSRR(2)WFHPNITGIEAEQLLLTRGVHGSFLARPSKSNPGDFTLSVR(2)RNDEVTHIKIQNSGDYYDLYGGEKFATLAELVQYYTEQH-DLLREKSGDIIELKYPLNCKDPTSER(2)
Bonytongue SHP-2L     MMSRR(2)WFHPNITGVEAEQLLLTRGVHGSFLARPSKSNPGDFTLSVR(2)RKNEVTHIKIQNSGDYYDLYGGEKFATLAELVQYYTEQH-DLLRERNGDVIELKYPLNCKDPTSER(2)
Tarpon SHP-2L         MTSRR(2)WFHPNITGMEAEQLLLARGVHGSFLARPSKSNPGDFTLSVR(2)RNDEVTHIKIQNSGDYYDLYGGEKFATLAELVQYYTEQH-DLLRERNGDVIELKYPLNCKDPTSER(2)
Weatherfish SHP-2L      MVR(2)WFHPNITGIEAEHLLLTRGVHGSFLARPSKSNPGDFTLSVR(2)RNDEVTHIKIQNSGDYYDLYGGEKFATLAELVQYYTEQH-DSLRERNGDVIELKYPLNCKDPTSER(2)
Zebrafish SHP-2L        MVR(2)WFHPNITGIEAEHLLLTRGVHGSFLARPSKSNPGDFTLSVR(2)RNDEVTHIKIQNSGDYYDLYGGEKFATLAELVQYYTEQH-DLLRERNGDVIELKYPLNCKDPTSER(2)
Salmon SHP-2L           MVR(2)WFHPNITGIEAEQLLLTRGVHGSFLARPSKSNPGDFTLSVR(2)RSDEVTHIKIQNSGDYYDLYGGEKFATLAELVQYYTEQH-DLLRERNGDVIELKYPLNCKDPTSER(2)
Perch SHP-2L            MVR(2)WFHPNITGIEAEQLLLTRGVHGSFLARPSKSNPGDFTLSVR(2)RNDEVTHIKIQNSGDYYDLYGGEKFATLAELVQYYTEQQ-DLLRERNGHVIELKYPLNCKDPTSER(2)
Medaka SHP-2L         MTSRR(2)WFHPNIIGVEAEQLLLTRGVHGSFLARPSKSNPGDFTLSVR(2)RSDEVTHIKIQNSGDYYDLYGGEKFATLAELVQYYTEQQ-DLLRERNGHVIELKYPLNCKDPTSER(2)
Mummichog SHP-2L        MVR(2)WFHPNITGIEAEQLLLTRGVHGSFLARPSKSNPGDFTLSVR(2)RNDEVTHIKIQNSGDYYDLYGGEKFATLAELVQYYTEQQ-DLLRERNGHVIELKYPLNCKDPTSER(2)
```

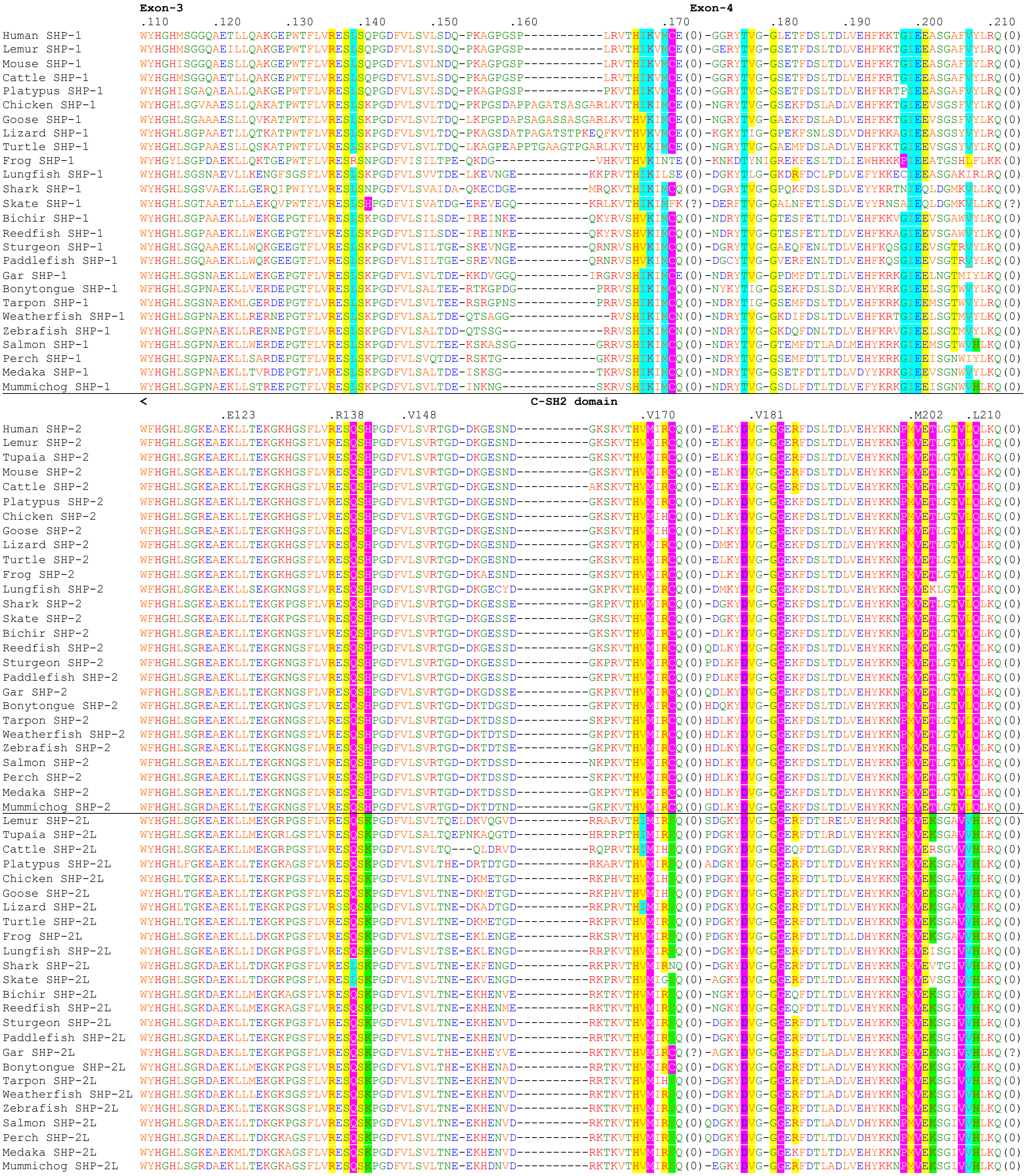
Exon-3
Exon-4
C-SH2 domain
.E123
.R138
.V148
.V170
.V181
.M202
.L210

```
                  Exon-5                                          Exon-6                                    Exon-7
                          .220      .230      .240                     .250      .260      .270          .280        .290      .300
Human SHP-1        PYYATRVNAADIENRVLELNKKQESEDTA------KAGFWEEFE(0)SLQKQEVKNLHQRLEGQRPENKGK----NRYKNILPF(1)DHSRVILQGRDSNIPGSDYINANYIK(0)
Lemur SHP-1        PYYATRVNAADIENRVLELNKKQESEDTA------KAGFWEEFE(0)SLQKQEVKNLHQRLEGQRPENKSK----NRYKNILPF(1)DHSRVILQGRDSNSPGSDYINANYVK(0)
Mouse SHP-1        PYYATRVNAADIENRVLELNKKQESEDTA------KAGFWEEFE(0)SLQKQEVKNLHQRLEGQRPENKSK----NRYKNILPF(1)DHSRVILQGRDSNIPGSDYINANYVK(0)
Cattle SHP-1       PYYATRVNAADIENRVLELNKKQESEETA------KAGFWEEFE(0)SLQKQEVKNLHQRLEGQRPENKSK----NRYKNILPF(1)DHSRVVLQGRDSNIPGSDYINANYVK(0)
Platypus SHP-1     PYYATRVNAADIENRVLELNKKHEAQETA------KAGFWEEFE(0)SLQKQEVKNLYERQEGQRPENKGK----NRYKNILPF(1)DHTRVVLQGRDSSIPGADYINANYVK(0)
Chicken SHP-1      PYYATRVNAADIENRVHELNKKSLSEETS------KAGFWEEFD(0)SLQKQEAKQLFDRHEGQRPENKSK----NRYKNILPF(1)DHSRVILQGRDPNIPGSDYINANYVK(0)
Goose SHP-1        PYYATRVNAADIENRVHELNKKSVSEETS------KAGFWEEFD(0)SLQKQEAKQLFDRHEGQRPENKSK----NRYKNILPF(1)DHSRVILQGRDPNIPGSDYINANYVK(0)
Lizard SHP-1       PFNATRVNAADIEDRVQMLNKRSQIEEAA------KGGFWEEFD(0)SLQKQETKNLHERNEGQRPENKCK----NRYKNILPF(1)DHSRVVLQDRDGNVAGSDYINANYIK(0)
Turtle SHP-1       PYYATRVNAADIENRVHELNKQSLAEETA------KAGFWEEFD(0)SLQKQEAKHLYDRHEGQRPENKGK----NRYKNILPF(1)DHSRVILQGRDPNIPGADYINANYIK(0)
Frog SHP-1         PCYSTRVNAADIENRMKELNKKSEQDEAA------KAGFWEEFD(0)ALQKQENKVHNDRKEGQRPENKSK----NRYKNILPY(1)DHTRVILKCGDNNTPGSDYINANYVN(0)
Lungfish SHP-1     PCYATRINAADIESRVQVLAKSSEDTESA------KAGFWEEFD(0)RLQKQETKNLLSRAEGQRPENKTK----NRYKNILPF(1)DETRVILKDRDPNVLGSDYINANYVE(0)
Shark SHP-1        PYHATRINAAEIQNRVEQLNK-TESPELV------KGGFWEEFD(0)ALQKQDDTKNQSRTEGQRPENKSK----NRYKNILPF(1)DSTRVILHTQDNSPPFSDYINANYIQ(0)
Skate SHP-1        PYHATRINAADIENRVEQLNK-PHSKEGA------KAGFWEEFD(?)ALQKQDDLKTLSREEGQRPENKSK----NRYKNILPF(?)DKTRVILKTTPDSPPFTDYINANYIQ(?)
Bichir SHP-1       PYYSTRVNAGDIDKRVQILA---ETGEVA-N----KAGFWEEFD(0)ALQKQEAKVKKSREEGMRPENKSK----NRYKNILPF(1)NETRVILKSGDPNIIGSDYINANYVK(0)
Reedfish SHP-1     PYYSTRVNAGDIDKRVQILA---ETGEVA-N----KAGFWEEFD(0)ALQKQEAKVKKSREEGMRPENKSK----NRYKNILPF(1)NDTRVSLESGDPNIIGSDYINANYVK(0)
Sturgeon SHP-1     PYYSTRINAADIDSRVQVLTKTNDTNEASKS----KAGFWEEFD(0)ALQKQEAKLKKSRNEGMRPENKSK----NRYKNILPF(1)DETRVILQSGDPNVVGSDYINANYIS(0)
Paddlefish SHP-1   PYYSTRINAADIDSRVQVLTKTNDADEASKS----KAGFWEEFD(0)ALQKQEAKLKKSRNEGMRPENKSK----NRYKNILPF(1)DETRVILQSGDPNVIGSDYINANYVS(0)
Gar SHP-1          PYYSTRVNAADIDSRVQVLEQTAGREDAQSSN---KAGFWEEFD(0)ALQKQEAKLRKSRDEGMRPENKSK----NRYKNILPF(1)NDTRVVLESGDPNVIGSDYINANYVK(0)
Bonytongue SHP-1   PYYSTRVNAADIENRVKVLDQTAARENGGEGEKKSKAGFWEEFD(0)ALQKHETKVKKSRSEGMRPENKSK----NRYKNILPF(1)DDTRVILQSADPSVVGSDYINGNYVK(0)
Tarpon SHP-1       PYYSTRVNAADIDSRVRVLDKTTDRDEEGGGEKKSKAGFWEEFD(0)YLQKQEAKVKKSRDEGMRPENKSK----NRYKNILPF(1)DETRVALQSGDPSIIGSDYINANYVK(0)
Weatherfish SHP-1  PYYSTRLNAADIESRVKQLDQTSEREEKEGSDKKIKAGFWEEFD(0)ALQKLETKVTKSRDEGMRPENKSK----NRYKNILPF(1)DETRVILANADPNVVGSDYINANYVI(0)
Zebrafish SHP-1    PYYSTRLNAADIQSRVNQLDQTSEREKMDGADKKIKAGFWEEFD(0)ALQKLETKVTKSRDEGMRPENKSK----NRYKNILPF(1)DETRVILENADPNVVGSDYINANYVI(0)
Salmon SHP-1       PYFSTRVNAADIDSRVRLLDQTAEREN-EG-DKKSKAGFWEEFD(0)ALQKQETKVKKSREEGMRPENKSK----NRYKNILPF(1)DETRVILSSGDPNIIGSDYINANYVT(0)
Perch SHP-1        PYYSTRVNAADIDNRVKELDQTKQQQEGEG-E-KSKAGFWEEFD(0)ALQKLEAKVKKSREEGQRPENKSK----NRYKNILPF(1)NDTRVILQDADPNVVGSDYINANYVK(0)
Medaka SHP-1       PYYSTRVNAADIDSRVRQLDQTPQEQLEEEGQ-KSKAGFWEEFD(0)ALQKLEAKVKKSREEGQRPENKSK----NRYKNILPF(1)NDTRVILQDTDPNVVGSDYINANYVK(0)
Mummichog SHP-1    PYFSTRVNAADIDSRVRQLDLTSDKPVESEGE-KSKAGFWEEFD(0)ALQKMEAKVKKSREEGQRPENKSK----NRYKNILPF(1)NDTRVILQNADPNVVGSDYINANYVK(0)
C-SH2-domain (continued)  >    <                                      PTP-domain
Human SHP-2        PLNTTRINAAEIESRVRELSKLAETTDKV------KQGFWEEFE(0)TLQQQECKLLYSRKEGQRQENKNK----NRYKNILPF(1)DHTRVVLHDGDPNEPVSDYINANIIM(0)
Lemur SHP-2        PLNTTRINAAEIESRVRELSKLAETTDKV------KQGFWEEFE(0)TLQQQECKLLYSRKEGQRQENKNK----NRYKNILPF(1)DHTRVVLHDGDPNEPVSDYINANIIM(0)
Tupaia SHP-2       PLNTTRINAAEIESRVRELSKLAETTDKV------KQGFWEEFE(0)TLQQQECKLLYSRKEGQRQENKNK----NRYKNILPF(1)DHTRVVLHDGDPNEPVSDYINANIIM(0)
Mouse SHP-2        PLNTTRINAAEIESRVRELSKLAETTDKV------KQGFWEEFE(0)TLQQQECKLLYSRKEGQRQENKNK----NRYKNILPF(1)DHTRVVLHDGDPNEPVSDYINANIIM(0)
Cattle SHP-2       PLNTTRINAAEIESRVRELSKLAETTDKV------KQGFWEEFE(0)TLQQQECKLLYSRKEGQRQENKNK----NRYKNILPF(1)DHTRVVLHDGDPNEPVSDYINANIIM(0)
Platypus SHP-2     PLNTTRINAAEIESRVRELSKLAETTDKV------KQGFWEEFE(0)TLQQQECKLLYSRKEGQRQENKNK----NRYKNILPF(1)DHTRVVLHDGDPNEPVSDYINANIIM(0)
Chicken SHP-2      PLNTTRINAAEIESRVRELSKLAETTDKV------KQGFWEEFE(0)TLQQQECKLLYSRKEGQRQENKNK----NRYKNILPF(1)DHTRVVLHDGDPNEPVSDYINANIIM(0)
Goose SHP-2        PLNTTRINAAEIESRVRELSKLAETTDKV------KQGFWEEFE(0)TLQQQECKLLYSRKEGQRQENKNK----NRYKNILPF(1)DHTRVVLHDGDPNEPVSDYINANIIM(0)
Lizard SHP-2       PLNTTRINAAEIESRVRELSKLAETTDKV------KQGFWEEFE(0)TLQQQECKLLYSRKEGQRQENKNK----NRYKNILPF(1)DHTRVVLHDGDPNEPVSDYINANIIM(0)
Turtle SHP-2       PLNTTRINAAEIESRVRELSKLAETTDKV------KQGFWEEFE(0)TLQQQECKLLYSRKEGQRQESKNK----NRYKNILPF(1)DHTRVVLHDGDPNEPVSDYINANIIM(0)
Frog SHP-2         PLNTTRINAAEIESRVRELSKPAETADKF------KQGFWEEFE(0)TLQQQECKLLYSRKEGQRQENKNK----NRYKNILPF(1)DHTRVELHDGDPNEQVSDYINANIIM(0)
Lungfish SHP-2     PLNTTRINAAEIESRVKELSKLAETTDKV------KQGFWEEFE(0)TLQQQECKLLYSRKEGQRPENKNK----NRYKNILPF(1)DHTRVVLHDGDPNEPVSDYINANIIT(0)
Shark SHP-2        PLNTTRINAAEIESRVRELSKPAEMMDKV------KQGFWEEFE(0)TLQQQECKLLYSRKEGQRPENKNK----NRYKNILPF(1)DHTRVVLHDGDVNESGSDYINANFIT(0)
Skate SHP-2        PLNTTRINAAEIESRVRELSKPAEMMDKV------KQGFWEEFE(0)TLQQQECKLLYSRKDGQRQENKNK----NRYKNILPF(1)DHTRVVLHDGDLTEAGSDYINANFIT(0)
Bichir SHP-2       PLNTTRINAAEIESRVRELSKLAEATDKF------KQGFWEEFE(0)TLQQQECKLLYSRKEGQRPENKNK----NRYKNILPF(1)DHTRVVLNDGDPNEPGSDYINANKIM(0)
Reedfish SHP-2     PLNTTRINAAEIESRVRELSKLAEATDKF------KQGFWEEFE(0)TLQQQECKLLYSRKEGQRPENKNK----NRYKNILPF(1)DHTRVVLNDGDPNEPGSDYINANKIM(0)
Sturgeon SHP-2     PLNTTRINAAEIESRVRELSKLAETTDKV------KQGFWEEFE(0)TLQQQECKLLYSRKEGQRAENKNK----NRYKNILPF(1)DHTRVMLNDKEINELGSDYINANIIM(0)
Paddlefish SHP-2   PLNTTRINAAEIESRVRELSKLAETTDKV------KQGFWEEFE(0)TLQQQECKLLYSRKEGQRAENKNK----NRYKNILPF(1)DHTRVMLNDKEINELGSDYINANIIM(0)
Gar SHP-2          PLNTTRINAAEIESRVKELSKLAEATDKV------KQGFWEEFE(0)TLQQQECKLLYSRKEGQRAENKNK----NRYKNILPF(1)DHTRVVLNDGDVNEPGSDYINANIIM(0)
Bonytongue SHP-2   PLNTTRINAAEIESRVRELSKLAETTDKV------KQGFWEEFE(0)TLQQQECKLLYSRKEGQRPENKNK----NRYKNILPF(1)DHTRVVLRDGDPNEPGSDYINANIIM(0)
Tarpon SHP-2       PLNTTRINAAEIESRVRELSKLAEATDKV------KQGFWEEFE(0)TLQQQECKLLYSRKEGQRAENKNK----NRYKNILPF(1)DHTRVVLNDGDTNEPGSDYINANIIM(0)
Weatherfish SHP-2  PLNTTRINAAEIESRVRELSKLAEATDKV------KQGFWEEFE(0)TLQQQECKLLYSRKEGQRPENKNK----NRYKNILPF(1)DHTRVMLIDGDANEPGSDYINANIIE(0)
Zebrafish SHP-2    PLNTTRINAAEIESRVRELSKLAEATDKV------KQGFWEEFE(0)TLQQQECKLLYSRKEGQRPENKNK----NRYKNILPF(1)DHTRVVLTDGDVNEQGSDYINANLIM(0)
Salmon SHP-2       PLNTTRINAAEIDSRVRELSKLAEATDKV------KQGFWEEFE(0)TLQQQECKLLYSRKEGQRAENKNK----NRYKNILPF(1)DHTRVVLNDGDGSEAGSDYINANLIM(0)
Perch SHP-2        PLNTTRINAAEIESRVRELSKLAEATDKV------KQGFWEEFE(0)TLQQQECKLLYSRKEGQRAENKNK----NRYKNILPF(1)DHTRVVLNDGDLNEPGSDYINANIIM(0)
Medaka SHP-2       PLNTTRINAAEIESRVRELSKLAEATDKV------KQGFWEEFE(0)TLQQQECKLLYSRKEGQRAENKNK----NRYKNILPF(1)DHTRVVLNDGDPNEPGSDYINANIIM(0)
Mummichog SHP-2    PLNTTRINAAEIESRVRELSKLAEATDKV------KQGFWEEFE(0)TLQQQECKLLYSRKEGQRPENKNK----NRYKNILPF(1)DHSRVVLDDGDPSEAGSDYINANIIM(0)
Lemur SHP-2L       PLKATRITAESIESRVRELSAAAAASE-A------KQGFWEEFE(0)MLQQQECRLLYPRKEGQRPENKPK----NRYKNILPF(1)DTTRVILRDVDDSVPGADYINANYIR(0)
Tupaia SHP-2L      PLKATRINADSIESRVQELAQAADTSGKA------TQGFWEEFE---VPSACCHPHTQTSGARAQEESERGR(0)NRYKNILPF(1)DTTRVVLHDVDHGTPGADYINANYIR(0)
Cattle SHP-2L      PLKATRISAASIESRVQELSEATDASEKA------KQGFWEEFE(0)MLQQQECRLLYPRKEGQRPENKPK----NRYKNILPF(1)DTTRVILHDVDDRVPGADYINANYIR(0)
Platypus SHP-2L    PFNATRINAANIENRVKELNKTADQSEKA------KQGFWEEFE(0)MLQQQECKLLYPRKEGQRPENKAK----NRYKNILPF(1)DTTRVTLQEVDHRVPGADYINANYIQ(0)
Chicken SHP-2L     PFNATRINAANIENRVKELNKMADHSEKA------KQGFWEEFE(0)MLQQQECKLLYPRKEGQRPENKAK----NRYKNILPF(1)DTTRVALRDVDESVPGSDYINANYIK(0)
Goose SHP-2L       PFNATRINAANIENRVKELNKMADHSEKA------KQGFWEEFE(0)MLQQQECKLLYPRKEGQRPENKAK----NRYKNILPF(1)DTTRVALRDVDESVPGSDYINANYIK(0)
Lizard SHP-2L      PFNATRIIAANIENRVKELNKMADHSEKA------KQGFWEEFE(0)MLQQQECKFLYPRKEGQRMENKAK----NRYKNILPF(1)DTTRVALREVDESLPGSDYINANYIR(0)
Turtle SHP-2L      PFNATRINAANIENRVKELNKMADHSEKA------KQGFWEEFE(0)MLQQQECKLLYPRKEGQRVENKAK----NRYKNILPF(1)DTTRVALRDVDESLPGSDYINANYIK(0)
Frog SHP-2L        PFNATRINAANIETRVRELNKTADNTEKA------KQGFWEEFE(0)MLQQQECKLLYPRKEGQRPENKSK----NRYKNILPF(1)DTTRVTLRDTDESVAGSDYINANYIK(0)
Lungfish SHP-2L    PFNATRINAANIENRVKELNKTAEHSEKA------KQGFWEEFE(0)VLQQQECKFLYPRKEGQRLENKSK----NRYKNILPF(1)DTTRVKLTELDETIMNYDYINANYIR(0)
Shark SHP-2L       PFNATRINAANIENRVKELNKTADNTEKA------KQGFWEEFE(0)LLQQQEYKLLYSRKEGQRIENKSK----NRYKNILPF(1)DNTRVTLKEVDETILGSDYINANNIT(0)
Skate SHP-2L       PFNATRINAANIENRVKELNKTADNTEKA------KQGFWEEFE(0)LLQQQEHKLLYSRKEGQRIENKNK----NRYKNILPF(1)DNTRVSLKEVDESVVGADYINANDIT(0)
Bichir SHP-2L      PFNTTRINAANIENRVKELNKVADNMEKP------KQGFWEEFE(0)MLQQQECKLLYPRKEGQRPENKSK----NRYKNILPF(1)DTTRVELKETDPEILGSDYINANYIQ(0)
Reedfish SHP-2L    PFNTTRINAANIENRVKELNKVADNMEKP------KQGFWEEFE(0)MLQQQECKLLYPRKEGQRPENKSK----NRYKNILPF(1)DTTRVELKETDPEILGSDYINANYIQ(0)
Sturgeon SHP-2L    PFNATRINAANIENRVKELNKVADHTEKP------KQGFWEEFE(0)MLQQQECKLLYPRKEGQKPENKTK----NRYKNILPF(1)DTTRVELRDADADVPGAEYINANYIR(0)
Paddlefish SHP-2L  PFNATRINAANIENRVRELNKVADHTEKP------KQGFWEEFE(0)MLQQQECKLLYPRKEGQKPENKSK----NRYKNILPF(1)DTTRVELRDADADIPGAEYINANYIR(0)
Gar SHP-2L         PFNATRINAANIENRVRELNKVADNMEKP------KQGFWEEFE(?)MLQQQECKLLYPRKEGQRPANKSK----NRYKNILPF(?)DTTRVILKEVDPEVPGSDYINANYIQ(?)
Bonytongue SHP-2L  PFNATRINAANIENRVRELNKVADNSEKP------KQGFWEEFE(0)VLQQQECKLLYPRKEGQRLENKSK----NRYKNILPF(1)DTTRVVLREADRDVPGADYINANYIQ(0)
Tarpon SHP-2L      PFNATRINAANIENRVRELNKVADNSEKP------KQGFWEEFE(0)VLQQQECKLLYPRKEGQRAENKSK----NRYKNILPF(1)DTTRVELKEADPGVPGSDYINANYIR(0)
Weatherfish SHP-2L PFNATRINAANIENRVRELNKVADNSEKP------KQGFWEEFE(0)VLQQQECKLLYPRKEGQRPENKNK----NRYKNILPF(1)DTTRVQIKEVDPDVLGSDYINANYIR(0)
Zebrafish SHP-2L   PFNATRINAANIENRVHELNKVADNSEKP------KQGFWEEFE(0)VLQQQECKLLYPRKEGQRPENKNK----NRYKNILPF(1)DTTRVQIKEADPDVPGSDYINANYIR(0)
Salmon SHP-2L      PFNATRINAANIENRVKELNKVADNSEKP------KQGFWEEFE(0)VLQQQECKLLYPRKEGQTAENKSK----NRYKNILPF(1)DTTRVEIREADADVPGSDYINANYIR(0)
Perch SHP-2L       PFNATRINAANIENRVRELNKVADNSEKP------KQGFWEEFE(0)VLQQQECKLLYPRKEGQKPENKSK----NRYKNILPF(1)DTTRVEIREKDPDVPGSDYINANYIR(0)
Medaka SHP-2L      PFNATRINAANIENRVRELNKVADNTEKP------KQGFWEEFE(0)VLQQQECKLLYPRKEGQKPENKSK----NRYKNILPF(1)DTTRVVIRDADPEVAGSDYINANYIR(0)
Mummichog SHP-2L   PFNATRINAANIENRVRELNKVADNSEKP------KQGFWEEFE(0)VLQQQECKLLYPRKEGQKPENKSK----NRYKNILPF(1)DTTRVVIKDSESDVPGSDYINANYIR(0)
```

```
                    Exon-8                                                    Exon-9
                        .310      .320      .330      .340      .350              .360      .370       .380      .390      .400
Human SHP-1            NQLLGPDENAKTYIASQGCLEATVNDFWQMAWQENSRVIVMTTREVEKGR(0)NKCVPYWPEVGMQ-RAYGPYSVTNCGEHDTTEYKLRTLQVSPLDN(0)
Lemur SHP-1            NQLLGPDENAKTYIASQGCLEATVNDFWQMVWQENTRIIVMTTREVEKGR(0)NKCVPYWPEVGTQ-RVYGLYSVTNCGEHDTAEHKLRTLQVSPLDN(0)
Mouse SHP-1            NQLLGPDENSKTYIASQGCLDATVNDFWQMAWQENTRVIVMTTREVEKGR(0)NKCVPYWPEVGTQ-RVYGLYSVTNSREHDTAEYKLRTLQISPLDN(0)
Cattle SHP-1           NQLLGPDENAKTYIASQGCLEATVNDFWQMVWQENTCVIVMTTREVEKGR(0)NKCVPYWPEVGSQ-RVYGPYTVTNCGEHDTTEYKLRNLQVSPLEN(0)
Platypus SHP-1         NQLVSVDENAKTYIASQGCLDATVNDFWQMVWQENSRVIVMTTREVEKGR(0)NKCVPYWPEVGSC-KPYGLYSVQNCAEHDAPEYKLRSLQVSPLDN(0)
Chicken SHP-1          NNLISPDECPKTYIASQGCLDTTVNDFWQMVWQENTHIIVMTTREVEKGR(0)NKCVPYWPEAGST-KEYGPYLVENVGEHDALEYKLRHLCVCPIND(0)
Goose SHP-1            NNLISPDECTKTYIASQGCLDATVNDFWQMVWQENTRIIVMTTREVEKGR(0)NKCVPYWPEVGST-KEYGPYLVENTGEHDALEYKLRHLCVCPKNN(0)
Lizard SHP-1           NTMVSPEECTKTYIASQGCLEATINDFWQMVWQENSRIIVMTTREVEKGR(0)NKCVPYWPEVGSS-KEYRPYIVENFGEHDALEYKLRQLRISPIDD(0)
Turtle SHP-1           NQLISPDEFTKTYIASQGCLDATVNDFWQMVWQEKSRIIVMTTREVEKGR(0)NKCVPYWPEEGST-KEYGPYIVENIGEHDALEYKLRQLSLSPVDN(0)
Frog SHP-1             NLLWAEGEPPKRFIACQGCLNSTTGDFWEMVWQENSRVIVMTTKEIEKGR(0)TKCVPYWPDVGTNMKEFGRFLVQLMSERDTKEYKVRLLRVSLTES(0)
Lungfish SHP-1         NRLF---GGGKMYIATQGCLLATVNDFWQMVWQENTRVIIMTTMEVEKGR(0)NKCVPYWPEMDGA-KDFEQYYVRCTQENILKEYKIRVLEVCPMDD(0)
Shark SHP-1         YESPVERDGKMVLKTCIATQGCLLTTVNDFWQMVWQERSCIIVMTTREVEKGR(0)NKCVPYWPDVGTA-KDYGPYTVCHVDERDATDYKVRELDVSLTAK(0)
Skate SHP-1         SETTVLKGGKVVPKTCIATQGCLNATVNEFWQMIWQENSSIIVMTTREVEKGR(?)NKCVLYWPAVDEE-KEFGVYYVRNLGEREAVDYKVRNLQVWPSDK(?)
Bichir SHP-1           NLLT-ESNFRKVYIATQGCLMTTVNDFWQMIWQERSRVIVMTTREVEKGR(0)NKCVPYWPEVETM-KEYGKYIVHHISEHDALDYKIRILEISPVDN(0)
Reedfish SHP-1         NLLT-ESNFRKVYIATQGCLMTTVNDFWQMIWQERSRVIVMTTREVEKGR(0)NKCVPYWPEVETM-KEYGKYIVHHLSEHDALDYKIRILEISPIDN(0)
Sturgeon SHP-1         NMLM-ESCDRKKFIASQGCLATTVNDFWEMVWQERSCVIVMTTREVEKGR(0)NKCVRYWPEPEEM-KEFGSYQVRHVSEHDAVDYTLRVLEISPLNQ(0)
Paddlefish SHP-1       NLLM-ESCDRKKFIASQGCLATTVNDFWEMVWQERSRVIVMTTREVEKGR(0)NKCVRYWPEPEDT-KEFGSYLVRHVSEHDAVDYTLRVLEISPLNQ(0)
Gar SHP-1              NLVR-EPGDQKVYIATQGCLQTTVNDFWQMVWQERSRVIVMTTREVEKGR(0)NKCVPYWPEPEGT-KEYGNYLVRNVSEHDATDYKIRILEISPQDE(0)
Bonytongue SHP-1       NKLS-DSGHQKVYIATQGCLATTVNDFWQMVWQENSRVIVMTTREVEKGR(0)NKCVPYWPELEGT-KEVGSYLVTCLSERDAADYKVRVLEIIPMDQ(0)
Tarpon SHP-1           NKLR-EPGDQKVYIATQGCLATTVNDFWQMVWQEQSRVIVMTTREVEKGR(0)NKCVPYWPELEGS-KEVGRYIVTCLSERDATDYKIRILEITPLDQ(0)
Weatherfish SHP-1      NKLM-EIDHQKVYIACQGCLATTVNDFWQMVWQEKTGVIVMTTREVEKGR(0)NKCVPYWPTTQGERKEAGRYVVTLLSEMDATDYKVRVMELSATHR(0)
Zebrafish SHP-1        NKLM-VTNPQKTYIACQGCLATTVDDFWQMMWQEDSRVIVMTTREVEKGR(0)NKCVPYWPTTEGESKEVGRYVVKLLSEMDAADYKVRVVELTAPHR(0)
Salmon SHP-1           NKLQ-ESGDQKVYIACQGCLATTVNDFWQMVWQEKTRVIVMTTREVEKGR(0)NKCVPYWPEMQGS-KEVGPYVVTCVSERDATDYKVRVMEISPLDQ(0)
Perch SHP-1            NTLW-ESGDQKVYIATQGCLATTVNDFWQMVWQENTSVIVMTTREVEKGR(0)NKCVPYWPELHSS-KEMGPYVVTCESEREAADYKVRVLEIALMDK(0)
Medaka SHP-1           NKLQ-ELGDQKVYIATQGCLATTVNDFWQMVWQENTRVIVMTTREVEKGR(0)NKCVPYWPELHSS-KEVGPYIVTCDTEREAVDYKVRVLEISPMAK(0)
Mummichog SHP-1        DKLW-ESGARKVYIATQGCLATTVNDFWQMVWQENTHVIVMTTREVEKGR(0)NKCVPYWPDLHAS-QESGAYVVSCESEREATDYKVRLLHIAPVNQ(0)
                                                              PTP-domain (continued)
Human SHP-2         PEFETKCNNSKPKKSYIATQGCLQNTVNDFWRMVFQENSRVIVMTTKEVERGK(0)SKCVKYWPDEYAL-KEYGVMRVRNVKESAAHDYTLRELKLSKVGQ(0)
Lemur SHP-2         PEFETKCNNSKPKKSYIATQGCLQNTVNDFWRMVFQENSRVIVMTTKEVERGK(0)SKCVKYWPDEYAL-KEYGVMRVRNVKESAAHDYTLRELKLSKVGQ(0)
Tupaia SHP-2        PEFETKCNNSKPKKSYIATQGCLQNTVNDFWRMVFQENSRVIVMTTKEVERGK(0)SKCVKYWPDEYSL-KEYGVMRVRNVKESAAHDYTLRELKLSKVGQ(0)
Mouse SHP-2         PEFETKCNNSKPKKSYIATQGCLQNTVNDFWRMVFQENSRVIVMTTKEVERGK(0)SKCVKYWPDEYAL-KEYGVMRVRNVKESAAHDYTLRELKLSKVGQ(0)
Cattle SHP-2        PEFETKCNNSKPKKSYIATQGCLQNTVNDFWRMVFQENSRVIVMTTKEVERGK(0)SKCVKYWPDEYAL-KEYGVMRVRNVKESAAHDYTLRELKLSKVGQ(0)
Platypus SHP-2      PEFETKCNNTKPKKSYIATQGCLQNTVNDFWRMVFQENSRVIVMTTKEVERGK(0)SKCVKYWPDEYAL-KEYGVMRVRNVKESAAHDYTLRELKLSKVGQ(0)
Chicken SHP-2       PEFETKCNNSKPKKSYIATQGCLQNTVNDFWRMVFQENSRVIVMTTKEVERGK(0)SKCVKYWPDEYSL-KEYGVMRVRNVKESAAHDYTLRELKLSKVGQ(0)
Goose SHP-2         PEFETKCNNSKPKKSYIATQGCLQNTVNDFWRMVFQENSRVIVMTTKEVERGK(0)SKCVKYWPDEYSL-KEYGVMRVRNVKESAAHDYTLRELKLSKVGQ(0)
Lizard SHP-2        PEFETKCNNTKPKKSYIATQGCLQNTVNDFWRMVFQENSRVIVMTTKEVERGK(0)SKCVKYWPDEHAL-KEYGVMRVRNVKESAAHDYTLRELKLSKVGQ(0)
Turtle SHP-2        PEFESKCNNSKPKKSYIATQGCLQNTVNDFWRMVFQENSRVIVMTTKEVERGK(0)SKCVKYWPDEYAL-KEYGVMRVRNVKESAAHDYTLRELKLSKVGQ(0)
Frog SHP-2          PEFETKCNNPKPKKRYIATQGCLQNTVNDFWRMVFQENSRVIVMTTKEVERGK(0)SKCVKYWPDEFAL-KEYGVMRVRNVKETPAHDYILRELKLSKVGQ(0)
Lungfish SHP-2      PESETRSNNSKSKKCYIATQGCLQNTVNDFWRMVFQENSMVIVMTTKEVERGK(0)SKCVKYWPDEYQL-KEYGVMRVRNVRETPAHDYILRELKLSKVGQ(0)
Shark SHP-2         HDIESNRTTTKPKKSYIATQGCLQNTVNDFWRMVFQENSSVIVMTTKEVERGK(0)SKCVKYWPDEMSL-KEYGGMRVRNVRESPAHDYILRELKLSKVGQ(0)
Skate SHP-2         HDIDSNRTTTRPKKSYIATQGCLQNTVNDFWRMVFQENSRVIVMTTKEVERGK(0)SKCVKYWPDEMSL-KEYGAMRVRNVRENPAHDYILRELKLSQVGQ(0)
Bichir SHP-2        PEFDSKCNNTKLKKCYIATQGCLQNTISDFWRMVFQENSRVIVMTTKEVERGK(0)SKCLRYWPDVSAL-KEYGAMRVRNVKETLAHDYILRELKLSKVGQ(0)
Reedfish SHP-2      PEFDSKCNNTRLKKSYIATQGCLQNTISDFWRMVFQENSRVIVMTTKEVERGK(0)SKCLRYWPDVSAL-KEYGAMRVRNVKETLAHDYILRELKLSKVGQ(0)
Sturgeon SHP-2      PEVEVKCNNTKPKKSYIATQGCLQNTISDFWRMVFQENSRVIVMTTKEVERGK(0)SKCVKYWPDVTVL-KEYGAMRVRNARETSAHDYILRELRMSKVGQ(0)
Paddlefish SHP-2    PEVEVKCNNTKPKKSYIATQGCLQNTISDFWRMVFQENSRVIVMTTKEVERGK(0)SKCVKYWPDVTVL-KEYGAMRVRNARETSAHDYILRELRMSKVGQ(0)
Gar SHP-2           PEFETKCNNSKSKKCYIATQGCLQNTISDFWRMVFQENSRVIVMTTKEVERGK(0)SKCVKYWPDVSAL-KEYGAMRVRNVKENSAHDYILRELKLSKVGQ(0)
Bonytongue SHP-2    PELEFKCNNAKVKKCYIATQGCLQNTVSDFWRMVFQENSCVIVMTTKEVERSK(0)SKCVKYWPDVSAL-KEYGAMRVRNVKETLAHDYILRELKLSKVGQ(0)
Tarpon SHP-2        PELESKCNNAKLKKSYIATQGCLQNTISDFWRMVFQENSRVIVMTTKEVERGK(0)SKCVKYWPDVSAL-KEYGAMRVRNVKETSAHDYILRELKLSKVGQ(0)
Weatherfish SHP-2   PELENKGNN-KLKRSYIATQGCLQNTISDFWRMVFQENTRVIVMTTKEVERGK(0)SKCVKYWPDATAL-KEYGAMRVRNVKETQAHDYILRELKLSKVGQ(0)
Zebrafish SHP-2     PDNEAKSNNSKLKRSYIATQGCLQNTISDFWRMVFQENSRVIVMTTKEVERGK(0)SKCVKYWPDVSAL-KEYGAMRVRNVKETMAHDYILRELKLSKVGQ(0)
Salmon SHP-2        PELEWKCNSTKLKKSYIATQGCLQNTISDFWRMVFQENSRVIVMTTKEVERGK(0)SKCVKYWPDMSSL-KEYGIMRVRNVKETSAHDYILRELKLSKVGQ(0)
Perch SHP-2         PELDSKCNSTKVKKSYIATQGCLQNTISDFWRMVFQENSRVIVMTTKEVERGK(0)SKCVKYWPDMNAL-KEYGAMRVRNVRETAAHDYILRELKLSKVGQ(0)
Medaka SHP-2        PELDAKCNSHKLKKSYIATQGCLQNTISDFWRMVFQENSRVIVMTTKEVERGK(0)SKCVKYWPDMSAL-KEYGAMRVRNVRETSAHDYILRELKLSKVGQ(0)
Mummichog SHP-2     PEADIKCNNAKPKKSYIATQGCLQNTISDFWRMVFQENSRVIVMTTKEVERGK(0)SKCVKYWPEMSAL-KEYGAMRVRNVRETNAHDYILRELKLSKVGQ(0)
Lemur SHP-2L         SDPEEKPGHERGKVYIATQGCLQTTVAAFWAMVYQENTHVIVMTTREVERGR(0)NKCFRYWPELHGS-QEYGHVHVRNVAERQAQGYCVRELQVWQPDQ(0)
Tupaia SHP-2L        SDPEEKPGHGLGKVYIATQGCLQTTVAAFWAMVHQENTCVIVMTTKEVERGR(0)NKCFRYWPELYSS-QEYGQVHVSNIAEHQAQGYCVRELQVWRPDQ(0)
Cattle SHP-2L        SDPEEKPGHGLGKVYIATQGCLPTTVAAFWAMVHQENTHVIVMTTREVERGR(0)NKCFRYWPELHGS-QEYGHLHVCNMAEHWAQGYCVRELQVWRPDQ(0)
Platypus SHP-2L      NIGEDGQSSEQDKVYIATQGCLQTTVNDFWAMVYQENCHVIVMTTKEMERGR(0)NKCFRYWPDRDSS-KEFGCICVRNTEEREAQGYLLRELEIRRTDR(0)
Chicken SHP-2L       SIPEDGRNSEHCKIYIATQGCLQTTVNDFWTMVYQENTHVIVMTTKEVERGR(0)NKCFRYWPDKGCA-KEYGCICVRNISEREAQGYYLRELEILHTDR(0)
Goose SHP-2L         SIPEDGRNSEHCKIYIATQGCLQTTVNDFWTMVYQENSHVIVMTTKEVERGR(0)NKCFRYWPDKGCA-KEYGCICVRNVSEREAQGYYLRELEIVRTDR(0)
Lizard SHP-2L        NIAEDGQSSEHSKVYIATQGCLQTTVNDFWAMVYQENTHVIVMTTKEVERGR(0)NKCFHYWPDKNCT-KDYGYISVRNVSELEAQGYHIRELEIARLDR(0)
Turtle SHP-2L        SIPEDGRSSEHCKVYIATQGCLQTTVNDFWAMVYQENTHVIVMTTKEVERGR(0)NKCFRYWPDKNCT-KEYGHICVRIVSEREAQGYYLRELEITRTDR(0)
Frog SHP-2L          STLDEARGSQHYKVYIATQGCLQNTVCDFWAMVYQENTHVIVMTTKEVERSR(0)KKCFRYWPDENCT-KDYGCIRVQSLEVQQKKDYILRELQISRMDK(0)
Lungfish SHP-2L      NIPEDGKSAEECKSYIATQGCLQNTVNDFWRMVYQENTHVIVMTTKEVERGR(0)NKCVRYWPEENSF-KEYGNICIRNVEEHTAQDYVLRILELTRLDR(0)
Shark SHP-2L             NGKLGEDSKHYIATQGCLQNTINDFWKMIYQENAHVIVMTTKEVERGR(0)NKCYRYWPDLETS-KLYGTISVRNLKERMAQDYIVRELEVIQTAW(0)
Skate SHP-2L             NGKSGDDCKNYIATQGCLQNTINDFWKIIYQENAHVIVMTTKEIERGK(0)NKCVRYWPDLETS-KEYGLISVRNLEERMAQDYIVRDLEVTRIDQ(0)
Bichir SHP-2L        SVQEDSRQLCKGKVFIATQGCLQNTVKDFWKMVYQENTHVIVMTTKEVERGR(0)NKCVRYWPDMEAT-KEYGSLQVRNIEERTAQDYVLRKLEVSCLNR(0)
Reedfish SHP-2L      SVQEDSRQLCKGKVFIATQGCLQNTVKDFWKMVYQENTHVIVMTTKEVERGR(0)NKCVRYWPDLETT-KEYGSLQVRNIEERTAQDYVLRKLEVSCLNG(0)
Sturgeon SHP-2L      SVHEDGRRPDECKVYIATQGCLQNTVNDFWNMVYQENTHVIVMTTKEVERGR(0)NKCVRYWPDVDSQ-KEYGLLCVRNMEERPAQDYVLRELEITRLDR(0)
Paddlefish SHP-2L    SVHEDGRRPDECKVYIATQGCLQNTVNDFWNMVYQENTHVIVMTTKEVERGR(0)NKCVRYWPDVDSR-KEYGLLCVRNMEERPAQDYVLRELEITRLDR(0)
Gar SHP-2L           SVQDDGRLVGVSKMYIATQGCLQNTVEDFWKMVYQENTHVIVMTTKELERGR(?)NKCVRYWPDCDCS-QDFGRVCVRNVEERPAQDYILRILNVTHLDR(?)
Bonytongue SHP-2L    SKHEEGHLADECKVYIATQGCLQNTVTDFWQMVYQENTHVIVMTTKEMERGR(0)NKCVRYWPDVDTT-KDFGKLSVRNIEERPAQDYVLRELEITRLDT(0)
Tarpon SHP-2L        NLHEDGRYVDEGKVFIATQGCLQNTVVDFWKMVYQENTHVIVMTTKEMERGR(0)NKCVRYWPDIDCT-KEFGKVRVKNIEERPAQDYVLRELEVTRTDR(0)
Weatherfish SHP-2L   STPEEGRHTDEGKVFIATQGCLQNTVLDFWKMVYQENTHVIVMTTKEMERGR(0)NKCVRYWPDLNST-REFGKLSVKNIEEHTAQDYIRRELEVTRLDR(0)
Zebrafish SHP-2L     SVNEEGRHMDEGKVFIATQGCLQNTVLDFWKMVYQENTHVIVMTTKEMERGR(0)NKCVRYWPDLNST-KEFGKVCVKNIEEHTAQDYIRRELEVTRLDR(0)
Salmon SHP-2L        SMHEEGCHVEEGKVFIATQGCLQNTVVDFWKMVYQENTHVIVMTTKEMERGR(0)NKCVRYWPDLNAT-KEFGKVCVKNVEERPAQDYILRELEVTRLDR(0)
Perch SHP-2L         SMHEEGRHMEEGKVFIATQGCLQNTVIDFWKMVYQENTHVIVMTTKEIERGR(0)NKCVRYWPDLNAT-KEFGKVLVRNVDERPAQDYILRKLEVTRLDR(0)
Medaka SHP-2L       QSMLEDGRHVVEGKVFIATQGCLQNTVVDFWKMVHQENTQVIVMTTKETERGR(0)NKCARYWPDLHCT-KEFGRLLVRNVDERPAQDYILRKLEVTCSDR(0)
Mummichog SHP-2L     NMNEDGRHVEESKVFIATQGCLQNTVIDFWKMVYQENAHVIVMTTKEMERGR(0)NKCVRYWPDLHGT-KEFGKVLLRNVEERPAQDYVLRKLEVTRLDR(0)
```

```
                  Exon-10                                                Exon-11
                         .410      .420      .430        .440      .450      .460         .470
Human SHP-1       GDLIREIWHYQYLSWPDHGVPSEPGGVLSF--LDQINQRQESLPHAGPIIVHCS(2)AGIGRTGTIIVIDMLMENISTKG(1)
Lemur SHP-1       GDLVREIWHYQYLSWPDHGVPSDPGGVLSF--LDQINQRQESLPHAGPIIVHCS(2)AGIGRTGTIIVIDMLMENISTKG(1)
Mouse SHP-1       GDLVREIWHYQYLSWPDHGVPSEPGGVLSF--LDQINQRQESLPHAGPIIVHCS(2)AGIGRTGTIIVIDMLMESISTKG(1)
Cattle SHP-1      ENLIREIWHYQYLSWPDHGVPSEPGGVLSF--LDQINQRQESLPHAGPIIVHCS(2)AGIGRTGTIIVIDMLMESISTKG(1)
Platypus SHP-1    GDLVREIWHYQYLSWPDHGVPSEPGGVLSF--LDQINQRQESLPHPGPIVVHCS(2)AGIGRTGTIIVIDMIMESISTKG(1)
Chicken SHP-1     SEAVREIWQYQYLSWPDHGVPSEPGGVLSF--LDQINQKQESIPNAGPILVHCS(2)AGIGRTGTIIVIDMIVETISTKG(1)
Goose SHP-1       GDAVREIWHYQYLSWPDHGVPSEPGGVLSF--LDQINQKQESIPAAGPILVHCS(2)AGIGRTGTIIVIDMIVETISTKG(1)
Lizard SHP-1      GEAVRDIWHYQYLSWPDHGVPSEPGGVLGF--LDQINQKQESIPAAGSIVVHCS(2)AGIGRTGTIIVIDMIVDMISTKG(1)
Turtle SHP-1      CKAVREIWQYQYLSWPDHGVPSEPGGVLSF--LDQINQKQESIPEAGPIVVHCS(2)AGIGRTGTIIVIDMIVETISTKG(1)
Frog SHP-1        TEKSRDIHHYQYLTWPDHGVPADPGGVLSF--LEEVNSMQESMPRAGAIVVHCS(2)AGIGRTGTIIAIDMLLDLIQMKG(1)
Lungfish SHP-1    GDNIREIWHYQYTAWPDHGVPSEPGGVLGF--LEVVNKKQQELGSVGPMVVHCS(2)AGIGRTGTIIVIDMLVDTIDLKG(1)
Shark SHP-1       GSKARRVYHYQYLSWPDHGVPSEPGGVLSF--LQQVNTQQAKLRHAGPIVVHCS(2)AGIGRTGTIIIIDMLVDKIDRQG(1)
Skate SHP-1       SASPRNIYHYQYMSWPDHGVPSEPGGVLSF--LEQVNQRQESLPDAGPIIIHCS(?)AGIGRTGTIIVIDMLIDKINRQG(?)
Bichir SHP-1      SDDTREIWHYQYLSWPDHGVPEEPGGVLSF--LEQVNAKQQEILGAGPIVVHCS(2)AGIGRTGTIIVIDMLVDTINIKG(1)
Reedfish SHP-1    SDDTREIWHYQYLSWPDHGVPEEPGGVLSF--LEQVNAKQQEILGAGPIVVHCS(2)AGIGRTGTIIVIDMLVDTINIKG(1)
Sturgeon SHP-1    SDDLREIWHYQYLSWPDHGVPAEPGGVLSF--LEQVNSKQSELRSAGPIVVHCS(2)AGIGRTGTIIVIDMVINTINTKG(1)
Paddlefish SHP-1  SDDQREIWHYQYLSWPDHGVPTEPGGVLSF--LEQVNSKQRELRGAGPIIVHCS(2)AGIGRTGTIIVIDMVINTINTKG(1)
Gar SHP-1         SEDTREIWHYQYLSWPDHGVPLEPGGVLSF--LTQVNNKQLEIRGAGPMIVHCS(2)AGIGRTGTIIVIDMLVETIDTKG(1)
Bonytongue SHP-1  SDCPRTIWHYQYLSWPDHGVPEEPGGVLSF--LTQVNSKQMEFADAGPMIIHCS(2)AGIGRTGTIVVIDMLVEIIDTKG(1)
Tarpon SHP-1      SDAPREIWHYQYLSWPDHGVPHEPGGVLSF--LSQVNSKQMEFPNAGPMIIHCS(2)AGIGRTGTIVVIDMLIETIDTKG(1)
Weatherfish SHP-1 KEAPRTIWHYQYLSWPDHGVPQEPGGVLSF--LEQVNGKQHELSSNGPMIIHCS(2)AGIGRTGTIVVIDMLIDTIDAKG(1)
Zebrafish SHP-1   NEAPRKIWHFQYLSWPDHGVPQEPGGVLSF--LDQVNRKQEELRSSAPIVIHCS(2)AGIGRTGTIVVIDMLIDSIDAKG(1)
Salmon SHP-1      SDSVRTIWHYQYLSWPDHGVPEEPGGVLSF--LSQVNLKQAEFTNAGPMIIHCS(2)AGIGRTGTIVVIDMIIKTIDTKG(1)
Perch SHP-1       PKQSRQVWHYQYLSWPDHGVPQQPGGVLSF--LTQVNAKQAEYPHAGPMIIHCS(2)AGIGRTGTILVIDMILETIDTLG(1)
Medaka SHP-1      PQLSRTIWHYQYLSWPDHGVPQEPGGVLSF--LIQVNGKQAEYPEAGPIIVHCS(2)AGIGRTGTILVIDMIIDTIDTLG(1)
Mummichog SHP-1   AKLSRPIWHYQYLSWPDHGVPQEPGGVLSF--LTQVNAKQAEYPEAGPMIIHCS(2)AGIGRTGTIVVIDMILETIDTIG(1)
                                               PTP-domain (continued)
Human SHP-2       GNTERTVWQYHFRTWPDHGVPSDPGGVLDF--LEEVHHKQESIMDAGPVVVHCS(2)AGIGRTGTFIVIDILIDIIREKG(1)
Lemur SHP-2       GNTERTVWQYHFRTWPDHGVPSDPGGVLDF--LEEVHHKQESIMDAGPVVVHCS(2)AGIGRTGTFIVIDILIDIIREKG(1)
Tupaia SHP-2      GNTERTVWQYHFRTWPDHGVPSDPGGVLDF--LEEVHHKQESIMDAGPVVVHCS(2)AGIGRTGTFIVIDILIDIIREKG(1)
Mouse SHP-2       GNTERTVWQYHFRTWPDHGVPSDPGGVLDF--LEEVHHKQESIVDAGPVVVHCS(2)AGIGRTGTFIVIDILIDIIREKG(1)
Cattle SHP-2      GNTERTVWQYHFRTWPDHGVPSDPGGVLDF--LEEVHHKQESIVDAGPVVVHCS(2)AGIGRTGTFIVIDILIDIIREKG(1)
Platypus SHP-2    GNTERTVWQYHFRTWPDHGVPSDPGGVLDF--LEEVHHKQENITDAGPVVVHCS(2)AGIGRTGTFIVIDILIDIIREKG(1)
Chicken SHP-2     GNTERTVWQYHFRTWPDHGVPSDPGGVLDF--LEEVHHKQESISDAGPVVVHCS(2)AGIGRTGTFIVIDILIDIIREKG(1)
Goose SHP-2       GNTERTVWQYHFRTWPDHGVPSDPGGVLDF--LEEVHHKQESISDAGPVVVHCS(2)AGIGRTGTFIVIDILIDIIREKG(1)
Lizard SHP-2      GNTERTVWQYHFRTWPDHGVPSDPGGVLDF--LEEVHHKQEGIADAGPVVVHCS(2)AGIGRTGTFIVIDILIDIIREKG(1)
Turtle SHP-2      GNTERTVWQYHFRTWPDHGVPSDPGGVLDF--LEEVHHKQESIADSGPVVVHCS(2)AGIGRTGTFIVIDILIDIIREKG(1)
Frog SHP-2        GNTERTVWQYHFKTWPDHGVPTDPGGVLDF--LEEVHHKQDGIADAGPVVVHCS(2)AGIGRTGTFIVIDILIDVIREKG(1)
Lungfish SHP-2    GNTERTVWQYHFKTWPDHGVPADPGGVLDF--LEEVHLKQENISEVGPIVVHCS(2)AGIGRTGTFIVIDILIDIIREKG(1)
Shark SHP-2       GNTERTVWQYHFKTWPDHGVPSDPGGVLDF--LEEVNIKQENIPETGPIVVHCS(2)AGIGRTGTFIVIDILIDIIREKG(1)
Skate SHP-2        STERTVWQYHFKTWPDHGVPSDPGGVLDF--LEEINIKQENIPETGPSVVHCS(2)AGIGRTGTFIVIDILIDIIREKG(1)
Bichir SHP-2      GNTERTVWQYHFRTWPDHGVPGDPGGVLDF--LEEVNHKQESIFEAGPIVVHCS(2)AGIGRTGTFIVIDILIDIIREKG(1)
Reedfish SHP-2    GNTERTVWQYHFRTWPDHGVPGDPGGVLDF--LEEVNHKQESILEAGPIVVHCS(2)AGIGRTGTFIVIDILIDIIREKG(1)
Sturgeon SHP-2   GPDDERTVWQYHFRAWPDHGVPGDPGGVLDF--LEEVNMKQESILEAGPIVVHCS(2)AGIGRTGTFIVIDMLIDVIREKG(1)
Paddlefish SHP-2 GPDDERTVWQYHFRAWPDHGVPGDPGGVLDF--LEEVNMKQESILEAGPIVVHCS(2)AGIGRTGTFIVIDMLIDVIREKG(1)
Gar SHP-2         GNTERTVWQYHFRAWPDHGVPGDPGGVLDF--LEEVNLKQESILEAGPIVVHCS(2)AGIGRTGTFIVIDILIDIIREKG(1)
Bonytongue SHP-2  GNTERTVWQYHFRAWPDHGVPTDPGGVLDF--LEEVNLKQESILEAGPIVVHCS(2)AGIGRTGTFIVIDILIDVIREKG(1)
Tarpon SHP-2      GNTERTVWQYHFRAWPDHGVPTDPGGVLDF--LEEVKLKQESILEAGPIVVHCS(2)AGIGRTGTFIVIDILIDVIREKG(1)
Weatherfish SHP-2 GNTERTVWQYHFRAWPDHGVPGDPGGVLDF--LEEVKLKQEGITGAGPIVVHCS(2)AGIGRTGTFIVIDILIDIIREKG(1)
Zebrafish SHP-2   GNTERTVWQYHFRAWPDHGVPGDPGGVLDF--LEEVKLKQEGITGAGPIVVHCS(2)AGIGRTGTFIVIDILIDIIREKG(1)
Salmon SHP-2      GNTERTVWQYHFRAWPDHGVPTDPGGVLDF--LEEVNLKQESILEAGPIVVHCS(2)AGIGRTGTFIVIDILIDVIREKG(1)
Perch SHP-2       GNTERTVWQYHFRAWPDHGVPTDPGGVLDF--LEEVNLKQESILDAGPIAVHCS(2)AGIGRTGTFIVIDILIDVIREKG(1)
Medaka SHP-2      GNTERTVWQYHFRAWPDHGVPTDPGGVLDF--LEEVNLKQESIADAGPIVVHCS(2)AGIGRTGTFIVIDILIDVIREKG(1)
Mummichog SHP-2   GNTERTVWQYHFRTWPDHGVPTDPGGVLDF--LEEVNLKQEGILDAGPITVHCS(2)AGIGRTGTFIVIDILIDVIREKG(1)
Lemur SHP-2L      EEPPRKVKHYQYFSWPDHGVPAEPAGVLGF--LDEVNRAQSSVPEAGPMVVHCS(2)AGIGRTGTIIVIDILVGVVRRKG(1)
Tupaia SHP-2L     VEPPRTVKHYQYFSWPDHGVPAEHAGVLGFRRLDEVNRTQSSVPGAGPMVVHCS(2)AGIGRTGTIIVIDILVDVIRKQG(1)
Cattle SHP-2L     EESPHTVKHYQYFSWPDHGVPAEPTGVLSF--LEEVNRTHSSMPGAGPMVVHCS(2)AGIGRTGTIIVIDILVDVIRRQG(1)
Platypus SHP-2L   EEQPRKVKHYQYFSWPDHGVPTEPGGVLSF--LDQVNRAQRGYVNTGPIIVHCS(2)AGIGRTGTIIVIDILVDVIHRQG(1)
Chicken SHP-2L    DERPRVVKHYQYFSWPDHGVPNEPGGVLSF--LDQVNRTQRSIPDTGPIIVHCS(2)AGIGRTGTIIVIDILVDIIHRQG(1)
Goose SHP-2L      DERPRVVKHYQYFSWPDHGVPNEPGGVLSF--LDQVNRAQRSIPDTGPIIVHCS(2)AGIGRTGTIIVIDILVDIIHRQG(1)
Lizard SHP-2L     EEQPRLIWHFQYISWPDHGVPNEPGGVLSF--LDQVNKAQRSVPGTGPIVVHCS(2)AGIGRTGTIIVIDILVDTIHRQG(1)
Turtle SHP-2L     EEHPRQVKHYQYFSWPDHGVPNEPGGVLSF--LDQVNRAQRSIPDSGPIVVHCS(2)AGIGRTGTIIVIDILVDTIHRQG(1)
Frog SHP-2L       VESPRPIWHYQYLSWPDHGVPIEPGGVLSF--LEEINAAQKYIPHSGPIVVHCS(2)AGIGRTGTIIVIDMLVDIINRQG(1)
Lungfish SHP-2L   HEVPRQIWQYQYLSWPDHGVPNEPGGVLSF--LDQVNQEQQSIPHAGPIIVHCS(2)AGIGRTGTIIVIDILIDIINRQG(1)
Shark SHP-2L      KEAPRYIWHYQYLSWPDHGVPNEPGGVLSF--LDQVNRTQQSIPDTGPIIVHCS(2)AGIGRTGTIIVIDMLVADINRQG(1)
Skate SHP-2L      NDLPRHIWHYQYLSWPDHGVPNEPGGVLSF--LDQVNRAQQSIPDMGPIIVHCS(2)AGIGRTGTIIVIDMLISDISRQG(1)
Bichir SHP-2L     DEPHRYIWHYQYLSWPDHGVPNEPGGVLSF--LEQVNRTQQSIPDTGPIVVHCS(2)AGIGRTGTIIVIDILIDIINRQG(1)
Reedfish SHP-2L   DEPHRYIWHYQYLSWPDHGVPNEPGGVLSF--LEQVNRTQQSIPDTGPIVVHCS(2)AGIGRTGTIIVIDILIDIINRQG(1)
Sturgeon SHP-2L   EEPPRYIWHYQYLSWPDHGVPNEPGGVLSF--LDQVNQAQQSIPDTGPIVVHCS(2)AGIGRTGTIIVIDILVDIISRQG(1)
Paddlefish SHP-2L EEPPRYIWHYQYLSWPDHGVPNEPGGVLSF--LDQVNQAQKSIPDTGPIVVHCS(2)AGIGRTGTIIVIDILIDIIGRQG(1)
Gar SHP-2L        NEPPRYIWHYQYLSWPDHGVPNEPGGVLSF--LDQVNRAQNSVQDTGPIVVHCS(?)AGIGRTGTIIVIDILIDIINRQG(?)
Bonytongue SHP-2L REPPRYIWHYQYLSWPDHGVPNEPGGVLSF--LDQVNRAQNSIPNTGPIVVHCS(2)AGIGRTGTIIVIDILIDIINRQG(1)
Tarpon SHP-2L     NDPPRYIWHYQYLSWPDHGVPNEPGGVLSF--LDQVNRAQSAFPQTGPIVVHCS(2)AGIGRTGTIIVIDLLIDIINRQG(1)
Weatherfish SHP-2LREPPRCIWHYQYLSWPDHGVPNEPGGVLSF--LEQVNRTQSAIPESGPIVVHCS(2)AGIGRTGTIIVIDILINIINRQG(1)
Zebrafish SHP-2L  REPPRCIWHYQYLSWPDHGVPNEPGGVLSF--LEQVNRTQSAIPESGPIVVHCS(2)AGIGRTGTIIVIDILIDIINRQG(1)
Salmon SHP-2L     REPMRYIWHYQYLSWPDHGVPNEPGGVLSF--LEQVNRTQSTIQDTGPIVVHCS(2)AGIGRTGTIIVIDILIDIINRQG(1)
Perch SHP-2L     LKEHSRYIWHYQYLSWPDHGVPNEPGGVLWF--LEEVNRTQSTIPDAGPIVVHCS(2)AGIGRTGTIIVIDILINIINRQG(1)
Medaka SHP-2L      EPLRYIWHYQYLSWPDHGVPNEPGGVLWF--LEEINRTQNSVKEAGPIVVHCS(2)AGIGRTGTIIVIDILIDIISRQG(1)
Mummichog SHP-2L  KEPQRHIWHYQYLSWPDHGVPNEPGGVLWF--LEEVNRTQGTIKEAGPIIVHCS(2)AGIGRTGTIIVIDILIDVINRQG(1)
```

```
                   Exon-12                                               Exon-13
                      .480      .490      .500      .510      .520                                .530        .540      .550
Human SHP-1        LDCDIDIQKTIQMVRAQRSGMVQTEAQYKFIYVAIAQFIETTKKKLEVLQ(0)------------------------SQKGQ--ESEYGNITYPPAMKNAHAKASRT-SSK(2)
Lemur SHP-1        LDCDIDIQKTIQMVRAQRSGMVQTEAQYKFIYVAIAQFIETTKKKLEVMQ(0)------------------------SQKGQ--ESEYGNINYPPAMKNAHAKAART-SSK(2)
Tupaia SHP-1       LDCDIDIQKTIQMVRAQRSGMVQTEAQYKFIYVAIAQFIETTKKKLEVMQ(0)------------------------SQKGQ--ESEYGNITYPPAMKNAHAKASRT-SSK(2)
Mouse SHP-1        LDCDIDIQKTIQMVRAQRSGMVQTEAQYKFIYVAIAQFIETTKKKLEIIQ(0)------------------------SQKGQ--ESEYGNITYPPAVRSAHAKASRT-SSK(2)
Cattle SHP-1       LDCDIDIQKTIQMVRAQRSGMVQTEAQYKFIYVAIAQFIETTKKKLEVMQ(0)------------------------SQKGR--ESEYGNITYPPAMKNAHAKASRT-SSK(2)
Platypus SHP-1     LDCDIDIQKTIQMVRAQRSGMVQTEAQYKFIYMAIAQFIETTKQKLDILQ(0)------------------------AQKGR-VESEYGNIGYPPAMKNAHAKACRT-SSR(2)
Chicken SHP-1      LDCDIDIQKTIQMVRAQRSGMVQTEAQYKFIYMAICQFIETTKRKLEVIQ(0)------------------------SQKSKQNESEYGNITYPPAVKNMHAKVSRK-SSK(2)
Goose SHP-1        LDCDIDIQKTIQMVRSQRSGMVQTEAQYKFIYMAICQFIETTKKKLEVIQ(0)------------------------SQKSKQNESEYGNIAYPPAMKNAHAKASRKASNR(2)
Lizard SHP-1       LDCDIDIPKVIQMVRSQRSGMVQTEAQYKFIYMAICQFIETTKKKLDVMQ(0)------------------------SQKGRPNESEYGNITYPPAQKTSHAKASRK-SSK(2)
Turtle SHP-1       LDCDIDIQKTIQMVRAQRSGMVQTEAQYKFIYMAICQFIETTKKKLDVMQ(0)------------------------SQKGRSKESEYGNIAYPPALKNQHAKASRK-SSK(2)
Frog SHP-1         VDSDIDVQKTIQMVRNQRSGMVQTEAQYKFIYSAIAQFIDSTKHKLKAME(0)------------------------NRNAT--ESEYGNLG----ASLTKMKVTRS-SSK(2)
Lungfish SHP-1     VDCDIDIQKTIQMVRSQRSGMVQTEAQYKFIYMSVAHYIETVKKRLEAGQ(0)------------------------NPSN---ESEYGNIRTL-GLKNPHAKAARK-SSH(2)
Shark SHP-1        VDCDIDIQKTIRAVRAQRSGMVQTEAQYKFIYMAVCQYIETTQRKLQLVQ(0)------------------------ENTDA--GSEYGNIRYPPLKSSQHGKLARKTSSN(2)
Skate SHP-1        LDCDIDIQKTIQSVRSQRSGMVQTEAQYKFIYMAVSQYIETTQRKLQFVE(?)------------------------GNKAV--ESEYGNISYPPQKSTHTKVSQRASNPG(?)
Bichir SHP-1       VDCDIDIQKSIQMVREQRSGMVQTEAQYKFIYMAVSQYIETTKRKLEEIG(0)------------------------KT-----ETEYGNLKYP---TAQHGKASRK-SSR(2)
Reedfish SHP-1     VDCDIDIQKSIQMVREQRSGMVQTEAQYKFIYMAVSQYIETTKRKLEEIG(0)------------------------KT-----ETEYGNLKYP---TGQHGKASRK-SSR(2)
Sturgeon SHP-1     LDCDIDIQKSIQMVREQRSGLVQTEAQYKFIYMAVSQYIETTKRKLDEMK(0)------------------------KT-----ETEYGNLHFP---MAQHSKASRK-LSK(2)
Paddlefish SHP-1   LDCDIDIPKSIQMVREQRSGLVQTEAQYKFIYMAVSQYIETTKRKLEEMK(0)------------------------KT-----ETEYGNLHFP---MAQHSKASRK-LSK(2)
Gar SHP-1          LDCDIDIQKCIQMVREQRSGMVQTEAQYKFIYLAVSQYIETTKKKLEALG(0)------------------------SA-----EMEYGNLSFP----NKHTKASRK-VSK(2)
Bonytongue SHP-1   LDCDIDIPKFIQMVREQRSGMVQTEAQYKFIYLAVLQYIETTKSKMQACE(0)------------------------NL-----ETEYGNLSLQ----PKHQKVSRK-VSK(2)
Tarpon SHP-1       LDCDIDIQKSIQMVREQRSGMVQTEAQYKFIYLAVSQYIETTKTKLQALG(0)------------------------NT-----ETEYGNLSLQ----PK-QKASRK-VSK(2)
Weatherfish SHP-1 LDCDIDIQKCIQMVRDQRSGMVQTEAQYKFIYMAVLHYIESTKVTHKAVL(0)------------------------ET-----ETEYGNLSIQ----PKHQKASRKTSSK(2)
Zebrafish SHP-1    LDCDIDIQKCIMMVRDQRSGMVQTEAQYKFIYLAVLQYVESTKVTRRAIM(0)------------------------ET-----ETEYGNLSIQ----SKHPKASRKASSK(2)
Salmon SHP-1       LDCDIDIQKSIQMVRDQRSGMVQTEAQYKFIYLAVSEYIEASKTYNK---(0)------------------------GA-----ETEYGNLQF------KHQPASRK-VSK(2)
Perch SHP-1        LDCDIDIPKYIQMVREQRSGMVQTEAQYKFIYLAVSEYIQTTKAKDSASM(0)------------------------ET-----ETEYGNLQL-------KQPASRK-VSK(2)
Medaka SHP-1       LDCDIDIPKYIQMVREQRSGMVQTEAQYKFIYLAVSEYIQTTKTKDCAYM(0)------------------------ET-----DTEYGNLQL-------KHQPASRKVSK(2)
Mummichog SHP-1    LDCDIDIPKYIQMVREQRSGMVQTEAQYKFIYLAVSEYIQTTKAKESASK(0)------------------------ET-----ETEYGNLQL-------KHQPASRKVSK(2)
                               PTP-domain (continued)      >
                                                                                                        .Y542
Human SHP-2        VDCDIDVPKTIQMVRSQRSGMVQTEAQYRFIYMAVQHYIETLQRRIEEEQ(0)------------------------KSKRK--GHEYTNIKYSLADQT-SGDQ--SPLPPCTPTP----PCAE(2)
Lemur SHP-2        VDCDIDVPKTIQMVRSQRSGMVQTEAQYRFIYMAVQHYIETLQRRIEEEQ(0)------------------------KSKRK--GHEYTNIKYSLADQT-SGDQ--SPLPPCTPTP----SCAE(2)
Tupaia SHP-2       VDCDIDVPKTIQMVRSQRSGMVQTEAQYRFIYMAVQHYIETLQRRIEEEQ(0)------------------------KSKRK--GHEYTNIKYSLTDQT-SGDQ--SPLPPCTPTP----PCAE(2)
Mouse SHP-2        VDCDIDVPKTIQMVRSQRSGMVQTEAQYRFIYMAVQHYIETLQRRIEEEQ(0)------------------------KSKRK--GHEYTNIKYSLVDQT-SGDQ--SPLPPCTPTP----PCAE(2)
Cattle SHP-2       VDCDIDVPKTIQMVRSQRSGMVQTEAQYRFIYMAVQHYIETLQRRIEEEQ(0)------------------------KSKRK--GHEYTNIKYSLTDQA-SGDQ--SPLPPCTPTP----SCAE(2)
Platypus SHP-2     VDCDIDVPKTIQMVRSQRSGMVQTEAQYRFIYMAVQHYIETLQRRIEEEQ(0)------------------------KSKRK--GHEYTNIKYSLADQT-SGDQ--XPLPPCTPTP----TCPE(2)
Chicken SHP-2      VDCDIDVPKTIQMVRSQRSGMVQTEAQYRFIYMAVQHYIETLQRRIEEEQ(0)------------------------KSKRK--GHEYTNIKYSLSDQT-SGDQ--SPLPPCTPTP----TCPE(2)
Goose SHP-2        VDCDIDVPKTIQMVRSQRSGMVQTEAQYRFIYMAVQHYIETLQRRIEEEQ(0)------------------------KSKRK--GHEYTNIKYSLSDQT-SGDQ--SPLPPCTPTP----TCPE(2)
Lizard SHP-2       VDCDIDVPKTIQMVRSQRSGMVQTEAQYRFIYMAVQHYIETLQRRIEEEQ(0)------------------------KSKRK--GHEYTNIKYSLADQA-GGDQ--SPLPPCTPTP----TCPE(2)
Turtle SHP-2       VDCDIDVPKTIQMVRSQRSGMVQTEAQYRFIYMAVQHYIETLQRRIEEEQ(0)------------------------KSKRK--GHEYTNIKYSLSDQT-SGDQ--SPLPPCTPTP----TCPE(2)
Frog SHP-2         VDCDIDVPKTIQMVRSQRSGMVQTEAQYRFIYMAVQHYIETLQRRIEEEQ(0)------------------------KSKRK--GHEYTNIKYSLSEQA--GDQ--SPLPPCTPTPLPTPTLPE(2)
Lungfish SHP-2     VDCDIDVPKTIQMVRSQRSGMVQTEAQYRFIYMAVQHYIETLQRRIEEEQ(0)------------------------KTKMK--GREYTNIKYSLSDLT--GDL--SPLPPGTPTS----ACTE(2)
Shark SHP-2        VDCDIDVPKSIQMVRSQRSGMVQTEAQYRFIYMAVQHYIETLQRRIEEEQ(0)------------------------KSKIK--GREYTNIKYSLSDLTCGGDQ--SPFPPCTPNP----ACTE(2)
Skate SHP-2        VDCDIDVPKSIQMVRSQRSGMVQTEAQYRFIYMAVQHYIETLQRRIEEEQ(0)------------------------KSKIK--GREYTNIKYSLSDLSCGGDQ--SPFPPCTPNP----VCTE(2)
Bichir SHP-2       VDCDIDVPKTIQMVRSQRSGMVQTEAQYRFIYMAVQHYIETLQRRILEEQ(0)------------------------KSKIK--GREYTNIKYSLSDMT-AGDQ--SPIPPCTPTP----SCTD(2)
Reedfish SHP-2     VDCDIDVPKTIQMVRSQRSGMVQTEAQYRFIYMAVQHYIETLQRRILEEQ(0)------------------------KSKIK--GREYTNIKYSLSDMT-AGDQ--SPIPPCTPTP----SCTD(2)
Sturgeon SHP-2     VDCDIDVPKSIQMVRSQRSGMVQTEAQYRFIYMAVQHYIETLQRRIEEEQ(0)------------------------KSKIK--GREYTNIKYSLTDLS-GGDQ--SPLPPCTPTP----VYAD(2)
Paddlefish SHP-2   VDCDIDVPKSIQMVRSQRSGMVQTEAQYRFIYMAVQHYIETLQRRIEEEQ(0)------------------------KSKIK--GREYTNIKYSLTDLS-GGDQ--SPLPPCTPTP----VYAD(2)
Gar SHP-2          VDCDIDVPKTIQMVRSQRSGMVQTEAQYRFIYMAVQHYIETLQRRILEEQ(0)------------------------KSKIK--GREYTNIKYSLSDLT-GGDQ--SPLPPCTPTS----ACAE(2)
Bonytongue SHP-2   VDCDIDVPKSIQMVRSQRSGMVQTEAQYRFIYMAVQHYIETLQRRIEEEQ(0)------------------------KSKIK--GREYTNIKYSLSDLT-SGDQ--SPLPPCTPTP----TCAD(2)
Tarpon SHP-2       VDCDIDVPKTIQMVRSQRSGMVQTEAQYRFIYMAVQHYIETLQRRIEEEQ(0)------------------------KSKIK--GREYTNIKYSLSDLS-GGDQ--SPLPPCTPTP----TCAD(2)
Weatherfish SHP-2 VDCDIDVPKTIQMVRSQRSGMVQTEAQYRFIYMAVQHYIETLQRRIEEEQ(0)------------------------KSKIK--GREYTNIKYSLSDLS-GGDQ--SPLPPCTPTP----TCVE(2)
Zebrafish SHP-2    VDCDIDVPKTIQMVRSQRSGMVQTEAQYRFIYMAVQHYIETLQRRIEEEQ(0)------------------------KSKIK--GREYTNIKYSLSDLS-GGDQ--SPLPPCTPTP----TCAD(2)
Salmon SHP-2       VDCDIDVPKSIQMVRSQRSGMVQTEAQYRFIYMAVQHYIETLQRRIEEEQ(0)------------------------KSKIK--GREYTNIKYSLSDLT-GGDQLQSPLPPCTPSP----TCAE(2)
Perch SHP-2        VDCDIDVPKTIQMVRSQRSGMVQTEAQYRFIYMAVQHYIETLQRRIEEEQ(0)------------------------KSKIK--GREYTNIKYSLSDLT-GGEQ--SPLPPCTPIP--TPTCTE(2)
Medaka SHP-2       VDCDMDVPKTIQMVRSKRSGMVQTEAQYRFIYMAVKHYIETLQRRIEEEQ(0)------------------------KSKIK--GREYTNIKYSLSDLT-AGEQ--GPLTPCTPLP--TPTCTE(2)
Mummichog SHP-2    VDCDIDVPKTIQMVRSQRSGMVQTEAQYRFIYMAVQHYIETLQRRIEEEQ(0)------------------------KSKIK--GREYTNIKYSLSDLS-GGEQ--SPLPLCSPIP--TPVCTE(2)
Lemur SHP-2L       LDCDIDIPKTIQLVRRQRSGMVQTEAQYKFVYLALQRYIQGEQLRLRE-Q---VGPGRGRSGACAGVPVVTRGPCPQREPPE--GRDYLNVRAAPAGPGRSRRPAP
Tupaia SHP-2L      LNCDIDIPKTIQMVRRQRSGMVQTEAQYKFVYLALQRYIQSEQLRLRE-Q(0)------------------------REPPE--EREYLNVGPAPANPSSSPRLQQS--R-PA-A(2)
Cattle SHP-2L      LDCDIDVPKTIQLVRRQRSGMVQTEAQYKFVYLALQRYIQGEQLRLRK-P---VGAGPGRGGACAGVTMVTRGPSPQRERQE--ERGSLNLGVSPVDPGCSPGPAPS--RAPA-A(2)
Platypus SHP-2L    LDCDIDIPKTIQMVRRQRSGMVQTEAQYKFVYMALQQFIETERKRLEEEQ(0)------------------------RSKRK--EREYLNIRYQPLDRARGKGNLPP-ARAQA-G(2)
Chicken SHP-2L     LDCDIDIPKTIQMVRRQRSGMVQTEAQYKFVYMAVQQFIEAEQKRLEEEQ(0)------------------------RSKRK--ERDYLNIGYSPVEKGRAKGQPPS-PRAQP-V(2)
Goose SHP-2L       LDCDIDIPKTIQMVRRQRSGMVQTEAQYKFVYMAVQQFIEAEQKRLEEEQ(0)------------------------RNKRK--ERDYLNIGYSPMEKGRAKGQPPS-PRAQS-V(2)
Lizard SHP-2L      LDCDIDIPKTIQMVRRQRSGMVQTEAQYKFVYMAVQHYIATEQKELEEEQ(0)------------------------RSMRK--EKEYLNIRYPPMEQPRGKARPPS-PRVQM-G(2)
Turtle SHP-2L      LDCDIDIPKTIQMVRRQRSGMVQTEAQYKFVYMAVQQYIETEQKRLEEEQ(0)------------------------RNKRK--ERDYLNIRHPPMDKARAKGQPPP-PRVSV-V(2)
Frog SHP-2L        LDCDIDIPKTIQMVRQQRSGMVQTEAQYKFIYMAVQKYIEVRKRHLEEEQ(0)------------------------MGKLK--EREYTNIQYSVGERARTKAQPAA-AQSQH-R(2)
Lungfish SHP-2L    LDCDIDIPKTIQMVRRQRSGMVQTEAQYKFIYMAVQQYIETVQKRLEEEQ(0)------------------------RNKTK--EREYSNIRYPPMEKPKSRPQTPS-ARTSP-M(2)
Shark SHP-2L       LDCDIDIPKTIQMVRKQRSGMVQTEAQYKFIYMAVQQYIETVQKRLQEEQ(0)------------------------KNKTK--EREYSNIHYL-TDKAKNKRYPAP-SRAFS-T(2)
Skate SHP-2L       LDCDIDIPKTIQIVRKQRSGMVQTEAQYKFIYMAVQQYIETVQRRLQEEQ(0)------------------------KNKTK--DREYSNIRYLPTEVKTKR-QAGT-SRDFS-L(2)
Bichir SHP-2L      LDCDIDIPKTIQMVRRQRSGMVQTEAQYKFIYMAVQQYIDTAQKRLEEEQ(0)------------------------RNKIK--EREYSNIRYPPMENTKLRPQTPT-PRGTS-A(2)
Reedfish SHP-2L    LDCDIDIPKTIQMVRRQRSGMVQTEAQYKFIYMAVQQYIDTAQKRLEEEQ(0)------------------------RNKIK--EREYSNIRYPPMENTKLRPQTPT-PRGTS-A(2)
Sturgeon SHP-2L    LDCDIDVPKTIQMVRRQRSGMVQTEAQYKFIYMAVQQYIDTVQKRLEEEQ(0)------------------------RNKTK--EREYSNIRYPPTENTKLWPHTPT-SRTSS-M(2)
Paddlefish SHP-2L LDCDIDVPKTIQMVRRQRSGMVQTEAQYKFIYMAVQQYIDTVQKRLEEEQ(0)------------------------RNKTK--EREYSNIRYPPTENTKLWPHTPT-SRTSS-M(2)
Gar SHP-2L         LDCDIDIPKTIQMVRCQRSGMVQTEAQYKFIYMAVQQYIDTVQKRLEEEQ(?)------------------------RNKTK--EREYSNIRYPPTENAKQKPGVPA-SQTPP-M(?)
Bonytongue SHP-2L LDCDIDIPKTIQMVRRQRSGMVQTEAQYKFIYMAVQQYIDTAQKRLDEEQ(0)------------------------RNKMK--EREYSNIKYPQMANAKVKANVGS-SRSSNSV(2)
Tarpon SHP-2L      LDCDIDIPKTIQMVRQQRSGMVQTEAQYKFIYMAVQQYIDTAQKRLEEEQ(0)------------------------RNKMK--EREYSNIKYPQMTNAKPKPNMTI-SRSSSSV(2)
Weatherfish SHP-2LLDCDIDIPKTIQRVRQQRSGMVQTEAQYKFIYMAVQQYIDTAQKRLEEEQ(0)------------------------RNKTK--EREYSNIRYPQMTNARAKPNMSS-SRTSS-V(2)
Zebrafish SHP-2L   LDCDIDIPKTIQRVRQQRSGMVQTEAQYKFIYMAVQQYIDTAQKRLEEEQ(0)------------------------RNKTK--EREYSNIKYPQMSNARAKPNMSS-SRSSS-V(2)
Salmon SHP-2L      LDCDIDIPKTIQRVRQQRSGMVQTEAQYKFIYMAVQQYIDTAQKRLEEEQ(0)------------------------RNKTK--EREYSNIKYPQMTNARSKPNMTCVSRSSS-V(2)
Perch SHP-2L       LDCDIDIPKTIQRVRQQRSGMVQTEAQYKFIYMAVQQYIDTAQKRLEEEQ(0)------------------------RNKM---EREYSNIKYPQMTNSRSKPNMAS-SRSSS-V(2)
Medaka SHP-2L      LDCDIDIPKTIQRVRQQRSGMVQTEAQYKFIYMAVQQHIDTAQKRLEEEQ(0)------------------------RNKMK--EREYSNIKYPQMTNSRSKQNTAS-SRSSS-V(2)
Mummichog SHP-2L   LDCDIDIPKTIQMVRQQRSGMVQTEAQYKFIYMAVQQYIDTAQKRLEEEQ(0)------------------------RNKMK--EREYSNIKYPQMTNSRSKHNMAS-SRSSS-V(2)
```

Exon-14

```
                   .560           .570      .580      .590
Human SHP-1       HKED----VYENLH-TKNKREEKVKKQRSADKEKSKG------SLKRK
Lemur SHP-1       HKED----VYENLQ-SKNRKEEKVRKQRSADKEKSKG------SLKRK
Tupaia SHP-1      HKED----VYENLH-SKGKKEERVKKQRSADREKGKG------SLKRK
Mouse SHP-1       HKEE----VYENVH-SKSKKEEKVKKQRSADKEKNKG------SLKRK
Cattle SHP-1      HRED----VYENVH-SKNKKEEKVKKQRSADKEKNKG------SLKRK
Platypus SHP-1    QKED----VYENLQG---KAKKEVKKQRSADKEKNKG------SLRKK
Chicken SHP-1     QKET----IYENLG----KKEEKVRKQLSSEK-KLKS------SLKKK
Goose SHP-1       QKEES--TVYENLG----KKEEKVRKQLSSDK-KLKG------SLKKK
Lizard SHP-1      QKEEP--TLYENLDKAKAKKEEKGTKPLGKEK-KPKG------SLKKK
Turtle SHP-1      QKEEP--TVYENLENTRGKKEERVRKQRSSEKEKLKG------SLKKK
Frog SHP-1        NQPEG--TVYENLNAGKQK-EEVVKK-KSSE--KQRS------SVKKK
Lungfish SHP-1    QKEES--QVYENLGSLKGKKEDKMKKQKSSEDKVKASSK--TSSIKKK
Shark SHP-1       CNED----VYENLS--KSKKDEGVRKQKS-EERVKGAKVK-PGSVKKK
Skate SHP-1       ADE---------SG--RTRKEEKVRKQRSAEER-KA------GSVRKK
Bichir SHP-1      NKDD----TYENAGLKGGKKDDKMKKQKS-EEKGKAG--N-PGSVRKR
Reedfish SHP-1    NKDD----TYENAGLKGGKKDDKVKKQKS-EEKGKAG--K-PGSVRKR
Sturgeon SHP-1    NKEE----VYENLGSKGGKKEDKVKKQKS-EEKDKAKSGK-SGSVRKR
Paddlefish SHP-1  KKED----VYENLSLKGGKKEDKVKKQKS-EEKDKAKSGK-SGSVRKR
Gar SHP-1         NKEE----VYENL---GGKGKKDVKKQKS-EEK------K-SGSVRKR
Bonytongue SHP-1  NKED----VYENL---GLKGKKDVKKQKS-EEK------K-SGSVRKR
Tarpon SHP-1      NKED----IYENL---GAKGKKDVKKQKS-EEK------K-SGSVRKR
Weatherfish SHP-1 KNED----VYENL---GAKGKKDVKKQKS-EDK------K-SGSVRKR
Zebrafish SHP-1   KNED----VYENL---GAKGKKDVKKQKS-EEK------KGGGSVRKR
Salmon SHP-1      NKEQE---LYENL----AKGKKDVKKQKS-EEK------K-SGSVRKR
Perch SHP-1       NKED----VYENL----SKGKKDAKKSKT-DKK--------SGSVRKR
Medaka SHP-1      NKED----VYENL----SKAKKEGKKAKP-DKK--------SGSVKKR
Mummichog SHP-1   NKED----VYENL----SKGKKDMKKSKS-EKK--------TGSVKKK
-----------------------------------------------------------------
                           .Y580
Human SHP-2       MREDSA-RVYENVGLMQQQ-KSFR
Lemur SHP-2       MREDNA-RVYENVGLMQQQ-KSFR
Tupaia SHP-2      MREDNA-RVYENVGLMQQQ-KSFR
Mouse SHP-2       MREDSA-RVYENVGLMQQQ-RSFR
Cattle SHP-2      MREDNA-RVYENVGLMQQQ-KSFR
Platypus SHP-2    MRDDSA-RVYENVGLMQQQ-KSFR
Chicken SHP-2     MREDSA-RVYENVGLMQQQ-KSFR
Goose SHP-2       MREDSA-RVYENVGLMQQQ-KSFR
Lizard SHP-2      MREDNAMRVYENVGLMQQQ-KSFR
Turtle SHP-2      MREDSA-RVYENVGLMQQQQKSFR
Frog SHP-2        MREDAG-RVYENVGLMQQQ-KSFR
Lungfish SHP-2    MRDDSA-RVYENVGVMQQQ-KSFR
Shark SHP-2       LKDEGG-RIYENVGLMQAQ-KGHR
Skate SHP-2       MKDEGG-RIYENVGLMQQQ-KGHR
Bichir SHP-2      MRDDNS-RVYENVGLMAQQ-KR
Reedfish SHP-2    IRDDNS-RVYENVGLMAQQ-KR
Sturgeon SHP-2    VRDDSS-RVYENVGLMQQQ-KSFR
Paddlefish SHP-2  VRDDSS-RVYENVGLMQQQ-KSFR
Gar SHP-2         MRDDSS-RVYENVGLMQQQ-KSFR
Bonytongue SHP-2  FRDEGS-RVYENVGLMQQQ-KSFR
Tarpon SHP-2      MRDDSS-RVYENVGLMQQQ-KSFR
Weatherfish SHP-2 MRDDSS-RVYENVGLMQQQ-KSHR
Zebrafish SHP-2   MRDDSS-RVYENVGLMQQQ-KSHR
Salmon SHP-2      MREDCS-RVYENVGLMQQQKSYR
Perch SHP-2       MREDSS-RVYENVGLMQQQKSYR
Medaka SHP-2      MRDDSS-RVYENVGLMQQQKSFR
Mummichog SHP-2   MRDDSS-RVYENVGLMQQQKTFR
-----------------------------------------
Lemur SHP-2L
Tupaia SHP-2L     T-QEISGGVYENLLTLRR
Cattle SHP-2L     T-REAPRHEYENLQGLEP
Platypus SHP-2L   M-DDDPASVYENLSLQGTKVFG-AGNTGR
Chicken SHP-2L    A-DDESASVYENLNIKSPKVSG-MSNTGR
Goose SHP-2L      V-DDESASVYENLNIKSPKVSG-MSNTGR
Lizard SHP-2L     V-DDESAAVYENLNVKAAKVSG-ITHSGR
Turtle SHP-2L     D-DDDSATVYENLNIKSSKVSG-MNNAGR
Frog SHP-2L       V-SDDAACVYENLNIRSTHMSS-TSNAGR
Lungfish SHP-2L   V-NEDPGCVYENLNIKNPKVVG-ISSARR
Shark SHP-2L      L-KE-PSCLYENLNIKSPKSSG-SSNTKR
Skate SHP-2L      S-KEESSCLYENLNIKTSKASG-GSNAKR
Bichir SHP-2L     L-EDDSTGVYENLNLKNPKVPG-ISNTRR
Reedfish SHP-2L   L-EDDSTGVYENLNLKNPKVPG-ISNTRR
Sturgeon SHP-2L   V-NEDAAGVYENLNIKNPKGPG-ISNTRR
Paddlefish SHP-2L V-NEDTAGVYENLNIKNPKCPG-ISNTRR
Gar SHP-2L        V-YDDSASVYENLNIKKPKASG-ISNARR
Bonytongue SHP-2L M-NDDPSNVYENLNVKTPKASG-SSNTRR
Tarpon SHP-2L     M-NDDSASVYENLNIKNPKTSG-STNSRR
Weatherfish SHP-  M-NDDS-SVYENLNIKNPKGSA-SANTRR
Zebrafish SHP-2L  M-NDDS-SVYENLNIKNPKGST-SSNTRR
Salmon SHP-2L     VTNDDPSAVYENLNIKNPSTSGNSSNTRK
Perch SHP-2L      MTNDDSSSVYENINFKSPQTSF-SSNTRR
Medaka SHP-2L     MTNEDSTGVYENINFKSPQASF-SSNTRR
Mummichog SHP-2L  MTNDDSSSVYENINFRTPQTSF-SSNTRR
```

(B) Phylogenetic tree.

The deduced SHP-1, SHP-2, and SHP-2L amino acid sequences shown in (A)—and, as outgroups, human PTPN1 (GenBank accession NP 002818), and human PTPN12 (NP 002826)—were aligned by ClustalW (Thompson et al., 1994, doi: 10.1093/nar/22.22.4673) in MEGA7 (Kumar et al., 2016, doi: 10.1093/molbev/msw054). Then, in MEGA7, evolutionary history was inferred by using the Neighbor-Joining method (Saitou and Nei, 1987, doi: 10.1093/oxfordjournals.molbev.a040454). The optimal tree with the sum of branch length = 7.90947389 is shown. The percentage of replicate trees in which the associated taxa clustered together in the bootstrap test (500 replicates) are shown next to the branches (Felsenstein, 1987, doi: 10.1111/j.1558-5646.1985.tb00420.x). The tree is drawn to scale, with branch lengths in the same units as those of the evolutionary distances used to infer the phylogenetic tree. The evolutionary distances were computed using the Poisson correction method (Zuckerkandl and Pauling, 1965, doi: 10.1016/B978-1-4832-2734-4.50017-6) and are in the units of the number of amino acid substitutions per site. The analysis involved 83 amino acid sequences. All ambiguous positions were removed for each sequence pair. There were a total of 851 positions in the final dataset.

Phylogenetic tree by Neighbor-joining method

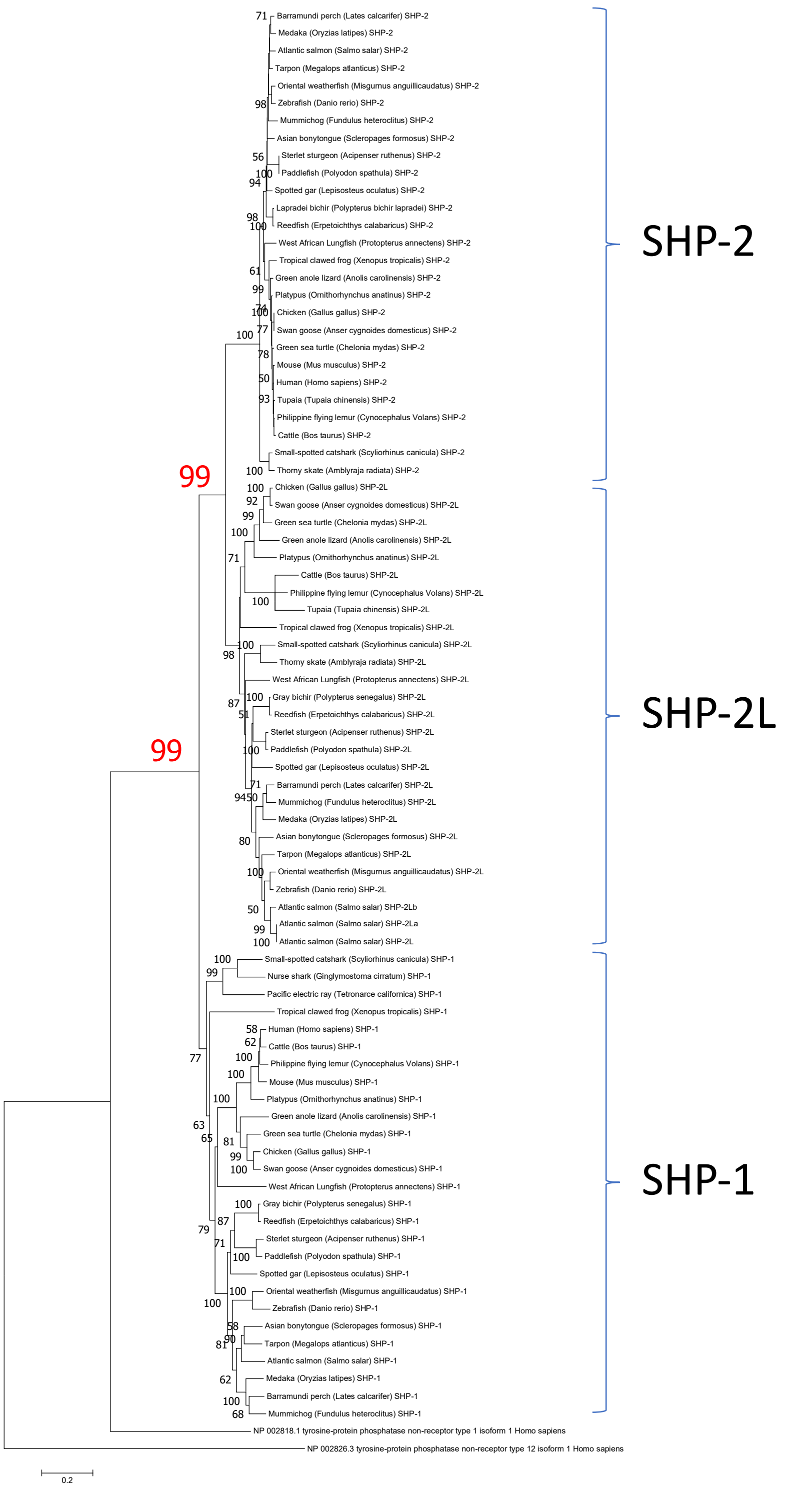

Supplement: Supplementary file 3 [file DataSheet3.pdf]
