# Supplementary material for: PD-1 is conserved from sharks to humans: new insights into PD-1, PD-L1, PD-L2, and SHP-2 evolution
Source: Front Immunol. 2025 May 28;16:1573492. doi: 10.3389/fimmu.2025.1573492 (PMC12151841; doi:10.3389/fimmu.2025.1573492)

Supplementary file 4

Expression patterns of *CD3Z*, *PD-1*, *SHP-1*, *SHP-2* and *SHP-2L* in fish and mammalian species. UMAP visualization of gene expression patterns (upper) and the distribution of normalized expression levels in corresponding UMAP clusters (lower) in Nurse shark (*Ginglymostoma cirratum*) spleen (**A**), Zebrafish (*Danio rerio*) spleen (**B**) and Cattle (*Bos taurus*) PBMC (Peripheral Blood Mononuclear Cells) (**C**).

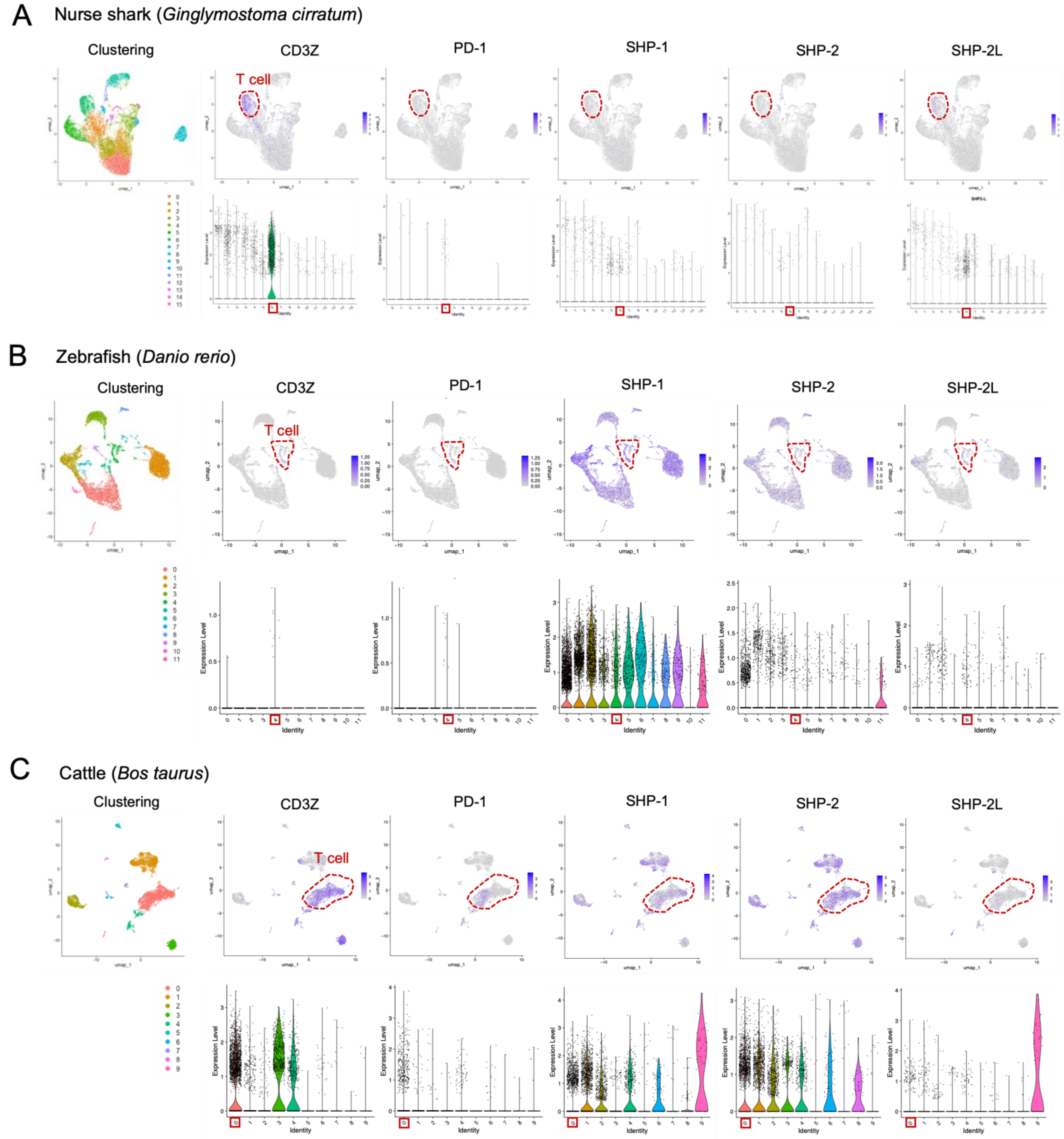

Supplement: Supplementary file 4 [file DataSheet4.pdf]
