# Supplementary material for: PD-1 is conserved from sharks to humans: new insights into PD-1, PD-L1, PD-L2, and SHP-2 evolution
Source: Front Immunol. 2025 May 28;16:1573492. doi: 10.3389/fimmu.2025.1573492 (PMC12151841; doi:10.3389/fimmu.2025.1573492)

Supplementary file 5 UMAP plots of scRNA-seq data (A and C), with colors indicating distinct clusters, and corresponding heatmaps (B and D) showing the top 20 differentially expressed genes (markers) for each cluster, selected based on adjusted *p*-values and log fold change. Data are from the spleen of Mouse (*Mus musculus*) (A and B) and Atlantic salmon (*Salmo salar*) (C and D).

Mouse (*Mus musculus*)

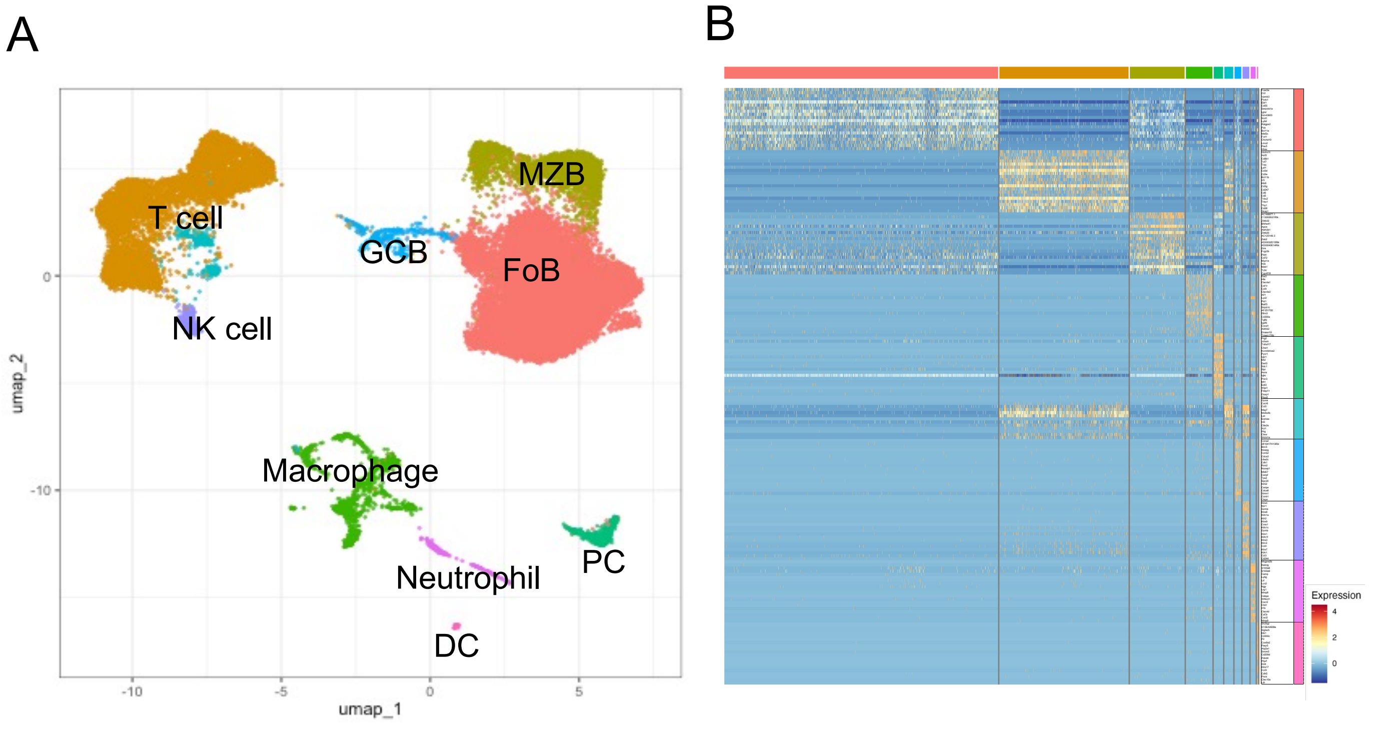

Atlantic salmon (*Salmo salar*)

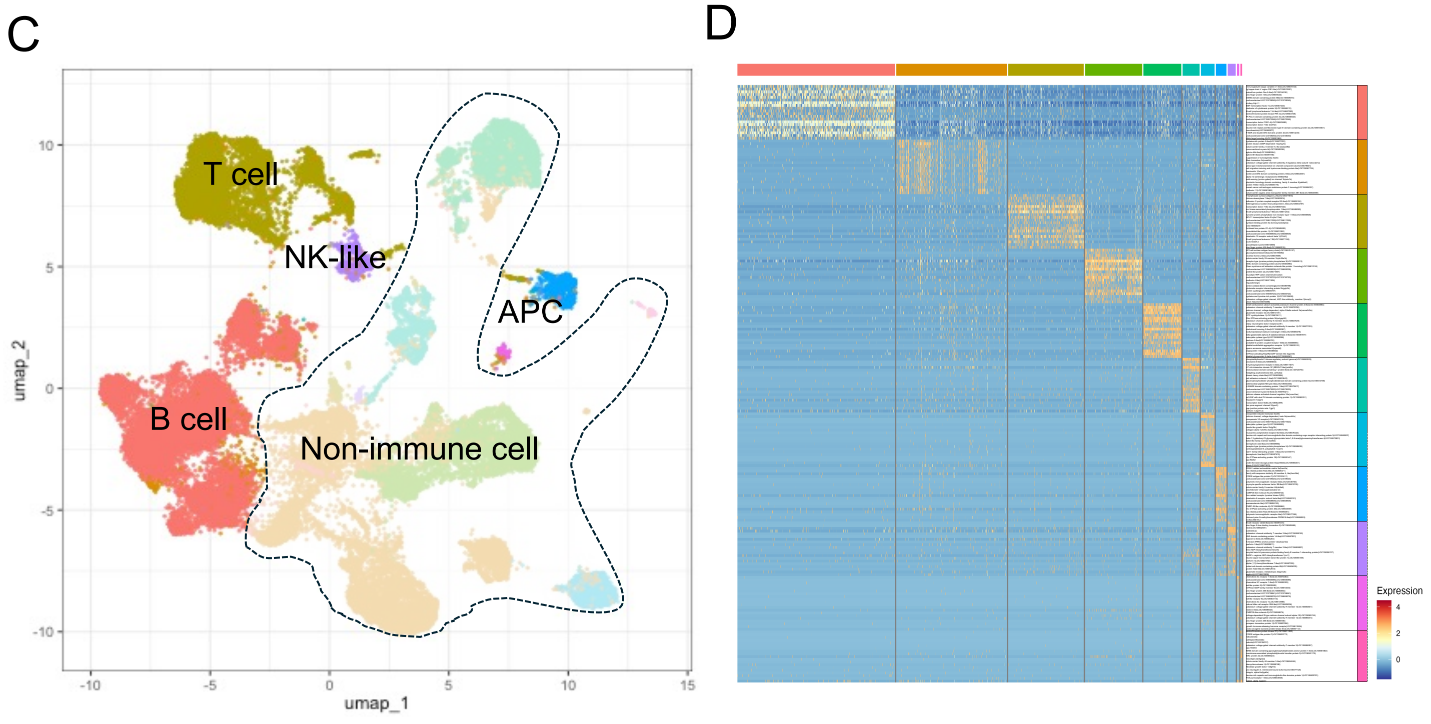

Supplement: Supplementary file 5 [file DataSheet5.pdf]
