# Supplementary material for: PD-1 is conserved from sharks to humans: new insights into PD-1, PD-L1, PD-L2, and SHP-2 evolution
Source: Front Immunol. 2025 May 28;16:1573492. doi: 10.3389/fimmu.2025.1573492 (PMC12151841; doi:10.3389/fimmu.2025.1573492)
Supplement: Supplementary file 6 [file DataSheet6.pdf]

Supplementary file 6

Gene sequences used in scRNA analysis

(A) A table of ortholog gene symbols and IDs analyzed for each species, and (B) the nucleotide sequences used for the analysis of genes not annotated as genes in the investigated databases.

A

	Salmo salar		*Danio rerio*		*Mus musculus*		*Bos taurus*	
Orthologous Group Nam	Gene symbol	Entrez gene ID	Gene symbol	Entrez gene ID	Gene symbol	Entrez gene ID	Gene symbol	Entrez gene ID
BCL11A	LOC106593355	106593355			Bcl11a	14025		
BLNK	blnk	106576789			Blnk	17060		
CD28	LOC106575093	106575093			Cd28	12487		
CD37	cd37	100195746			Cd37	12493		
CD3E	cd3e	100136516			Cd3e	12501	CD3E	281054
CD3G	LOC100136515	100136515			Cd3g	12502		
CD4	LOC100136502	100136502			Cd4	12504		
CD6	LOC106569780	106569780			Cd6	12511		
CD74A	cd74a	106604844			Cd74	16149		
CD79A	LOC106605673	106605673			Cd79a	12518		
CD8A	cd8a	100136450			Cd8a	12525		
CTLA4	LOC106586938	106586938			Ctla4	12477		
EBF1	LOC106567440	106567440			Ebf1	13591		
FOXP3B	LOC106572328	106572328			Foxp3	20371		
FYN	LOC106571317	106571317			Fyn	14360		
IGHD	ighd	106606764			Ighd	380797		
LAG-3	LOC106576634	106576634			Lag3	16768		
MEF2C	mef2cb	106580134			Mef2c	17260		
PAX5	LOC123744230	123744230			Pax5	18507		
PD-1	LOC106574172	106574172			Pdcd1	18566	PDCD1	613842
SHP-2	ptpn11a	106568295	ptpn11a	333979	Ptpn11	19247	PTPN11	533590
SHP-2Lb	LOC106566948	106566948						
SHP-2L (SHP-2La)	LOC106572187	106572187	ptpn11b	393426			LOC526769	526769
SWAP70	LOC106562355	106562355			Swap70	20947		
SYK	syk	100380388			Syk	20963		
TNFRSF4	LOC106582621	106582621			Tnfrsf4	22163		
TOX	LOC106599578	106599578			Tox	252838		
TSC22D3	tsc22d3	106612265			Tsc22d3	14605		
ZAP70	LOC106584369	106584369			Zap70	22637		

B

>Danio_rerio_CD3e

GGCGACTGAGGTTTTCCGCTTCCTGTAGAAACGCTCAGAGATGCTCAGAAACACACACACACACACACACGCTCAGAG
GAACACACACTGCAGCTGCCGAAGCGCTTCATAAACCCAACAGCAGCTCAGAGAACCTGCAGCAGCCATGAGGGTCTGGA
TCATCACTGCACTCATGCTGACGGCGACAGCGACTAATGCTCAAGATGTGGAGTTTCTGAGTGACGGTGTGGTTCTGACC
TGTCCAGGAGAAAAAAACTGGAAGTGGATAGATAAAAAAAGCACGAATAAAACCTTAACAATATCAGCTGATCAGGGAAC
AGTGAAGGGGGAGCAGAGATGTGAATATGAGGAGGATTCTAAAACACAGACACACGTCTTCTACATCGACGCCAAGGTGT
GCAAGGGCTGTGTAGAGTTTAATGGATGGTTGGCGTCGGCTGTTATATTTGGAGATCTGCTGCTGACTGGAGGAGTGATT
CTCCTCGTCTACCTGTGTGCCACCAAAAACAGCACACACACCCAGCAGAAGGCCTCGACTCCACGCTCTGCTAATCCTCC
TCGTCCCCCAAACCCGGACTATGAGAAACTGAACACTCAGACTCTCAGCAGCGGTCTGTACGCCGGCATCAACAAGACCT
GAGGAGCTTTACTGTAATGATGATTCACACACACTTCTGGAACGCTCCACGCCTCGTCTATGACTTCTGTAATGCTGAGT
GTGTGTGTGTGCGTGTGTGCGTGTGTGTGTGTGTGGAAAGTTCAGCTCACCTAACACCGGAAACAAGTATTTTCAAATGG
AAAGACTGAACAAACACTGCTGACATTTCTGCTTCTCTGAGTGTGTGTGTGTGTGTGTGTGTGTATATACAAGTGTGTAA
GTGTATAGTGTATATGTGTGTGTGTGTGTGTGTGTGTGTGTGTGCATGTGTGTGAGGTTGATTCAGTGGAGTAATATG
GAGCTGTAATGCGCTCAAACCTGCCGGAACTACTTTATCTGTGTGTTTATCTGTTAGTACGGGAGAGTTCCTCCTTAGCC
AATCAGAATGCAGCATTCAGCAGCCCTGCGCTATAAAGATGCATTTTCTCTTTCCTTCAGTGTGTGTGTGTGTGTGTGTG
TGTGTGTGTGTGTGTGTGTGTGTGTGTGTGTGTG

>Danio_rerio_PD-1

CGGCCTGCCTGCGCTCACTTGCTCTGTTTGGTCTGCAGGATGAAGCGTGACCTCAAGAAACTCTTACTTTTTTCATTCAT
GCTCATCTTGGTTCATAACGTGGATTCATTGAATGGTACATTGGGAAAAAACATCACTGTTCGATTCACGTTACAAGGTT
GTGATATTAACAGCGGGCACAGCAAATTAATCCTGTACAAGAATGGCCACAAGAAAGAAACCTGCAAACAAACTGAACCT
TTCTGCTCCACAAACTTTGTCTTCGGTGATGCGGAAAACAGCACGGTCACACTACACATCACGAACCTCACAATGGAGCA
CGGAGGAGAATATCATGTAGCAATGCGCACTACAAACTGCAATGCCGAAAAACCTCTGATTGAGAGCAATAAAGTATCCA
TCAGAGTCACATTACCCCGTACAACTACAGAGACTGTACCCACATCAGTGCATGAAAGCACATCGACAAGTCAAAAACCA
GAGACGCTGCAACAAAAGTCATTCATCGTTTTCTGCACTGCATCGGTGATAATAATCATATCTCTCTTTGTCGGAATACT
GTGCTGGTTTTATAGGAGCTATCCAAGGAAACAGGATGCTGAAAACCCAGCAGTTCAGAACAACAGAGCGACACAACAAG
GGCAGTGTGAGAGATCCGGCCCTGTGGTTGTCAGCTGTGTGGAGTACGGAGAATTAGACTTTCAAAGCAGACCTCAGAGA
GATGACAGAGGAAAAGCTGCTGAATCGACATCAAACGAGCAGGATGGAGTGGAATACGCAGCCATCATTTTCCCTCAGCA
GAAACAGACGCCTTGTGGGCGGATAAGAAATAAACAGCAAGTACCTGCCGTTAAACCATAGAGTCAGAAAAACCATTAAC
CTGTAACGCCTTGTCC

>Ginglymostoma_cirratum_CD3e_(from_Pettinello_R_et_al_2017)

GCAGTCTCCGCTTCTGTCTACCTGACCATGAAGACGCTGGGACAATGCTACATGACCGTTTTGACTGCGGCCATTCTGCT
GCTTGGGAGTTTAGCTTACGGGGTCGCAGAAAGTGTAAATGGATACGTGGCATACGATGAGCAGGCACTGAATTTAACTT
GCCCATCGGAGGGTGGTACAATTTCCTGGAAAGACGCGGATCGACATCAAGAGCTTGGCAGCGAATTAGTGCACCAGTTA
GAGTTCCGCAAAGGTATCGCACAGGGCCACGTCTATTGTGAAGCTGGTGGAAAAAAACATCATTTTTATATCAATGTGAA
AGTTTGTAATGACTGTATTAACCTCGATCCGGGCACGGTGATTGGGATCATTTGTGGAGACATGTTATTTACCCTCTTGG
TGGTCATGGGCGTTTATTGTTTTGCCAAGAAGCGAGGAGGAAACTCGAAAGAATTCCGCCATCCGCAGTTTGAGCCCATG
GAGACTCCGGCAGGTCGAAGTGCCAATGTGACAGCTTCCCATCAACAATCTCATTACGCGCCAATCAAAAGTGGACAGCG
AGATGTTTATGACAAATTGCAGCGATAAATCTGAGTGAGGAGGGAGTGAGAAATGGATTTCCTGGGTCTTCTACAACTCC
TGGATCTCCTAGACTTCCTGGATCTCCTGGACCTCTTGGATCTTCTGGATCTCTTAGACCTCCCTGATATCTCTCACTGT
CCTCCCCACAAGTACCCAACAATATAAACACAAATTTGTCTTAAACTCCTCGACTTGCCTGAAGGAAGTGCTTTGTACTT
TGTTGGGACTGTTTTCTTCTGACTAGTAGTAAGTCATTTGACCGTGGGATATCACTGATGCTGGTACATTTACAGTAAGA
ATGAGAGACATTGGTGAGAAACTCTTATGCTATTAGATATCTCAACAAAATTTCCTCTTACCCCTCTCTTCTCCACGGAG
AGCAATCCCAGCTTCTCCACTGTCTACACATTCCCTGAAGTCCCTCACCCTGGAACCATCTTTGTAAATCTCCTTGATCC
AGGTGTGGTGCCCACGATAGAGACTCCCAGCTGGACTCTAACCAGTGTTTATACTAGTTTAAGTTAAAATGCCTTTGATT
TTGACCTTTATGTTTGTTTTTAAAGCCAACACTCCATTTGCTTTCTTAAAAAAGTAGCTGACACTGTCGATGAATCAGTC
TGATGGCATTGTATCCTGTGAAGGTTCAGTCTTTTACTCAATGTCCCAGTCCCACTGTGCTCACTGGGAGAGCCATGTGA
TAGTGTTAATGCACATTCACAATCTTCCCCATTACGACACAAACCTTCAATCAGACAATGTGCCCAAGGCAAACCTGGTC
CGC

>Ginglymostoma_cirratum_PD-1_(GDQH01026610.1)

GTGAATCACTAATTCTATTACAGTATCCTATGACTATTCAGAAGCAGTGTGGTCAGTCAGCTCAGATCTACTGTGAACTT
CGCAATGGAAATTCTACTGAAGACATGAGACTTGTATGGTATAAATACACATATTATAGATCAAAGATTGGAGAAATCGA
TTTAGGGACAAGTATGTCCACAGCCATTGACCACATCCATTTACAATGGGACCTTGGCACCTACACAGCATCAATGTCAA
TAAGACATCTGGTGAAAAATGACTCTGGAGAATATGGTTGTGAACTGTTCTCATTTTCTGGCCCTCCGATTATAACGAAA
GCCAATGCAACATACCTCATTGTTACTGACAAACAGCGGATTAGTCCACCATACAGAAACAACACATACAGAAATGAGAC

CGCAGAATTCCAAAATAAGAAAGCTAGAATTCAAATAATTGTTTCAGCGGTAATAGCTGCCTTTGTCATCATCTGCCTGC
TAATTTACATTTTATTCAGATACCGACCAAAAAAACAAGGCCCTAACGCCAATCCTCCTCCAGCAGATGTCGGTTGCCAG
AAAGCAGAGGATCCAATTTCTACAGTGTTCTGTCCTGACTATGCTGCACTACAGGTCCCCGGGCAAAATATTCGACAGAC
TCTAGCATCTTCAATTGCCTCTGATGATTCATACTACGCTACCATTGTATTTGCACCTCAACAACAAGCTACTGGTGTGC
AGAAAATAACTGTTGTTAACTAAGGGGAAACATGTTCGATATAGT

>Ginglymostoma_cirratum_SHP-2_(GIWU01214799.1)
GAGACAGAGAGAGAGAGAGACAGACAGACGCTGGGCGGGAAGTGCCTGAGCTCGAGTCAGGGCCCCTTGCCCCATAGGAG
CTGCGGCAGAGGCAGAGTGCCTACCAACTCTCCCACCCATCACCGGCGGAGGCAGAAAACAGCCGTCAGGCCAGTGTGGG
AGGGAGTGTCCTCCGTCCATCACCATGACATCCCGAAGGTGGTTTCACCCCAACATCACTGGAGTGGAGGCAGAGAACTT
GTTGCTGACTAGAGGAGTTGACGGGAGTTTCTTGGCTCGACCGAGTAAGAGTAATCCTGGTGACTTCACGCTTTCAGTGA
GGCGAAATGGGGCAGTAACGCACATTAAAATTCAGAATACAGGTGATTACTATGATTTGTATGGAGGAGAGAAGTTTGCT
ACACTGGCTGAGCTGGTGCAGTACTACATGGAGCACCATGGACAACTCAAGGAGAAAAATGGCGATGTCATTGAACTGAA
ATACCCACTAAACTGTGCTGATCCTACCTCTGAGAGGTGGTTCCATGGACACCTCTCAGGAAAAGAGGCAGAGAAGCTGC
TGACAGAAAAAGGAAAACCAGGAAGTTTCCTTGTGCGAGAGAGCCAAAGCCATCCAGGAGATTTTGTTCTGTCTGTGAGG
ACTGGCGATGATAAAGGAGATAGCAGTGAGGGAAAGCCAAAGGTCACTCATGTAATGATCCGATGCCAGGATGGGAAATA
TGATGTTGGTGGAGGGGAGAAGTTTGATTCCTTGACAGACCTGGTGGAGCACTACAAAAAGAACCCTATGGTGGAAACCC
TGGGCACTGTCTTGCAGCTCAAGCAGCCGTTAAACACAACACGAATTAATGCAGCTGAGATTGAAAGCCGAGTGAGGGAA
CTGAGCAAACCAGCGGAGATGATGGACAAGGTGAAGCAAGGTTTCTGGGAAGAGTTTGAGACACTACAACAACAAGAGTG
TAAACTTCTCTACAGTCGAAAGGAAGGACAGCGACTAGAAAATAAAAACAAGAATCGCTACAAGAACATCCTGCCCTTCG
ATCACACTCGAGTGGTCCTACATGATGGAGACGTGACTGAATCTGGTTCTGATTACATCAATGCCAATTTCATAACGCAT
GACATTGAAAGCAATCGAACCACTACAAAACCAAAGAAGAGTTACATTGCTACGCAGGGCTGTTTGCAGAACACGGTGAA
TGACTTCTGGAGGATGGTTTTTCAAGAAAACTCAAGTGTAATTGTCATGACTACAAAAGAAGTGGAACGAGGAAAAAGCA
AATGTGTGAAATACTGGCCAGATGAAATGTCCCTGAAGGAATATGGTGGTATGCGTGTCAGGAATGTGCGAGAGAGCCCA
GCACACGATTATATCTTGAGGGAGCTCAAACTCTCTAAAGTAGGACAGGGATGCACTGAAAGGACTGTGTGGCAATATCA
TTTCAAAACGTGGCCAGATCATGGTGTTCCCAGTGATCCAGGTGGTGTTCTGGATTTTCTGGAAGAAGTAAATATGAAAC
AGGAGAACATCCCAGAAACTGGGCCAATTGTTGTTCATTGCAGTGCAGGGATTGGGCGCACAGGAACCTTTATTGTGATT
GACATTCTGATTGACATCATCAGAGAGAAAGGTGTGGACTGTGACATTGATGTTCCAAAGAGTATCCAGATGGTACGGTC
TCAGCGTTCTGGGATGGTTCAGACAGAGGCCCAATACAGGTTCATTTACATGGCTGTTCAGCACTATATTGAAACCCTGC
AGCGTCGCATCGAAGAGGAACAGAAAAGCAAGATTAAAGGCCGTGAATACACAAACATCAAATATTCCTTATCAGACCTA
ACGTGTGGAGGAGATCAGAGTCCTTTCCCACCCTGCACACCCAATCCAGGGTGCACAGAGATGAAAGATGAGGGTGGCCG
GATCTATGAAAATGTTGGCTTGATGCAGCAGCAAAAGGGCCATAGATGAGATAATACCAGTGTTCGGAACAGAATAGAAA
GGAACAAGTGGCTGTGAAAAGCAGAGGAGAAATGGAAATACGTCAGATGTGAGTGGAATGAAAAATGACCGGCGAAGGTT
TAGTTCAGAGTTTTGTGGAATGGATTTTCCCCCTTCCCCACATTTTAACACTAAAAGTGAAAAGTAACTTCCCATATTGC
TTTCTAAATGCCATTCTCTGTGCTAGGAAGTGAAGCATCACCTATAATTTATGCATCATTCACATCGCTTTTCAGTATTT
TACTGACGTGAAACCCAAGTGCCTTTTTACAGTCACAGTC

>Ginglymostoma_cirratum_SHP-2L_(GIWU01218728.1)
GCGGTGGATCCATCCAGCTTCTTTTAACAGCCATGACATCCCGGAGGTGGTTCCATCCCAATATCACAGGGATTGAAGCT
GAGCGCCTTTTACTGACAAGAGGTGTTCATGGAAATTTCCTAGCTCGACCCAGCAAAAGTAATCCTGGGGATTTCACTTT
ATCCGTTAGGCGAAATGAAGAAGTTACGCATATCAAGATCCAGAATACAGGGGACTATTATGACCTGTATGGAGGAGAAA
AGTTTGCAACATTGGCAGAATTGGTTCAGTATTACACAGAGCAACAAGGTCTCTTACGAGAGAAGAATGGAGACCTAATT
GAACTGAAGTATCCTCTGAATTGCCAAGATCCAACGTCTGAAAGGTGGTATCATGGACATCTATCGGGTAAAGATGCTGA
AAAACTCCTGACAGACAAAGGAAAACCTGGAAGTTTCCTGGTGCGTGAAAGTCTCAGTAAACCAGGAGATTTTGTTCTAT

CAGTGCTGACTAATGAAGAAAAATTTGAAAATGGAGACCGTAAGCCAAGAGTAACACATGTAATGATTCGGAATCAGGAT
GGGAAGTATGATGTTGGTGGAGGTGAAAGATTTGACACGCTTACTGACCTAGTGGACCACTACAAAAAAAACCCAATGGT
AGAAGTTACTGGAATTGTCGTTCACCTTAAACAGCCATTCAATGCTACACGAATAAATGCTGCAAACATAGAGAATCGAG
TCAAGGAACTCAATAAAACAGCAGATAACACTGAAAAGGCCAAGCAAGGTTTTTGGGAAGAGTTTGAGCTTCTTCAGCAA
CAAGAATATAAACTACTCTATAGCAGAAAGGAAGGACAAAGAATAGAAAACAAAAGCAAGAACAGATACAAAAATATTTT
ACCATTTGATAATACCAGAGTTACACTGAAGGAAGTGGATGAAACCATATTAGGATCTGATTATATCAATGCTAATAACA
TCACAAATGGAAAAATTGGAGATGATTCCAAAAACTACATTGCAACACAAGGCTGTCTTCAGAATACAATCAATGATTTT
TGGAAGATGATTTATCAGGAAAACACACACGTCATTGTTATGACAACCAAAGAAGTAGAAAGAGGAAGGAATAAATGTTA
CCGTTATTGGCCAGATTTAGAAACAGCAAAACTGTACGGTACGATTTCTGTGCGAAATCTGAAAGAACGCCTGGCGCAAG
ATTACGTTGTGCGTGAACTGGAGGTTGTACAAACAGCCCGGAAGGAAGCTCCTAGACACACATGGCACTATCAGTATCTC
AGCTGGCCAGATCACGGAGTGCCCAATGAACCAGGAGGAGTACTGAGTTTCCTGGACCAAGTAAATCGGACACAGCAAAG
CATTCCAGATACTGGCCCTATTGTAGTGCACTGCAGTGCTGGAATTGGAAGAACAGGAACCATAATTGTTATAGATATGT
TGGTAGCTGACATTAACAGACAAGGTTTGGACTGTGATATTGACATCCCTAAAACGATTCAAATGGTACGAAAACAAAGA
TCAGGCATGGTACAGACAGAGGCTCAGTATAAATTCATATACATGGCTGTACAACAGTATATTGAGACAGTTCAAAAAAG
ACTACAGGAGGAGCAGAAAAACAAAACAAAGGAACGAGAGTATTCAAATATCCACTATCTTACGGACAAAGCAAAAAACA
AAAGATACGCTGCCCCATCTCGAGTTTTTTCCACGTTGAAAGAAGAACCATCCTGTCTATATGAAAATTTAAACATCAAA
AGCCCCAAATCGTCAGGAAGTAGCAACGCCAAGAGATAAAAGCACCAAGGGTAGAGAGGTTGTGTACAATAAGCACATTA
ACAATACACATTAACAGTACCCATTACTTAAAAATTCTGTTTATAATGCACATACAAAATAGTAACATATGAAAATACAG
GTGTAAGTGTGATCATGTACATTGTTGTGAAATGTTCCTTGTTGAAGTGCATTCATGTCTTGGATGCTTTCTTCTCACCT
GAGCACCCTATCACCCCAACTTCTCTTATCTTAGGCGTTACTAGCATGGACCTCACCTTGTTCTTTTTTAGTAATTCTTT
CACAGTACCCTTTCCTTCTGTGTTGCATTATACCCAGTTATATCTCCCTGCCTTAACAGCAATATTTTCTTGCTGAATTC
CAATGTTGCCCGTATATTAGGTTTCCTCCGTGACCTCTGGATCTGGGTGGTGCTCTGTTATTCAGCACCGGTTACTCCTA
GCGGTCTCCAAAAATCTGCTTCTTTTCTCACTCCTAACATTTATGCAGCTGCCTGCCATTCTCTCTAATGGCCAAGTTCT
CCAGCTCCCTCCCCATCCTTTCTCTCGTTTTGATACACACCAACTAACTCTGGCACTAATCTCCTTCCCCAACCTGATCC
AGTGGGACAATAATTATTCATCCATCTGCATTGATATGTAGAATGTCTGTTCCCTCAAACAAGGGTTTCCCCAACTCATG
GATGGAGGTGATCTCCAGAAGTGGAGGTCAGTGTATTGGCACATGATCAGCGTGTGCATGATCACATAGCTTAGAGGTGA
CATTGCTGATAACCGTTACCCTCGTATAGCTCACTGCCTGATTATTTAGTCCACCATCCAAACCACAATAGATGGCAGTA
CTCTGATTGTATCACGTCTGTGTCCTTTATGCCCCATGACTTCATTTGTGCAATCCATCTTCACTATTCCTACCAATCCT
CATAGATTCCTTTAAGTTTGTCAGTTTCTACAATCCTACCTGTATTCATCATGCAAATCTCTTCCCCAGGAATTCAATTT
CTACACAGAAATGCTCTTTCTTTTCGAGCAAACTTCACCCTTCCACACGCATCATTGTGCTTCATCTGTTCTGAATGAAA
GAGTAGACAGCAGATAATTAACCTTGCCTTTCATATAAACTGCCCCACACACACTGCCTTCCTCTGGATCTTGCAATTTC
ACAACTCTCCAAGTCTTTACTAACTGGATCATTTCCTCACTCCAAACCTTGGGCTTACACCATCTCCTACCCCACCCATC
CTCGCTTTACCTTTTTTGCCGACACTACACTTCTCTCTCAAACACTTGCCTAAAACCACTGTATCTCACTTCCTGAGGGA
ATATTTTAGAAAACTGTATTGTCCTGTGATTTCAACTTCTGCAGGCACTGTGCCCCTGACCACCACCACCACATATAATG
TTTCTTTTCCCTATTAGCTTTGTGAAGATAAACAAATACTAAGTATGCCTTTAAAATTTTTAAGTTATTTTCAGTATCAA
CTTTTCTGTTATAAATACAAACTACTTTTGCATTTACGTTGCAAACTTTGTAGGAAAAATCTGATATTACTAATCACAAT
GATGAAGGATTGAATCATTATAAGATTTTGCAATGTTTGTTTAATACACGTTTCTGCCTTCTAATTTCCCCTGCCCCACC
TTTTAAACGTGAGCAGTAACGAGAATGATCCTGTCACCGTAATATCCGATTTTTTGCTCTCTCTAGAATTACTTCAGTTT
ACGTAAAGCTGCTGCCTGCATTTGTAATAAGAATTGCTGTGTCCCAGCTTGCCTCTGGATCTCTGTGCAGTGCAATTCTA
CAAACCAAATGTCTACAGAAAACTGCCTTTTAAAGACTTTAATTTATGAACAAGACTTTTAAAAAAAAAATTATATGTTTT
TTTTGTCAGCTTGTATTTATATATGTACTTTAAGTGCCTCTTCTTGAAATTGATAAATAATGGAGTTTAATTTTAAGATT
TCATGACTGTCCGAACAAGATATTGCATGCATTTAGGAAAAAAAACAGAAATAATTGACCTGAAAAACTGTAAGCCCATG
CATTTCATCATCTGCACTGCATGTTTTTAAGATGTAAAAAGGTAAATCATCATGACGAAAAGTTAAACTATGACAACCAA
ATTTTGCAGGTGTGTTTTTGTACCTACCTATAAAACAAGCTGCGCTGGTGGCACTGCCCAGCTTGACAGCAATTCTGATG

CTAAAGAAGCCAATCGCCTCAGCAGCAGTACCACCAGCAGGAACCATTGTGGTACTGCAACGAGAGGAGTTTTAAAAAAA

ACTACAGTAATTGTTATTCGTTGTTTCGCATTGCAATGATATTGTAACAACTTTGTAAATTTTAAAGTTGAGGAACATTT

GAGATTCTGTTATATTTGGAAGTGCCCAAGCTTTGACTGGCACAACTCATTGCACATTACAACCTTGATAAAATGGCACT

TCCCACATATTGTATTTGAAGTTGGTGGATATATTCACTAGCCAAAGATGAAGGTTTGTATGTTTTTCTTTAAAATTTAT

GTGTTGATCACGTCATTAATAAGGCATTCAGTGCTGAATGAAGACAATTTTCTTATAAGGCTTTTTTATATAATAAAAAA

TCTCTCAAACCAAATTTAGTCTAATATGAATGGTGGGTTCACTTACAATTTTGATATGTTTTCAGCGTTAATTGCCTCAA

TGTCAATGGGATGCTGTCG
